# The Global, Regional, and National Burden of Adult Lip, Oral, and Pharyngeal Cancer in 204 Countries and Territories: A Systematic Analysis for the Global Burden of Disease Study 2019

**DOI:** 10.1001/jamaoncol.2023.2960

**Published:** 2023-09-07

**Authors:** GBD 2019 Lip, Oral, and Pharyngeal Cancer Collaborators, Amanda Ramos da Cunha, Kelly Compton, Rixing Xu, Rashmi Mishra, Mark Thomas Drangsholt, Jose Leopoldo Ferreira Antunes, Alexander R. Kerr, Alistair R. Acheson, Dan Lu, Lindsey E. Wallace, Jonathan M. Kocarnik, Weijia Fu, Frances E. Dean, Alyssa Pennini, Hannah Jacqueline Henrikson, Tahiya Alam, Emad Ababneh, Sherief Abd-Elsalam, Meriem Abdoun, Hassan Abidi, Hiwa Abubaker Ali, Eman Abu-Gharbieh, Tigist Demssew Adane, Isaac Yeboah Addo, Aqeel Ahmad, Sajjad Ahmad, Tarik Ahmed Rashid, Maxwell Akonde, Hanadi Al Hamad, Fares Alahdab, Yousef Alimohamadi, Vahid Alipour, Sadeq Ali Al-Maweri, Ubai Alsharif, Alireza Ansari-Moghaddam, Sumadi Lukman Anwar, Anayochukwu Edward Anyasodor, Jalal Arabloo, Aleksandr Y. Aravkin, Raphael Taiwo Aruleba, Malke Asaad, Tahira Ashraf, Seyyed Shamsadin Athari, Sameh Attia, Sina Azadnajafabad, Mohammadreza Azangou-Khyavy, Muhammad Badar, Nayereh Baghcheghi, Maciej Banach, Mainak Bardhan, Hiba Jawdat Barqawi, Nasir Z. Bashir, Azadeh Bashiri, Habib Benzian, Eduardo Bernabe, Devidas S. Bhagat, Vijayalakshmi S. Bhojaraja, Tone Bjørge, Souad Bouaoud, Dejana Braithwaite, Nikolay Ivanovich Briko, Daniela Calina, Giulia Carreras, Promit Ananyo Chakraborty, Vijay Kumar Chattu, Akhilanand Chaurasia, Meng Xuan Chen, William C. S. Cho, Dinh-Toi Chu, Isaac Sunday Chukwu, Eunice Chung, Natália Cruz-Martins, Omid Dadras, Xiaochen Dai, Lalit Dandona, Rakhi Dandona, Parnaz Daneshpajouhnejad, Reza Darvishi Cheshmeh Soltani, Aso Mohammad Darwesh, Sisay Abebe Debela, Meseret Derbew Molla, Fikadu Nugusu Dessalegn, Mostafa Dianati-Nasab, Lankamo Ena Digesa, Shilpi Gupta Dixit, Abhinav Dixit, Shirin Djalalinia, Iman El Sayed, Maha El Tantawi, Daniel Berhanie Enyew, Daniel Asfaw Erku, Rana Ezzeddini, Adeniyi Francis Fagbamigbe, Luca Falzone, Getahun Fetensa, Takeshi Fukumoto, Piyada Gaewkhiew, Silvano Gallus, Mesfin Gebrehiwot, Ahmad Ghashghaee, Paramjit Singh Gill, Mahaveer Golechha, Pouya Goleij, Ricardo Santiago Gomez, Giuseppe Gorini, Andre Luiz Sena Guimaraes, Bhawna Gupta, Sapna Gupta, Veer Bala Gupta, Vivek Kumar Gupta, Arvin Haj-Mirzaian, Esam S. Halboub, Rabih Halwani, Asif Hanif, Ninuk Hariyani, Mehdi Harorani, Hamidreza Hasani, Abbas M. Hassan, Soheil Hassanipour, Mohammed Bheser Hassen, Simon I. Hay, Khezar Hayat, Brenda Yuliana Herrera-Serna, Ramesh Holla, Nobuyuki Horita, Mehdi Hosseinzadeh, Salman Hussain, Olayinka Stephen Ilesanmi, Irena M. Ilic, Milena D. Ilic, Gaetano Isola, Abhishek Jaiswal, Chinmay T. Jani, Tahereh Javaheri, Umesh Jayarajah, Shubha Jayaram, Nitin Joseph, Vidya Kadashetti, Eswar Kandaswamy, Shama D. Karanth, Ibraheem M. Karaye, Joonas H. Kauppila, Harkiran Kaur, Mohammad Keykhaei, Yousef Saleh Khader, Himanshu Khajuria, Javad Khanali, Mahalaqua Nazli Khatib, Hamid Reza Khayat Kashani, Mohammad Amin Khazeei Tabari, Min Seo Kim, Farzad Kompani, Hamid Reza Koohestani, G. Anil Kumar, Om P. Kurmi, Carlo La Vecchia, Dharmesh Kumar Lal, Iván Landires, Savita Lasrado, Caterina Ledda, Yo Han Lee, Massimo Libra, Stephen S. Lim, Stefan Listl, Platon D. Lopukhov, Ahmad R. Mafi, Rashidul Alam Mahumud, Ahmad Azam Malik, Manu Raj Mathur, Sazan Qadir Maulud, Jitendra Kumar Meena, Entezar Mehrabi Nasab, Tomislav Mestrovic, Reza Mirfakhraie, Awoke Misganaw, Sanjeev Misra, Prasanna Mithra, Yousef Mohammad, Mokhtar Mohammadi, Esmaeil Mohammadi, Ali H. Mokdad, Mohammad Ali Moni, Paula Moraga, Shane Douglas Morrison, Hamid Reza Mozaffari, Sumaira Mubarik, Christopher J. L. Murray, Tapas Sadasivan Nair, Sreenivas Narasimha Swamy, Aparna Ichalangod Narayana, Hasan Nassereldine, Zuhair S. Natto, Biswa Prakash Nayak, Serban Mircea Negru, Haruna Asura Nggada, Hasti Nouraei, Virginia Nuñez-Samudio, Bogdan Oancea, Andrew T. Olagunju, Ahmed Omar Bali, Alicia Padron-Monedero, Jagadish Rao Padubidri, Anamika Pandey, Shahina Pardhan, Jay Patel, Raffaele Pezzani, Zahra Zahid Piracha, Navid Rabiee, Venkatraman Radhakrishnan, Raghu Anekal Radhakrishnan, Amir Masoud Rahmani, Vahid Rahmanian, Chythra R. Rao, Sowmya J. Rao, Goura Kishor Rath, David Laith Rawaf, Salman Rawaf, Reza Rawassizadeh, Mohammad Sadegh Razeghinia, Nazila Rezaei, Negar Rezaei, Nima Rezaei, Aziz Rezapour, Abanoub Riad, Thomas J. Roberts, Esperanza Romero-Rodríguez, Gholamreza Roshandel, Manjula S., Chandan S. N., Basema Saddik, Mohammad Reza Saeb, Umar Saeed, Mohsen Safaei, Maryam Sahebazzamani, Amirhossein Sahebkar, Amir Salek Farrokhi, Abdallah M. Samy, Milena M. Santric-Milicevic, Brijesh Sathian, Maheswar Satpathy, Mario Šekerija, Subramanian Senthilkumaran, Allen Seylani, Omid Shafaat, Hamid R. Shahsavari, Erfan Shamsoddin, Mequannent Melaku Sharew, Javad Sharifi-Rad, Jeevan K. Shetty, K. M. Shivakumar, Parnian Shobeiri, Seyed Afshin Shorofi, Sunil Shrestha, Sudeep K. Siddappa Malleshappa, Paramdeep Singh, Jasvinder A. Singh, Garima Singh, Dhirendra Narain Sinha, Yonatan Solomon, Muhammad Suleman, Rizwan Suliankatchi Abdulkader, Yasaman Taheri Abkenar, Iman M. Talaat, Ker-Kan Tan, Abdelghani Tbakhi, Arulmani Thiyagarajan, Amir Tiyuri, Marcos Roberto Tovani-Palone, Bhaskaran Unnikrishnan, Bay Vo, Simona Ruxandra Volovat, Cong Wang, Ronny Westerman, Nuwan Darshana Wickramasinghe, Hong Xiao, Chuanhua Yu, Deniz Yuce, Ismaeel Yunusa, Vesna Zadnik, Iman Zare, Zhi-Jiang Zhang, Mohammad Zoladl, Lisa M. Force, Fernando N. Hugo

**Affiliations:** 1 School of Public Health, University of São Paulo, São Paulo, Brazil; 2 Institute for Health Metrics and Evaluation, University of Washington, Seattle; 3 Department of Data and Tooling, Sage Bionetworks, Seattle, Washington; 4 Department of Oral Medicine, School of Dentistry, University of Washington, Seattle; 5 Oral Medicine Clinic, School of Dentistry, University of Washington, Seattle; 6 Department of Oral and Maxillofacial Pathology, Radiology, and Medicine, College of Dentistry, New York University, New York, New York; 7 Department of Mathematics, University of California, Berkeley; 8 Department of Global Health, School of Public Health, Boston University, Boston, Massachusetts; 9 Pathology and Laboratory Medicine Institute, Cleveland Clinic, Cleveland, Ohio; 10 Tropical Medicine Department, Faculty of Medicine, Tanta University, Tanta, Egypt; 11 Department of Medicine, University of Setif Algeria, Setif, Algeria; 12 Laboratory Technology Sciences Department, Yasuj University of Medical Sciences, Yasuj, Iran; 13 Department of Banking and Finance, University of Human Development, Sulaymaniyah, Iraq; 14 Clinical Sciences Department, University of Sharjah, Sharjah, United Arab Emirates; 15 Department of Clinical and Psychosocial Epidemiology, University of Groningen, Groningen, the Netherlands; 16 Centre for Social Research in Health, University of New South Wales, Sydney, New South Wales, Australia; 17 Quality and Systems Performance Unit, Cancer Institute NSW, Sydney, New South Wales, Australia; 18 Department of Medical Biochemistry, Shaqra University, Shaqra, Saudi Arabia; 19 Department of Health and Biological Sciences, Abasyn University, Peshawar, Pakistan; 20 Department of Computer Science and Engineering, University of Kurdistan Hewler, Erbil, Iraq; 21 Department of Epidemiology and Biostatistics, Arnold School of Public Health, University of South Carolina, Columbia; 22 Geriatric and Long Term Care Department, Hamad Medical Corporation, Doha, Qatar; 23 Rumailah Hospital, Hamad Medical Corporation, Doha, Qatar; 24 Evidence-Based Practice Center Program, Mayo Clinic Foundation for Medical Education and Research, Rochester, Minnesota; 25 Health Research Center, Baqiyatallah University of Medical Sciences, Tehran, Iran; 26 Health Management and Economics Research Center, Iran University of Medical Sciences, Tehran, Iran; 27 Department of Health Economics, Iran University of Medical Sciences, Tehran, Iran; 28 College of Dental Medicine, Qatar University, Doha, Qatar; 29 Dortmund Clinic, Dortmund, Germany; 30 Department of Epidemiology and Biostatistics, Zahedan University of Medical Sciences, Zahedan, Iran; 31 Department of Surgery, Faculty of Medicine, Gadjah Mada University, Yogyakarta, Indonesia; 32 School of Dentistry and Medical Sciences, Charles Sturt University, Orange, New South Wales, Australia; 33 Department of Applied Mathematics, College of Arts & Sciences, University of Washington, Seattle; 34 Department of Health Metrics Sciences, School of Medicine, University of Washington, Seattle; 35 Department of Molecular and Cell Biology, University of Cape Town, Cape Town, South Africa; 36 Department of Plastic Surgery, University of Texas, Houston; 37 University Institute of Radiological Sciences and Medical Imaging Technology, The University of Lahore, Lahore, Pakistan; 38 Department of Immunology, Zanjan University of Medical Sciences, Zanjan, Iran; 39 Department of Oral and Maxillofacial Surgery, Justus Liebig University of Giessen, Giessen, Germany; 40 Non-Communicable Diseases Research Center, Tehran University of Medical Sciences, Tehran, Iran; 41 Social Determinants of Health Research Center, Shahid Beheshti University of Medical Sciences, Tehran, Iran; 42 Gomal Center of Biochemistry and Biotechnology, Gomal University, Dera Ismail Khan, Pakistan; 43 Department of Nursing, Saveh University of Medical Sciences, Saveh, Iran; 44 Department of Hypertension, Medical University of Lodz, Lodz, Poland; 45 Polish Mothers’ Memorial Hospital Research Institute, Lodz, Poland; 46 Department of Molecular Microbiology and Bacteriology, National Institute of Cholera and Enteric Diseases, Kolkata, India; 47 Department of Molecular Microbiology, Indian Council of Medical Research, New Delhi, India; 48 School of Oral and Dental Sciences, University of Bristol, Bristol, England, United Kingdom; 49 Health Information Management, Shiraz University of Medical Sciences, Shiraz, Iran; 50 Department of Epidemiology and Health Promotion, College of Dentistry, New York University, New York, New York; 51 Faculty of Dentistry, Oral & Craniofacial Sciences, King’s College London, London, England, United Kingdom; 52 Department of Forensic Chemistry, Government Institute of Forensic Science, Aurangabad, India; 53 Department of Anatomy, Royal College of Surgeons in Ireland Medical, University of Bahrain, Busaiteen, Bahrain; 54 Department of Global Public Health and Primary Care, University of Bergen, Bergen, Norway; 55 Cancer Registry of Norway, Oslo, Norway; 56 Department of Medicine, University Ferhat Abbas of Setif, Setif, Algeria; 57 Department of Epidemiology and Preventive Medicine, University Hospital Saadna Abdenour, Setif, Algeria; 58 Department of Epidemiology, College of Public Health and Health Professions and College of Medicine, University of Florida, Gainesville; 59 Cancer Control and Population Sciences Program, University of Florida Health Cancer Center, Gainesville; 60 Department of Epidemiology and Evidence-Based Medicine, I.M. Sechenov First Moscow State Medical University, Moscow, Russia; 61 Department of Clinical Pharmacy, Faculty of Pharmacy, University of Medicine and Pharmacy of Craiova, Craiova, Romania; 62 Institute for Cancer Research, Prevention and Clinical Network, Florence, Italy; 63 School of Population and Public Health, Faculty of Medicine, The University of British Columbia, Vancouver, British Columbia, Canada; 64 Department of Community Medicine, Datta Meghe Institute of Medical Sciences, Sawangi, India; 65 Saveetha Medical College and Hospitals, Saveetha Institute of Medical and Technical Sciences (SIMATS), Chennai, India; 66 Department of Oral Medicine and Radiology, King George’s Medical University, Lucknow, India; 67 Department of Oral Biological and Medical Sciences, The University of British Columbia, Vancouver, British Columbia, Canada; 68 Department of Clinical Oncology, Queen Elizabeth Hospital, Hong Kong, China; 69 Center for Biomedicine and Community Health, International School, Vietnam National University, Hanoi, Vietnam; 70 Department of Paediatric Surgery, Federal Medical Centre, Umuahia, Nigeria; 71 Department of Therapeutic and Diagnostic Technologies, Polytechnic and University Higher Education Cooperative, Gandra, Portugal; 72 Institute for Research and Innovation in Health, University of Porto, Porto, Portugal; 73 Section Global Health and Rehabilitation, Western Norway University of Applied Sciences, Bergen, Norway; 74 Public Health Foundation of India, Gurugram, India; 75 Indian Council of Medical Research, New Delhi, India; 76 Department of Pathology, Johns Hopkins Medicine, Baltimore, Maryland; 77 Department of Pathology, Isfahan University of Medical Sciences, Isfahan, Iran; 78 Environmental Health, Arak University of Medical Sciences, Arak, Iran; 79 Department of Information Technology, University of Human Development, Sulaymaniyah, Iraq; 80 School of Public Health, Salale University, Fiche, Ethiopia; 81 Department of Biochemistry, University of Gondar, Gondar, Ethiopia; 82 Department of Public Health, College of Medicine, Madda Walabu University, Bale Goba, Ethiopia; 83 Department of Epidemiology, Faculty of Health, Medicine and Life Sciences, Maastricht University, Maastricht, the Netherlands; 84 Department of Epidemiology, Shiraz University of Medical Sciences, Shiraz, Iran; 85 Department of Comprehensive Nursing, Arba Minch University, Arba Minch, Ethiopia; 86 Department of Anatomy, All India Institute of Medical Sciences, Jodhpur, India; 87 Department of Physiology, All India Institute of Medical Sciences, Jodhpur, India; 88 Development of Research and Technology Center, Ministry of Health and Medical Education, Tehran, Iran; 89 Department of Biomedical Informatics and Medical Statistics, Medical Research Institute, Alexandria University, Alexandria, Egypt; 90 Department of Pediatric Dentistry and Dental Public Health, Faculty of Dentistry, Alexandria University, Alexandria, Egypt; 91 Department of Health Informatics, Haramaya University, Harar, Ethiopia; 92 Centre for Applied Health Economics, Griffith University, Gold Coast, Queensland, Australia; 93 Department of Clinical Biochemistry, Tarbiat Modares University, Tehran, Iran; 94 Department of Epidemiology and Medical Statistics, College of Medicine, University of Ibadan, Ibadan, Nigeria; 95 The Institute of Applied Health Sciences, University of Aberdeen, Aberdeen, Scotland, United Kingdom; 96 Epidemiology and Biostatistics Unit, National Cancer Institute IRCCS Fondazione G. Pascale, Naples, Italy; 97 Department of Biomedical and Biotechnological Sciences, University of Catania, Catania, Italy; 98 Department of Nursing, College of Medical and Health Sciences, Wollega University, Nekemte, Ethiopia; 99 Department of Dermatology, Kobe University, Kobe, Japan; 100 Department of Community Dentistry, Faculty of Dentistry, Mahidol University, Ratchathewi, Thailand; 101 Population and Patient Health Group, King’s College London, London, England, United Kingdom; 102 Department of Environmental Health Sciences, Mario Negri Institute for Pharmacological Research, Milan, Italy; 103 Department of Environmental Health, College of Medicine and Health Sciences, Wollo University, Dessie, Ethiopia; 104 School of Public Health, Qazvin University of Medical Sciences, Qazvin, Iran; 105 Warwick Medical School, University of Warwick, Coventry, England, United Kingdom; 106 Department of Health Systems and Policy Research, Indian Institute of Public Health, Gandhinagar, India; 107 Department of Genetics, Sana Institute of Higher Education, Sari, Iran; 108 Department of Oral Surgery and Pathology, School of Dentistry, Federal University of Minas Gerais, Belo Horizonte, Brazil; 109 Oncological Network, Institute for Cancer Research, Prevention and Clinical Network, Florence, Italy; 110 School of Dentistry, State University of Montes Claros, Montes Claros, Brazil; 111 Department of Public Health, Torrens University Australia, Melbourne, Victoria, Australia; 112 Toxicology Department, Shriram Institute for Industrial Research, Delhi, India; 113 School of Medicine, Deakin University, Geelong, Victoria, Australia; 114 Faculty of Medicine, Health and Human Sciences, Macquarie University, Sydney, New South Wales, Australia; 115 Department of Pharmacology, Shahid Beheshti University of Medical Sciences, Tehran, Iran; 116 Obesity Research Center, Shahid Beheshti University of Medical Sciences, Tehran, Iran; 117 College of Dentistry, Jazan University, Jazan, Saudi Arabia; 118 School of Dentistry, Sana’a University, Sana’a, Yemen; 119 College of Medicine, University of Sharjah, Sharjah, United Arab Emirates; 120 University Institute of Public Health, The University of Lahore, Lahore, Pakistan; 121 Department of Dental Public Health, Airlangga University, Surabaya, Indonesia; 122 Australian Research Centre for Population Oral Health, University of Adelaide, Adelaide, South Australia, Australia; 123 Department of Nursing, School of Nursing, Arak University of Medical Sciences, Arak, Iran; 124 Department of Ophthalmology, Iran University of Medical Sciences, Karaj, Iran; 125 Gastrointestinal and Liver Diseases Research Center, Guilan University of Medical Sciences, Rasht, Iran; 126 Caspian Digestive Disease Research Center, Guilan University of Medical Sciences, Rasht, Iran; 127 National Data Management Center for Health (NDMC), Ethiopian Public Health Institute, Addis Ababa, Ethiopia; 128 Institute of Pharmaceutical Sciences, University of Veterinary and Animal Sciences, Lahore, Pakistan; 129 Department of Pharmacy Administration and Clinical Pharmacy, Xi’an Jiaotong University, Xi’an, China; 130 Department of Oral Health, Faculty of Health, Autonomous University of Manizales, Manizales, Colombia; 131 Kasturba Medical College, Mangalore, Manipal Academy of Higher Education, Manipal, India; 132 Department of Pulmonology, Yokohama City University, Yokohama, Japan; 133 National Human Genome Research Institute (NHGRI), National Institutes of Health, Bethesda, Maryland; 134 Institute of Research and Development, Duy Tan University, Da Nang, Vietnam; 135 Department of Computer Science, University of Human Development, Sulaymaniyah, Iraq; 136 Czech National Centre for Evidence-Based Healthcare and Knowledge Translation, Masaryk University, Brno, Czech Republic; 137 Institute of Biostatistics and Analyses, Faculty of Medicine, Masaryk University, Brno, Czech Republic; 138 Department of Community Medicine, College of Medicine, University of Ibadan, Ibadan, Nigeria; 139 Department of Community Medicine, University College Hospital, Ibadan, Ibadan, Nigeria; 140 Faculty of Medicine, University of Belgrade, Belgrade, Serbia; 141 Department of Epidemiology, Faculty of Medical Sciences, University of Kragujevac, Kragujevac, Serbia; 142 Department of General Surgery and Surgical-Medical Specialties, University of Catania, Catania, Italy; 143 Centre for Community Medicine, All India Institute of Medical Sciences, New Delhi, India; 144 Department of Internal Medicine, Mount Auburn Hospital, Harvard University, Cambridge, Massachusetts; 145 Health Informatics Lab, Boston University, Boston, Massachusetts; 146 Postgraduate Institute of Medicine, University of Colombo, Colombo, Sri Lanka; 147 Department of Surgery, National Hospital of Sri Lanka, Colombo, Sri Lanka; 148 Department of Biochemistry, Government Medical College, Mysuru, India; 149 Department of Community Medicine, Kasturba Medical College, Mangalore, Manipal Academy of Higher Education, Mangalore, India; 150 Department of Oral and Maxillofacial Pathology, Krishna Vishwa Vidyapeeth (Deemed to be University), Karad, India; 151 Department of Periodontics, School of Dentistry, Louisiana State University Health Sciences Center, New Orleans; 152 University of Florida Health Cancer Center, Gainesville; 153 School of Health Professions and Human Services, Hofstra University, Hempstead, New York; 154 Surgery Research Unit, University of Oulu, Oulu, Finland; 155 Department of Molecular Medicine and Surgery, Karolinska Institute, Stockholm, Sweden; 156 Students’ Scientific Research Center (SSRC), Tehran University of Medical Sciences, Tehran, Iran; 157 Department of Public Health and Community Medicine, Jordan University of Science and Technology, Irbid, Jordan; 158 Amity Institute of Forensic Sciences, Amity University, Noida, India; 159 Global Consortium for Public Health Research, Jawaharlal Nehru Medical College, Datta Meghe Institute of Higher Education and Research, Wardha, India; 160 Department of Neurosurgery, Shahid Beheshti University of Medical Sciences, Tehran, Iran; 161 Department of Medicine, Mazandaran University of Medical Sciences, Sari, Iran; 162 MAZUMS Office, Universal Scientific Education and Research Network, Tehran, Iran; 163 Department of Genomics and Digital Health, Samsung Advanced Institute for Health Sciences & Technology (SAIHST), Seoul, South Korea; 164 Public Health Center, Ministry of Health and Welfare, Wando, South Korea; 165 Children’s Medical Center, Tehran University of Medical Sciences, Tehran, Iran; 166 Social Determinants of Health Research Center, Saveh University of Medical Sciences, Saveh, Iran; 167 Faculty of Health and Life Sciences, Coventry University, Coventry, England, United Kingdom; 168 Department of Medicine, Faculty of Health Sciences, McMaster University, Hamilton, Ontario, Canada; 169 Department of Clinical Sciences and Community Health, University of Milan, Milan, Italy; 170 Unit of Genetics and Public Health, Institute of Medical Sciences, Las Tablas, Panama; 171 Ministry of Health, Herrera, Panama; 172 Department of Otorhinolaryngology, Father Muller Medical College, Mangalore, India; 173 Department of Clinical and Experimental Medicine, University of Catania, Catania, Italy; 174 Department of Preventive Medicine, College of Medicine, Korea University, Seoul, South Korea; 175 Department of Dentistry, Radboud University, Nijmegen, the Netherlands; 176 Department of Translational Health Economics, Heidelberg University Hospital, Heidelberg, Germany; 177 Department of Clinical Oncology, Shahid Beheshti University of Medical Sciences, Tehran, Iran; 178 NHMRC Clinical Trials Centre, University of Sydney, Sydney, New South Wales, Australia; 179 Rabigh Faculty of Medicine, King Abdulaziz University, Jeddah, Saudi Arabia; 180 Department of Health Policy Research, Public Health Foundation of India, Gurugram, India; 181 Institute of Population Health Sciences, University of Liverpool, Liverpool, England, United Kingdom; 182 Department of Biology, College of Science, Salahaddin University, Erbil, Iraq; 183 Department of Preventive Oncology, All India Institute of Medical Sciences, New Delhi, India; 184 Tehran Heart Center, Tehran University of Medical Sciences, Tehran, Iran; 185 University Centre Varazdin, University North, Varazdin, Croatia; 186 Department of Genetics, Shahid Beheshti University of Medical Sciences, Tehran, Iran; 187 National Data Management Center for Health, Ethiopian Public Health Institute, Addis Ababa, Ethiopia; 188 Department of Surgical Oncology, All India Institute of Medical Sciences, Jodhpur, India; 189 Internal Medicine Department, King Saud University, Riyadh, Saudi Arabia; 190 Department of Information Technology, Lebanese French University, Erbil, Iraq; 191 Tehran University of Medical Sciences, Tehran, Iran; 192 Faculty of Medicine, Tehran University of Medical Sciences, Tehran, Iran; 193 School of Health and Rehabilitation Sciences, The University of Queensland, Brisbane, Queensland, Australia; 194 Computer, Electrical and Mathematical Sciences and Engineering Division, King Abdullah University of Science and Technology, Thuwal, Saudi Arabia; 195 Division of Facial Plastic and Reconstructive Surgery, Department of Otolaryngology–Head and Neck Surgery, University of Washington, Seattle; 196 Department of Oral and Maxillofacial Medicine, Kermanshah University of Medical Sciences, Kermanshah, Iran; 197 Department of Epidemiology and Biostatistics, School of Medicine, Wuhan University, Wuhan, China; 198 Health Workforce Department, World Health Organization, Geneva, Switzerland; 199 Mysore Medical College and Research Institute, Government Medical College, Mysore, India; 200 Manipal College of Dental Sciences, Manipal, Manipal Academy of Higher Education, Manipal, India; 201 Department of Dental Public Health, Faculty of Dentistry, King Abdulaziz University, Jeddah, Saudi Arabia; 202 Department of Oral Health Policy and Epidemiology, School of Dental Medicine, Harvard University, Boston, Massachusetts; 203 Department of Oncology, Victor Babes University of Medicine and Pharmacy, Timisoara, Romania; 204 Department of Histopathology, University of Maiduguri Teaching Hospital, Maiduguri, Nigeria; 205 Department of Human Pathology, University of Maiduguri, Maiduguri, Nigeria; 206 Department of Medical Mycology and Parasitology, Shiraz University of Medical Sciences, Shiraz, Iran; 207 Unit of Microbiology and Public Health, Institute of Medical Sciences, Las Tablas, Panama; 208 Department of Public Health, Ministry of Health, Herrera, Panama; 209 Department of Applied Economics and Quantitative Analysis, University of Bucharest, Bucharest, Romania; 210 Department of Psychiatry and Behavioural Neurosciences, Faculty of Health Sciences, McMaster University, Hamilton, Ontario, Canada; 211 Department of Psychiatry, Faculty of Clinical Science, University of Lagos, Lagos, Nigeria; 212 Diplomacy and Public Relations Department, University of Human Development, Sulaymaniyah, Iraq; 213 National School of Public Health, Institute of Health Carlos III, Madrid, Spain; 214 Department of Forensic Medicine and Toxicology, Kasturba Medical College, Mangalore, Manipal Academy of Higher Education, Mangalore, India; 215 Vision and Eye Research Institute, Anglia Ruskin University, Cambridge, England, United Kingdom; 216 Global Health Governance Programme, University of Edinburgh, Edinburgh, Scotland, United Kingdom; 217 School of Dentistry, University of Leeds, Leeds, England, United Kingdom; 218 Endocrinology Unit, Department of Medicine, University of Padova, Padova, Italy; 219 Associazione Italiana Ricerca Oncologica di Base (AIROB), Padova, Italy; 220 International Center of Medical Sciences Research, Islamabad, Pakistan; 221 School of Engineering, Macquarie University, Sydney, New South Wales, Australia; 222 Pohang University of Science and Technology, Pohang, South Korea; 223 Department of Medical Oncology, Cancer Institute (WIA), Chennai, India; 224 Future Technology Research Center, National Yunlin University of Science and Technology, Yunlin, Taiwan; 225 Department of Public Health, Torbat Jam Faculty of Medical Sciences, Torbat Jam, Iran; 226 Department of Community Medicine, Kasturba Medical College, Manipal, Manipal Academy of Higher Education, Manipal, India; 227 Department of Oral Pathology and Microbiology, Sharavathi Dental College and Hospital, Shimogga, India; 228 Department of Radiation Oncology, All India Institute of Medical Sciences, New Delhi, India; 229 WHO Collaborating Centre for Public Health Education and Training, Imperial College London, London, England, United Kingdom; 230 Inovus Medical, St Helens, England, United Kingdom; 231 Department of Primary Care and Public Health, Faculty of Medicine, Imperial College London, London, England, United Kingdom; 232 Academic Public Health England, Public Health England, London, England, United Kingdom; 233 Department of Computer Science, College of Arts & Sciences, Boston University, Boston, Massachusetts; 234 Department of Immunology and Laboratory Sciences, Sirjan School of Medical Sciences, Sirjan, Iran; 235 Department of Immunology, Kerman University of Medical Sciences, Kerman, Iran; 236 Endocrinology and Metabolism Research Institute, Tehran University of Medical Sciences, Tehran, Iran; 237 Research Center for Immunodeficiencies, Tehran University of Medical Sciences, Tehran, Iran; 238 Network of Immunity in Infection, Malignancy and Autoimmunity (NIIMA), Universal Scientific Education and Research Network (USERN), Tehran, Iran; 239 Department of Public Health, Masaryk University, Brno, Czech Republic; 240 Czech National Centre for Evidence-based Healthcare and Knowledge Translation, Masaryk University, Brno, Czech Republic; 241 Department of Medicine, Massachusetts General Hospital, Boston; 242 Harvard Medical School, Harvard University, Boston, Massachusetts; 243 Clinical and Epidemiological Research in Primary Care (GICEAP), Maimonides Biomedical Research Institute of Cordoba (IMIBIC), Cordoba, Spain; 244 Golestan Research Center of Gastroenterology and Hepatology, Golestan University of Medical Sciences, Gorgan, Iran; 245 Department of Oral and Maxillofacial Surgery, JSS Academy of Higher Education and Research, Mysore, India; 246 Sharjah Institute for Medical Research, University of Sharjah, Sharjah, United Arab Emirates; 247 Department of Polymer Technology, Faculty of Chemistry, Gdańsk University of Technology, Gdańsk, Poland; 248 Multidisciplinary Laboratory Foundation University School of Health Sciences (FUSH), Foundation University, Islamabad, Pakistan; 249 Advanced Dental Sciences Research Center, Kermanshah University of Medical Sciences, Kermanshah, Iran; 250 Department of Medical Biochemistry, Rafsanjan University of Medical Sciences, Rafsanjan, Iran; 251 Medical Laboratory Sciences, Sirjan School of Medical Sciences, Sirjan, Iran; 252 Applied Biomedical Research Center, Mashhad University of Medical Sciences, Mashhad, Iran; 253 Biotechnology Research Center, Mashhad University of Medical Sciences, Mashhad, Iran; 254 Department of Immunology, Pasteur Institute of Iran, Tehran, Iran; 255 Department of Entomology, Faculty of Science, Ain Shams University, Cairo, Egypt; 256 Medical Ain Shams Research Institute (MARSI), Ain Shams University, Cairo, Egypt; 257 School of Public Health and Health Management, University of Belgrade, Belgrade, Serbia; 258 Faculty of Health and Social Sciences, Bournemouth University, Bournemouth, England, United Kingdom; 259 UGC Centre of Advanced Study in Psychology, Utkal University, Bhubaneswar, India; 260 Udyam-Global Association for Sustainable Development, Bhubaneswar, India; 261 Department of Medical Statistics, University of Zagreb, Zagreb, Croatia; 262 Department of Epidemiology and Prevention of Chronic Noncommunicable Diseases, Croatian Institute of Public Health, Zagreb, Croatia; 263 Emergency Department, Manian Medical Centre, Erode, India; 264 National Heart, Lung, and Blood Institute, National Institutes of Health, Rockville, Maryland; 265 Department of Radiology and Radiological Science, Johns Hopkins Medicine, Baltimore, Maryland; 266 Department of Radiology and Interventional Neuroradiology, Isfahan University of Medical Sciences, Isfahan, Iran; 267 Department of Chemistry, Institute for Advanced Studies in Basic Sciences (IASBS), Zanjan, Iran; 268 Department of Oral Health, Non-Communicable Diseases Research Center (NCDRC), Tehran, Iran; 269 Non-Communicable Diseases Committee, National Institute for Medical Research Development (NIMAD), Tehran, Iran; 270 Institute of Public Health, University of Gondar, Gondar, Ethiopia; 271 Faculty of Medicine, University of Azuay, Cuenca, Ecuador; 272 Department of Biochemistry, Royal College of Surgeons in Ireland Medical University of Bahrain, Busaiteen, Bahrain; 273 Department of Public Health Dentistry, Krishna Vishwa Vidyapeeth (Deemed to be University), Karad, India; 274 Department of International Studies, Non-Communicable Diseases Research Center (NCDRC), Tehran, Iran; 275 Department of Medical-Surgical Nursing, Nasibeh School of Nursing and Midwifery, Mazandaran University of Medical Sciences, Sari, Iran; 276 College of Nursing and Health Sciences, Flinders University, Adelaide, South Australia, Australia; 277 School of Pharmacy, Monash University, Selangor Darul Ehsan, Malaysia; 278 Department of Hematology-Oncology, Baystate Medical Center, Springfield, Massachusetts; 279 Department of Radiodiagnosis, All India Institute of Medical Sciences, Bathinda, India; 280 Heersink School of Medicine, University of Alabama at Birmingham, Birmingham; 281 Department of Medicine Service, US Department of Veterans Affairs, Birmingham, Alabama; 282 Department of Community Medicine, Lady Hardinge Medical College, New Delhi, India; 283 Department of Community Medicine, All India Institute of Medical Sciences, Jodhpur, India; 284 Department of Epidemiology, School of Preventive Oncology, Patna, India; 285 Department of Epidemiology, Healis Sekhsaria Institute for Public Health, Mumbai, India; 286 Department of Nursing, Dire Dawa University, Dire Dawa, Ethiopia; 287 Center for Biotechnology and Microbiology, University of Swat, Mingora, Pakistan; 288 School of Life Sciences, Xiamen University, Xiamen, China; 289 National Institute of Epidemiology, Indian Council of Medical Research, Chennai, India; 290 Living Systems Institute, University of Exeter, Exeter, England, United Kingdom; 291 Pathology Department, Alexandria University, Alexandria, Egypt; 292 Department of Surgery, National University of Singapore, Singapore, Singapore; 293 Department of Cell Therapy and Applied Genomics, King Hussein Cancer Center, Amman, Jordan; 294 Department of Clinical Epidemiology, Leibniz Institute for Prevention Research and Epidemiology, Bremen, Germany; 295 Department of Epidemiology and Biostatistics, Birjand University of Medical Sciences, Birjand, Iran; 296 Department of Epidemiology and Biostatistics, Iran University of Medical Sciences, Tehran, Iran; 297 Saveetha Dental College and Hospitals, Saveetha Institute of Medical and Technical Sciences (SIMATS), Chennai, India; 298 Modestum LTD, Eastbourne, England, United Kingdom; 299 Kasturba Medical College, Mangalore, Manipal Academy of Higher Education, Mangalore, India; 300 Faculty of Information Technology, Ho Chi Minh City University of Technology (HUTECH), Ho Chi Minh City, Vietnam; 301 Department of Medical Oncology, University of Medicine and Pharmacy “Grigore T Popa” Iaşi, Iaşi, Romania; 302 Department of Medical Oncology, Regional Institute of Oncology, Iaşi, Romania; 303 Department of Medicine, Vanderbilt University, Nashville, Tennessee; 304 Competence Center of Mortality-Follow-Up of the German National Cohort, Federal Institute for Population Research, Wiesbaden, Germany; 305 Department of Community Medicine, Faculty of Medicine and Allied Sciences, Rajarata University of Sri Lanka, Anuradhapura, Sri Lanka; 306 School of Public Health, Zhejiang University, Zhejiang, China; 307 Public Health Sciences Division, Fred Hutchinson Cancer Research Center, Seattle, Washington; 308 Hacettepe University Cancer Institute, Ankara, Turkey; 309 Department of Clinical Pharmacy and Outcomes Sciences, College of Pharmacy, University of South Carolina, Columbia; 310 Epidemiology and Cancer Registry Sector, Institute of Oncology Ljubljana, Ljubljana, Slovenia; 311 Research and Development Department, Sina Medical Biochemistry Technologies, Shiraz, Iran; 312 School of Medicine, Faculty of Medical Sciences, Wuhan University, Wuhan, China; 313 Department of Nursing, Yasuj University of Medical Sciences, Yasuj, Iran; 314 Division of Hematology-Oncology, Department of Pediatrics, University of Washington, Seattle; 315 Department of Preventive and Social Dentistry, Federal University of Rio Grande do Sul, Porto Alegre, Brazil

## Abstract

**Importance:**

Lip, oral, and pharyngeal cancers are important contributors to cancer burden worldwide, and a comprehensive evaluation of their burden globally, regionally, and nationally is crucial for effective policy planning.

**Objective:**

To analyze the total and risk-attributable burden of lip and oral cavity cancer (LOC) and other pharyngeal cancer (OPC) for 204 countries and territories and by Socio-demographic Index (SDI) using 2019 Global Burden of Diseases, Injuries, and Risk Factors (GBD) Study estimates.

**Evidence Review:**

The incidence, mortality, and disability-adjusted life years (DALYs) due to LOC and OPC from 1990 to 2019 were estimated using GBD 2019 methods. The GBD 2019 comparative risk assessment framework was used to estimate the proportion of deaths and DALYs for LOC and OPC attributable to smoking, tobacco, and alcohol consumption in 2019.

**Findings:**

In 2019, 370 000 (95% uncertainty interval [UI], 338 000-401 000) cases and 199 000 (95% UI, 181 000-217 000) deaths for LOC and 167 000 (95% UI, 153 000-180 000) cases and 114 000 (95% UI, 103 000-126 000) deaths for OPC were estimated to occur globally, contributing 5.5 million (95% UI, 5.0-6.0 million) and 3.2 million (95% UI, 2.9-3.6 million) DALYs, respectively. From 1990 to 2019, low-middle and low SDI regions consistently showed the highest age-standardized mortality rates due to LOC and OPC, while the high SDI strata exhibited age-standardized incidence rates decreasing for LOC and increasing for OPC. Globally in 2019, smoking had the greatest contribution to risk-attributable OPC deaths for both sexes (55.8% [95% UI, 49.2%-62.0%] of all OPC deaths in male individuals and 17.4% [95% UI, 13.8%-21.2%] of all OPC deaths in female individuals). Smoking and alcohol both contributed to substantial LOC deaths globally among male individuals (42.3% [95% UI, 35.2%-48.6%] and 40.2% [95% UI, 33.3%-46.8%] of all risk-attributable cancer deaths, respectively), while chewing tobacco contributed to the greatest attributable LOC deaths among female individuals (27.6% [95% UI, 21.5%-33.8%]), driven by high risk-attributable burden in South and Southeast Asia.

**Conclusions and Relevance:**

In this systematic analysis, disparities in LOC and OPC burden existed across the SDI spectrum, and a considerable percentage of burden was attributable to tobacco and alcohol use. These estimates can contribute to an understanding of the distribution and disparities in LOC and OPC burden globally and support cancer control planning efforts.

## Key Points

**Question:**

What was the burden of lip and oral cavity cancer (LOC) and other pharyngeal cancer (OPC) globally, regionally, and across Socio-demographic Index (SDI) strata between 1990 and 2019?

**Findings:**

In this systematic analysis of estimates from the Global Burden of Diseases, Injuries, and Risk Factors Study 2019, the global age-standardized mortality rate due to LOC and OPC in 2019 was 3.8 and 2.2 deaths per 100 000, respectively, and the age-standardized incidence rate was 7.1 and 3.2 new cases per 100 000, respectively. Low-middle and low SDI regions consistently showed the highest age-standardized mortality rates from 1990 to 2019.

**Meanings:**

Tackling the inequities across SDI strata should be a priority to global LOC and OPC control efforts.

## Teaser

This systematic analysis assesses the total and risk-attributable burden of lip and oral cavity cancer and other pharyngeal cancer for 204 countries and territories and by Socio-demographic Index using 2019 Global Burden of Diseases, Injuries, and Risk Factors Study estimates.

## Introduction

In 2019, it was estimated that 10 million people died due to cancer worldwide.^1^ While a relatively small percentage of global cancer deaths were caused by lip, oral cavity, and pharyngeal cancers (3.2%), there is broad variation in survival around the world,^2^ and those who survive may have substantial reductions in their quality of life.^1,3^ Reasons for differences in outcomes are multifactorial but likely include differences in access to early-stage detection and effective therapies, as well as potential differences in risk factor exposure patterns.^1^ Established risk factors for oral and pharyngeal cancers are tobacco, alcohol, and betel quid consumption,^4,5^ all of which increase cancer risk in a dose- and time-dependent fashion.^6,7^ Infection with human papillomavirus (HPV) is also a known risk factor—established for oral cavity, tonsil, and oropharynx cancers^4,5^—which is especially relevant in certain geographic areas of the world.^8,9^

Monitoring the magnitude of cancer burden, as well as the demographic, spatial, and temporal variations in cancer burden, is necessary for tailoring health planning and setting priorities for future clinical care and research.^10,11^ Policymakers require locally relevant information on the burden of different cancers to assess the effect of cancer control programs, benchmark progress, and allocate resources in their health care systems, but some countries do not have cancer surveillance systems in place. Global cancer estimation frameworks, including the Global Burden of Diseases, Injuries, and Risk Factors (GBD) Study from the Institute for Health Metrics and Evaluation and the GLOBOCAN study from the International Agency for Research on Cancer provide estimates of cancer burden where data are scarce or do not exist. Prior studies have reported on the global incidence and mortality estimates of oral and pharyngeal cancers from previous iterations of the GBD study^12,13^ and GLOBOCAN.^14^ However, to our knowledge, there has not been a publication from the GBD Collaborator Network on these 2 cancer types, and the existing analyses do not provide a comprehensive global overview of their burden over time nor quantify the role of risk factors on their global distribution. Understanding the distribution of these 2 types of cancer worldwide and their associated risk factors is particularly relevant at present, given the adoption of an oral health resolution at the World Health Organization (WHO) 2021 World Health Assembly that includes oral cavity cancers and calls for prevention strategies among other actions.^15^

This systematic analysis aimed to analyze the incidence, mortality, and disability-adjusted life years (DALYs) of lip and oral cavity cancer (LOC) and other pharyngeal cancer (OPC; ie, pharyngeal other than nasopharyngeal) globally, regionally, nationally, and by Socio-demographic Index (SDI) from 1990 to 2019, as well as to assess the burden of these cancers attributable to tobacco and alcohol use in 2019 by using estimates from the GBD 2019 study.

## Methods

### Overview

The estimates that are presented in this report originated from the GBD 2019 study.^1,10^ With each new edition of the GBD, data are updated and new methods are used; thus, estimates for the entire time series supplant previously reported GBD round estimates. In this section, we provide information on the key methodological steps for the estimates reported in this study. More detailed descriptions of the methods are available in the eMethods in Supplement 1 and in the literature produced by the GBD 2019 study.^10,16^

The University of Washington Institutional Review Board committee approved the GBD 2019 study. Informed consent was waived because of the use of deidentified data. The GBD complies with the Guidelines for Accurate and Transparent Health Estimates Report (GATHER) statement.^17^ This article was produced through the GBD Collaborator Network and in accordance with the GBD Protocol.^18^

### Definitions

All estimates are reported for adults, defined in this study as 20 years and older. The estimates are presented by sex, 5-year age groups (20-24, 25-29, 30-34, … ≥95 years), globally, and by region for the years 1990 to 2019. The estimates by region are based on 3 geographic classifications: GBD super-regions (7 total), GBD world regions (21 total), and countries or territories (204 total) (eFigures 3-5 and eTable 7 in Supplement 1).^10^ The countries were also classified by SDI quintiles for presentation of select results (eTable 7 in Supplement 1). All rates are presented for every 100 000 people per year, and age-standardized rates use the GBD world population standard (eMethods in Supplement 1). All estimates are reported with 95% uncertainty intervals (UIs), which are created from the 25th and 975th values of 1000 draws and propagated through each estimation step. Lip and oral cavity cancer includes *International Statistical Classification of Diseases and Related Health Problems, Tenth Revision *(*ICD-10*) codes C00 to C08, and OPC includes *ICD-10* codes C09 to C10 and C12 to C13. More detailed mapping of *International Classification of Diseases, Ninth Revision (ICD-9) *and *ICD-10* codes to the GBD cancer causes LOC and OPC are summarized in eTable 1 in Supplement 1.

The present analysis uses the term *other pharyngeal cancer* (synonymous with *other pharynx cancer* in the GBD 2019 study and throughout Supplement 1) because nasopharyngeal cancer is separately estimated. Nasopharyngeal cancer is not included in this publication because it differs epidemiologically from cancers that occur in other pharyngeal sites, and we use the term OPC to be consistent with publicly available GBD results and visualizations.

### Mortality Estimates

The GBD cancer estimation process begins with mortality (eFigure 1 in Supplement 1). The sources of these data were vital registration systems, cancer registries, and verbal autopsies. The data reported were mapped to a list of underlying causes (cancer types) in the GBD causes of death hierarchy (eTable 1 in Supplement 1).^1,10^ Uninformative cause of death codes (the “garbage codes”^19^) were redistributed among appropriate underlying causes of death.^1,10^ Mortality data were not captured by vital registries in some countries where cancer incidence data were available. To expand data availability informing mortality models, incidence data were transformed into mortality estimates using modeled mortality-to-incidence ratios (MIRs). The MIR modeling process used cancer registry data from locations where incidence and mortality of the same year were available. This model includes a linear-step mixed-effects model with logit link functions, with age, sex, and the Healthcare Access and Quality Index as covariates. The results of this step were smoothed over space and time and adjusted through a spatiotemporal Gaussian process regression.^1,10^ Death data and estimates were included in cancer cause and sex-specific Cause of Death Ensemble models (eMethods and eTables 2-4 in Supplement 1).^20^ Finally, cancer mortality estimates were adjusted to independently modeled all-cause mortality.^10^

### Incidence and DALYs Estimates

Incidence estimates were obtained by dividing the final mortality estimates by their corresponding MIRs (eFigure 2 in Supplement 1). The 10-year prevalence estimate was derived from the estimated incidence and the survival modeled using MIRs.^10^ Years lived with disability were estimated by separating 10-year cancer prevalence into 4 sequelae with associated disability weights (eTables 5 and 6 in Supplement 1). Disability weights range from 0 to 1 and represent the magnitude of health loss (0, no health loss; 1, health loss equivalent to death). Years lived with disability were obtained by multiplying each sequela duration by the corresponding disability weight. Finally, years of life lost were estimated by multiplying the difference between the standard GBD life expectancy at the age of death and the estimated number of deaths at that age. Years lived with disability and years of life lost were summed by cause, sex, age group, location, and year to result in DALYs.^10^

### Socio-demographic Index

The SDI incorporates 3 aspects of development: (1) total fertility rate for female individuals younger than 25 years, (2) mean education for those 15 years and older, and (3) lag-distributed income per capita.^10^ The eMethods in Supplement 1 present further SDI details as well as which countries make up each SDI quintile (eTable 7 in Supplement 1).

### Risk Factors: Population Attributable Fraction Estimation

The GBD 2019 comparative risk assessment framework was used to estimate the proportion of deaths and DALYs for LOC and OPC in 2019 attributable to the risk factors estimated. The risk assessment used in the framework was the attributable burden, which means the discount in the current disease burden that would have been possible if past population risk exposure had changed to an alternative or counterfactual distribution of exposure. Theoretical minimum risk exposure level was the alternative distribution used in the model, which represents the level of risk exposure that minimizes risk at the population level or the level of risk that captures the maximum attributable burden. This study presents the proportion of mortality and DALYs due to LOC attributable to smoking, chewing tobacco, and alcohol consumption, as well as the proportion of mortality and DALYs due to OPC attributable to smoking and alcohol consumption; the estimates considered the population aged 20 years and older. These risk factors were chosen based on the risk-outcome pairs that the GBD 2019 study assessed as meeting the World Cancer Research Fund grades of convincing or probable evidence. The theoretical minimum risk exposure level for smoking and chewing tobacco was that all individuals were lifelong nonusers and for alcohol use was an estimated distribution of 0 to 10 g per day. Detailed methodology for risk factor estimation can be found in the eMethods in Supplement 1 and the GBD 2019 risk factors capstone publication.^16^

## Results

### The Global Burden of LOC in 2019

In 2019, 370 000 (95% UI, 338 000-401 000) new cases of LOC occurred globally, and the global age-standardized incidence rate (ASIR) was 7.1 (95% UI, 6.5-7.7) per 100 000. Deaths from LOC were estimated to be 199 000 (95% UI, 181 000-217 000) globally, with an age-standardized mortality rate (ASMR) of 3.8 (95% UI, 3.5-4.2) deaths per 100 000. Lip and oral cavity cancer was responsible for 5.45 million (95% UI, 4.95-5.97 million ) DALYs in 2019 (eTable 8 in Supplement 1).

**Figure 1.**
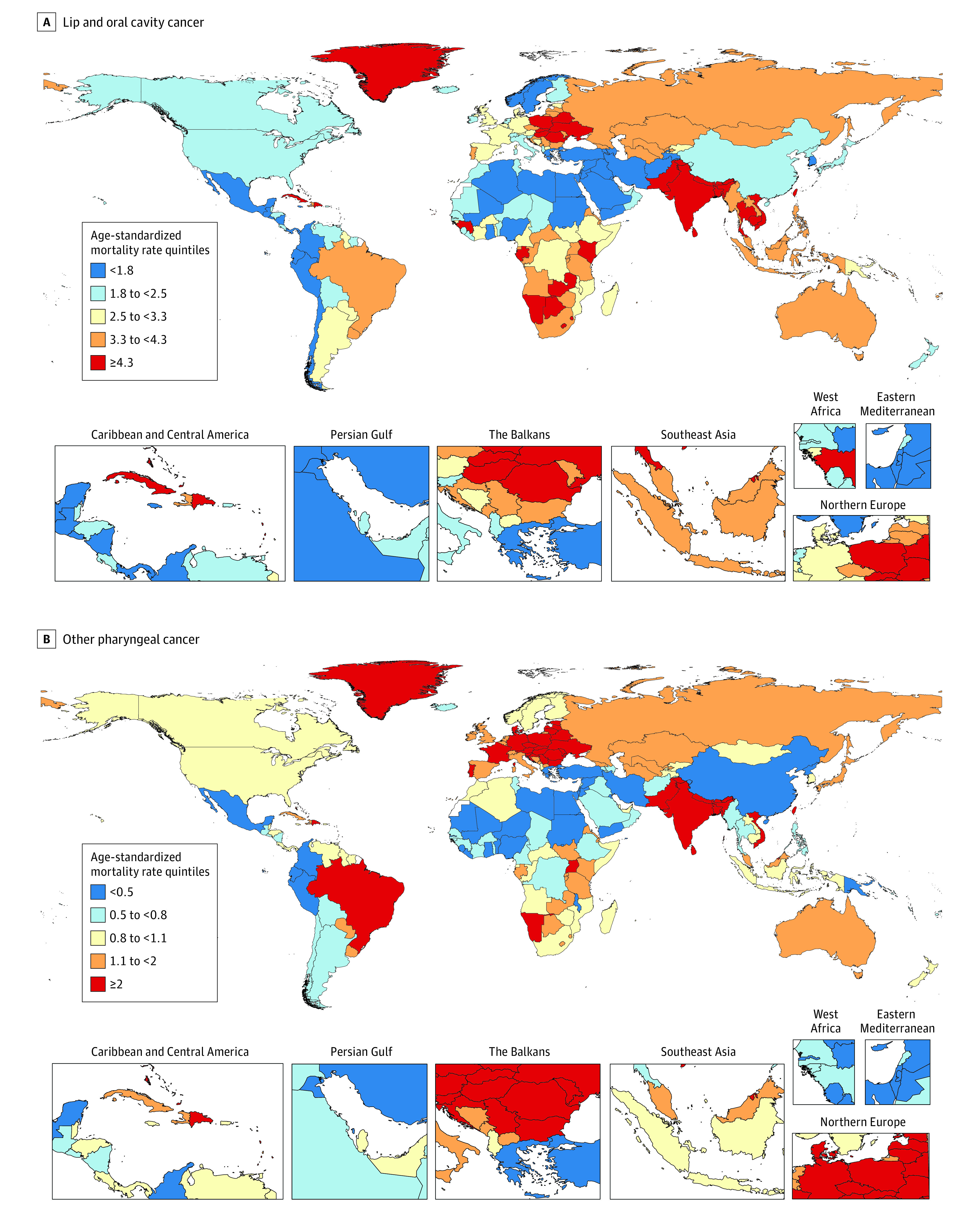
Global Maps of Age-Standardized Mortality Rates for Lip, Oral, and Other Pharyngeal Cancer for Both Sexes Combined in 2019 Each map represents estimates at the national level and for the age range of 20 to older than 95 years. Quintiles are based on age-standardized mortality rates per 100 000 person-years. There are several geographic locations where estimates are not available (eg, Western Sahara, French Guiana) because they were not modeled locations in the Global Burden of Diseases, Injuries, and Risk Factors Study 2019; these locations are white on the maps. eFigure 6 in Supplement 1 provides global maps showing the age-standardized incidence rate quintiles for lip and oral cavity cancer and other pharyngeal cancer among both sexes in 2019. eFigures 7 through 10 in Supplement 1 provide additional global maps of age-standardized mortality and incidence rates for lip and oral cavity cancer and other pharyngeal cancer in male and female individuals separately in 2019.

**Figure 2.**
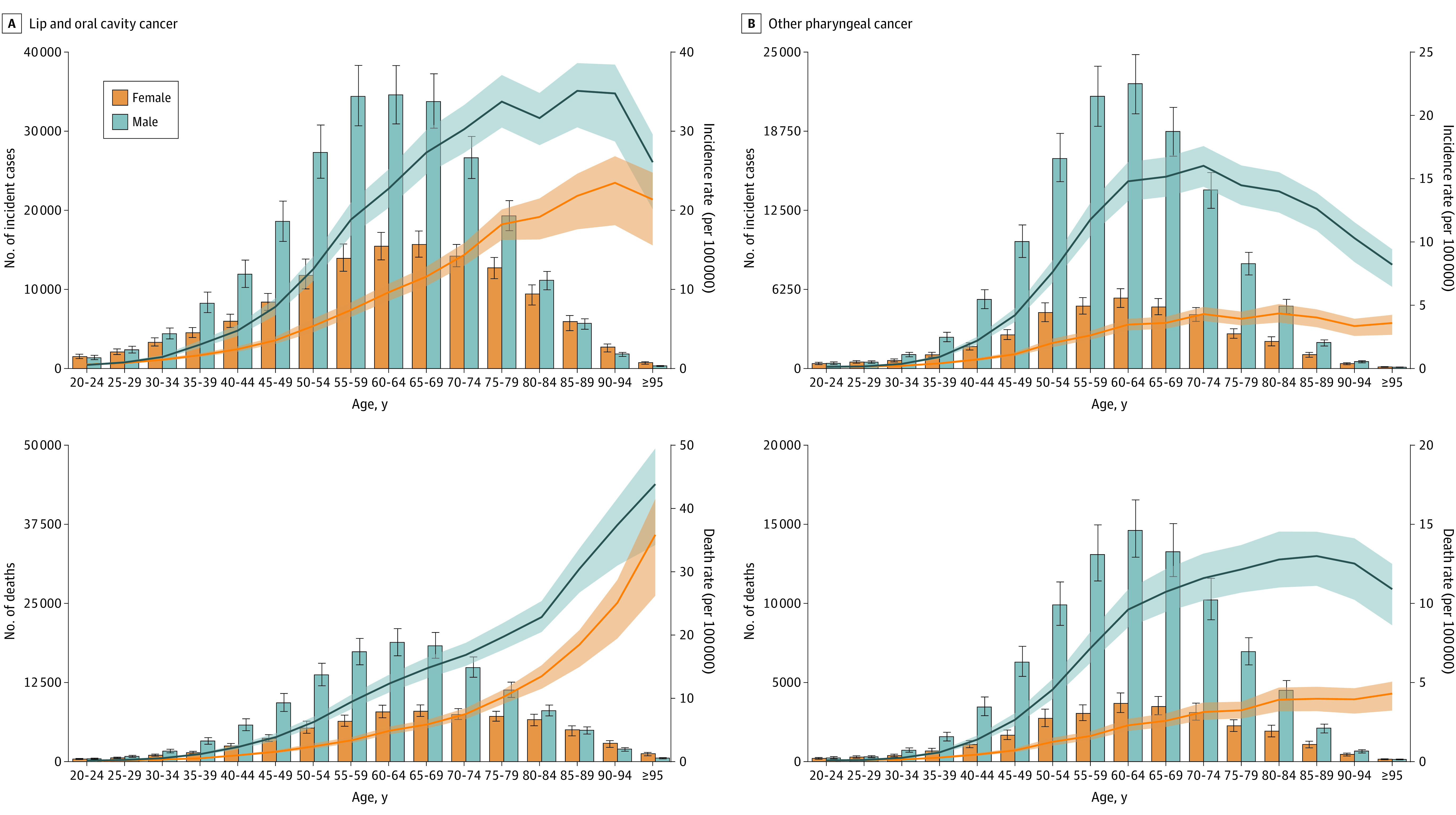
Global Absolute Cases and Deaths and Age-Specific Incidence and Mortality Rates for Lip, Oral, and Other Pharyngeal Cancer by Age Group and Sex in 2019 Bars indicate absolute numbers, with error bars representing 95% uncertainty intervals. Lines indicate rates, with shaded areas representing respective 95% uncertainty intervals. For an additional version of this Figure showing absolute disability-adjusted life years and age-specific disability-adjusted life year rates (per 100 000), see eFigure 11 in Supplement 1.

Figure 1A shows the distribution of national-level ASMRs in 2019 due to LOC (for a map of ASIRs, see eFigure 6 in Supplement 1). Eastern Europe and South and Southeast Asia exhibited a concentration of countries with ASMRs in the highest quintile. The LOC age-specific rates for incidence, mortality, and DALYs were higher among male individuals than among female individuals in all age groups (Figure 2 and eFigure 11 in Supplement 1).

The South Asia and High-income super-regions had the highest ASIRs for LOC in 2019 (15.1 [95% UI, 12.8-17.5] and 7.2 [95% UI, 6.4-7.9] new cases per 100 000 inhabitants, respectively). South Asia also had the highest ASMR (10.0 [95% UI, 8.6-11.7] per 100 000); however, the second-largest ASMR occurred in Central Europe, Eastern Europe, and Central Asia (4.2 [95% UI, 3.8-4.6] per 100 000). The low-middle SDI regions had the highest ASIR and ASMR (10.4 [95% UI, 9.1-11.8] new cases and 7.0 [95% UI, 6.1-7.9] deaths per 100 000, respectively; eTable 8 in Supplement 1). The country with the highest ASIR in 2019 was Palau, with 46.6 (95% UI, 36.3-59.2) new cases per 100 000, while the highest ASMR occurred in Pakistan, with 23.2 (95% UI, 18.7-28.9) deaths per 100 000 (eTable 10 in Supplement 1).

### The Burden of LOC Over Time

**Figure 3.**
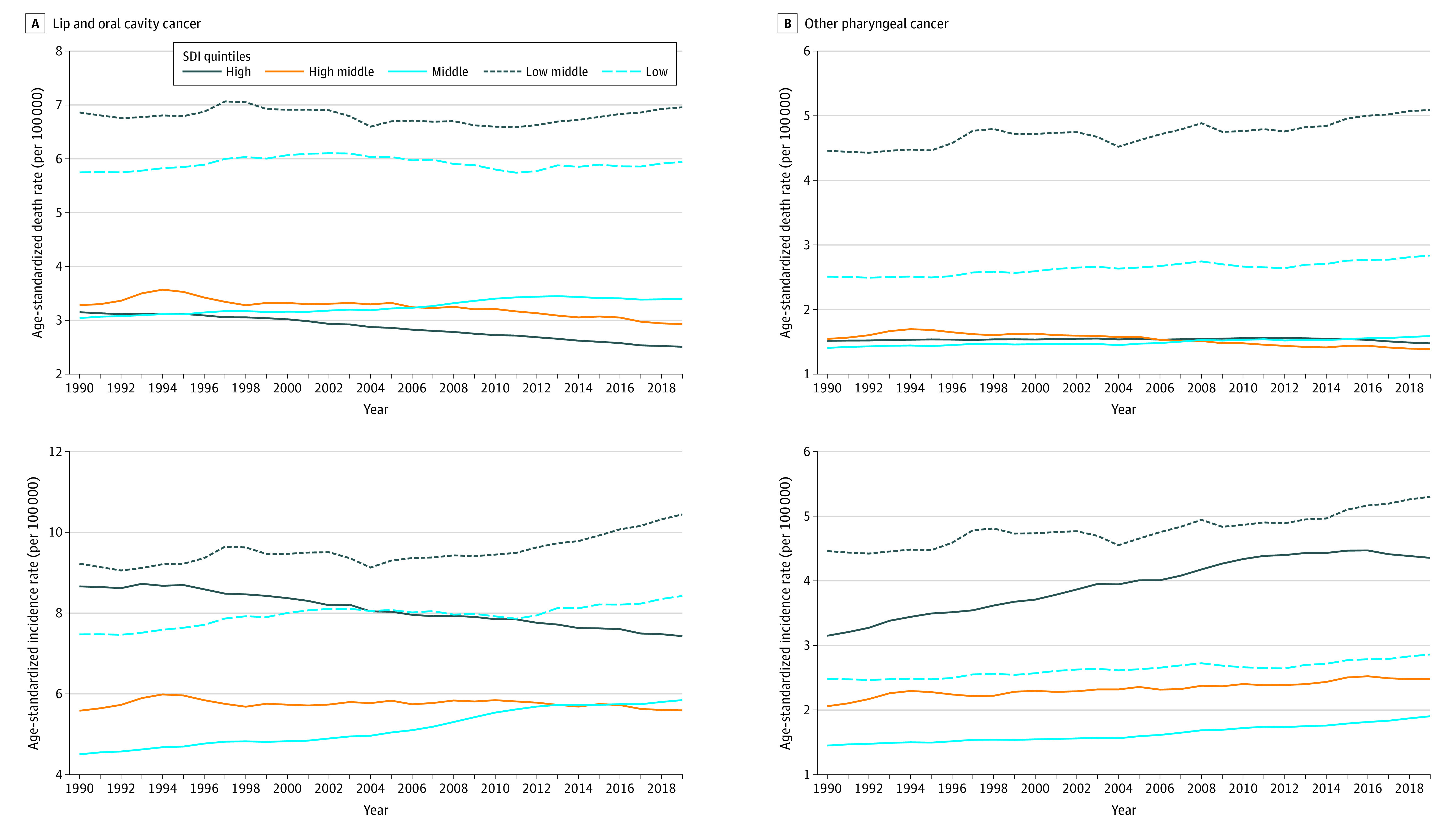
Time Trends of Age-Standardized Death and Incidence Rates for Lip, Oral, and Other Pharyngeal Cancer From 1990 to 2019 by Socio-demographic Index (SDI) Quintile Rates represent both sexes combined and are expressed per 100 000 person-years. See eFigure 3 and eTable 7 in Supplement 1 for details and definitions of the SDI quintiles. For additional versions of this Figure, showing time trends of deaths, incidence, and disability-adjusted life years, see eFigure 12 (by SDI quintile), eFigure 13 (by sex), eFigure 14 (by 10-year age group), and eFigure 15 (by Global Burden of Diseases, Injuries, and Risk Factors Study super-region) in Supplement 1.

From 1990 to 2019, the high SDI regions showed a decreasing pattern in ASIR, ASMR, and age-standardized DALY rates (Figure 3 and eFigure 12 in Supplement 1). The high-middle SDI quintile had decreasing ASMR and age-standardized DALY rates, while the ASIR of the middle SDI regions increased (eTable 8 in Supplement 1). Low-middle and low SDI regions consistently showed the highest ASMRs due to LOC from 1990 to 2019 (Figure 3). The LOC ASIRs, ASMRs, and age-standardized DALY rates over time by sex and super-region, as well as the age-specific rates over time and by age group, are reported in eFigures 13, 15, and 14 in Supplement 1, respectively.

### The Global Burden of OPC in 2019

The estimated number of new OPC cases in 2019 was 167 000 (95% UI, 153 000-180 000), with an ASIR of 3.2 (95% UI, 2.9-3.4) per 100 000 inhabitants. It was estimated that 114 000 (95% UI, 103 000-126 000) people died of OPC, revealing a global ASMR of 2.2 (95% UI, 2.0-2.4) per 100 000. Other pharyngeal cancer caused 3.23 million (95% UI, 2.90-3.57 million) DALYs in 2019 (eTable 9 in Supplement 1).

Figure 1B presents the distribution of national-level ASMRs in 2019 due to OPC (for a map of ASIRs, see eFigure 6 in Supplement 1). Most European countries were in the highest quintile of ASMRs; there was also a concentration of South Asian countries in this quintile. The OPC age-specific rates of incidence, mortality, and DALYs among male individuals were higher than female individuals in all age groups; the difference between male and female individuals in OPC age-specific rates was greater in middle age than in the older and younger age groups (Figure 2 and eFigure 11 in Supplement 1).

In 2019, South Asia had the highest ASIR and ASMR for OPC (7.7 [95% UI, 6.5-8.9] new cases and 7.4 [95% UI, 6.3-8.5] deaths per 100 000 inhabitants, respectively). Low-middle and high SDI regions had the highest ASIRs (5.3 [95% UI, 4.6-6.1] and 4.4 [95% UI, 3.9-4.9] new cases per 100 000, respectively). The low-middle SDI quintile also had the highest ASMR (5.1 [95% UI, 4.4-5.9] per 100 000), but the low SDI quintile had the second highest ASMR (2.8 [95% UI, 2.4-3.3] per 100 000) (eTable 9 in Supplement 1). Taiwan (province of China) was the country with the highest ASIR in 2019 (9.8 [95% UI, 7.4-13.0] incident cases per 100 000), while India had the highest ASMR (7.7 [95% UI, 6.4-9.2] deaths per 100 000) (eTable 11 in Supplement 1).

### The Burden of OPC Over Time

The OPC ASIRs increased from 1990 to 2019 in the high, high-middle, and middle SDI quintiles. There was a general pattern of stability across all SDI settings regarding OPC ASMRs, except for the high-middle strata, which showed a reduction (eTable 9 in Supplement 1). For age-standardized DALY rates, high and high-middle SDI settings exhibited a decreasing pattern (eTable 9 in Supplement 1). From 1990 to 2019, low-middle and low SDI regions always showed the highest ASMRs due to OPC (Figure 3). The OPC ASIRs, ASMRs, and age-standardized DALY rates over time by sex and super-region, as well as the age-specific rates over time by age group, are reported in eFigures 13, 15, and 14 in Supplement 1, respectively.

### Risk Factors: Population Attributable Fraction

**Figure 4.**
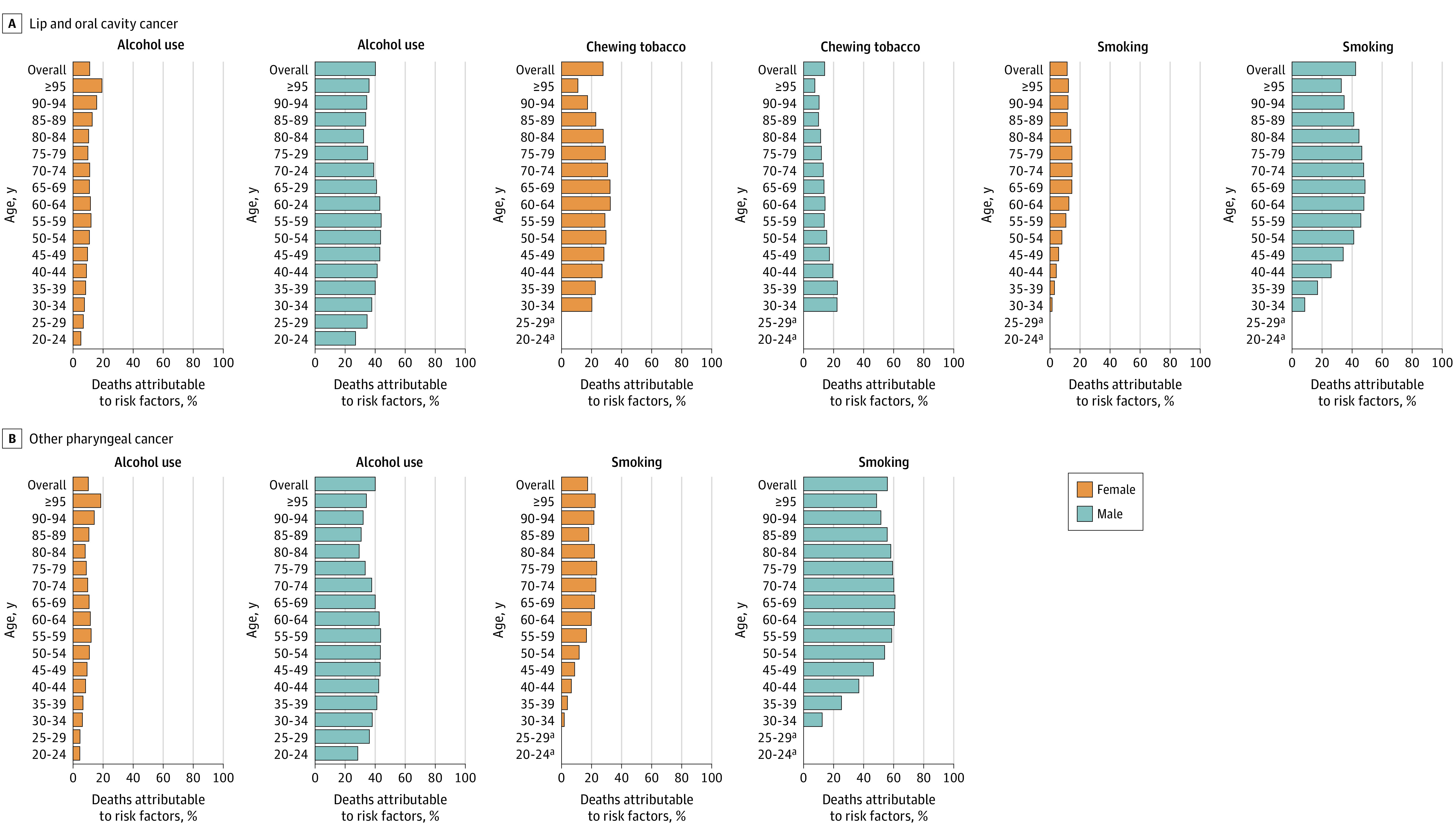
Proportion of Deaths Attributable to Risk Factors for Lip, Oral, and Other Pharyngeal Cancer by Age Group and Sex Globally in 2019 eFigure 16 in Supplement 1 provides further information on deaths attributable to risk factors for lip, oral, and other pharyngeal cancers in 2019 by Global Burden of Diseases, Injuries, and Risk Factors (GBD) Study world region. eFigure 17 in Supplement 1 provides further information on risk-attributable disability-adjusted life-years for lip, oral, and other pharyngeal cancers by 5-year age groups globally and by GBD study world region. For Tables summarizing the underlying results of risk-attributable deaths and disability-adjusted life-years by country or territory, see eTable 12 (for lip and oral cavity cancer) and eTable 13 (for other pharyngeal cancer) in Supplement 1. An additional version of this Figure showing percentage totals for each category is provided in eFigure 18 in Supplement 1. ^a^The chewing tobacco and smoking risk factors were modeled with lower age restrictions of 30 years in the GBD 2019 study; thus, estimates were not produced for these risk factors in the age groups of 20 to 24 years and 25 to 29 years.

Figure 4 shows the proportion of LOC and OPC deaths attributable to alcohol and tobacco consumption in 2019. Among male individuals, tobacco smoking and alcohol consumption were responsible for a large proportion of LOC deaths globally (42.3% [95% UI, 35.2%-48.6%] and 40.2% [95% UI, 33.3%-46.8%], respectively). For male individuals in the younger age groups (≤54 years old) and in the oldest age group (≥95 years old), alcohol consumption was a more important risk factor than smoking (Figure 4). Among female individuals, the highest proportion of risk-attributable LOC deaths globally were due to chewing tobacco (27.6% [95% UI, 21.5%-33.8%]; Figure 4), with this burden concentrated in South and Southeast Asia (eFigure 16 in Supplement 1).

Concerning OPC, for male individuals older than 45 years and female individuals older than 50 years, the highest proportion of deaths globally was attributable to smoking tobacco; in younger age groups, however, alcohol consumption played the greater role (Figure 4). Globally, 55.8% (95% UI, 49.2%-62.0%) of OPC deaths among male individuals were attributable to tobacco smoking and 40.0% (95% UI, 31.8%-48.1%) to alcohol consumption; among female individuals, 17.4% (95% UI, 13.8%-21.2%) were due to smoking and 10.1% (95% UI, 7.1%-13.3%) to alcohol. For risk-attributable DALYs, see eFigure 17 in Supplement 1 and for results by country, eTables 12 and 13 in Supplement 1.

## Discussion

This study provides an updated and comprehensive overview of lip, oral, and other pharyngeal cancer burden in the past 30 years, globally and by region using GBD 2019 estimates, including for areas where observed data are scarce. It is also, to our knowledge, the first report of the burden of LOC and OPC attributable to risk factors globally, providing important information for addressing these cancers around the world. Disparities in ASIRs and ASMRs and trends across the SDI spectrum suggested that populations from regions with a lower level of sociodemographic development have a higher chance of death when affected by LOC or OPC, with South Asia carrying substantial LOC and OPC burden. Smoking, chewing tobacco, and alcohol were substantial contributors to LOC and OPC deaths and DALYs and are crucial targets to decreasing future burden of LOC and OPC globally.

Throughout the entire study period, the low and low-middle SDI strata had the highest LOC and OPC ASMRs and age-standardized DALY rates, even though they did not always have the highest ASIRs. The differences between ASMRs and ASIRs were smaller in the low and low-middle SDI regions, particularly for OPC. The ASMRs and age-standardized DALY rates for LOC and OPC showed a pattern of stability across time for almost all SDI groups, but the high and high-middle SDI groups exhibited a decline in these rates for LOC in the past 2 decades. These results may reflect different regional patterns of exposure to the risk factors estimated in this study (tobacco and alcohol), as well as other risk factors not estimated herein, such as oncogenic HPV infection, which is responsible for higher incidence rates of HPV-associated oropharynx cancer in wealthier countries^9^ and is reported to carry lower mortality.^21,22^ The latter may also be one factor contributing to the increasing trend of ASIRs in the high SDI quintile noted in this analysis.^9^ Smoking may lead to poorer survival in HPV-associated oropharyngeal cancer and was found to contribute to a large proportion of OPC deaths and DALYs in High-income regions in this analysis, highlighting the critical role of this risk factor even in settings where HPV-associated oropharyngeal cancer may be more prevalent.^9,23^ The differences in OPC ASMRs in the setting of similar ASIRs that were identified in some SDI strata (eg, high-middle and low SDI) could suggest that fewer patients survive their OPC diagnoses in the most impoverished parts of the world, potentially due to later-stage diagnoses and/or less access to cancer treatment.^24,25^

At the World Health Assembly in 2021, the WHO adopted a resolution on oral health—the WHA74.5 resolution^15^—which was a considerable step forward for the oral health agenda. This resolution enabled the development of a global strategy on oral health, a global action plan adopted by the member states in the 2022 World Health Assembly, which has the vision to reach universal health coverage for oral health for all individuals and communities by 2030.^26^ In addition, the WHO global noncommunicable diseases action plan for 2013 to 2030 advocates for specific interventions for oral cancer, including screening in high-risk groups linked with timely diagnostic and comprehensive cancer treatment.^27^ These efforts provide a collective opportunity to improve patient outcomes in a historically neglected area of health care.^28,29^

The present results suggest that inequities exist in LOC and OPC burden worldwide, which should be considered in oral health planning and implementation initiatives. Public health strategies to control exposure to major risk factors and promote access to early diagnosis and treatment in low- and low-middle–income regions are urgently needed, given the higher mortality rates in lower SDI countries. Including oral health care as part of the universal health care agenda can support early diagnosis and access to timely treatment of LOC and OPC; oral health care is unaffordable for many individuals around the world, and most health systems with universal coverage do not currently include it.^30^ Delay in diagnosis and treatment of these cancers negatively affects survival, so referral pathways capable of quick diagnosis of suspected LOC and OPC cases and treatment of those confirmed are crucial.^24^ Finally, monitoring actions should include creating and expanding population-based registration systems that include information on the staging of these cancers at the time of diagnosis and their subsequent outcomes.

This study showed that smoking tobacco remains an important risk factor for oral cavity and other pharyngeal cancers globally. Tobacco control efforts have been mobilized over the past several decades, including the WHO Framework Convention on Tobacco Control in 2003,^31^ the first global health treaty aimed at reducing tobacco consumption in member states. Evidence indicates that tobacco control measures adopted by the countries participating in the Framework Convention on Tobacco Control effectively reduced the prevalence of smoking. However, the pace of implementation of these measures has been heterogeneous around the world, and the tobacco epidemic is still far from over.^32,33^ The high percentage of LOC and OPC deaths and DALYs attributable to smoking tobacco in 2019 reinforces the need to strengthen the implementation of tobacco control measures.

The percentage of DALYs and deaths for LOC attributable to chewing tobacco is concentrated in certain world regions, mainly in South and Southeast Asia. The largest ASMRs and ASIRs for LOC and OPC in 2019 occurred in South Asia, and for LOC these rates were more than double the next highest region rates. The GBD 2019 study estimated that 83.3% (95% UI, 82.2%-84.2%) of the chewing tobacco users in 2019 lived in South Asia,^34^ with the present study suggesting that chewing tobacco in South and Southeast Asia is especially relevant among female individuals. The critical contribution of chewing tobacco to oral cavity cancer burden among female individuals is concerning because the prevalence of this risk factor in female individuals has been constant in recent decades, unlike smoking tobacco, which has been decreasing.^34^ These results imply that tobacco control initiatives need to be expanded, intensified, or better able to address chewing tobacco in some world regions.^35^ Because chewing tobacco is a habit related to local culture and beliefs,^7^ these initiatives should be planned in close collaboration with groups and institutions that are aware of the local challenges and opportunities.

The present results indicate that a large proportion of LOC and OPC deaths in 2019 were attributable to alcohol, particularly in young men. A previous GBD study indicated that the prevalence of alcohol consumption varied considerably worldwide, being higher in high SDI areas, and revealed that alcohol and its effects on health may become an increasing challenge as regions advance across the SDI spectrum.^36^ This suggests that low and low-middle SDI regions could benefit from policies aimed at reducing population-level exposure to alcohol while this habit is not so widespread, in addition to addressing this risk factor in higher SDI countries. While the synergistic effect of alcohol and tobacco on the risk of developing LOC and OPC is well recognized in the literature,^37^ the importance of alcohol alone is less explored; however, the present results on the critical role of alcohol as a risk factor for LOC and OPC are in line with previous studies.^6,38^

### Limitations

While the GBD 2019 study provided useful estimates of global LOC and OPC burden, there are several limitations. Locations where observed data on disease burden were unavailable are reported with appropriate uncertainty, but their estimation is limited by the data available across space and time, highlighting the importance of expanding cancer and vital registration systems. The availability and quality of primary data are also a limitation in the estimation of risk-attributable cancer burden.^16^ In addition, there are limitations more specific to the estimation of LOC and OPC. Currently, it is not possible in the GBD study to separately analyze cancers at a more granular level than the LOC and OPC categories reported herein, and the current GBD classification groups include some anatomical cancer sites that do not share the same pathophysiological determinants, such as lip cancer with other sites of the oral cavity. Potential improvements to the GBD categorization of these cancers would include, depending on data availability, (1) the separation of lip, salivary gland, oropharynx, and hypopharynx cancers into individual categories and (2) the inclusion of cancer of the base of the tongue in the oropharyngeal cancer category, instead of analyzing it as part of oral cavity cancers.^39,40^ Finally, the current study did not estimate the burden of LOC and OPC attributable to other risk factors for these diseases, such as HPV infection, an important and growing risk factor for OPC,^22,41^ and betel quid without tobacco consumption for LOC.^4^

## Conclusions

In this systematic analysis, disparities in LOC and OPC incidence, mortality, and DALY rates across the SDI spectrum were evident in 2019. Actions to reduce the burden of LOC and OPC through improvements in early diagnosis, access to treatment, and reduction of exposure to risk factors should consider the inequities in the global distribution of these diseases. Including oral health into universal health coverage is one crucial strategy to reach this goal, as part of comprehensive cancer control efforts. Tobacco and alcohol use are important targets to decreasing the future burden of LOC and OPC globally.

## Notes

Supplement 1.
eMethods.
**eFigure 1.** Input data and methodological summary for mortality and Years of Life Lost (YLLs) for all cancers, including LOC and OPC **eTable 1.** List of International Classification of Diseases (ICD) codes mapped to the Global Burden of Disease causes Lip and oral cavity cancer and Other pharynx cancer for cancer incidence data **eTable 2.** Age restrictions for LOC and OPC in GBD 2019 modeling **eTable 3.** Covariates for lip and oral cavity cancer **eTable 4. **Covariates for other pharynx cancer **eFigure 2.** Flowchart of GBD cancer incidence and Years Lived with Disability (YLDs) estimation, including LOC and OPC **eTable 5.** Duration (months) of each sequalae for LOC and OPC **eTable 6.** Lay description of cancer states and corresponding disability weights
eReferences
**eFigure 3.** Socio-demographic Index quintiles for the Global Burden of Disease Study 2019 **eTable 7.** Socio-demographic Index (SDI) quintiles for countries and territories estimated in GBD 2019 **eFigure 4.** Map of GBD world super-regions, 2019 **eFigure 5.** Map of GBD world regions, 2019 **eTable 8.** Global and regional deaths, incidence, and DALYs counts and age-standardized rates (per 100 000) for Lip and oral cavity, both sexes combined, in 2019, and change in age-standardized rates from 1990 to 2019 **eTable 9.** Global and regional deaths, incidence, and DALYs counts and age-standardized rates (per 100 000) for Other pharynx cancer, both sexes combined, in 2019, and change in age-standardized rates from 1990 to 2019 **eTable 10.** Deaths, incidence, and DALYs counts and age-standardized rates (per 100 000) for Lip and oral cavity cancer (LOC), both sexes combined, by country or territory, in 2019, and change in age-standardized rates from 1990 to 2019 **eTable 11.** Deaths, incidence, and DALYs counts and age-standardized rates (per 100 000) for Other pharynx cancer (OPC), both sexes combined, by country or territory, in 2019, and change in age-standardized rates from 1990 to 2019 **eFigure 6.** Global map of age-standardized incidence rate quintiles for A) lip and oral cavity cancer, and B) other pharynx cancer, both sexes combined, 2019 **eFigure 7.** Global map of A) age-standardized mortality rate quintiles, and B) age-standardized incidence rate quintiles for lip and oral cavity cancer, males, 2019 **eFigure 8.** Global map of A) age-standardized mortality rate quintiles, and B) age-standardized incidence rate quintiles for other pharynx cancer, males, 2019 **eFigure 9.** Global map of A) age-standardized mortality rate quintiles, and B) age-standardized incidence rate quintiles for lip and oral cavity cancer, females, 2019 **eFigure 10.** Global map of A) age-standardized mortality rate quintiles, and B) age-standardized incidence rate quintiles for other pharynx cancer, females, 2019 **eFigure 11.** Global absolute DALYs and age-specific DALY rates (per 100,000) for Lip and oral cavity cancer, and Other pharynx cancer by 5-year age group and sex in 2019 **eFigure 12.** Time trends of age-standardized DALY rates for Lip and oral cavity cancer and Other pharynx cancer from 1990 to 2019, by SDI quintile **eFigure 13.** Time trends of age-standardized deaths, incidence, and DALY rates for Lip and oral cavity cancer and Other pharynx cancer from 1990 to 2019, by sex **eFigure 14.** Time trends of age-specific deaths, incidence, and DALY rates for Lip and oral cavity cancer and Other pharynx cancer from 1990 to 2019, by ten-year age group globally **eFigure 15.** Time trends of age-standardized deaths, incidence, and DALY rates for Lip and oral cavity cancer and Other pharynx cancer from 1990 to 2019, by GBD super-region **eFigure 16.** Proportion of deaths attributable to risk factors for Lip and oral cavity cancer and Other pharynx cancer for males and females in 2019 by GBD world region **eFigure 17.** Proportion of DALYs attributable to risk factors for Lip and oral cavity cancer and Other pharynx cancer A) by five-year age group globally, and B) by GBD world region, for males and females in 2019 **eTable 12.** Proportion of Lip and oral cavity cancer (LOC) deaths and DALYs attributable to risk factors, in 2019, by country or territory, both sexes combined **eTable 13.** Proportion of Other pharynx cancer (OPC) deaths and DALYs attributable to risk factors, in 2019, by country or territory, both sexes combined

Supplement 2. Data Sharing Statement
