# Supplementary material for: The Global, Regional, and National Burden of Adult Lip, Oral, and Pharyngeal Cancer in 204 Countries and Territories: A Systematic Analysis for the Global Burden of Disease Study 2019
Source: JAMA Oncol. 2023 Sep 7;9(10):1401–16. doi: 10.1001/jamaoncol.2023.2960 (PMC10485745; doi:10.1001/jamaoncol.2023.2960)
Supplement: Supplement 1. — eMethods. eFigure 1. Input data and methodological summary for mortality and Years of Life Lost (YLLs) for all cancers, including LOC and OPC eTable 1. List of International Classification of Diseases (ICD) codes mapped to the Global Burden of Disease causes Lip and oral cavity cancer and Other pharynx cancer for cancer incidence data eTable 2. Age restrictions for LOC and OPC in GBD 2019 modeling eTable 3. Covariates for lip and oral cavity cancer eTable 4. Covariates for other pharynx cancer eFigure 2. Flowchart of GBD cancer incidence and Years Lived with Disability (YLDs) estimation, including LOC and OPC eTable 5. Duration (months) of each sequalae for LOC and OPC eTable 6. Lay description of cancer states and corresponding disability weights eReferences eFigure 3. Socio-demographic Index quintiles for the Global Burden of Disease Study 2019 eTable 7. Socio-demographic Index (SDI) quintiles for countries and territories estimated in GBD 2019 eFigure 4. Map of GBD world super-regions, 2019 eFigure 5. Map of GBD world regions, 2019 eTable 8. Global and regional deaths, incidence, and DALYs counts and age-standardized rates (per 100 000) for Lip and oral cavity, both sexes combined, in 2019, and change in age-standardized rates from 1990 to 2019 eTable 9. Global and regional deaths, incidence, and DALYs counts and age-standardized rates (per 100 000) for Other pharynx cancer, both sexes combined, in 2019, and change in age-standardized rates from 1990 to 2019 eTable 10. Deaths, incidence, and DALYs counts and age-standardized rates (per 100 000) for Lip and oral cavity cancer (LOC), both sexes combined, by country or territory, in 2019, and change in age-standardized rates from 1990 to 2019 eTable 11. Deaths, incidence, and DALYs counts and age-standardized rates (per 100 000) for Other pharynx cancer (OPC), both sexes combined, by country or territory, in 2019, and change in age-standardized rates from 1990 to 2019 eFigure 6. Global map of age-standardized incide [file jamaoncol-e232960-s001.pdf]

Supplemental Online Content

Cunha AR, Compton K, Xu R, et al. The global, regional, and national burden of adult lip, oral, and pharyngeal cancer in 204 countries and territories: a systematic analysis for the Global Burden of Disease Study 2019. *JAMA Oncol*. Published online September 7, 2023. doi:10.1001/jamaoncol.2023.2960

eMethods.
eFigure 1. Input data and methodological summary for mortality and Years of Life Lost (YLLs) for all cancers, including LOC and OPC
eTable 1. List of International Classification of Diseases (ICD) codes mapped to the Global Burden of Disease causes Lip and oral cavity cancer and Other pharynx cancer for cancer incidence data
eTable 2. Age restrictions for LOC and OPC in GBD 2019 modeling
eTable 3. Covariates for lip and oral cavity cancer
eTable 4. Covariates for other pharynx cancer
eFigure 2. Flowchart of GBD cancer incidence and Years Lived with Disability (YLDs) estimation, including LOC and OPC
eTable 5. Duration (months) of each sequalae for LOC and OPC
eTable 6. Lay description of cancer states and corresponding disability weights
eReferences
eFigure 3. Socio-demographic Index quintiles for the Global Burden of Disease Study 2019
eTable 7. Socio-demographic Index (SDI) quintiles for countries and territories estimated in GBD 2019
eFigure 4. Map of GBD world super-regions, 2019
eFigure 5. Map of GBD world regions, 2019
eTable 8. Global and regional deaths, incidence, and DALYs counts and age-standardized rates (per 100 000) for Lip and oral cavity, both sexes combined, in 2019, and change in age-standardized rates from 1990 to 2019
eTable 9. Global and regional deaths, incidence, and DALYs counts and age-standardized rates (per 100 000) for Other pharynx cancer, both sexes combined, in 2019, and change in age-standardized rates from 1990 to 2019
eTable 10. Deaths, incidence, and DALYs counts and age-standardized rates (per 100 000) for Lip and oral cavity cancer (LOC), both sexes combined, by country or territory, in 2019, and change in age-standardized rates from 1990 to 2019
eTable 11. Deaths, incidence, and DALYs counts and age-standardized rates (per 100 000) for Other pharynx cancer (OPC), both sexes combined, by country or territory, in 2019, and change in age-standardized rates from 1990 to 2019
eFigure 6. Global map of age-standardized incidence rate quintiles for A) lip and oral cavity cancer, and B) other pharynx cancer, both sexes combined, 2019
eFigure 7. Global map of A) age-standardized mortality rate quintiles, and B) age-standardized incidence rate quintiles for lip and oral cavity cancer, males, 2019
eFigure 8. Global map of A) age-standardized mortality rate quintiles, and B) age-standardized incidence rate quintiles for other pharynx cancer, males, 2019

eFigure 9. Global map of A) age-standardized mortality rate quintiles, and B) age-standardized incidence rate quintiles for lip and oral cavity cancer, females, 2019
eFigure 10. Global map of A) age-standardized mortality rate quintiles, and B) age-standardized incidence rate quintiles for other pharynx cancer, females, 2019
eFigure 11. Global absolute DALYs and age-specific DALY rates (per 100,000) for Lip and oral cavity cancer, and Other pharynx cancer by 5-year age group and sex in 2019
eFigure 12. Time trends of age-standardized DALY rates for Lip and oral cavity cancer and Other pharynx cancer from 1990 to 2019, by SDI quintile
eFigure 13. Time trends of age-standardized deaths, incidence, and DALY rates for Lip and oral cavity cancer and Other pharynx cancer from 1990 to 2019, by sex
eFigure 14. Time trends of age-specific deaths, incidence, and DALY rates for Lip and oral cavity cancer and Other pharynx cancer from 1990 to 2019, by ten-year age group globally
eFigure 15. Time trends of age-standardized deaths, incidence, and DALY rates for Lip and oral cavity cancer and Other pharynx cancer from 1990 to 2019, by GBD super-region
eFigure 16. Proportion of deaths attributable to risk factors for Lip and oral cavity cancer and Other pharynx cancer for males and females in 2019 by GBD world region
eFigure 17. Proportion of DALYs attributable to risk factors for Lip and oral cavity cancer and Other pharynx cancer A) by five-year age group globally, and B) by GBD world region, for males and females in 2019
eTable 12. Proportion of Lip and oral cavity cancer (LOC) deaths and DALYs attributable to risk factors, in 2019, by country or territory, both sexes combined
eTable 13. Proportion of Other pharynx cancer (OPC) deaths and DALYs attributable to risk factors, in 2019, by country or territory, both sexes combined
eFigure 18. Proportion of deaths attributable to risk factors for A) Lip and oral cavity cancer and B) Other pharynx cancer for males and females in 2019

This supplemental material has been provided by the authors to give readers additional information about their work.

Supplement to: GBD 2019 Lip, Oral, and Pharyngeal Cancer Collaborators. The global, regional, and national burden of lip, oral, and pharyngeal cancer in 204 countries and territories: a systematic analysis for the Global Burden of Disease Study 2019.

This appendix provides further methodological detail for "The global, regional, and national burden of lip, oral, and pharyngeal cancer in 204 countries and territories: a systematic analysis for the Global Burden of Disease study 2019." This study complies with the Guidelines for Accurate and Transparent Health Estimates Reporting (GATHER) recommendations.[1] It includes detailed tables and information on data to maximize transparency in our estimation processes and provides a comprehensive description of analytical steps. A completed GATHER checklist can be found in the eMethods section.

Please note that portions of this supplement were copied from the supplementary content to the recent GBD publications:

> Kocarnik J, Compton K, Dean FE, et al. Cancer incidence, mortality, years of life lost, years lived with disability, and disability-adjusted life years for 29 cancer groups from 2010 to 2019: a systematic analysis for the Global Burden of Disease Study 2019. *JAMA Oncol.* 2022; **8**(3): 420–444. doi:10.1001/jamaoncol.2021.6987.[2];
>
> Force LM, Abdollahpour I, Advani SM, et al. The global burden of childhood and adolescent cancer in 2017: an analysis of the Global Burden of Disease Study 2017. *Lancet Oncol.* 2019; **20**: 1211–25.[3];
>
> Vos T, Lim SS, Abbafati C, et al. Global burden of 369 diseases and injuries in 204 countries and territories, 1990–2019: a systematic analysis for the Global Burden of Disease Study 2019. *Lancet* 2020; **396**: 1204–22.[4];
>
> and
>
> Murray CJL, Aravkin AY, Zheng P, et al. Global burden of 87 risk factors in 204 countries and territories, 1990–2019: a systematic analysis for the Global Burden of Disease Study 2019. *Lancet* 2020; **396**: 1223–49.[5]

References are provided and renumbered for reproduced sections.

eMethods.

THE GLOBAL BURDEN OF DISEASE (GBD) STUDY

The Global Burden of Disease (GBD) study was created in an effort to establish comprehensive and comparable health metrics. A key principle in the GBD approach to estimation of disease burden is that an individual can have only one cause of death, while recognising that this may underestimate disease burden due to intermediate causes of death. In addition to reporting estimates of mortality and years of life lost (YLLs) for over 300 diseases and injuries, the GBD study also quantifies non-fatal components of disease including years lived with disability (YLDs) and disability-adjusted life-years (DALYs), a metric that represents a combination of both the fatal and non-fatal components of disease. The GBD approach uses all relevant data sources, rather than a single type of data. Finally, as there is continual methodological refinement with each GBD iteration, the results in each successive iteration supersede the results of prior GBD studies for the entire newly estimated time series. A protocol for the GBD study can be found online at http://www.healthdata.org/sites/default/files/files/Projects/GBD/GBD_Protocol.pdf.

GATHER[1] Guidelines Checklist

Item #	Checklist item	Reported on page #
**Objectives and funding**		
**1**	Define the indicator(s), populations (including age, sex, and geographic entities), and time period(s) for which estimates were made.	Appendix pg. 7
**2**	List the funding sources for the work.	See main manuscript
**Data Inputs**		
For all data inputs from multiple sources that are synthesized as part of the study:		
**3**	Describe how the data were identified and how the data were accessed.	Appendix pg. 7
**4**	Specify the inclusion and exclusion criteria. Identify all ad-hoc exclusions.	Appendix pg. 8
**5**	Provide information on all included data sources and their main characteristics. For each data source used, report reference information or contact name/institution, population represented, data collection method, year(s) of data collection, sex and age range, diagnostic criteria or measurement method, and sample size, as relevant.	http://ghdx.healthdata.org/gbd-2019
**6**	Identify and describe any categories of input data that have potentially important biases (e.g., based on characteristics listed in item 5).	Appendix pg. 8–9
For data inputs that contribute to the analysis but were not synthesized as part of the study:		
**7**	Describe and give sources for any other data inputs.	http://ghdx.healthdata.org/gbd-2019
For all data inputs:		
**8**	Provide all data inputs in a file format from which data can be efficiently extracted (e.g., a spreadsheet rather than a PDF), including all relevant meta-data listed in item 5. For any data inputs that cannot be shared because of ethical or legal reasons, such as third-party ownership, provide a contact name or the name of the institution that retains the right to the data.	http://ghdx.healthdata.org/gbd-2019
**Data analysis**		
**9**	Provide a conceptual overview of the data analysis method. A diagram may be helpful.	Appendix pg. 10 & 22 (eFigures 1 & 2)
**10**	Provide a detailed description of all steps of the analysis, including mathematical formulae. This description should cover, as relevant, data cleaning, data pre-processing, data adjustments and weighting of data sources, and mathematical or statistical model(s).	Appendix pg. 11–48
**11**	Describe how candidate models were evaluated and how the final model(s) were selected.	Found in *Section 3: Causes of death modelling methods* of the Supplementary appendix 1 to "Global burden of 369 diseases and injuries in 204 countries and territories, 1990–2019: a systematic analysis for the Global Burden of Disease Study 2019."[4] Details of covariate selection for cancer models can be found in: Appendix pg. 19–20 (eTables 3 & 4)

**12**	Provide the results of an evaluation of model performance, if done, as well as the results of any relevant sensitivity analysis.	Found in eTable 10 of the Supplementary Appendix to "Cancer incidence, mortality, years of life lost, years lived with disability, and disability-adjusted life years for 29 cancer groups from 2010 to 2019: a systematic analysis for the Global Burden of Disease Study 2019."[2]
**13**	Describe methods for calculating uncertainty of the estimates. State which sources of uncertainty were, and were not, accounted for in the uncertainty analysis.	Appendix pg. 26
**14**	State how analytic or statistical source code used to generate estimates can be accessed.	http://ghdx.healthdata.org/gbd-2019/code
**Results and Discussion**		
**15**	Provide published estimates in a file format from which data can be efficiently extracted.	GBD 2019 estimates are available online (https://vizhub.healthdata.org/gbd-compare/ and http://ghdx.healthdata.org/gbd-results-tool)
**16**	Report a quantitative measure of the uncertainty of the estimates (e.g., uncertainty intervals).	See main manuscript, "Results"
**17**	Interpret results in light of existing evidence. If updating a previous set of estimates, describe the reasons for changes in estimates.	See main manuscript, "Discussion"
**18**	Discuss limitations of the estimates. Include a discussion of any modelling assumptions or data limitations that affect interpretation of the estimates.	Appendix pg. 27

Part I – Burden of Diseases Analysis

1. Overview and definition of indicator

This article presents and details the estimates (incidence, mortality, and DALYs) produced by the GBD Study 2019 for two types of cancer: Lip and oral cavity cancer (LOC) and Other pharynx cancer (OPC). "Other pharynx cancer" is used throughout this appendix for consistency with the standard naming system of the GBD 2019 study, but the term is considered interchangeable with "other pharyngeal cancer" in the main text for the purpose of this analysis. Details on LOC and OPC coding by the International Classification of Diseases (ICD) ninth and tenth revisions[6,7] can be found on pages 11 & 12 of this appendix. We present the estimates by sex, five-year GBD age group (20 to 24, 25 to 29, 30 to 34, etc. until 95+) and region, for the years 1990–2019. The report by region considered three geographic classifications: GBD super-regions (seven categories), GBD world regions (21 categories), and countries and territories (n = 204). The regions were also classified by Socio-demographic Index (SDI) quintiles, which is explained further in this appendix.

2. GBD causes of death database

For GBD 2019 Study, all available data on causes of death (CoD) are standardized and pooled into a single database used to generate cause-specific mortality estimates by age, sex, year, and region. Figures S1 and S2 (pages 1439 and 1440) of Appendix 1 of "Global burden of 369 diseases and injuries in 204 countries and territories, 1990–2019: a systematic analysis for the Global Burden of Disease Study 2019"[4] present the high-level view of data inputs, analytical steps, and outputs of the CoD analysis frame. In this appendix, we will highlight the processes for cancer estimation in GBD, specifically for LOC and OPC.

3. Data sources

The GBD 2019 Study synthesizes many data input sources, including surveys, censuses, vital statistics, and other health-related data sources. The data from these sources are used to estimate morbidity, illness, and injury, and attributable risk for 204 countries and territories from 1990 to 2019, while mortality deaths are estimated from 1980 to 2019. The input sources are accessible through an interactive citation tool available in the GHDx: http://ghdx.healthdata.org/gbd-2019/data-input-sources.

3.1. Cancer registry (CR) data sources

Cancer incidence and mortality data were sought from individual population-based cancer registries, such as the Surveillance, Epidemiology, and End Results (SEER) Program[8]; provided by collaborators; or downloaded from aggregated databases of cancer registry data such as "Cancer Incidence in Five Continents" (CI5)[9–19], NORDCAN[20], and EUREG[21]. Only population-based cancer registries were included, with inclusion criteria that they included all cancers (i.e., were not specialty registries), reported data for all age groups (except for pediatric cancer registries), and reported data for both sexes. Pathology-based cancer registries were included if they had a defined population. Hospital-based cancer registries were excluded. Redundant cancer registry data were excluded from either the final incidence data input or the MIR model input if a more detailed source (e.g., providing more detailed age or diagnostic groups) was available for the same population. Preference was given to registries with national coverage over those with only local coverage, except those from countries where the GBD study provides subnational estimates. Data were excluded if the coverage population was unknown, except for in high SDI quintile locations with full geographic coverage where the GBD population could be substituted. A list of the cancer registries included in our analysis and the years covered can be found in the online GBD citation tool http://ghdx.healthdata.org/gbd-2019.

3.2. Mortality-to-incidence ratio (MIR) data sources

Most cancer registries only report cancer incidence. However, if a cancer registry also reported cancer mortality, mortality data were also extracted. CR sources with matching incidence and mortality data were used in the mortality-to-incidence ratio estimation.[2]

3.3. Cancer mortality data in the cause of death (CoD) database other than cancer registry data

In addition to cancer registry data, the GBD cause of death (CoD) database also contains cancer mortality data originating from multiple sources, including vital registration (VR) and verbal autopsy (VA) data. Most of the cause of death data in GBD, including mortality from cancer, is vital registration data obtained from the World Health Organization (WHO) Mortality Database. VR is also obtained from country-specific mortality databases operated by official offices. In countries without VR systems, VA studies are a viable data source to inform CoD. VA data are obtained by trained interviewers who use a standardized questionnaire to ask relatives about the signs, symptoms, and demographic characteristics of recently deceased family members. CoD is assigned based on the answers to the questionnaires. Each cause is coded directly to the most detailed CoD when possible, whereas cause codes in data tabulated by International Classification of Disease (ICD-) are coded to aggregated cause groups. A detailed description of the data sources and processing steps for the CoD database can be found in Section 2 (page 20), Appendix 1 of "Global burden of 369 diseases and injuries in 204 countries and territories, 1990–2019: a systematic analysis for the Global Burden of Disease Study 2019,"[4] as well as in the online GBD citation tool http://ghdx.healthdata.org/gbd-2019.

3.4. Bias of categories of input cancer data

Potential biases of the input data included for the CoD database can also be found in the Supplementary Appendix 1 to the GBD 2019 paper "Global burden of 369 diseases and injuries in 204 countries and territories, 1990–2019: a systematic analysis for the Global Burden of Disease Study 2019."4 Cancer registry data can be biased in multiple ways. A high proportion of ill-defined cancer cases in the cancer registry data requires redistribution of these cases to other cancers, which introduces a potential for bias. Changes between coding systems can lead to artificial differences in disease estimates; however, we adjust for this bias by mapping the different coding systems to GBD cancer causes. Underreporting of cancers that require advanced diagnostic techniques can be an issue in cancer registries from low-income countries. On the other hand, misclassification of metastatic sites as primary cancer can lead to overestimation of cancer sites that are common sites for metastases. Since many cancer registries are located in urban areas, the representativeness of the registry for the general non-urban population can also be problematic. The accuracy of mortality data reported in cancer registries usually depends on the quality of the vital registration system. If the vital registration system is incomplete or of poor quality, the mortality-to-incidence ratio can be biased to lower ratios.

4. CoD cause-specific modeling descriptions – neoplasms

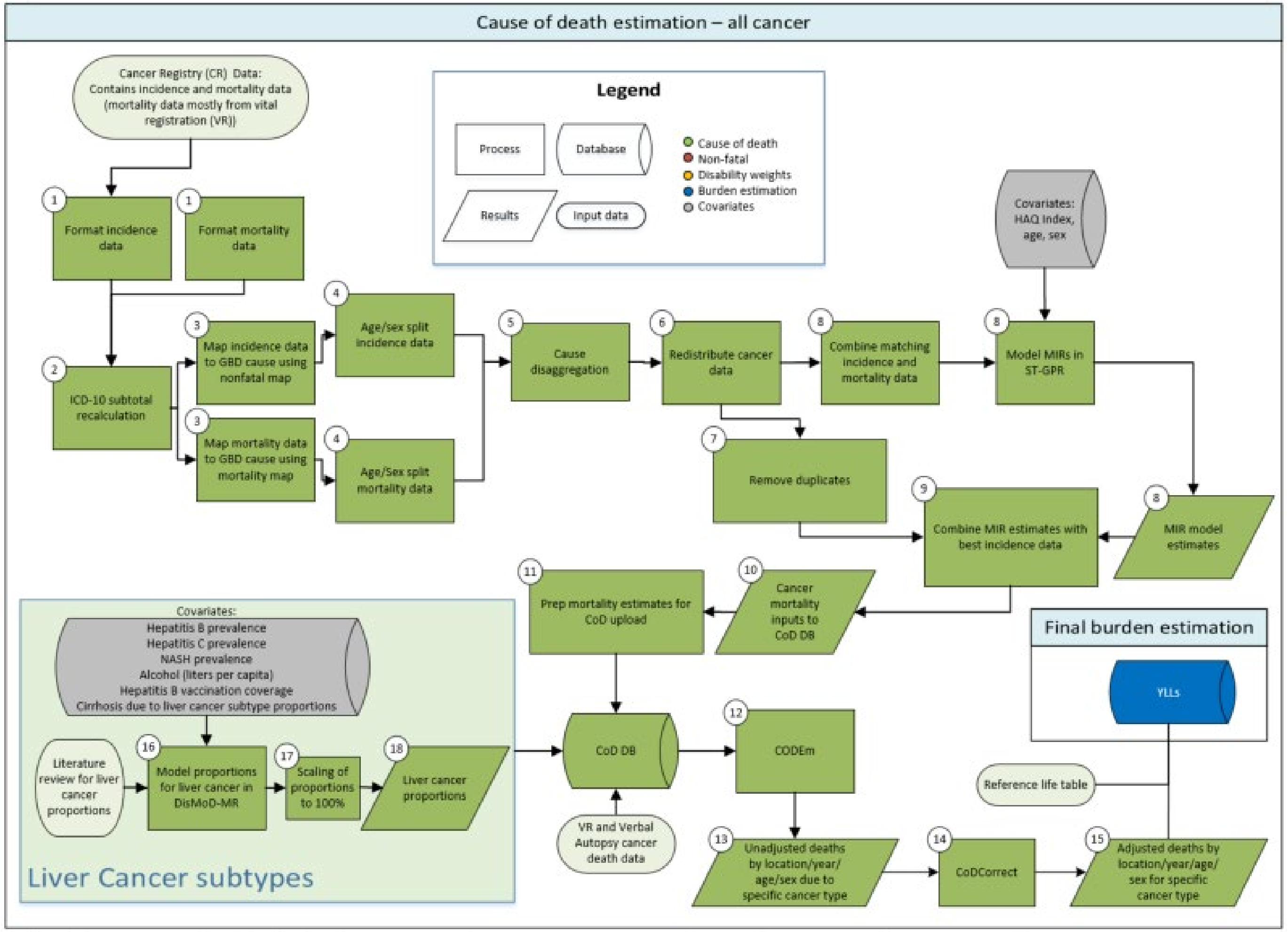

eFigure 1. Input data and methodological summary for mortality and Years of Life Lost (YLLs) for all cancers, including LOC and OPC

Abbreviations: CoD, causes of death; CODEm, cause of death ensemble model; DB, database; DisMod-MR, disease model – Bayesian meta-regression; HAQ Index, Healthcare Access and Quality Index; ICD, International Classification of Diseases; ST-GPR, spaciotemporal Gaussian process regression; MIR, mortality-to-incidence ratio; NASH, nonalcoholic steatohepatitis; VR, vital registration; YLL, years of life lost

4.1 Cancer registry data processing

Cancer registry data went through multiple processing steps before entering the CoD database,

1. Formatting incidence and mortality data. First, the original data are transformed into standardized files, which included standardization of format, categorization, and registry names (#1 in eFigure 1).

2. Subtotal recalculation. Some cancer registries report individual codes as well as aggregated totals. An example of this would be where the registry data reports C18, C19, and C20 individually, and also the aggregated group of C18–C20 (colon and rectum cancer). The data processing step, "subtotal recalculation" (#2 in eFigure 1), verifies these totals and subtracts the values of any individual codes from the aggregates.

3. Mapping data to GBD causes. In the third step (#3 in eFigure 1), cancer registry incidence data and cancer registry mortality data are mapped to GBD causes. A different map is used for incidence and for mortality data because of the assumption that there are no deaths for certain cancers. One example is benign or in situ neoplasms. Because cancer registries do not collect non-malignant neoplasms in a standardized way, any benign or in situ neoplasms reported in a cancer registry incidence dataset are dropped from that dataset. The same neoplasms reported in a cancer registry mortality dataset are instead mapped to the respective invasive cancer. Maps of ICD-codes to GBD LOC and OPC causes for incidence and mortality data can be found in eTable 1. A full list of ICD mapping to all cancers estimated in the GBD study can be found in eTables 1 & 2 in the Supplementary Appendix to "Cancer incidence, mortality, years of life lost, years lived with disability, and disability-adjusted life years for 29 cancer groups from 2010 to 2019: a systematic analysis for the Global Burden of Disease Study 2019."[2]

eTable 1. List of International Classification of Diseases (ICD) codes mapped to the Global Burden of Disease causes Lip and oral cavity cancer and Other pharynx cancer for cancer incidence data

Cause	ICCC3	ICD-10	ICD-9
Lip and oral cavity cancer	XIf1	C00, C00.0, C00.1, C00.2, C00.3, C00.4, C00.5, C00.6, C00.8, C00.9, C01, C01.9, C02, C02.0, C02.1, C02.2, C02.3, C02.4, C02.8, C02.9, C03, C03.0, C03.1, C03.9, C04, C04.0, C04.1, C04.8, C04.9, C05, C05.0, C05.1, C05.2, C05.8, C05.9, C06, C06.0, C06.1, C06.2, C06.8, C06.80, C06.89, C06.9, C07, C07.0, C07.9, C08, C08.0, C08.1, C08.8, C08.9	140, 140.0, 140.1, 140.2, 140.3, 140.4, 140.5, 140.6, 140.7, 140.8, 140.9, 141, 141.0, 141.1, 141.2, 141.3, 141.4, 141.5, 141.6, 141.8, 141.9, 142, 142.0, 142.1, 142.2, 142.3, 142.8, 142.9, 143, 143.0, 143.1, 143.8, 143.9, 144, 144.0, 144.1, 144.4, 144.8, 144.9, 145, 145.0, 145.1, 145.2, 145.3, 145.4, 145.5, 145.6, 145.8, 145.9
Other pharynx cancer	NA	C09, C09.0, C09.1, C09.8, C09.9, C1, C10, C10.0, C10.1, C10.2, C10.3, C10.4, C10.8, C10.9, C12, C12.0, C12.9, C13, C13.0, C13.1, C13.2, C13.8, C13.9	146, 146.0, 146.1, 146.2, 146.3, 146.4, 146.5, 146.6, 146.7, 146.8, 146.9, 148, 148.0, 148.1, 148.2, 148.3, 148.4, 148.5, 148.8, 148.9

4. Age/sex splitting. In the fourth data processing step (#4 in eFigure 1), cancer registry data are standardized to the GBD age groups. For each cancer, the minimum age group estimated was determined as the youngest age-group where SEER reported at least 50 cases over the period 1990 to 2015.[8] The modeled starting and ending age groups for each cancer included in this analysis are reported in eTable 2. Reference global age-specific incidence rates are generated using hospital inpatient data as described in Section 4.3 of the appendix to the GBD 2019 paper "Global burden of 369 diseases and injuries in 204 countries and territories, 1990–2019: a systematic analysis for the Global Burden of Disease Study 2019."[4] Reference age-specific mortality rates were generated using aggregated deaths from processed VR data, using the approach described in Section 2.5 of the appendix to the aforementioned GBD 2019 paper.

For incidence or mortality datasets that require age-splitting, age-specific proportions are then generated by applying the reference age-specific rates to the registry population to produce the expected number of cases (or deaths for a mortality dataset) for that registry by age. The expected number of cases (or deaths) for each sex, age, and cancer were normalized to 1, creating final, age-specific proportions. These proportions were then applied to the total number of cases (or deaths) by sex and cancer to get the GBD age group-specific number of cases (or deaths) related to that dataset.

In the rare case that the cancer registry only contains data for both sexes combined, the age-specific cases or deaths are split and reassigned to separate sexes using the same weights that are used for the age-splitting process. Starting from the expected number of deaths, global proportions are generated by sex for each age. For example, if for ages 15–19 years old there are 6 expected deaths for males from cause of death data and 4 expected deaths for females, then 60% of the combined-sex deaths for ages 15–19 years would be assigned to males and the remaining 40% would be assigned to females.

5. Cause disaggregation. In the fifth step (#5 in eFigure 1), data for cause entries that are aggregates of GBD causes were redistributed across those GBD causes. Examples of these aggregated causes include some cancer registries reporting ICD-10 codes C00-C14 together as "lip, oral cavity, and pharyngeal cancer." These groups are broken down into subcauses that can be individually mapped to single GBD causes. In this example, the more specific ICD-10 codes within C00–C14 are "lip and oral cavity cancer" (C00–C08), "nasopharynx cancer" (C11), "cancer of other parts of the pharynx" (C09–C10, C12–C13), and "Malignant neoplasm of other and ill-defined sites in the lip, oral cavity, and pharynx" (C14). To redistribute the data, weights were created using the same "rate-applied-to-population" method employed in age-sex splitting (see step four above). For the undefined code (C14 in the example) an "average all cancer" weight was used, calculated on the high-quality cancer registry data from SEER[8]/NORDCAN[20]/CI5[9–19] by dividing the sum of the cases across these registries by the combined population across these registries. Then, proportions were generated by subcause for each aggregate cause as in the sex-splitting example above (see step four). The total number of cases from the aggregated group (C00–C14) was recalculated for each subgroup and the undefined code (C14). C14 was then redistributed as a "garbage code" in step six.

*6. Redistribution***.** In the sixth step (#6 in eFigure 1), unspecified ICD codes ("garbage codes") such as "ill-defined cancer site" (for example, C76 or C80) are redistributed across relevant causes estimated within the GBD hierarchy. Redistribution of cancer registry incidence and mortality data mirrored the process of the redistribution used in the cause of death database and utilized the same redistribution maps as specified in Section 2.4 of the Supplementary Appendix 1 to the GBD 2019 Diseases and Injuries capstone, "Global burden of 369 diseases and injuries in 204 countries and territories, 1990–2019: a systematic analysis for the Global Burden of Disease Study 2019."[4] Sources and targets of garbage codes can be found in eTable 6 of the Supplementary Appendix to "Cancer Incidence, Mortality, Years of Life Lost, Years Lived with Disability, and Disability-Adjusted Life Years for 29 Cancer Groups from 2010 to 2019: A Systematic Analysis of Cancer Burden Globally, Nationally, and by Socio-demographic Index for the Global Burden of Disease Study 2019."[2]

7. Removal of duplicates. In the seventh step (#7 in eFigure 1), duplicate or redundant data sources were removed from the processed cancer registry dataset. Duplicate sources were present if, for example, a cancer registry was part of the CI5 database but we also had data from that registry directly. Redundancies occurred and were removed as described in "Cancer Incidence Data Sources," where more detailed data were available, or when national registry data could replace regionally representative data. From here, two parallel selection processes were run; one to generate input data for the mortality-to-incidence ratio (MIR) models, and one to generate incidence for final mortality estimation. When creating the final incidence input, higher priority was given to registry data from the most standardized source; whereas for the MIR model input, only sources that reported both incidence and mortality were used.

8. Combine matching incidence and mortality data and model MIRs. In the eighth step (#8 in eFigure 1), the processed incidence and mortality data from cancer registries were matched by cancer cause, age, sex, year, and location to generate MIRs. The resulting MIRs were used as input for a three-step modeling approach using the general GBD spatiotemporal Gaussian process regression (ST-GPR[5]) approach, with the Healthcare Access and Quality (HAQ) Index as a covariate in the linear mixed effects model using logit transformed MIR as outcome.[22]

$$\text{logit}\left(MIR_{c,a,s,t}\right) = \alpha + \beta_1(HAQIndex_{c,t}) + \sum_{a}^{A} \beta_2 I_a + \beta_3 I_s + \epsilon_{c,a,s,t}$$

MIR: mortality-to-incidence ratio
c: country (or subnational for subnationally modeled locations), a: age group, t: time (years); s: sex
HAQ Index: Healthcare Access and Quality Index
I: indicator variable
$\epsilon_{c,a,s,t}$: error term

Information on ST-GPR can be found in "Section 4.3.3: Spatiotemporal Gaussian process regression (ST-GPR) modeling" in Supplementary Appendix 1 to "Global burden of 369 diseases and injuries in 204 countries and territories, 1990–2019: a systematic analysis for the global burden of disease study 2019."[4] Predictions were made without the random effects. The ST-GPR model has three main hyper-parameters that control for smoothing across time, age, and geography.[4] These hyper-parameters were adjusted for GBD 2019 in order to improve model performance in locations with sparse data. The time adjustment parameter lambda (λ) aims to borrow strength from neighboring time points (i.e., the value in this year is highly correlated with the value in the previous year but less so further back in time). For GBD 2019, lambda was lowered from 2 to 0.05, increasing the weight of more distant years. The age adjustment parameter omega (ω) borrows strength from data in neighboring age groups and was lowered from 1.0 to

0.5, increasing the weight of more distant age groups. The space adjustment parameter zeta (ξ) aims to borrow strength across the hierarchy of geographical locations. Zeta was lowered from 0.95 to 0.01, reducing the weight of more distant geographical data at the region or super region level. For the remaining parameters in the Gaussian process regression, we lowered the amplitude from 2 to 1 (reducing fluctuation from the mean function) and reduced the scale value from 15 to 10 (reducing the time distance over which points are correlated).

Data-cleaning steps for MIR estimation were similar to those for GBD 2017. For each cancer, MIRs from locations in HAQ Index quintiles 1–4 were dropped if they were below the median of MIRs from locations in HAQ Index quintile 5. We also dropped MIRs from locations in HAQ Index quintiles 1–4 if the MIRs were above an outlier threshold calculated as the third quartile + 1.5 * IQR (inter-quartile range). We dropped all MIR data that were based on fewer than 15 incident cases to avoid excessive variation in the ratio due to small numbers (this threshold was 25 cases in GBD 2017, but was lowered in GBD 2019 in order to include additional data). For the lower end of the age spectrum where cancers are generally rarer, we also aggregated incidence and mortality to the youngest five-year age bin where SEER[8] reported at least 50 cases from 1990 to 2015, to avoid unstable MIR predictions in young age groups because of too few data. The MIR estimates in this SEER-based minimum age-bin were then copied down to all younger GBD age groups estimated for that cancer.

Since MIRs can be above 1, especially in older age groups and cancers with low cure rates, we used the 95th percentile (by age group) of the cleaned dataset (detailed above) to cap the MIR input data. These "upper cap" values were used to allow MIRs over 1 in some age groups but to constrain the MIRs to a maximum level. Any MIR values over this cap were Winsorized to the cap value. To run the logit model, the input data were first divided by the upper caps to get proportional data ranging from 0 to 1. Model predictions from ST-GPR were then rescaled back by multiplying them by the upper caps. To constrain the MIRs at the lower end, we used the fifth percentile of the cancer and age-specific cleaned MIR input data to Winsorize all model predictions below this lower cap.

9. Generate mortality estimates from incidence and MIRs. Final estimated MIRs were matched with the cleaned cancer registry incidence dataset finalized in the ninth step (#9 in eFigure 1) to generate mortality estimates (#10 in eFigure 1):

$$MIR_{estimates} * incidence_{registry} = mortality_{CR\ inputs}$$

These mortality estimates were then smoothed by a Bayesian noise-reduction algorithm (to deal with zero counts; this is also applied to the VR and VA data), as specified in Section 2.14 of the Supplementary Appendix 1 to the GBD 2019 paper "Global burden of 369 diseases and injuries in 204 countries and territories, 1990–2019: a systematic analysis for the Global Burden of Disease Study 2019."[4] These data were uploaded into the CoD database as CR data (#11 in eFigure 1). Cancer-specific mortality modeling then followed the general CODEm process[23] using the totality of VA, VR, and CR data.

4.2 Causes of death (CoD) data processing

Formatting of data sources for the cause of death (CoD) database, including VR and VA data, is similar to many of the steps outlined above for CR data (#11 in eFigure 1) and is described in Section 2 of the Supplementary Appendix 1 to the GBD 2019 paper "Global burden of 369 diseases and injuries in 204 countries and territories, 1990–2019: a systematic analysis for the Global Burden of Disease Study 2019."[4]

VA data may not capture cancer deaths as accurately or comprehensively as cancer registries or vital registration systems, but provides a useful contribution to cancer models in locations without VR or CR data. Additional processing and restrictions are performed on VA to ensure quality standards and feasible inputs. More details on VA data processing are provided in the appendix noted above, particularly Sections 2.2 (VA overview), 2.10 (VA cause restrictions), 2.14 (noise reduction), 2.15 (outlier identification), and 2.16 (data quality ratings).

5. CODEm overview

Cause of death ensemble modeling (CODEm) is the framework used to model most cause-specific death rates in the GBD. It relies on four key components:

1. All available data are identified and gathered to be used in the modeling process. Although the data may vary in quality, they all contain some signal of the true epidemiological process.
2. A diverse set of plausible models are developed to capture well-documented associations in the estimates. Using a wide variety of individual models to create an ensemble predictive model has been shown to outperform techniques using only a single model both in CoD estimation and in more general prediction applications.
3. The out-of-sample predictive validity is assessed for all individual models, which are then ranked for use in the ensemble modeling stage.
4. Differently weighted combinations of individual models are evaluated to select the ensemble model with the highest out-of-sample predictive validity.

Separate models are run for different age ranges, when applicable. Restrictions were applied on age by each cancer type in GBD 2019 according to eTable 2.

eTable 2. Age restrictions for LOC and OPC in GBD 2019 modeling

Cause	Minimum Age	Maximum Age
Lip and oral cavity cancer	5	None
Other pharynx cancer	20	None

Additionally, separate models are developed for countries with extensive, complete, and representative VR for every cause to ensure that uncertainty can better reflect the more complete data in these locations.

To ensure the addition of subnational locations are not driving changes in estimates, in GBD 2019, we run a global model that excludes data from non-standard locations; the resulting covariate betas are then used as priors for the true global model.

5.1. Model pool development

Because many factors may co-vary with any given CoD, a range of plausible statistical models are developed for each cause. In the CODEm framework, four families of statistical models are used: linear mixed effects regression (LMER) models of the natural log of the cause-specific death rate, LMER models of the logit of the cause fraction, spatiotemporal Gaussian process regression (ST-GPR) models of the natural logarithm of the cause-specific death rate, and ST-GPR models of the logit of the cause fraction. For each family of models, all plausible relationships between covariates and the response variable are identified. Because all possible combinations of selected covariates are considered for each family of models, multi-collinearity between covariates may produce implausible signs on coefficients or unstable coefficients. Each combination is therefore tested for statistical significance (covariate coefficients must have a coefficient with p-value < 0.05) and plausibility (the coefficients must have the directions expected based on the literature). Only covariate combinations meeting these criteria are retained. This selection process is run for both cause fractions and death rates, then ST-GPR and LMER-only models are created for each set of covariates.

5.2. Data variance estimation

The families of models that go through ST-GPR incorporate information about data variance. The main inputs for a Gaussian process regression (GPR) are a mean function, a covariance function, and data variance for each data point. For GBD 2019, we have updated this calculation to incorporate garbage code redistribution uncertainty.

Three components of data variance are now used in CODEm: sampling variance, non-sampling variance, and garbage code redistribution variance. The computation of sampling variance and nonsampling variance has not changed since previous iterations of the GBD. Garbage code redistribution variance is computed in the CoD database process described in Section 2.7 (page 31) of the Appendix 1 of "Global burden of 369 diseases and injuries in 204 countries and territories, 1990–2019: a systematic analysis for the Global Burden of Disease Study 2019."[4] Since variance is additive, we calculate total data variance as the sum of sampling variance, non-sampling variance, and redistribution variance. Increased data variance in GPR results in the GPR draws not following the data point as closely.

5.3. Testing model pool on 15% sample

The performance of all models (individual and ensemble) is evaluated by means of out-of-sample predictive validity tests. Thirty percent of the data are randomly excluded from the initial model fits. These individual model fits are evaluated and ranked by using half of the excluded data (15% of the total), then used to construct the ensembles on the basis of their performance. Data are held out from the analysis on the basis of the cause-specific missingness patterns for ages and years across locations. Out-of-sample predictive validity testing is repeated 20 times for each model, which has been shown to produce stable results. These performance tests include the root mean square error (RMSE) for the log of the cause-specific death rate, the direction of the predicted versus actual trend in the data, and the coverage of the predicted 95% UI.

5.4. Ensemble development and testing

The component models are weighted on the basis of their predictive validity rank to determine their contribution to the ensemble estimate. The relative weights are determined both by the model ranks and by a parameter ψ, whose value determines how quickly the weights taper off as rank decreases. The distribution of ψ is described in more detail in Foreman et al.[23] A set of ensemble models is then created by using the weights constructed from the combinations of ranks and ψ values. These ensembles are tested by using the predictive validity metrics described in Section "Testing model pool on 15% sample" on the remaining 15% of the data, and the ensemble with the best performance in out-of-sample trend and RMSE is chosen as the final model.

5.5. Final estimation

Once a weighting scheme has been chosen, 1000 draws are created for the final ensemble, and the number of draws contributed by each model is proportional to its weight. The mean of the draws is used as the final estimate for the CODEm process, and a 95% UI is created from the 0.025 and 0.975 quantiles of the draws. The validity of the UI can be checked via its coverage of

the out-of-sample data; ideally, the 95% UI would capture 95% of these data. Higher coverage suggests that the UIs are too large, and lower coverage suggests overfitting.

5.6. Model-specific covariates

Modelers select covariates to be used in CODEm, but those covariates may not be significant or in the direction specified during the covariate selection step of CODEm and will therefore not be used in the model. Additionally, covariates may be selected by CODEm but only exist in submodels that perform poorly and may end up with zero draws included in the final ensemble. CODEm covariates used, level of covariate, and expected direction of covariate by cause, sex, and age for the cancers relevant to this analysis can be found in eTables 3 & 4 below; a comprehensive list for all causes of death modeled in the GBD 2019 study can be found in Table S16, page 1570, in Appendix 1 of "Global burden of 369 diseases and injuries in 204 countries and territories, 1990–2019: a systematic analysis for the Global Burden of Disease Study 2019."[4]

eTable 3. Covariates for lip and oral cavity cancer

Level	Covariate	Direction
1	Alcohol (liters consumed per capita)	+
	Cumulative cigarettes (10 years)	+
	Cumulative cigarettes (20 years)	+
	Tobacco (cigarettes per capita)	+
	Log-transformed summary exposure value (SEV) scalar for LOC	+
2	Age- and sex-specific SEV for high red meat	+
	Age- and sex-specific SEV for low vegetables	+
	Age- and sex-specific SEV for low fruits	+
	Healthcare access and quality index	-
3	Education (years per capita)	-
	Lag-distributed income (I$ per capita)	+
	Socio-demographic Index	+

Lag-distributed income per capita (I$): gross domestic product per capita that has been smoothed over the preceding 10 years;
Summary exposure value (SEV): for definitions and calculations, please see Section 2.6: "Step 5. Estimate summary exposure values" in the Supplementary Appendix 1 to "Global burden of 87 risk factors in 204 countries and territories, 1990–2019: a systematic analysis for the Global Burden of Disease Study 2019"[5]; covariates with "C" following a cancer site name refer to a cancer site (e.g., uterus C = uterus cancer) and were shortened due to space limitations in covariate names.

eTable 4. Covariates for other pharynx cancer

Level	Covariate	Direction
1	Alcohol (liters consumed per capita)	+
	Smoking prevalence	+
	Log-transformed summary exposure value (SEV) scalar for other pharynx cancer	+
2	Cumulative cigarettes (5 years)	+
	Age- and sex-specific SEV for low fruits	+
	Age- and sex-specific SEV for low vegetables	+
	Population density (over 1000 ppl/sqkm, proportion)	+
	Population density (under 150 ppl/sqkm, proportion)	+
	Healthcare access and quality index	-
3	Education (years per capita)	-
	Lag-distributed income (I$ per capita)	+
	Socio-demographic Index	+

Lag-distributed income per capita (I$): gross domestic product per capita that has been smoothed over the preceding 10 years;
Summary exposure value (SEV): for definitions and calculations, please see Section 2.6: "Step 5. Estimate summary exposure values" in the Supplementary Appendix 1 to "Global burden of 87 risk factors in 204 countries and territories, 1990–2019: a systematic analysis for the Global Burden of Disease Study 2019"[5]; covariates with "C" following a cancer site name refer to a cancer site (e.g., uterus C = uterus cancer) and were shortened due to space limitations in covariate names.

6. CoDCorrect

CODEm models estimate the individual cause-level mortality without taking into account the independently modeled all-cause mortality (#13 in eFigure 1). To ensure that all single causes add up to the all-cause mortality and that all child-causes add up to the parent cause, an algorithm called "CoDCorrect" is used (#14 and #15 in eFigure 1). Further details on the CoDCorrect algorithm can be found in Section 3.3.2 of the Supplementary Appendix 1 to the GBD 2019 paper "Global burden of 369 diseases and injuries in 204 countries and territories, 1990–2019: a systematic analysis for the Global Burden of Disease Study 2019."[4] Final mortality estimates at the 1000-draw level provide an estimated mean mortality with 95% uncertainty interval.

7. Years of life lost calculation

To calculate years of life lost (YLLs), final death estimates after CoDCorrect adjustment are multiplied by the standard GBD life expectancy given the age at death, sex, and location. Further details on GBD life expectancy values can be found in the GBD 2019 paper "Global age-sex-specific fertility, mortality, health life expectancy (HALE), and population estimates in 204 countries and territories, 1950–2019: a comprehensive demographic analysis for the Global

Burden of Disease Study 2019."[24] Uncertainty is propagated from the CoDCorrect mortality estimates, calculating YLLs for each of the 1000 CoDCorrect draws to provide estimated mean YLLs with corresponding 95% uncertainty intervals.

8. Non-fatal cause-specific modeling descriptions

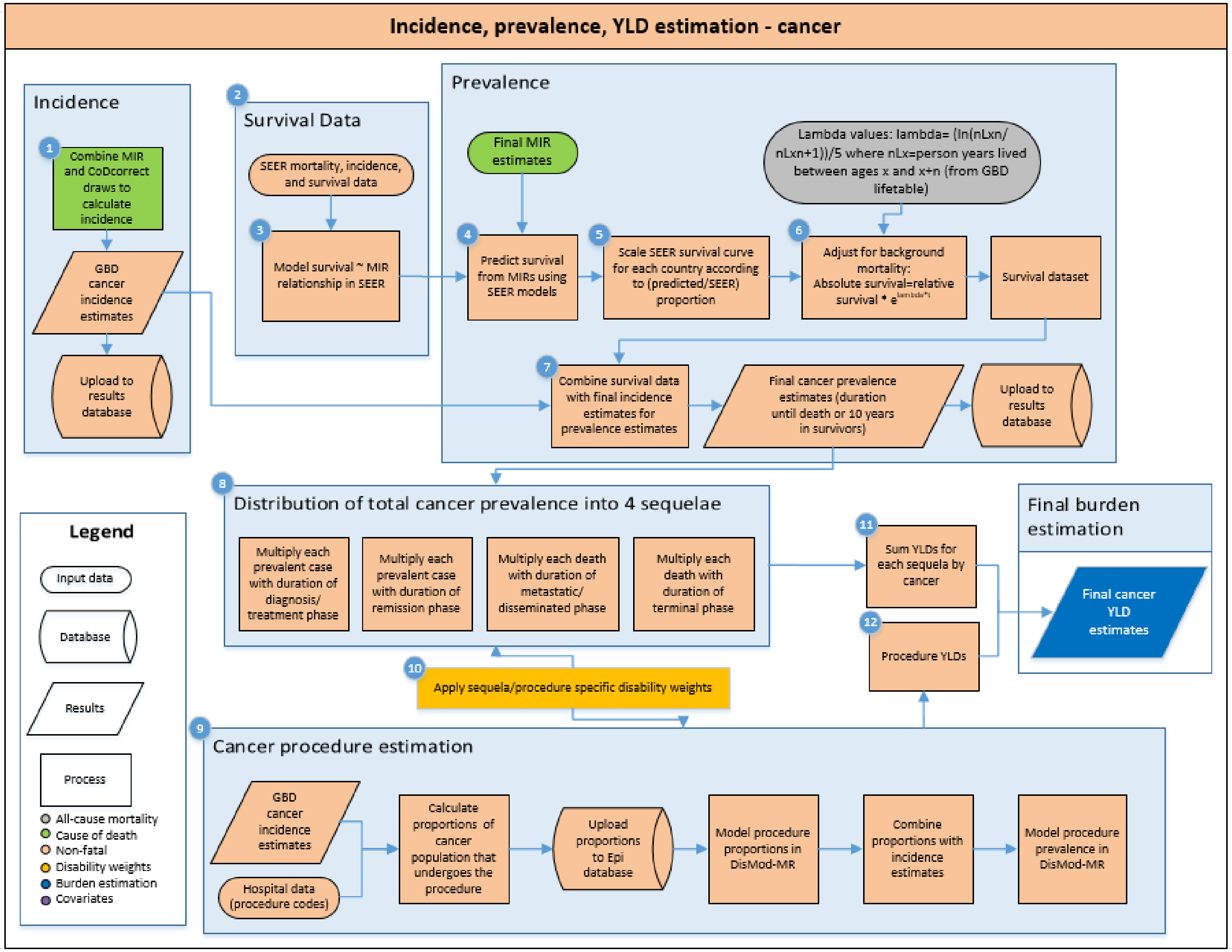

eFigure 2. Flowchart of GBD cancer incidence and Years Lived with Disability (YLDs) estimation, including LOC and OPC.
Abbreviations: GBD, Global Burden of Disease Study; MIR, mortality-to-incidence ratio; SEER, Surveillance, Epidemiology and End Results Program; YLD, years lived with disability.

8.1 Incidence estimation

The final GBD cancer mortality estimates (after CoDCorrect adjustment) were transformed to incidence estimates by using the MIRs specific to that cancer cause (#1 in eFigure 2). Final mortality estimates at the 1000-draw level were divided by the modeled MIR estimates (also at the 1000-draw level) to generate 1000 draws of incidence estimates (which provides an estimated mean incidence with 95% uncertainty interval). It was assumed that uncertainty in the MIRs is independent of uncertainty in the estimated mortality.

8.2 Prevalence estimation

After transforming the final GBD cancer mortality estimates to incidence estimates (step 1 in eFigure 2), incidence was combined with annual relative survival estimates from 1 to 10 years after diagnosis (step 7 in eFigure 2). Previous reports suggest that the value of (1 – MIR) may serve as a proxy for 5-year relative survival, with the exact correlation varying slightly by cancer type.[25] Because this correlation varies, we trained cancer-specific prediction models to estimate 5-year survival from MIRs, using data from SEER.[8] We used SEER*Stat[26] to obtain mortality, incidence, and relative survival statistics from the nine SEER registries reporting from 1980–2014 (step 2), by cancer type, sex, 5-year blocks (i.e., 1980–84, 1985–1989, etc.), and 5-year age groups (except combining 80+). For each cancer, we modelled SEER 5-year relative survival using MIRs calculated from SEER mortality and incidence. For GBD 2019 we updated this model from the Poisson regression used in GBD 2017[27] to using a generalized linear model with a quasibinomial family and logit link, weighted by the number of index cases (step 3 in eFigure 2). To reduce variability due to small samples, we only included MIRs based on at least 25 incident cases (except for the cancers mesothelioma, nasopharynx cancer, and acute lymphoid leukemia, where MIRs based on at least 10 cases were included). These models were then applied to the GBD MIR estimates to predict an estimated 5-year survival for each age/sex/year/location (step 4). To prevent unrealistic values, predicted 5-year survival values were Winsorized to be between 0% and 100% survival.

To generate yearly survival estimates up to 10 years, we downloaded SEER[8] sex- and age-specific annual 1- through 10-year relative survival data from persons diagnosed between 2001 and 2010 (2001 through 2010 so that all cases had at least 5 years of follow-up, with half having the full 10 years of follow-up). This is updated from GBD 2017, where we downloaded all-ages survival data from persons diagnosed in 2004 (2004 so that all cases had the full 10 years of follow-up[28]). A proportional scalar was calculated as the predicted GBD 5-year survival estimate divided by the SEER 5-year survival statistic, and was then used to generate yearly survival estimates by scaling the 1–10 year SEER curve to the GBD survival predictions under the proportional hazard assumption (step 5).

The estimated relative survival is next transformed into absolute survival estimates (steps 6 and 7 in eFigure 2). To account for background mortality in the relative survival estimates, GBD 2019 lifetables were used to calculate lambda (λ) values[24]:

$$\lambda = \frac{\ln\left(\frac{nLx_n}{nLx_{n+1}}\right)}{5}$$

nLx = person-years lived between ages x and x+n (from GBD lifetable).

Absolute survival was then calculated using an exponential survival function:

$$absolute\ survival = relative\ survival * e^{\lambda * t}$$

t = time (in years)

Absolute survival is combined with incidence to estimate the prevalence at each year after diagnosis, which is then split into the four sequelae (step 8 in eFigure 2). For the purposes of calculating disability due to cancer, survivors beyond 10 years were considered cured.

For this group, the survivor population prevalence was divided into two sequelae (1. diagnosis and primary therapy; 2. controlled phase). For the population that did not survive beyond 10 years, the yearly prevalence was divided into the four sequelae by assigning the fixed durations for each of the diagnosis and primary therapy phase, metastatic phase, and terminal phase, and assigning the remaining prevalence to the controlled phase (step 8 in eFigure 2). eTable 5 lists the duration of each, along with the sources used to determine their length.

eTable 5. Duration (months) of each sequalae for LOC and OPC

Phase	Lip and oral cavity cancer*	Other pharynx cancer*
(1) Diagnosis and primary treatment	5.3[29]	5.3[29]
(2) Controlled	Calculated based on the remainder of time after attributing other sequelae	Calculated based on the remainder of time after attributing other sequelae
(3) Metastatic	9.3[30]	7.9[30]
(4) Terminal	1	1

* Superscripts refer to references used to inform these values.

Lastly, the general sequelae prevalence were multiplied with their respective disability weights (eTable 6) to obtain the number of YLDs (steps 11 and 12 in eFigure 2). In brief, disability weights are created from survey data to represent the magnitude of health loss associated with an outcome. These disability weights range from 0, implying a state equivalent to full health, to 1, a state equivalent to death. The sum of these YLDs is the final YLD estimate associated with each cancer.

eTable 6. Lay description of cancer states and corresponding disability weights

**Health state**	**Lay description**	**Disability weight (95% uncertainty interval)**
Cancer, diagnosis and primary therapy *All cancers*	This person has pain, nausea, fatigue, weight loss and high anxiety.	0.288 (0.193 to 0.399)
Cancer, controlled phase *All cancers*	This person has a chronic disease that requires medication every day and causes some worry but minimal interference with daily activities.	0.049 (0.031 to 0.072)
Cancer, metastatic *All cancers*	This person has severe pain, extreme fatigue, weight loss and high anxiety.	0.451 (0.307 to 0.600)
Terminal phase, with medication *All cancers*	This person has lost a lot of weight and regularly uses strong medication to avoid constant pain. The person has no appetite, feels nauseous, and needs to spend most of the day in bed.	0.540 (0.377 to 0.687)

9. Estimation process for DALYs

To estimate DALYs for GBD 2019, we started by estimating cause-specific mortality and non-fatal health loss. For each year for which YLDs have been estimated, we computed DALYs by adding YLLs and YLDs for each age-sex-location. Uncertainty in YLLs was assumed to be independent of uncertainty in YLDs. We calculated 1000 draws for DALYs by summing the first draw of the 1000 draws for YLLs and YLDs and then repeating for each subsequent draw. 95% UIs were computed by using the 25th and 975th ordered draw of the DALY uncertainty distribution. We calculated DALYs as the sum of YLLs and YLDs for each cause, location, age group, sex, and year.

10. Reporting Standards

The GBD world population age standard was used to calculate age-standardized rates presented throughout GBD. In GBD 2019, we used the non-weighted mean of the GBD year's age-specific proportional distributions for national locations with populations greater than 5 million in the GBD year to update the world population age standard.[24] The final values used for the age standard are specified in Appendix Table 13 of the GBD 2019 paper "Global age-sex-specific fertility, mortality, health life expectancy (HALE), and population estimates in 204 countries and territories, 1950–2019: a comprehensive demographic analysis for the Global Burden of Disease Study 2019."[24]

11. Socio-demographic Index (SDI) definition and calculation

Socio-demographic Index (SDI) is a summary indicator to represent background levels of social and economic conditions that can influence health outcomes in a given location. This summary indicator comprises three indices: lag-distributed income per capita, mean education for those aged 15 years or older, and total fertility rate for those younger than 25 years of age. Possible values for each of these three indices range from 0 to 1, representing the bounds with which lower or higher values of the level of development for that index would no longer worsen or improve health outcomes, respectively. The composite SDI is the geometric mean of these three indices for a given location-year. For reporting purposes, values were multiplied by 100 to obtain SDI on a scale of 0 to 100. The SDI cutoffs for determining SDI quintiles for analysis were computed by using the country-level estimates of SDI for the year 2019, excluding countries with populations less than 1 million. For GBD 2019 analyses, all locations are assigned to these quintiles according to their SDI value in the year 2019. See Section 6 in Supplementary Appendix 1 to the GBD 2019 Diseases & Injuries capstone[4] for more details regarding SDI estimation, and page 54 of this Appendix for the SDI quintile estimate for each country or territory in the GBD 2019 study.

12. Uncertainty estimation

Uncertainty in cancer estimates begins with the availability of and variability in cancer cause-specific data by age, sex, location and year. The uncertainty in cancer mortality estimates arises from CODEm and CoDCorrect. For more information see the CODEm methodology paper by Foreman et al., and Supplementary Appendix 1 to "Global burden of 369 diseases and injuries in 204 countries and territories, 1990–2019: a systematic analysis for the global burden of disease study 2019."[4,23] Uncertainty in cancer incidence estimates results from both the uncertainty in mortality estimates as well as the uncertainty in the

MIR estimates, which result from the ST-GPR models. Uncertainty from the mortality estimates and the MIRs were assumed to be independent. Cancer prevalence uncertainty results from both the incidence uncertainty as well as the uncertainty from survival estimates. These were assumed to be independent. Uncertainty in cancer YLD estimation results from the uncertainty in the prevalence of each cancer sequela and uncertainty in the disability weight and is propagated into the final comorbidity-corrected YLD result. The uncertainty in prevalence and the uncertainty in disability weights are assumed to have no correlation. Cancer YLL uncertainty results from uncertainty in mortality estimates as well as uncertainty in life expectancy estimates. Uncertainty in cancer DALY estimates results from the uncertainty in YLLs and the uncertainty in YLDs, which were assumed to be independent. The same technique for propagating uncertainty elsewhere in the GBD study is applied in the cancer estimation process. In brief, the distribution of each step in the computation process is stored in 1000 draws. The distributions are determined from the data input sampling error, the uncertainty of the model coefficients, and the uncertainty of severity distributions and disability weights. The 1000 draws are used for every step in the process, with final estimates computed using the mean estimate across 1000 draws. The 95% uncertainty intervals are determined by the 25th and 975th ranked values across all 1000 draws.[4] More specific information regarding uncertainty intervals can be found in the GBD 2019 capstone papers.[4,5,24]

13. Limitations

There are certain limitations to consider when interpreting the GBD mortality cancer estimates. First, even though every effort is made to include the most recently available data for each country, data seeking resources are not limitless and new data cannot always be accessed as soon as they are made available. It is therefore possible that the GBD study does not include all available data sources for cancer incidence or cancer mortality. Second, different redistribution methods can potentially change the cancer estimates substantially if the data sources used for the estimated location contain a large number of undefined causes; however, neglecting to account for these undefined deaths would likely introduce an even greater bias in the disease estimates. Third, using mortality-to-incidence ratios to transform cancer registry incidence data to mortality estimates requires accurate MIRs. For GBD 2019 we have made further refinements to the estimation of MIRs, but the method remains sensitive to under-diagnosis of cancer cases or under-ascertainment of cancer deaths. However, given that the majority of data used for the cancer mortality estimation come from vital registration data and not cancer registry data, this is not a major limitation. Finally, no estimates are available for some locations, such as Western Sahara and French Guiana, as they were not modelled locations in the Global Burden of Diseases, Injuries, and Risk Factors Study 2019. These areas are shaded white in the global map figures included in this paper.

Part II – Risk Factors Analysis

1. Overview and definition of indicator

This study presents the proportion of mortality and DALYs from lip and oral cancer (LOC) attributed to three risk factors: smoking, chewing tobacco, and alcohol consumption; it also presented the proportion of mortality and DALYs from other pharynx cancer (OPC) attributed to two risk factors: smoking and alcohol consumption. These data were produced by the GBD 2019 study. Definitions related to risk factors are described in the sections dedicated to each risk factor, later in this appendix.

2. Data input sources overview

GBD 2019 incorporated a large number and wide variety of input sources to estimate mortality, causes of death and illness, and risk factors for 204 countries and territories from 1990–2019. These input sources are accessible through an interactive citation tool available in the GHDx: http://ghdx.healthdata.org/gbd-2019/data-inputsources.

3. Risk factor estimation

The comparative risk assessment (CRA) conceptual framework was developed by Murray and Lopez[31], who established a causal web of hierarchically organized risks or causes that contribute to health outcomes, which allows for quantification of risks or causes at any level in the framework. In GBD 2019, as in previous iterations of the GBD study, we evaluated a set of behavioral, environmental and occupational, and metabolic risks, in which risk-outcome pairs were included based on evidence rules. These risks were organized in four hierarchical Levels, where:

- **Level 1** represents the overarching categories (behavioral, environmental and occupational, and metabolic) nested within Level 1 risks;
- **Level 2** contains both single risks and risk clusters
- **Level 3** contains the disaggregated single risks from within Level 2 risk clusters
- and **Level 4** details risks with the most granular disaggregation,

All risk factors analyzed in the GBD 2019 Study, and their respective hierarchy level, are listed in Table S2 (page 303) of Appendix 1 of “Global burden of 87 risk factors in 204 countries and territories, 1990–

2019: a systematic analysis for the Global Burden of Disease Study 2019."[5] At each level of risk, we evaluated whether risk combinations were additive, multiplicative, or shared common pathways for intervention. This approach allows the quantification of the proportion of risk-attributable burden shared with another risk or combination of risks and the measurement of potential overlaps between behavioral, environmental and occupational, and metabolic risks. We do provide some insights into the potential magnitude of distal social, cultural, and economic factors through an analysis of the relationship between risk exposures and development measured by using the Socio-demographic Index (SDI).

Two types of risk assessments are possible within the CRA framework: attributable burden and avoidable burden. Attributable burden is the reduction in current disease burden that would have been possible if past population exposure had shifted to an alternative or counterfactual distribution of risk exposure. Avoidable burden is the potential reduction in future disease burden that could be achieved by changing the current distribution of exposure to a counterfactual distribution of exposure. Murray and Lopez identified four types of counterfactual exposure distributions: (1) theoretical minimum risk; (2) plausible minimum risk; (3) feasible minimum risk; and (4) cost-effective minimum risk.[32] The TMREL is the level of risk exposure that minimizes risk at the population level or the level of risk that captures the maximum attributable burden. Other possible forms of risk quantification include plausible minimum risk – which reflects the distribution of risk that is conceivably possible and would minimize population-level risk if achieved – whereas feasible minimum risk describes the lowest risk distribution that has been attained within a population and cost-effective minimum risk is the lowest risk distribution for a population that can be attained in a cost-effective manner. Because no robust set of forecasts for all components of GBD is available, in this study we focus on quantifying attributable burden by using the theoretical minimum risk counterfactual distribution.

A description with a high-level overview of the analytical logic and the sufficient detail on the methods and overall structure of the estimation process can be found on Section 2 (starting on page 16) of Appendix 1 of "Global burden of 87 risk factors in 204 countries and territories, 1990–2019: a systematic analysis for the Global Burden of Disease Study 2019."[5] Here we aim to provide a synthesis focused on smoking, chewing tobacco, and alcohol consumption risk factors.

4. GBD risk-specific methods summaries

The following section provides further methodological detail and GBD case or exposure definitions for risks where the estimation process differs from the general GBD risk factors modelling framework described above. These write-ups were copied from "Section 4: Risk-specific modelling descriptions" in the appendix to the GBD 2019 paper, "Global burden of 87 risk factors in 204 countries and territories, 1990–2019: a systematic analysis for the Global Burden of Disease Study 2019".[5]

Modeling risk factors in GBD 2019 often requires disease context across many different communicable and non-communicable diseases; although cancers specifically are the focus of this analysis, the following risk factors methods section will often reference GBD causes (diseases and injuries) that are outside of the scope of this paper. This broader disease context is included for completeness and accuracy.

4.1. Smoking

Flowchart

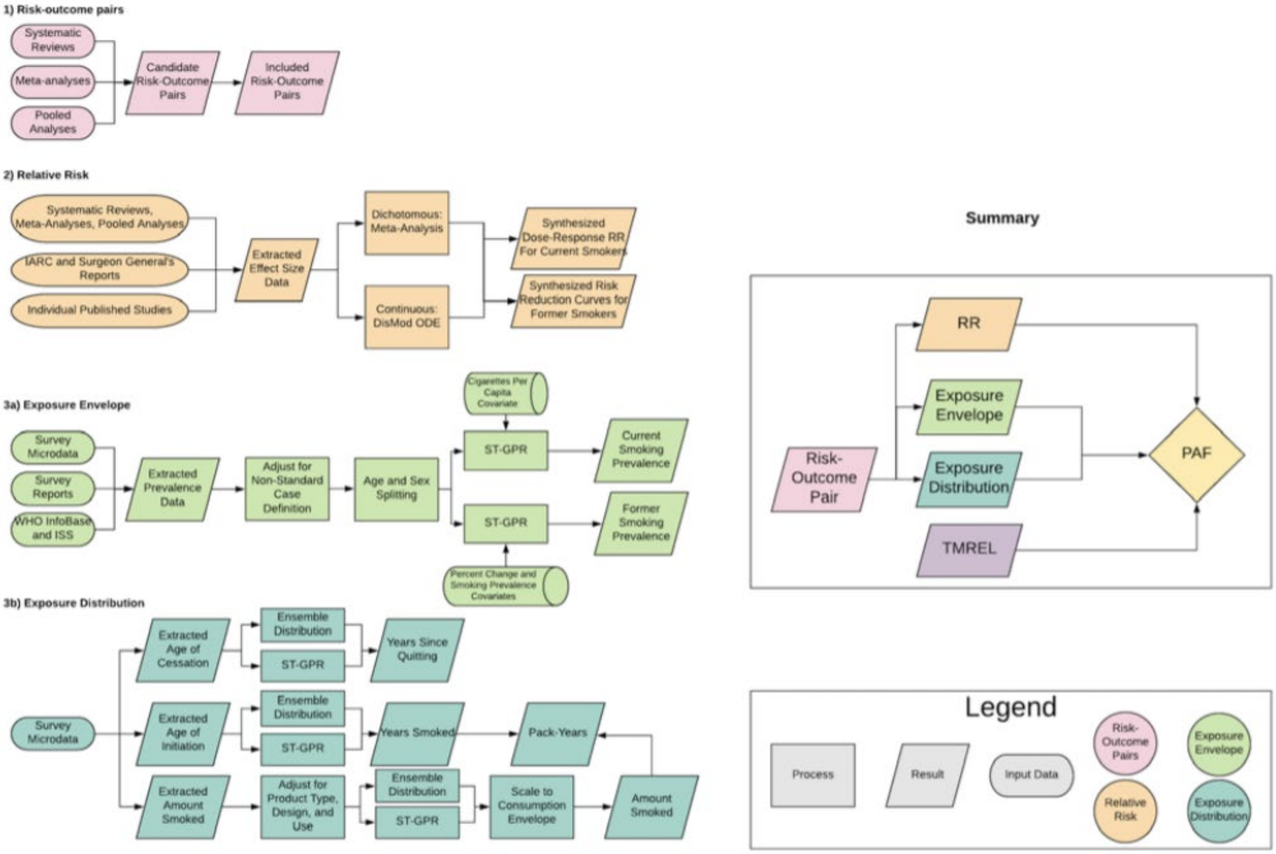

IARC = International Agency for Research on Cancer; RR = relative risk; WHO = World Health Organization; ST-GPR = spaciotemporal Gaussian process regression; PAF = Population attributable fraction; TMREL = Theoretical minimum-risk exposure level.

Input data and methodological summary

Exposure

Case definition

We estimated the prevalence of current smoking and the prevalence of former smoking using data from cross-sectional nationally representative household surveys. We defined current smokers as individuals who currently use any smoked tobacco product on a daily or occasional basis. We defined former smokers as individuals who quit using all smoked tobacco products for at least six months, where possible, or according to the definition used by the survey.

Input data

We extracted primary data from individual level microdata and survey report tabulations. We extracted data on current, former, and/or ever smoked tobacco use reported as any combination of frequency of use (daily,

occasional, and unspecified, which includes both daily and occasional smokers) and type of smoked tobacco used (all smoked tobacco, cigarettes, hookah, and other smoked tobacco products such as cigars or pipes), resulting in 36 possible combinations. Other variants of tobacco products, for example hand-rolled cigarettes, were grouped into the four type categories listed above based on product similarities.

For microdata, we extracted relevant demographic information, including age, sex, location, and year, as well as survey metadata, including survey weights, primary sampling units, and strata. This information allowed us to tabulate individual-level data in the standard GBD five-year age-sex groups and produce accurate estimates of uncertainty. For survey report tabulations, we extracted data at the most granular age-sex group provided.

Crosswalk

Our GBD smoking case definitions were current smoking of any tobacco product and former smoking of any tobacco product. All other data points were adjusted to be consistent with either of these definitions. Some sources contained information on more than one case definition and these sources were used to develop the adjustment coefficient to transform alternative case definitions to the GBD case definition. The adjustment coefficient was the beta value derived from a linear model with one predictor and no intercept. We used the same crosswalk adjustment coefficients as in GBD 2017, and thus we have not included a methods explanation in this appendix, as it has been detailed previously.

Age and sex splitting

As in GBD 2017, we split data reported in broader age groups than the GBD 5-year age groups or as both sexes combined by adapting the method reported in Ng et al. to split using a sex- geography- time specific reference age pattern.[33] We separated the data into two sets: a training dataset, with data already falling into GBD sex-specific 5-year age groups, and a split dataset, which reported data in aggregated age or sex groups. We then used spatiotemporal Gaussian process regression (ST-GPR) to estimate sex-geography-time-specific age patterns using data in the training dataset. The estimated age patterns were used to split each source in the split dataset.

The ST-GPR model used to estimate the age patterns for age-sex splitting used an age weight parameter value that minimizes the effect of any age smoothing. This parameter choice allowed the estimated age pattern to be driven by data, rather than being enforced by any smoothing parameters of the model. Because these age-sex split data points were to be incorporated in the final ST-GPR exposure model, we did not want to doubly enforce a modelled age pattern for a given sex-location-year on a given aggregate data point.

Modelling strategy

Smoking prevalence modelling

We used ST-GPR to model current and former smoking prevalence. The model is nearly identical to that in GBD 2017. Full details on the ST-GPR method can be found in "Section 4.3.3: Spatiotemporal Gaussian process regression (ST-GPR) modeling" in Supplementary Appendix 1 to "Global burden of 369 diseases and injuries in 204 countries and territories, 1990–2019: a systematic analysis for the global burden of disease study 2019."[4] Briefly, the mean function input to GPR is a complete time series of estimates generated from a mixed effects hierarchical linear model plus weighted residuals smoothed across time, space, and age. The linear model formula for current smoking, fit separately by sex using restricted maximum likelihood in R, is:

$$logit(p_{g,a,t}) = \beta_0 + \beta_1 CPC_{g,t} + \sum_{k=2}^{19} \beta_k I_{A[a]} + \alpha_s + \alpha_r + \alpha_g + \epsilon_{g,a,t}$$

where $CPC_{g,t}$ is the tobacco consumption covariate by geography g and time t, described above, $I_{A[a]}$ is a dummy variable indicating specific age group A that the prevalence point $p_{g,a,t}$ captures, and α_s, α_r, and α_g are super-region, region, and geography random intercepts, respectively. Random effects were used in model fitting but not in prediction.

The linear model formula for former smoking is:

$$logit(p_{g,a,t}) = \beta_0 + \beta_1 PctChange_{A[a],g,t} + \beta_3 CSP_{A[a],g,t} + \sum_{k=3}^{20} \beta_k I_{A[a]} + \alpha_s + \alpha_r + \alpha_g + \epsilon_{g,a,t}$$

where $PctChange_{A[a],g,t}$ is the percentage change in current smoking prevalence from the previous year, and $CSP_{A[a],g,t}$ is the current smoking prevalence by specific age group A, geography g, and time t that point $p_{g,a,t}$ captures, both derived from the current smoking ST-GPR model defined above.

Supply-side estimation

The methods for modelling supply-side-level data were changed substantially from those used in GBD 2017. The raw data were domestic supply (USDA Global Surveillance Database and UN FAO) and retail supply (Euromonitor) of tobacco. Domestic supply was calculated as production + imports - exports. The data went through three rounds of outliering. First, they were age-sex split using daily smoking prevalence to generate number of cigarettes per smoker per day for a given location-age-sex-year. If more than 12

points for a particular source-location-year (equal to over 1/3 of the split points) were above the given thresholds, that source-location-year was outliered. A point would not be outliered if it was (in cigarettes per smoker): under five (10–14 year olds); under 20 (males, 15–19 year olds); under 18 (females, 15–19 year olds); under 38/35 and over three (males/females, 20+ year olds). These thresholds were chosen by visualising histograms of the data for each age-sex, as well as with expert knowledge about reasonable consumption levels. In the second round of outliering, the mean tobacco per capita value over a 10-year window was calculated. If a point was over 70% of that mean value away from the mean value, it was outliered. The 70% limit was chosen using histograms of these distances. Additionally, some manual outliering was performed to account for edge cases. Finally, data smoothing was performed by taking a three-year rolling mean over each location-year.

Next, a simple imputation to fill in missing years was performed for all series to remove compositional bias from our final estimates. Since the data from our main sources covered different time periods, by imputing a complete time series for each data series, we reduced the probability that compositional bias of the sources was leading to biased final estimates. To impute the missing years for each series, we modelled the log ratio of each pair of sources as a function of an intercept and nested random effects on super-region, region, and location. The appropriate predicted ratio was multiplied by each source that we did have, and then the predictions were averaged to get the final imputed value. For example, if source A was missing for a particular location-year, but sources B and C were present, then we predicted A twice: once from the modelled ratio of A to B, and again from the modelled ratio of A to C. These two predictions were then averaged. For some locations where there was limited overlap between series, the predicted ratio did not make sense, and a regional ratio was used.

Finally, variance was calculated both across series (within a location-year) as well as across years (within a location-source). Additionally, if a location-year had one imputed point was, the variance was multiplied by 2. If a location-year had two imputed points, the variance was multiplied by 4. The average estimates in each location-year were the input to an ST-GPR model. For this, we used a simple mixed effects model, which was modelled in log space with nested location random effects. Subnational estimates were then further modelled by splitting the country-level estimates using current smoking prevalence.

Theoretical minimum-risk exposure level

The theoretical minimum-risk exposure level is 0.

Exposure among current and former smokers

Identical to GBD 2017, we estimated exposure among current smokers for two continuous indicators: cigarettes per smoker per day and pack-years. Pack-years incorporates aspects of both duration and amount.

One pack-year represents the equivalent of smoking one pack of cigarettes (assuming a 20- cigarette pack) per day for one year. Since the pack-years indicator collapses duration and intensity into a single dimension, one pack-year of exposure can reflect smoking 40 cigarettes per day for six months or smoking 10 cigarettes per day for two years.

To produce these indicators, we simulated individual smoking histories based on distributions of age of initiation and amount smoked. We informed the simulation with cross-sectional survey data capturing these indicators, modelled at the mean level for all locations, years, ages, and sexes using ST-GPR. We rescaled estimates of cigarettes per smoker per day to an envelope of cigarette consumption based on supply-side data. We estimated pack-years of exposure by summing samples from age- and time-specific distributions of cigarettes per smoker for a birth cohort in order to capture both age trends and time trends and avoid the common assumption that the amount someone currently smokes is the amount they have smoked since they began smoking. All distributions were age-, sex-, and region- specific ensemble distributions, which were found to outperform any single distribution.

We estimated exposure among former smokers using years since cessation. We utilized ST-GPR to model mean age of cessation using cross-sectional survey data capturing age of cessation. Using these estimates, we generated ensemble distributions of years since cessation for every location, year, age group, and sex.

Relative risk

The same risk-outcome pairs from GBD 2017 were used: tuberculosis; lower respiratory tract infections; esophageal cancer; stomach cancer; bladder cancer; liver cancer; larynx cancer; tracheal, bronchus, and lung cancer; breast cancer; cervical cancer; colon and rectum cancer; lip and oral cancer; nasopharynx cancer; other pharynx cancer; pancreatic cancer; kidney cancer; leukemia; ischemic heart disease; ischemic stroke; hemorrhagic stroke; subarachnoid hemorrhage; atrial fibrillation and flutter; aortic aneurysm; peripheral artery disease; chronic obstructive pulmonary disease; other chronic respiratory diseases; asthma; peptic ulcer disease; gallbladder and biliary tract diseases; Alzheimer's disease and other dementias; Parkinson disease (protective); multiple sclerosis; diabetes mellitus type 2; rheumatoid arthritis; low back pain; cataract; age-related macular degeneration; and fracture.

Dose-response risk curves

Input data for relative risks were nearly the same as in GBD 2017. The only addition was for chronic obstructive pulmonary disease, for which a few additional studies were included. We synthesized effect sizes by cigarettes per smoker per day, pack-years, and years since quitting from cohort and case-control

studies to produce nonlinear dose-response curves using a Bayesian meta-regression model. For outcomes with significant differences in effect size by sex or age, we produced sex- or age-specific risk curves.

We estimated risk curves of former smokers compared to never smokers taking into account the rate of risk reduction among former smokers seen in the cohort and case-control studies, and the cumulative exposure among former smokers within each age, sex, location, and year group.

Population attributable fraction

As in GBD 2017, we estimated PAFs based on the following equation:

$$PAF = \frac{p(n) + p(f) \int \exp(x) * rr(x) + p(c) \int \exp(y) * rr(y) - 1}{p(n) + p(f) \int \exp(x) * rr(x) + p(c) \int \exp(y) * rr(y)}$$

where $pp(nn)$ is the prevalence of never smokers, $pp(ff)$ is the prevalence of former smokers, $pp(cc)$ is the prevalence of current smokers, $\exp(xx)$ is a distribution of years since quitting among former smokers, $rrrr(xx)$ is the relative risk for years since quitting, $\exp(yy)$ is a distribution of cigarettes per smoker per day or pack-years, and $rrrr(yy)$ is the relative risk for cigarettes per smoker per day or pack-years.

We used pack-years as the exposure definition for cancers and chronic respiratory diseases, and cigarettes per smoker per day for cardiovascular diseases and all other health outcomes.

4.2. Chewing tobacco

Flowchart

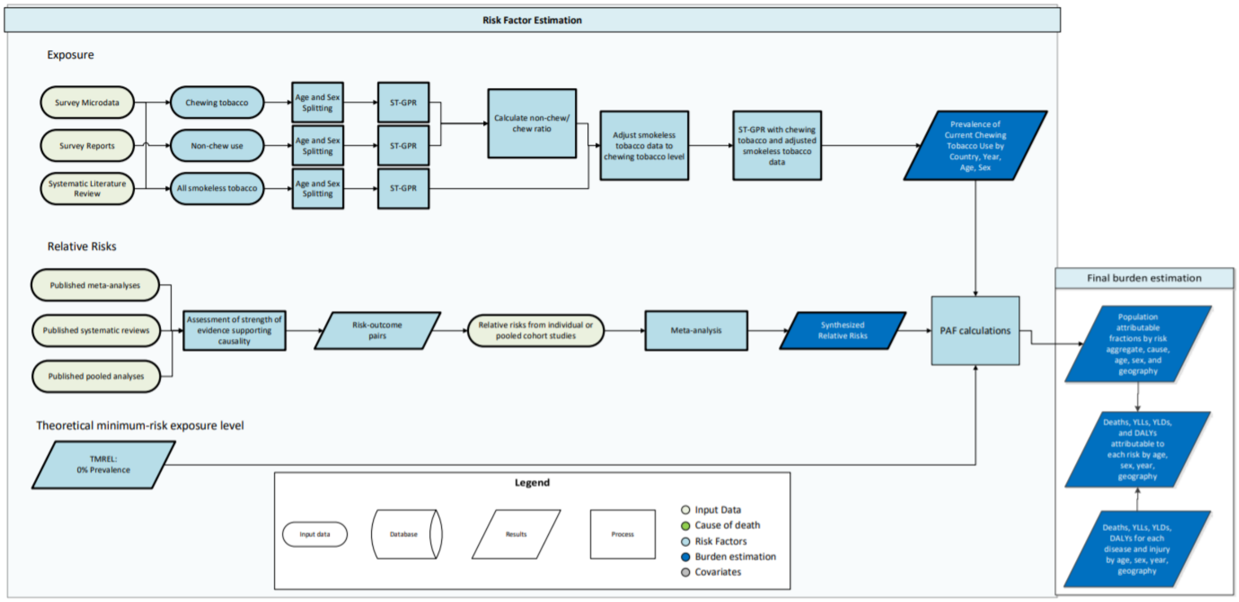

TMREL = Theoretical minimum-risk exposure level; ST-GPR = spaciotemporal Gaussian process regression; PAF = Population attributable fraction; YLL = Years of life lost; YLD = Years lived with disability; DALYs = Disability-adjusted life-years.

Input data and methodological summary

Exposure

Case definition

Current chewing tobacco use is defined as current use (use within the last 30 days where possible, or according to the closest definition available from the survey) of any frequency (any, daily, or less than daily). Chewing tobacco includes local products, such as betel quid with tobacco.

Input data

As in GBD 2017, we included sources that reported primary chewing tobacco, non-chew smokeless tobacco, and all smokeless tobacco use among respondents over age 10. To be eligible for inclusion, sources had to be representative for their level of estimation (i.e., national sources needed to be nationally representative, subnational sources subnationally representative). We included only self-reported use data and excluded data from questions asking about others' tobacco use behaviors.

We extracted primary data from individual-level microdata and survey report tabulations on chewing tobacco, non-chew smokeless tobacco, and all smokeless tobacco use. We extracted data on current,

former, and/or ever use as well as frequency of use (daily, occasional, and unspecified, which includes both daily and occasional smokers). Products that do not include tobacco, such as betel quid without tobacco, were excluded or estimated separately as part of the drug use risk factor, if applicable.

For microdata, we extracted relevant demographic information, including age, sex, location, and year, as well as survey metadata, including survey weights, primary sampling units, and strata. This information allowed us to tabulate individual-level data in the standard GBD five-year age-sex groups and produce accurate estimates of uncertainty. For survey report tabulations, we extracted data at the most granular age-sex group provided.

Age and sex splitting

We split data reported in broader age groups than the GBD five-year age groups or as both sexes combined by adapting the method reported in Ng and colleagues to split using a sex-geography-time-specific reference age pattern.[33] We separated the data into two sets: a training dataset, with data already falling into GBD sex-specific five-year age groups, and a split dataset, which reported data in aggregated age or sex groups. We then used spatiotemporal Gaussian process regression (ST-GPR) to estimate sex-geography-time-specific age patterns using data in the training dataset. The estimated age patterns were then used to split each source in the split dataset.

The ST-GPR model used to estimate the age patterns for age-sex splitting used an age weight parameter value that minimizes the effect of any age smoothing. This parameter choice allows the estimated age pattern to be driven by data, rather than being enforced by any smoothing parameters of the model. Because these age-sex-split datapoints will be incorporated in the final ST-GPR exposure model, we do not want to doubly enforce a modelled age pattern for a given sex-location-year on a given aggregate datapoint. We run three separate ST-GPR models for age-sex splitting – one for each smokeless tobacco category (chew, non-chew, and all smokeless).

Modelling strategy

Prevalence modelling

We used a ST-GPR to model chewing tobacco prevalence. Full details on the ST-GPR method can be found in "Section 4.3.3: Spatiotemporal Gaussian process regression (ST-GPR) modeling" in Supplementary Appendix 1 to "Global burden of 369 diseases and injuries in 204 countries and territories, 1990–2019: a systematic analysis for the global burden of disease study 2019."[4] Briefly, the mean function input to GPR is a complete time series of estimates generated from a mixed effects hierarchical linear model plus

weighted residuals smoothed across time, space, and age. The linear model formula for chewing tobacco, fit separately by sex using restricted maximum likelihood in R, is:

$$logit(p_{g,a,t}) = \beta_0 + \sum_{k=1}^{18} \beta_k I_{A[a]} + \alpha_s + \alpha_r + \alpha_g + \epsilon_{g,a,t}$$

where

> *$I_{A[a]}$ is a dummy variable indicating specific age group A that the prevalence point $p_{g,a,t}$ captures, and α_s, α_r, and α_g are super-region, region, and geography random intercepts, respectively. The hyperparameters are the same as in GBD 2017.*

We run three ST-GPR models for each prevalence category – one for each smokeless tobacco category (chew, non-chew, and all smokeless).

All smokeless tobacco prevalence adjustment

Using the 1000 draws from each of the prevalence ST-GPR models, we calculated 1000 draws of chewing tobacco prevalence divided by the sum of chewing tobacco and non-chewing tobacco prevalence for each location, age group, sex, and year. The draws were unordered, as we did not want to enforce an assumption about the relationship between the levels of chewing tobacco and non-chewing tobacco prevalence.

The draws of the ratio of chewing to non-chewing tobacco were then multiplied by the draws from the all smokeless tobacco prevalence model to adjust the estimates to chewing tobacco prevalence. These were then averaged to get the mean estimate. The variance across the ratios was calculated for each location, year, age, and sex, and was added to the variance from the original all smokeless tobacco draws.

Final chewing tobacco prevalence model

To calculate the final chewing tobacco prevalence, we ran an additional ST-GPR model with both the original chewing tobacco data (post-age-sex splitting), as well as the adjusted data. These adjusted data add more information to the model – as surveys will often only ask about all smokeless tobacco consumption – while taking into consideration the uncertainty from the ratio calculation.

Theoretical minimum-risk exposure level

The theoretical minimum risk exposure level is that everyone in the population has been a lifelong nonuser of chewing tobacco.

Relative risk

As in GBD 2017, we included outcomes based on the strength of available evidence supporting a causal relationship. There was sufficient evidence to include Lip and oral cavity cancer and Esophageal cancer as health outcomes caused by chewing tobacco use.

Relative risk estimates were derived from prospective cohort studies and population-based case-control studies. We used the same underlying effect size estimates from prospective cohort studies and population-based case-control studies as in GBD 2017. Briefly, we did not include hospital-based case control studies due to concerns over representativeness. We only included sources that adequately adjusted for major confounders, especially smoking status. Summary effect size estimates were calculated in R, using the 'metafor' package. We performed a random effects meta-analysis using the DerSimonian and Laird method, which does not assume a true effect size but considers each input study as selected from a random sample of all possible sets of studies for the outcome of interest. The random-effects method allows for more variation between the studies, and incorporates this variance into the estimation process. We used an inverse-variance weighting method to determine component study weights. We found significantly different relative risks for oral cancer for males and females, and estimated relative risks separately by sex for Lip and oral cavity cancer alone.

4.3. Alcohol use

Flowchart

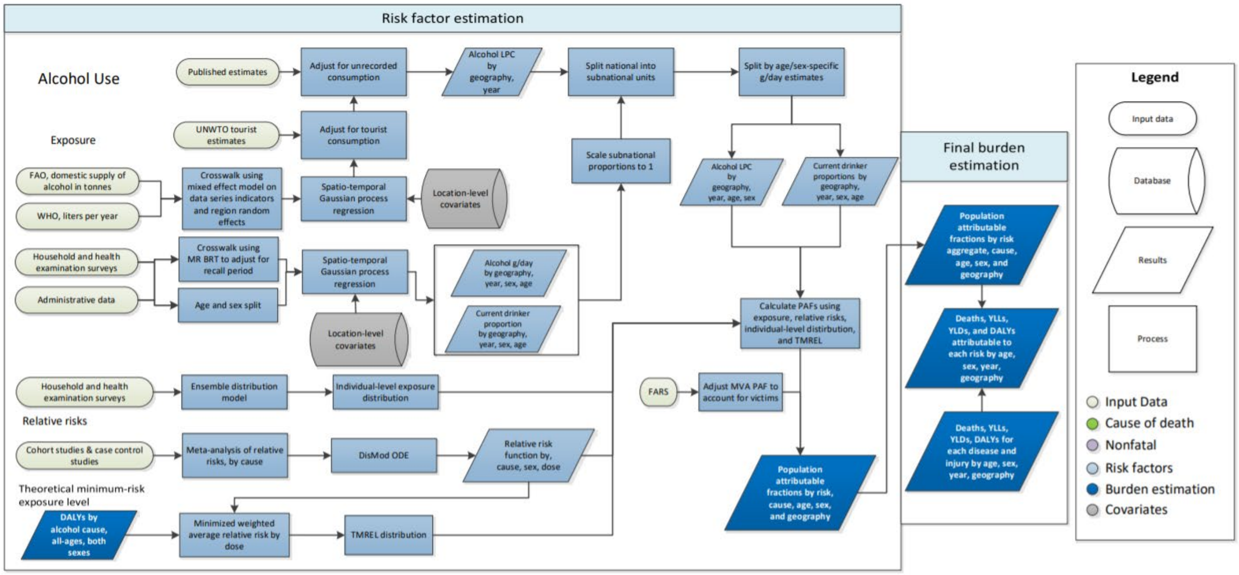

FAO = food and agriculture organization; WHO = World Health Organization; UWNTO = World Tourism Organization; MR BRT = a network meta-regression; DisMod ODE = the "engine" of DisMod-MR 2.1; TMREL = Theoretical minimum-risk exposure level; LPC = liters per capita; MVA = motor vehicle accidents; PAF = Population attributable fraction; FARS = Fatal Accident Reporting System; YLL = Years of life lost; YLD = Years lived with disability; DALYs = Disability-adjusted life-years.

Input data and methodological summary

Exposure

Case definition

We defined exposure as the grams per day of pure alcohol consumed among current drinkers. We constructed this exposure using the indicators outlined below:

1. Current drinkers, defined as the proportion of individuals who have consumed at least one alcoholic beverage (or some approximation) in a 12-month period.
2. Alcohol consumption (in grams per day), defined as grams of alcohol consumed by current drinkers, per day, over a 12-month period.
3. Alcohol liters per capita stock, defined in liters per capita of pure alcohol, over a 12-month period.

We also used three additional indicators to adjust alcohol exposure estimates to account for different types of bias:

1. Number of tourists within a location, defined as the total amount of visitors to a location within a 12-month period.

2. Tourists' duration of stay, defined as the number of days resided in a hosting country.

Unrecorded alcohol stock, defined as a percentage of the total alcohol stock produced outside established markets.

Input data

A systematic review of the literature was performed to extract data on our primary indicators. The Global Health Exchange (GHDx), IHME's online database of health-related data, was searched for population survey data containing participant-level information from which we could formulate the required alcohol use indicators on current drinkers and alcohol consumption. Data sources were included if they captured a sample representative of the geographical location under study. We documented relevant survey variables from each data source in a spreadsheet and extracted using STATA 13.1 and R 3.3. A total of 6172 potential data sources were available in the GHDx, of which 5091 have been screened and 1125 accepted.

Estimates of current drinking prevalence were split by age and sex where necessary. First, studies that reported prevalence for both sexes were split using a region-specific sex ratio estimated using MR-BRT. Second, where studies reported estimates across non-GBD age groups, these were split into standard five-year age groups using the global age pattern estimated by ST-GPR.

MR-BRT sex splitting adjustment factors for current drinking

**Data input**	**Gamma**	**Beta coefficient, log (95% CI)**	**Adjustment factor***
Female: Male	0	-0.16 (-0.17, -0.14)	0.85
Age < 50	0	0.06 (0.06, 0.06)	1.07
East Asia	0.36	-1.02 (-1.74, -0.29)	0.36
Southeast Asia	0.64	-1.06 (-2.34, 0.22)	0.35
Central Asia	0.41	-0.35 (-1.16, 0.46)	0.70
Central Europe	0.18	-0.21 (-0.58, 0.14)	0.80
Eastern Europe	0.10	-0.07 (-0.28, 0.14)	0.93
High-income Asia Pacific	1.27	-1.11 (-4.90, 2.68)	0.33
Western Europe	0.08	0.03 (-0.14, 0.20)	1.03
Southern Latin America	1.26	-0.67 (-4.18, 2.84)	0.51
High-income North America	0.09	-0.07 (-0.26, 0.11)	0.93
Caribbean	0.25	-0.52 (-1.02, -0.03)	0.59
Andean Latin America	0.76	-0.16 (-1.66, 1.34)	0.85
Central Latin America	0.30	-0.52 (-1.12, 0.08)	0.59
Tropical Latin America	0.08	-0.61 (-0.79, -0.44)	0.54
North Africa and Middle East	1.21	-1.44 (-3.91, 1.03)	0.24
South Asia	0.71	-1.17 (-2.57, 0.23)	0.31
Eastern sub-Saharan Africa	0.28	-0.53 (-1.10, 0.03)	0.58
Southern sub-Saharan Africa	0.20	-0.16 (-0.56, 0.23)	0.85
Western sub-Saharan Africa	0.32	-0.19 (-0.83, 0.45)	0.83
Oceania	0.94	-0.54 (-2.42, 1.34)	0.58

**Adjustment factor is the transformed beta coefficient in normal space and can be interpreted as the factor by which the alternative case definition is adjusted to reflect the ratio by which both-sex data points were split.*

To allow for the inclusion of data that did not meet our reference definition for current drinking, two crosswalks were performed using MR-BRT. The first crosswalk converted estimates of one-month drinking prevalence to what they would be if data represented estimates of 12-month drinking prevalence. This crosswalk incorporated two binary covariates: male and age ≥ 50. The second crosswalk converted estimates of one-week drinking prevalence to 12-month drinking prevalence. This crosswalk incorporated age < 20 and male as covariates. The covariates utilized in both crosswalks were included as both x and z covariates. A uniform prior of 0 was set as the upper bound for the beta coefficients to enforce the logical constraint that one-month and one-week prevalence could not be greater than 12-month prevalence.

MR-BRT crosswalk adjustment factors for alcohol use current drinking model

Data input	Reference or alternative case definition	Gamma	Beta coefficient, logit (95% CI)
12-month prevalence	Ref	---	---
1-month prevalence	Alt	0.22	-0.60 (-1.05, -0.16)
Age ≥ 50		0.13	0.16 (-0.10, 0.43)
Male		0.29	0.01 (-0.57, 0.59)
1-week prevalence	Alt	0.46	-1.51 (-2.42, -0.59)
Age < 20		0.47	-0.29 (-1.34, 0.76)
Male		0.00	0.38 (0.15, 0.60)

The methods for modelling supply-side-level data were changed substantially from those used in GBD 2017. The raw data are domestic supply (FAO[34]; WHO GISAH[35]) and retail supply (Euromonitor) of liters of pure ethanol consumed. Domestic supply is calculated as the sum of production and imports, subtracting exports. The WHO and FAO sources were combined, so that FAO data were only used if there were no data available for that location-year from WHO. This was done because the WHO source takes into consideration FAO values when available. Since the WHO data are given in more granular alcohol types, the following adjustments were made:

$$LPC\ Pure\ Ethanol = 0.13 * \left(\frac{Wine}{0.973}\right)$$

$$LPC\ Pure\ Ethanol = 0.05 * \left(\frac{Beer}{0.989}\right)$$

$$LPC\ Pure\ Ethanol = 0.4 * \left(\frac{Spirits}{0.91}\right)$$

Three outliering strategies are used to omit implausible datapoints and data that created implausible model fluctuations. First, estimates from the current drinking model are used to calculate the grams of alcohol consumed per drinker per day. A point is outliered if the grams of pure ethanol per drinker per day for a given source-location-year is greater than 100 (approximately ten drinks). These thresholds were chosen by using expert knowledge about reasonable consumption levels. In the second round of outliering, the mean liters per capita value over a ten-year window is calculated. If a point is over 70% of that mean value away from the mean value, it is outliered. The 70% limit was chosen using histograms of these distances. Additionally, some manual outliering is performed to account for edge cases. Finally, data smoothing is performed by taking a three-year rolling mean over each location-year.

Next, an imputation to fill in missing years is performed for all series to remove compositional bias from our final estimates. Since the data from our main sources cover different time periods, by imputing a complete time series for each data series, we reduce the probability that compositional bias of the sources is leading to biased final estimates. To impute the missing years for each series, we model the log ratio of each pair of sources as a function of an intercept and nested random effects on superregion, region, and location. The appropriate predicted ratio is multiplied by the source that we do have, which generates an estimated value for the missing source. For some locations where there was limited overlap between series, the predicted ratio did not make sense, and a regional ratio was used.

Finally, variance was calculated both across series (within a location-year) as well as across years (within a location-source). Additionally, if a location-year had one imputed point, the variance was multiplied by 2. If a location-year had two imputed points, the variance was multiplied by 4. The average estimates in each location-year were the input to an ST-GPR model. This uses a mixed-effects model modelled in log space with nested location random effects.

We obtained data on the number of tourists and their duration of stay from the UNWTO.[36] We applied a crosswalk across different tourist categories, similar to the one used for the liters per capita data, to arrive at a consistent definition (i.e., visitors to a country).

We obtained estimates on unrecorded alcohol stock from data available in WHO GISAH database,[35] consisting of 189 locations. For locations with no data available, the national or regional average was used.

For relative risks, in GBD 2016 we performed a systematic literature review of all cohort and case-control studies reporting a relative risk, hazard ratio, or odds ratio for any risk-outcome pairs studied in GBD 2016. Studies were included if they reported a categorical or continuous dose for alcohol consumption, as well as uncertainty measures for their outcomes, and the population under study was representative.

Modelling strategy

While population-based surveys provide accurate estimates of the prevalence of current drinkers, they typically underestimate real alcohol consumption levels.[37–39] As a result, we considered the liter per capita input to be a better estimate of overall volume of consumption. Per capita consumption, however, does not provide age- and sex-specific consumption estimates needed to compute alcohol attributable burden of disease. Therefore, we use the age-sex pattern of consumption among drinkers modelled from the population survey data and the overall volume of consumption from FAO, GISAH, and Euromonitor to determine the total amount of alcohol consumed within a location. In the paragraphs below, we outline how we estimated each primary input in the alcohol exposure model, as well as how we combined these inputs to arrive at our final estimate of grams per day of pure alcohol. We estimated all models below using 1000 draws.

For data obtained through surveys, we used spatiotemporal Gaussian process regression (ST-GPR) to construct estimates for each location/year/age/sex. We chose to use ST-GPR due to its ability to leverage information across the nearby locations or time periods. We also modelled the alcohol liters per capita (LPC) data, as well as the total number of tourists, using ST-GPR.

Given the heterogeneous nature of the estimates on unrecorded consumption, as well as the wide variation across countries and time periods, we took 1000 draws from the uniform distribution of the lowest and highest estimates available for a given country. We did this to incorporate the diffuse uncertainty within the unrecorded estimates reported. We used these 1000 draws in the equation below.

We adjusted the alcohol LPC for unrecorded consumption using the following equation:

$$Alcohol\ LPC = \frac{Alcohol\ LPC}{(1 - \%\ Unrecorded)}$$

We then adjusted the estimates for alcohol LPC for tourist consumption by adding in the per capita rate of consumption abroad and subtracting the per capita rate of tourist consumption domestically.

$$Alcohol\ LPC_d = Unadjusted\ Alcohol\ LPC_d + Alcohol\ LPC_{Domestic\ consumption\ abroad} - Alcohol\ LPC_{Tourist\ consumption\ domestically}$$

$$Alcohol\ LPC_i = \frac{\sum_l Tourist\ Population_l * Proportion\ of\ tourists_{i,l} * Unadjusted\ Alcohol\ LPC_l * \frac{Average\ length\ of\ stay_{i,l}}{365} *}{Population_d}$$

where:

l is the set of all locations, i is either Domestic consumption abroad *or* Tourist consumption domestically*, and d is a domestic location.*

After adjusting alcohol LPC by tourist consumption and unrecorded consumption for all location/years reported, sex-specific and age-specific estimates were generated by incorporating estimates modelled in ST-GPR for percentage of current drinkers within a location/year/sex/age, as well as consumption trends modelled in the ST-GPR grams per day model. We do this by first calculating the proportion of total consumption for a given location/year by age and sex, using the estimates of alcohol consumed per day, the population size, and the percentage of current drinkers. We then multiply this proportion of total stock for a given location/year/sex/age by the total stock for a given location/year to calculate the consumption in terms of liters per capita for a given location/year/sex/age. We then convert these estimates to be in terms of grams/per day. The following equations describe these calculations:

$$Proportion\ of\ total\ consumption_{l,y,s,a} = \frac{Alcohol\ \ g/day_{\ l,y,s,a} * Population_{l,y,s,a} * \%\ Current\ drinkers_{l,y,s,a}}{\sum_{s,a} Alcohol\ \ g/day_{l,y,s,a} * Population_{l,y,s,a} * \%\ Current\ drinkers_{l,y,s,a}}$$

$$Alcohol\ LPC_{l,y,s,a} = \frac{Alcohol\ LPC_{l,y} * Population_{l,y} * Proportion\ of\ total\ consumption_{l,y,s,a}}{\%\ Current\ drinkers_{l,y,s,a} * Population_{l,y,s,a}}$$

$$Alcohol\ g/day_{l,y,s,a} = Alcohol\ LPC_{l,y,s,a} * \frac{1000}{365}$$

where:

l is a location, y is a year, s is a sex, and a is an age group.

We then used the gamma distribution to estimate individual-level variation within location, year, sex, age drinking populations, following the recommendations of other published alcohol studies.[40,41] We chose parameters of the gamma distribution based on the mean and standard deviation of the 1,000 draws of alcohol g/day exposure for a given population. Standard deviation was calculated using the following formula.[40] We tested several alternative models using our data and found this model performed best.

$$standard\ deviation = mean * (0.087 * female + \ 1.171\ )$$

Theoretical minimum-risk exposure level

We calculated TMREL by first calculating the overall risk attributable to alcohol. We did this by weighting each relative risk curve by the share of overall DALYs for a given cause. We then took the minimum of this overall-risk curve as the TMREL of alcohol use. More formally,

$$TMREL = argmin\ average\ overall\ risk_{\omega}(g/day)$$

$$Average\ overall\ risk_{\omega}(g/day) = \sum_{i}^{\omega} RR_i(g/day) * \frac{DALY_i}{\sum_i^{\omega} DALY_i}$$

where:

ω is the set of causes associated with alcohol, i is a given cause from that set, DALY is the global DALY rate in 2010, and RR is the dose response curve for a given cause and exposure level in grams per day.

In other words, we chose TMREL as being the exposure that minimizes your risk of suffering burden from any given cause related to alcohol. We weight the risk for a particular cause in our aggregation by the proportion of DALYs due to that cause (e.g., since more observed people die from ischemic heart disease [IHD], we weight the risk for IHD more in the above calculation of average risk compared to, say, diabetes, even if both have the same relative risk for a given level of consumption).

Relative risks

We used the studies identified through the systematic review to calculate a dose-response, modelled using DisMod ODE. We chose DisMod ODE rather than a conventional mixed effects meta-regression because of its ability to estimate nonparametric splines over doses (i.e., for most alcohol causes, there is a nonlinear relationship with different doses) and incorporate heterogeneous doses through dose integration (i.e., most studies report doses categorically in wide ranges. DisMod ODE estimates specific doses when categories overlap across studies, through an integration step.). We used the results of the meta-regression to estimate a non-parametric curve for all doses between zero and 150 g/day and their corresponding relative risks. For all causes, we assumed the relative risk was the same for all ages and sexes, with the exception of ischemic heart disease, ischemic stroke, hemorrhagic stroke, and diabetes, which we estimated by sex.

For outcomes that are by definition caused by alcohol, such as liver cancer due to alcohol use or cirrhosis due to alcohol use, PAFs are set to 1. PAFs for cirrhosis due to all causes that are in excess of the proportion of all cirrhosis burden due to alcohol are proportionally redistributed over cirrhosis due to hepatitis B, cirrhosis due to hepatitis C, and cirrhosis due to other causes.

Regarding injuries outcomes, we constructed relative risks based on chronic exposure to alcohol rather than acute exposure immediately preceding injury, which has a weaker relationship to the outcome, though still significant.[42–47] We decided to use chronic exposure given the lack of available data on acute exposure, as well as the lack of cohort studies using acute exposure as a metric. Further, using chronic exposure allowed

us to construct relative risks curves for unintentional injuries, interpersonal violence, motor vehicle road injuries, and self-harm using the same method as reported above.

In the case of motor vehicle road injuries, we adjusted the PAF to account for victims of drunk drivers who are involved in accidents. Using data from the Fatality Analysis Reporting System (FARS) in the US,[48] we calculated the average number of fatalities in a car crash involving alcohol, as well as the percentage of those fatalities distributed by age and sex. We aggregated FARS data across the years 1985–2015, given there was little variation in the data temporally and the number of cases in old age groups had too much variance when constructing estimates by year. To adjust PAFs, we multiplied attributable deaths by the average number of fatalities from FARS and redistributed the PAF among each population, based on the probability of being a victim to a certain drunk driver by age and sex, based on the FARS data. The following equation describes this process:

$$Adjusted\ PAF_i = \frac{\sum_d PAF_d * DALY_d * Avg\ Fatalities_d * \ P(i\ is\ a\ victim)_d}{DALY_i}$$

where:

i is a population by location, year, age, sex and d is the set of all age and sex exposed groups within that location and year.

Population attributable fraction

For all causes, we defined PAF as:

$$PAF(x) = \frac{P_A + \int_0^{150} P(x) * RR_C(x)\, dx - 1}{P_A + \int_0^{150} P(x) * RR_C(x)\, dx} \qquad P(x) = P_C * \Gamma(\boldsymbol{p})$$

where:

*P_c is the prevalence of current drinkers, P_a is the prevalence of abstainers, $RR_c(x)$ is the relative risk function for current drinkers and **p** are parameters determined by the mean and sd of exposure*

We performed the above equation for 1000 draws of the exposure and relative risk models. We then used the estimated PAF draws to calculate YLL, YLDs, and DALYs, as per the other risk factors.

eReferences

For methodological summaries included on pages 11–48: ***The Global Burden of Disease (GBD) Study, Part I – Burden of Diseases Analysis, and Part II – Risk Factors Analysis***

1 Stevens G, Alkema L, Black R, et al. Guidelines for Accurate and Transparent Health Estimates Reporting: the GATHER statement. *Lancet* 2016; **388**: 19–23.

2 Kocarnik J, Compton K, Dean FE, et al. Cancer incidence, mortality, years of life lost, years lived with disability, and disability-adjusted life years for 29 cancer groups from 2010 to 2019: a systematic analysis for the Global Burden of Disease Study 2019. *JAMA Oncol*. 2022; **8**(3): 420–444. doi:10.1001/jamaoncol.2021.6987.

3 Force LM, Abdollahpour I, Advani SM, et al. The global burden of childhood and adolescent cancer in 2017: an analysis of the Global Burden of Disease Study 2017. *Lancet Oncol.* 2019; **20**: 1211–25.

4 Vos T, Lim SS, Abbafati C, et al. Global burden of 369 diseases and injuries in 204 countries and territories, 1990–2019: a systematic analysis for the Global Burden of Disease Study 2019. *Lancet* 2020; **396**: 1204–22.

5 Murray CJL, Aravkin AY, Zheng P, et al. Global burden of 87 risk factors in 204 countries and territories, 1990–2019: a systematic analysis for the Global Burden of Disease Study 2019. *Lancet* 2020; **396**: 1223–49.

6 World Health Organization. International Classification of Diseases: Ninth Revision, basic tabulation list with alphabetic index. Accessed March 1, 2021. https://apps.who.int/iris/handle/10665/39473

7 World Health Organization. International Statistical Classification of Diseases and Related Health Problems, 10th revision, 2nd edition. Accessed March 1, 2021. https://apps.who.int/iris/handle/10665/42980

8 Surveillance, Epidemiology, and End Results (SEER) Program (Www.Seer.Cancer.Gov) SEER*Stat Database: Incidence – SEER 18.

9 Doll R, Payne P, Waterhouse J, editors. *Cancer Incidence in Five Continents, Vol. I.* Geneva: Union Internationale Contre le Cancer, 1966 https://publications.iarc.fr/Non-Series-Publications/Other-Non-Series-Publications/Cancer-Incidence-In-Five-Continents-Volume-I-1966 (accessed Feb 24, 2021).

10 Doll R, Muir CS, Waterhouse JA. *Cancer Incidence in Five Continents, Vol. II.* Geneva: Union Internationale Contre le Cancer, 1970.

11 Waterhouse J, Muir C, Correa P, Powell J. *Cancer Incidence in Five Continents III.* Lyon: IARC; 1976.

12 Waterhouse J, Muir C, Shanmugaratnam K, Powell J. *Cancer Incidence in Five Continents IV.* Lyon: IARC; 1982.

13 Muir C, Mack T, Powell J, Whelan S. *Cancer Incidence in Five Continents V.* Lyon: IARC; 1987.

14 Parkin D, Muir C, Whelan S, Gao Y, Ferlay J, Powell J. *Cancer Incidence in Five Continents VI.* Lyon: IARC; 1992.

15 Parkin D, Whelan S, Ferlay J, Raymond L, Young J. *Cancer Incidence in Five Continents VII.* Lyon: IARC; 1997.

16 Parkin D, Whelan S, Ferlay J, Teppo L, Thomas D. *Cancer Incidence in Five Continents VIII.* Lyon: IARC; 2002.

17 Curado M, Edwards B, Shin H, et al. *Cancer Incidence in Five Continents IX*. Lyon: IARC; 2007. http://www.iarc.fr/en/publications/pdfs-online/epi/sp160/CI5vol9-A.pdf.

18 Forman D, Bray F, Brewster D, et al. *Cancer Incidence in Five Continents X.* http://ci5.iarc.fr. Published 2013.

19 Bray F, Colombet M, Mery L, et al., editors. *Cancer Incidence in Five Continents.* Lyon, France: International Agency for Research on Cancer, 2017 https://ci5.iarc.fr.

20 Engholm G, Ferlay J, Christensen N, et al. NORDCAN--a Nordic tool for cancer information, planning, quality control and research. *Acta Oncol* 2010; **49**: 725–36.

21 Steliarova-Foucher E, O'Callaghan M, Ferlay J, et al. The European Cancer Observatory: A new data resource. *Eur J Cancer* 2015; **51**: 1131–43.

22 Barber RM, Fullman N, Sorensen RJD, et al. Healthcare Access and Quality Index based on mortality from causes amenable to personal health care in 195 countries and territories, 1990–2015: a novel analysis from the Global Burden of Disease Study 2015. *Lancet* 2017; **390**: 231–66.

23 Foreman KJ, Lozano R, Lopez AD, Murray CJL. Modeling causes of death: an integrated approach using CODEm. *Popul Health Metr* 2012; **10**: 1.

24 Wang H, Abbas KM, Abbasifard M, et al. Global age-sex-specific fertility, mortality, healthy life expectancy (HALE), and population estimates in 204 countries and territories, 1950–2019: a comprehensive demographic analysis for the Global Burden of Disease Study 2019. *Lancet* 2020; **396**: 1160–203.

25 Asadzadeh Vostakolaei F, Karim-Kos HE, Janssen-Heijnen MLG, Visser O, Verbeek ALM, Kiemeney LALM. The validity of the mortality to incidence ratio as a proxy for site-specific cancer survival. *Eur J Public Health* 2011; **21**: 573–7.

26 SEER*Stat Software. 2014 http://seer.cancer.gov/seerstat/.

27 Fitzmaurice C, Abate D, Abbasi N, et al. Global, Regional, and National Cancer Incidence, Mortality, Years of Life Lost, Years Lived With Disability, and Disability-Adjusted Life-Years for 29 Cancer Groups, 1990 to 2017: A Systematic Analysis for the Global Burden of Disease Study. *JAMA Oncol* 2019; **5**: 1749–68.

28 SEER Cancer Statistics Review 1975–2011. http://seer.cancer.gov/csr/1975_2011/results_merged/topic_survival_by_year_dx.p.

29 Neal RD, Din NU, Hamilton W, et al. Comparison of cancer diagnostic intervals before and after implementation of NICE guidelines: analysis of data from the UK General Practice Research Database. *British Journal of Cancer* 2014; **110**: 584–92.

30 Surveillance, Epidemiology, and End Results (SEER) Program (www.seer.cancer.gov) SEER*Stat Database: Incidence – SEER 18 Regs Research Data + Hurricane Katrina Impacted Louisiana Cases, Nov 2012 Sub (1973–2010 varying) – Linked To County Attributes – Total U.S., 1969–2011 Counties, National Cancer Institute, DCCPS, Surveillance Research Program, Surveillance Systems Branch, released April 2013, based on the November 2012 submission.

31 Murray CJL, Lopez AD. Global mortality, disability, and the contribution of risk factors: Global Burden of Disease Study. *Lancet* 1997; **349**: 1436–42.

32 Murray CJL, Lopez AD. On the comparable quantification of health risks: lessons from the Global Burden of Disease Study. *Epidemiology* 1999; **10**: 594–605.

33 Ng M, Freeman MK, Fleming TD, et al. Smoking Prevalence and Cigarette Consumption in 187 Countries, 1980–2012. *JAMA* 2014 Jan 8; **311**(2): 183–92.

34 Food and Agriculture Organization of the United Nations (FAO). FAOSTAT Food Balance Sheets, October 2014. Rome, Italy: Food and Agriculture Organization of the United Nations (FAO).

35 World Health Organization (WHO). WHO Global Health Observatory – Recorded adult per capita alcohol consumption, Total per country. Geneva, Switzerland: World Health Organization (WHO).

36 UN World Tourism Organization (UNWTO). UN World Tourism Organization Compendium of Tourism Statistics 2015 [Electronic]. Madrid, Spain: UN World Tourism Organization (UNWTO), 2016.

37 Ramstedt M. How much alcohol do you buy? A comparison of self-reported alcohol purchases with actual sales. *Addiction* 105.4 (2010): 649–654.

38 Stockwell T, Donath S, Cooper-Stanbury M, Chikritzhs T, Catalano P, Mateo C. Under-reporting of alcohol consumption in household surveys: a comparison of quantity–frequency, graduated–frequency and recent recall. *Addiction* 99.8 (2004): 1024–1033.

39 Kerr WC, and Greenfield TK. Distribution of alcohol consumption and expenditures and the impact of improved measurement on coverage of alcohol sales in the 2000 National Alcohol Survey. *Alcoholism: Clinical and Experimental Research* 31.10 (2007): 1714–1722.

40 Kehoe T, Gmel G, Shield KD, Gmel G, Rehm J. Determining the best population-level alcohol consumption model and its impact on estimates of alcohol-attributable harms. *Population Health Metrics* 10 6. (2012).

41 Rehm J, Kehoe T, Gmel G, et al. Statistical modeling of volume of alcohol exposure for epidemiological studies of population health: the US example. *Popul Health Metrics* **8**, 3 (2010). https://doi.org/10.1186/1478-7954-8-3

42 Vinson DC, Guilherme B, Cheryl JC. The risk of intentional injury with acute and chronic alcohol exposures: a case-control and case-crossover study. *Journal of studies on alcohol* 64.3 (2003): 350–357.

43 Vinson DC, Maclure M, Reidinger C, et al. A population-based case-crossover and case-control study of alcohol and the risk of injury. *Journal of studies on alcohol* 64.3 (2003): 358–366.

44 Chen L-H, Baker SP, Li G. Drinking history and risk of fatal injury: comparison among specific injury causes. *Accident Analysis & Prevention* 37.2 (2005): 245–251.

45 Bell NS, Amoroso PJ, Yore MM, Smith GS, Jones BH. Self-reported risk-taking behaviors and hospitalization for motor vehicle injury among active duty army personnel. *American journal of preventive medicine* 18.3 (2000): 85–95.

46 Margolis KL, Kerani RP, McGovern P, et al. Risk factors for motor vehicle crashes in older women. *The Journals of Gerontology Series A: Biological Sciences and Medical Sciences* 57.3 (2002): M186–M191.

47 Sorock GS, Chen L-H, Gonzalgo SR, Baker SP. Alcohol-drinking history and fatal injury in older adults. *Alcohol* 40.3 (2006): 193–199.

48 Fatal Accident Reporting System (FARS). National Highway Traffic Safety Administration, National Center for Statistics and Analysis Data Reporting and Information Division (NVS-424); 1985, 1990, 1995, 2000, 2005, 2010, 2015.

Additional Methodology Tables and Figures

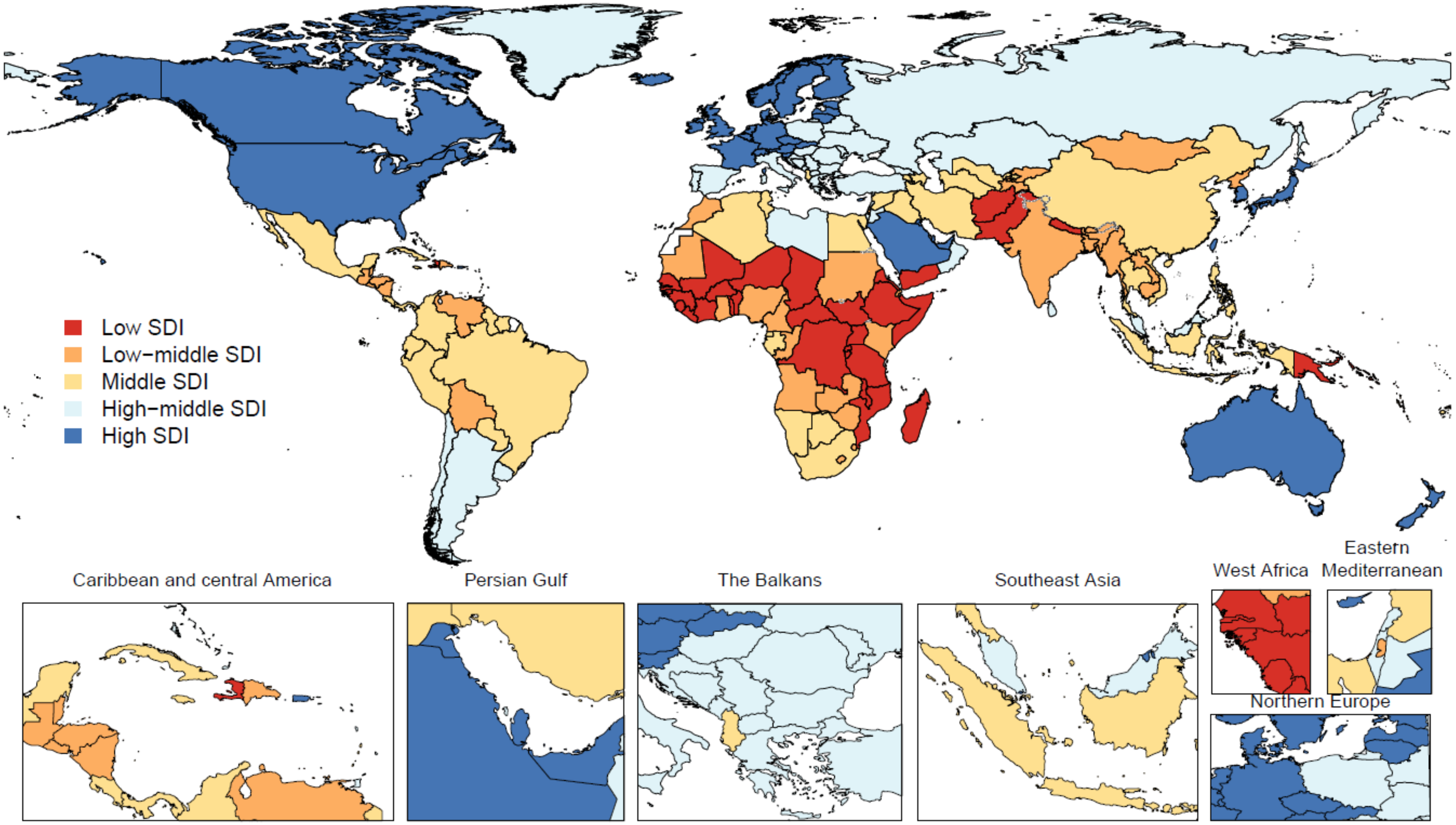

eFigure 3. Socio-demographic Index quintiles for the Global Burden of Disease Study 2019
SDI = Socio-demographic Index.

eTable 7. Socio-demographic Index (SDI) quintiles for countries and territories estimated in GBD 2019

SDI Quintile	Locations included based on SDI values in 2019 from GBD 2019 results
High SDI	Andorra, Australia, Austria, Belgium, Bermuda, Brunei Darussalam, Canada, Cyprus, Czechia, Denmark, Estonia, Finland, France, Germany, Guam, Iceland, Ireland, Japan, Kuwait, Latvia, Lithuania, Luxembourg, Monaco, Netherlands, New Zealand, Norway, Puerto Rico, Qatar, San Marino, Saudi Arabia, Singapore, Slovakia, Slovenia, Republic of Korea, Sweden, Switzerland, Taiwan (Province of China), United Arab Emirates, United Kingdom, United States of America
High-middle SDI	American Samoa, Antigua and Barbuda, Argentina, Bahamas, Bahrain, Barbados, Belarus, Bosnia and Herzegovina, Bulgaria, Chile, Cook Islands, Croatia, Dominica, Georgia, Greece, Greenland, Hungary, Israel, Italy, Jordan, Kazakhstan, Lebanon, Libya, Malaysia, Malta, Mauritius, Republic of Moldova, Montenegro, Niue, North Macedonia, Northern Mariana Islands, Oman, Palau, Poland, Portugal, Romania, Russian Federation, Saint Kitts and Nevis, Serbia, Seychelles, Spain, Sri Lanka, Trinidad and Tobago, Turkey, Ukraine, United States Virgin Islands, Uruguay
Middle SDI	Albania, Algeria, Armenia, Azerbaijan, Botswana, Brazil, China, Colombia, Costa Rica, Cuba, Ecuador, Egypt, Equatorial Guinea, Fiji, Gabon, Grenada, Guyana, Indonesia, Iran (Islamic Republic of), Iraq, Jamaica, Mexico, Namibia, Nauru, Panama, Paraguay, Peru, Philippines, Saint Lucia, Saint Vincent and the Grenadines, Samoa, South Africa, Suriname, Syrian Arab Republic, Thailand, Tokelau, Tonga, Tunisia, Turkmenistan, Uzbekistan, Viet Nam
Low-middle SDI	Angola, Bangladesh, Belize, Bhutan, Bolivia (Plurinational State of), Cambodia, Cameroon, Cabo Verde, Congo , Djibouti, Dominican Republic, El Salvador, Eswatini, Ghana, Guatemala, Honduras, India, Kenya, Kiribati, Kyrgyzstan, Lao People's Democratic Republic, Lesotho, Maldives, Marshall Islands, Mauritania, Micronesia (Federated States of), Mongolia, Morocco, Myanmar, Nicaragua, Nigeria, Democratic People's Republic of Korea, Palestine, São Tomé and Príncipe, Sudan, Tajikistan, Timor-Leste, Tuvalu, Vanuatu, Venezuela (Bolivarian Republic of), Zambia, Zimbabwe

Low SDI	Afghanistan, Benin, Burkina Faso, Burundi, Central African Republic, Chad, Comoros, Côte d'Ivoire, Democratic Republic of the Congo, Eritrea, Ethiopia, Gambia, Guinea, Guinea-Bissau, Haiti, Liberia, Madagascar, Malawi, Mali, Mozambique, Nepal, Niger, Pakistan, Papua New Guinea, Rwanda, Senegal, Sierra Leone, Solomon Islands, Somalia, South Sudan, United Republic of Tanzania, Togo, Uganda, Yemen

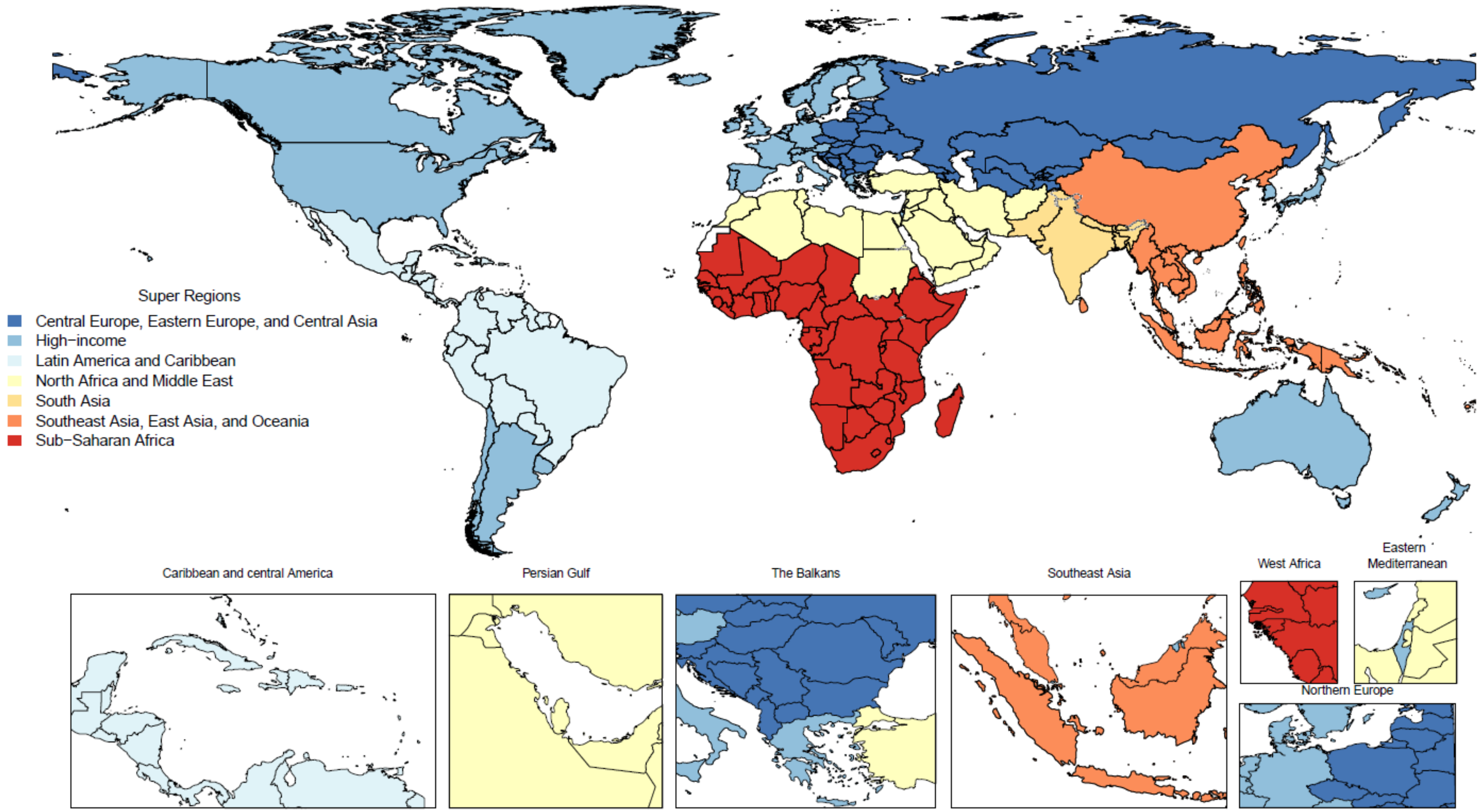

eFigure 4. Map of GBD world super-regions, 2019

There are several geographic locations where estimates are not available (e.g., Western Sahara, French Guiana) as they were not modelled locations in the Global Burden of Diseases, Injuries, and Risk Factors 2019 study; these locations are white in this map. GBD=Global Burden of Diseases, Injuries, and Risk Factors Study.

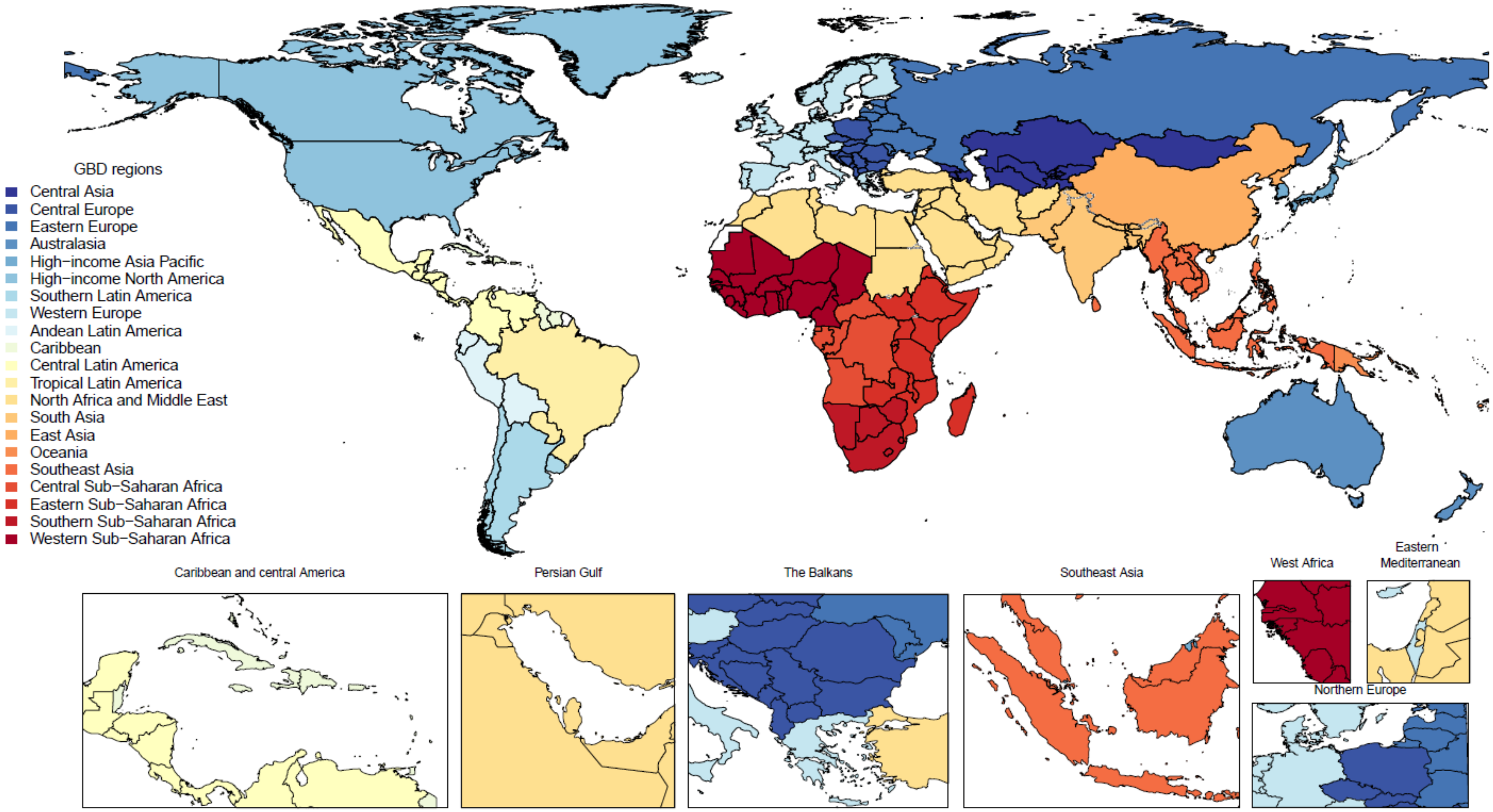

eFigure 5. Map of GBD world regions, 2019
There are several geographic locations where estimates are not available (e.g., Western Sahara, French Guiana) as they were not modelled locations in the Global Burden of Diseases, Injuries, and Risk Factors 2019 study; these locations are white in this map. GBD=Global Burden of Diseases, Injuries, and Risk Factors Study.

Additional Results in eTables and eFigures

eTable 8. Global and regional deaths, incidence, and DALYs counts and age-standardized rates (per 100 000) for Lip and oral cavity, both sexes combined, in 2019, and change in age-standardized rates from 1990 to 2019

	Deaths			Incidence			DALYs		
Location *(Global, **Super-region**, **region**, or SDI Quintile)*	**Counts, 2019 (95% UI)**	**Age-standardized rate, 2019 (95% UI)**	**% change of age-standardized rates, 1990-2019 (95% UI)**	**Counts, 2019 (95% UI)**	**Age-standardized rate, 2019 (95% UI)**	**% change of age-standardized rates, 1990-2019 (95% UI)**	**Counts, 2019 (95% UI)**	**Age-standardized rate, 2019 (95% UI)**	**% change of age-standardized rates, 1990-2019 (95% UI)**
Global	199 000 (181 000 to 217 000)	3.8 (3.5 to 4.2)	0.0 (-12.0 to 12.1)	370 000 (338 000 to 401 000)	7.1 (6.5 to 7.7)	5.4 (-5.9 to 15.9)	5 450 000 (4 950 000 to 5 970 000)	103.3 (93.9 to 113.2)	-1.5 (-14.3 to 11.1)
Global Female	67 400 (60 400 to 75 300)	2.5 (2.2 to 2.7)	10.4 (-0.5 to 22.4)	128 000 (116 000 to 141 000)	4.7 (4.2 to 5.2)	18.4 (7.6 to 29.6)	1 720 000 (1 540 000 to 1 920 000)	63.2 (56.5 to 70.8)	9.0 (-2.8 to 21.8)
Global Male	131 000 (117 000 to 145 000)	5.4 (4.8 to 6.0)	-5.9 (-20.3 to 9.7)	242 000 (218 000 to 267 000)	9.7 (8.7 to 10.7)	-1.4 (-15.0 to 11.9)	3 730 000 (3 300 000 to 4 150 000)	145.7 (129.3 to 161.8)	-6.1 (-21.3 to 10.3)
Low SDI	19 800 (17 500 to 22 300)	5.9 (5.3 to 6.6)	3.4 (-14.6 to 26.8)	30 300 (26 700 to 34 200)	8.4 (7.5 to 9.5)	12.7 (-7.4 to 38.2)	615 000 (541 000 to 697 000)	158.5 (140.2 to 178.2)	2.2 (-17.0 to 26.6)
Low-middle SDI	60 000 (52 700 to 68 600)	7.0 (6.1 to 7.9)	1.4 (-18.6 to 24.0)	94 500 (82 100 to 107 000)	10.4 (9.1 to 11.8)	13.2 (-9.0 to 37.1)	1 760 000 (1 550 000 to 2 030 000)	186.4 (163.8 to 214.1)	1.8 (-18.4 to 24.8)
Middle SDI	52 000 (45 900 to 58 600)	3.4 (3.0 to 3.8)	11.5 (-4.5 to 28.4)	93 800 (82 400 to 105 000)	5.8 (5.1 to 6.5)	29.8 (11.4 to 48.1)	1 420 000 (1 240 000 to 1 600 000)	85.3 (74.9 to 96.6)	8.6 (-8.5 to 25.6)
High-middle SDI	37 400 (34 500 to 40 400)	2.9 (2.7 to 3.2)	-10.7 (-18.7 to -2.9)	71 400 (64 600 to 77 900)	5.6 (5.1 to 6.1)	0.2 (-9.7 to 10.0)	987 000 (905 000 to 1 070 000)	77.8 (71.3 to 84.2)	-13.2 (-21.3 to -4.8)
High SDI	29 300 (27 000 to 30 900)	2.5 (2.3 to 2.6)	-20.4 (-23.7 to -17.0)	80 300 (72 500 to 88 600)	7.4 (6.7 to 8.2)	-14.2 (-22.3 to -5.5)	666 000 (629 000 to 702 000)	64.2 (60.8 to 67.9)	-25.1 (-28.4 to -21.1)
Central Europe, Eastern Europe, and Central Asia	16 100 (14 600 to 17 600)	4.2 (3.8 to 4.6)	3.5 (-6.5 to 13.2)	25 600 (23 400 to 27 800)	6.7 (6.1 to 7.3)	15.0 (5.0 to 25.3)	449 000 (407 000 to 490 000)	120.3 (109.3 to 131.3)	3.1 (-7.2 to 13.2)
**Central Asia**	1 370 (1 240 to 1 520)	2.9 (2.6 to 3.2)	5.2 (-7.6 to 18.6)	2 380 (2 150 to 2 640)	4.8 (4.4 to 5.3)	11.8 (-1.6 to 26.2)	40 900 (36 900 to 45 700)	76.8 (69.5 to 85.2)	0.2 (-11.9 to 13.2)
**Central Europe**	5 550 (4 870 to 6 260)	4.4 (3.9 to 5.0)	-1.2 (-13.9 to 11.1)	10 300 (8 970 to 11 700)	8.4 (7.3 to 9.6)	11.7 (-2.3 to 26.0)	146 000 (127 000 to 165 000)	125.6 (108.8 to 142.4)	-3.6 (-16.9 to 9.1)
**Eastern Europe**	9 210 (8 150 to 10 400)	4.4 (3.9 to 5.0)	9.6 (-3.5 to 24.5)	12 900 (11 600 to 14 500)	6.3 (5.6 to 7.0)	21.0 (6.9 to 36.4)	262 000 (231 000 to 295 000)	130.9 (115.5 to 147.5)	12.4 (-1.7 to 28.3)
High-income	31 000 (28 300 to 32 400)	2.3 (2.2 to 2.4)	-25.2 (-28.3 to -22.9)	86 100 (77 400 to 95 100)	7.2 (6.4 to 7.9)	-18.7 (-27.2 to -9.9)	674 000 (639 000 to 699 000)	58.4 (55.8 to 60.4)	-31.2 (-33.4 to -29.1)
**Australasia**	995 (897 to 1 070)	3.2 (2.9 to 3.5)	-44.3 (-48.3 to -40.3)	1 840 (1 500 to 2 250)	6.4 (5.2 to 7.8)	-39.4 (-51.1 to -25.6)	21 600 (20 000 to 23 200)	77.6 (72.2 to 83.2)	-47.0 (-50.9 to -43.0)
**High-income Asia Pacific**	5 550 (4 710 to 6 030)	1.8 (1.6 to 1.9)	7.2 (-0.8 to 13.0)	8 470 (7 190 to 9 670)	3.2 (2.8 to 3.7)	15.8 (1.8 to 32.3)	96 600 (87 500 to 103 000)	40.3 (37.6 to 42.4)	-0.9 (-5.8 to 3.6)
**High-income North America**	8 890 (8 370 to 9 250)	2.2 (2.1 to 2.3)	-28.9 (-31.5 to -26.6)	33 100 (28 700 to 38 300)	8.8 (7.6 to 10.2)	-19.8 (-30.7 to -6.8)	203 000 (194 000 to 211 000)	55.4 (53.0 to 57.5)	-33.3 (-35.8 to -31.0)

© 2023 Cunha AR et al. *JAMA Oncol.*

	Deaths			Incidence			DALYs		
Location (*Global*, ***Super-region***, **region,** or *SDI Quintile*)	**Counts, 2019 (95% UI)**	**Age-standardized rate, 2019 (95% UI)**	**% change of age-standardized rates, 1990-2019 (95% UI)**	**Counts, 2019 (95% UI)**	**Age-standardized rate, 2019 (95% UI)**	**% change of age-standardized rates, 1990-2019 (95% UI)**	**Counts, 2019 (95% UI)**	**Age-standardized rate, 2019 (95% UI)**	**% change of age-standardized rates, 1990-2019 (95% UI)**
**Southern Latin America**	1 170 (1 090 to 1 250)	2.2 (2.1 to 2.4)	-14.7 (-20.5 to -8.7)	2 130 (1 670 to 2 670)	4.1 (3.2 to 5.2)	-5.9 (-26.6 to 19.2)	28 000 (26 200 to 30 000)	55.2 (51.6 to 59.1)	-19.9 (-25.7 to -13.4)
**Western Europe**	14 400 (13 300 to 15 100)	2.6 (2.5 to 2.7)	-28.0 (-30.8 to -25.2)	40 500 (35 100 to 46 700)	8.1 (7.0 to 9.4)	-20.3 (-31.3 to -7.8)	325 000 (308 000 to 340 000)	68.2 (65.1 to 71.4)	-34.3 (-37.0 to -31.2)
Latin America and Caribbean	9 870 (9 070 to 10 600)	2.7 (2.4 to 2.9)	-16.7 (-22.8 to -10.3)	16 800 (15 500 to 18 100)	4.5 (4.1 to 4.8)	-6.4 (-13.8 to 1.2)	251 000 (234 000 to 270 000)	66.0 (61.2 to 70.9)	-17.3 (-23.4 to -10.7)
**Andean Latin America**	519 (421 to 626)	1.5 (1.2 to 1.8)	-9.0 (-27.4 to 14.0)	853 (689 to 1 040)	2.4 (1.9 to 2.9)	6.5 (-16.3 to 34.6)	12 500 (10 000 to 15 400)	34.3 (27.5 to 42.0)	-14.7 (-33.8 to 8.9)
**Caribbean**	1 240 (1 060 to 1 440)	3.8 (3.2 to 4.4)	-9.1 (-22.3 to 5.6)	2 090 (1 780 to 2 440)	6.4 (5.4 to 7.4)	-2.2 (-17.1 to 14.8)	29 800 (25 500 to 34 900)	90.9 (77.6 to 106.4)	-8.6 (-21.9 to 6.8)
**Central Latin America**	2 470 (2 130 to 2 870)	1.7 (1.5 to 2.0)	-20.1 (-30.7 to -8.2)	4 210 (3 590 to 4 880)	2.8 (2.4 to 3.3)	-7.5 (-20.6 to 6.7)	58 600 (50 300 to 68 500)	38.5 (33.1 to 45.0)	-19.7 (-31.1 to -6.7)
**Tropical Latin America**	5 640 (5 280 to 5 960)	3.7 (3.4 to 3.9)	-14.7 (-19.9 to -9.1)	9 700 (9 120 to 10 200)	6.2 (5.8 to 6.5)	-4.1 (-10.3 to 2.4)	151 000 (142 000 to 159 000)	94.9 (89.7 to 100.2)	-15.2 (-20.3 to -9.5)
North Africa and Middle East	3 510 (3 080 to 4 060)	1.3 (1.2 to 1.5)	-7.9 (-24.7 to 15.2)	6 550 (5 720 to 7 580)	2.3 (2.0 to 2.6)	9.7 (-10.4 to 37.1)	98 500 (85 100 to 115 000)	32.2 (28.0 to 37.3)	-11.1 (-27.8 to 11.5)
South Asia	88 800 (76 500 to 104 000)	10.0 (8.6 to 11.7)	-3.8 (-23.7 to 18.6)	142 000 (119 000 to 164 000)	15.1 (12.8 to 17.5)	9.3 (-14.1 to 33.9)	2 650 000 (2 280 000 to 3 100 000)	271.1 (233.8 to 317.1)	-1.1 (-22.0 to 22.2)
Southeast Asia, East Asia, and Oceania	40 700 (35 300 to 46 300)	2.5 (2.2 to 2.8)	15.7 (-1.5 to 34.6)	80 800 (71 000 to 92 200)	4.7 (4.1 to 5.3)	42.9 (22.4 to 65.6)	1 060 000 (922 000 to 1 210 000)	60.4 (52.4 to 68.7)	13.3 (-4.7 to 33.0)
**East Asia**	25 200 (21 500 to 29 600)	2.0 (1.7 to 2.3)	30.6 (5.9 to 60.0)	51 900 (44 100 to 60 800)	3.9 (3.3 to 4.6)	67.9 (38.1 to 106.0)	654 000 (556 000 to 772 000)	48.7 (41.6 to 57.2)	26.5 (1.4 to 56.5)
**Oceania**	139 (107 to 191)	3.2 (2.5 to 4.2)	8.3 (-10.4 to 32.7)	244 (188 to 329)	5.0 (3.9 to 6.5)	10.4 (-8.9 to 35.7)	4 430 (3 330 to 6 210)	82.2 (63.2 to 112.5)	6.8 (-13.6 to 33.9)
**Southeast Asia**	15 300 (12 900 to 18 200)	4.2 (3.6 to 5.0)	1.3 (-16.5 to 21.5)	28 700 (24 000 to 34 400)	7.4 (6.2 to 8.9)	14.0 (-6.7 to 36.2)	403 000 (334 000 to 481 000)	98.7 (82.4 to 117.8)	-3.7 (-21.4 to 15.8)
Sub-Saharan Africa	8 630 (7 490 to 9 820)	2.9 (2.6 to 3.3)	3.7 (-8.7 to 17.7)	12 800 (11 100 to 14 600)	4.0 (3.5 to 4.6)	7.7 (-5.7 to 21.8)	263 000 (225 000 to 302 000)	76.1 (65.8 to 86.8)	-0.1 (-13.7 to 15.2)
**Central Sub-Saharan Africa**	1 050 (790 to 1 340)	3.2 (2.4 to 4.0)	-3.8 (-24.9 to 22.8)	1 500 (1 130 to 1 900)	4.2 (3.1 to 5.3)	0.3 (-23.6 to 30.1)	32 200 (24 000 to 41 200)	80.4 (60.5 to 102.7)	-6.2 (-29.4 to 22.8)
**Eastern Sub-Saharan Africa**	4 040 (3 430 to 4 640)	3.8 (3.2 to 4.3)	7.2 (-9.2 to 26.5)	6 000 (5 030 to 6 940)	5.2 (4.4 to 5.9)	12.3 (-5.7 to 33.8)	128 000 (107 000 to 149 000)	101.5 (85.8 to 117.1)	3.2 (-14.3 to 25.2)
**Southern Sub-Saharan Africa**	1 390 (1 280 to 1 540)	3.9 (3.6 to 4.3)	-9.9 (-21.0 to 3.5)	2 190 (2 000 to 2 430)	5.9 (5.3 to 6.5)	-7.4 (-18.1 to 5.3)	40 300 (36 700 to 44 800)	102.6 (93.7 to 113.7)	-13.2 (-23.5 to -1.1)
**Western Sub-Saharan Africa**	2 140 (1 830 to 2 480)	1.9 (1.6 to 2.1)	14.8 (-3.3 to 34.9)	3 130 (2 650 to 3 650)	2.5 (2.2 to 2.9)	19.2 (-0.2 to 41.3)	62 100 (51 600 to 72 600)	45.3 (38.4 to 52.5)	9.8 (-8.8 to 32.4)

SDI=Socio-demographic Index; DALYs=disability-adjusted life years; UI=uncertainty interval. See eFigure 3 (p53) and eTable 7 (p54) for details and definitions of the SDI regions.

eTable 9. Global and regional deaths, incidence, and DALYs counts and age-standardized rates (per 100 000) for Other pharynx cancer, both sexes combined, in 2019, and change in age-standardized rates from 1990 to 2019

	Deaths			Incidence			DALYs		
Location *(Global, **Super-region**, **region,** or SDI Quintile)*	**Counts, 2019 (95% UI)**	**Age-standardized rate, 2019 (95% UI)**	**% change of age-standardized rates, 1990-2019 (95% UI)**	**Counts, 2019 (95% UI)**	**Age-standardized rate, 2019 (95% UI)**	**% change of age-standardized rates, 1990-2019 (95% UI)**	**Counts, 2019 (95% UI)**	**Age-standardized rate, 2019 (95% UI)**	**% change of age-standardized rates, 1990-2019 (95% UI)**
Global	114 000 (103 000 to 126 000)	2.2 (2.0 to 2.4)	9.5 (-6.2 to 25.0)	167 000 (153 000 to 180 000)	3.2 (2.9 to 3.4)	24.7 (9.7 to 37.5)	3 230 000 (2 900 000 to 3 570 000)	60.9 (54.6 to 67.2)	5.7 (-9.8 to 20.8)
Global Female	26 200 (22 500 to 30 500)	1.0 (0.8 to 1.1)	6.1 (-14.4 to 28.0)	37 600 (33 100 to 42 300)	1.4 (1.2 to 1.5)	22.6 (1.0 to 43.1)	731 000 (627 000 to 857 000)	26.9 (23.1 to 31.6)	1.9 (-19.5 to 24.5)
Global Male	88 000 (78 000 to 98 700)	3.5 (3.1 to 4.0)	9.7 (-9.9 to 26.8)	129 000 (116 000 to 142 000)	5.1 (4.6 to 5.6)	24.6 (7.7 to 39.1)	2 500 000 (2 220 000 to 2 820 000)	96.9 (85.8 to 109.0)	6.7 (-12.5 to 24.1)
Low SDI	9 460 (8 110 to 11 000)	2.8 (2.4 to 3.3)	13.0 (-10.9 to 42.2)	9 910 (8 440 to 11 600)	2.9 (2.4 to 3.3)	15.3 (-10.4 to 44.6)	282 000 (242 000 to 330 000)	74.8 (64.1 to 87.1)	6.8 (-15.9 to 35.2)
Low-middle SDI	44 700 (38 600 to 51 800)	5.1 (4.4 to 5.9)	14.1 (-11.1 to 40.3)	47 700 (40 900 to 54 900)	5.3 (4.6 to 6.1)	18.9 (-6.7 to 45.9)	1 300 000 (1 120 000 to 1 500 000)	137.6 (119.2 to 159.4)	10.7 (-13.7 to 36.7)
Middle SDI	25 600 (22 300 to 28 800)	1.6 (1.4 to 1.8)	13.0 (-7.0 to 30.1)	31 400 (27 400 to 35 200)	1.9 (1.7 to 2.1)	31.2 (7.2 to 49.2)	730 000 (634 000 to 824 000)	43.0 (37.4 to 48.4)	10.2 (-9.9 to 27.4)
High-middle SDI	18 000 (16 700 to 19 300)	1.4 (1.3 to 1.5)	-10.2 (-17.8 to -2.2)	32 100 (29 300 to 34 800)	2.5 (2.3 to 2.7)	20.4 (9.1 to 31.9)	508 000 (470 000 to 544 000)	39.5 (36.5 to 42.3)	-13.3 (-20.8 to -4.8)
High SDI	16 400 (15 400 to 17 400)	1.5 (1.4 to 1.6)	-2.6 (-8.0 to 3.3)	45 800 (41 100 to 51 300)	4.4 (3.9 to 4.9)	38.3 (23.8 to 55.8)	414 000 (390 000 to 440 000)	40.3 (37.9 to 42.8)	-9.5 (-14.8 to -3.5)
Central Europe, Eastern Europe, and Central Asia	9 720 (8 770 to 10 700)	2.5 (2.3 to 2.8)	23.9 (10.1 to 37.3)	18 000 (16 200 to 19 900)	4.7 (4.2 to 5.2)	66.8 (48.7 to 86.8)	285 000 (257 000 to 315 000)	75.8 (68.3 to 83.8)	21.1 (7.3 to 34.6)
**Central Asia**	661 (593 to 737)	1.3 (1.2 to 1.5)	6.9 (-8.6 to 27.0)	822 (736 to 920)	1.6 (1.4 to 1.8)	16.7 (-0.1 to 38.4)	20 000 (17 900 to 22 500)	36.7 (33.0 to 41.0)	2.2 (-12.5 to 21.8)
**Central Europe**	3 870 (3 360 to 4 420)	3.2 (2.7 to 3.6)	36.6 (18.3 to 56.7)	6 250 (5 440 to 7 120)	5.3 (4.6 to 6.0)	83.6 (58.9 to 109.3)	111 000 (95 100 to 127 000)	96.4 (82.6 to 110.7)	31.8 (13.7 to 52.4)
**Eastern Europe**	5 190 (4 500 to 5 920)	2.5 (2.1 to 2.8)	23.4 (4.4 to 45.0)	10 900 (9 440 to 12 600)	5.3 (4.6 to 6.1)	73.3 (46.0 to 105.0)	155 000 (134 000 to 177 000)	76.0 (65.8 to 86.9)	23.5 (3.5 to 45.8)
High-income	16 800 (15 800 to 17 800)	1.4 (1.3 to 1.4)	-7.7 (-12.4 to -2.2)	47 100 (42 100 to 52 600)	4.1 (3.6 to 4.6)	34.2 (19.5 to 51.3)	418 000 (396 000 to 442 000)	37.0 (35.1 to 39.1)	-14.8 (-19.2 to -9.3)
**Australasia**	387 (345 to 432)	1.3 (1.2 to 1.5)	-31.1 (-38.9 to -22.5)	682 (528 to 861)	2.4 (1.9 to 3.0)	-10.6 (-30.9 to 14.9)	9 500 (8 490 to 10 600)	34.4 (30.9 to 38.5)	-32.7 (-40.5 to -23.9)
**High-income Asia Pacific**	3 240 (2 930 to 3 500)	1.2 (1.1 to 1.3)	67.4 (55.7 to 79.6)	6 900 (5 810 to 8 190)	2.7 (2.3 to 3.3)	150.7 (110.7 to 200.1)	66 400 (61 400 to 71 700)	27.6 (25.6 to 29.7)	53.7 (43.0 to 65.4)
**High-income North America**	3 510 (3 330 to 3 690)	0.9 (0.9 to 1.0)	-13.8 (-19.1 to -9.0)	15 200 (12 800 to 18 100)	4.2 (3.5 to 5.0)	11.6 (-6.8 to 33.3)	91 200 (86 400 to 96 000)	25.1 (23.8 to 26.4)	-16.0 (-21.6 to -11.0)

© 2023 Cunha AR et al. *JAMA Oncol.*

Location *(Global, **Super-region**, **region**, or SDI Quintile)*	Deaths			Incidence			DALYs		
	**Counts, 2019 (95% UI)**	**Age-standardized rate, 2019 (95% UI)**	**% change of age-standardized rates, 1990-2019 (95% UI)**	**Counts, 2019 (95% UI)**	**Age-standardized rate, 2019 (95% UI)**	**% change of age-standardized rates, 1990-2019 (95% UI)**	**Counts, 2019 (95% UI)**	**Age-standardized rate, 2019 (95% UI)**	**% change of age-standardized rates, 1990-2019 (95% UI)**
**Southern Latin America**	371 (343 to 403)	0.7 (0.7 to 0.8)	-50.3 (-55.4 to -45.6)	504 (393 to 644)	1.0 (0.8 to 1.3)	-39.2 (-53.6 to -22.7)	9 460 (8 700 to 10 300)	18.7 (17.2 to 20.3)	-53.3 (-58.0 to -48.4)
**Western Europe**	9 330 (8 680 to 9 960)	1.9 (1.7 to 2.0)	-9.6 (-15.4 to -2.8)	23 800 (20 400 to 27 900)	5.0 (4.3 to 5.9)	40.1 (19.5 to 65.4)	242 000 (226 000 to 259 000)	52.5 (49.2 to 56.4)	-16.8 (-22.4 to -10.1)
Latin America and Caribbean	5 390 (5 030 to 5 740)	1.4 (1.3 to 1.5)	-18.5 (-24.2 to -12.3)	6 530 (6 060 to 6 970)	1.7 (1.6 to 1.8)	-7.0 (-13.8 to -0.2)	148 000 (138 000 to 158 000)	38.4 (35.9 to 40.9)	-18.6 (-24.4 to -12.4)
**Andean Latin America**	144 (116 to 176)	0.4 (0.3 to 0.5)	-36.2 (-49.4 to -19.5)	165 (132 to 204)	0.5 (0.4 to 0.6)	-27.0 (-42.8 to -7.5)	3 610 (2 850 to 4 530)	9.9 (7.8 to 12.5)	-40.1 (-53.5 to -23.6)
**Caribbean**	579 (489 to 674)	1.8 (1.5 to 2.1)	-20.2 (-32.4 to -6.6)	709 (597 to 830)	2.2 (1.8 to 2.5)	-10.1 (-24.2 to 5.6)	14 600 (12 200 to 17 300)	44.4 (37.2 to 52.6)	-19.3 (-32.1 to -4.9)
**Central Latin America**	742 (624 to 880)	0.5 (0.4 to 0.6)	-27.8 (-39.4 to -14.6)	892 (744 to 1 060)	0.6 (0.5 to 0.7)	-15.8 (-30.0 to -0.6)	18 400 (15 300 to 22 200)	12.0 (10.0 to 14.4)	-28.7 (-40.6 to -14.2)
**Tropical Latin America**	3 920 (3 660 to 4 170)	2.5 (2.3 to 2.7)	-11.9 (-18.4 to -5.6)	4 770 (4 470 to 5 040)	3.0 (2.8 to 3.2)	0.5 (-6.8 to 7.2)	111 000 (104 000 to 119 000)	69.5 (64.9 to 74.0)	-12.3 (-18.9 to -5.9)
North Africa and Middle East	1 410 (1 240 to 1 640)	0.5 (0.4 to 0.6)	-9.8 (-25.4 to 11.2)	1 870 (1 640 to 2 180)	0.6 (0.6 to 0.7)	9.8 (-9.8 to 35.0)	41 000 (35 700 to 47 800)	13.1 (11.5 to 15.3)	-13.1 (-29.0 to 6.7)
South Asia	66 300 (56 600 to 76 900)	7.4 (6.3 to 8.5)	10.5 (-15.0 to 37.3)	71 200 (60 500 to 82 800)	7.7 (6.5 to 8.9)	16.1 (-10.0 to 42.0)	1 930 000 (1 650 000 to 2 240 000)	198.5 (169.4 to 230.0)	8.6 (-16.3 to 35.0)
Southeast Asia, East Asia, and Oceania	12 100 (10 500 to 13 600)	0.7 (0.6 to 0.8)	-8.1 (-23.7 to 7.0)	19 600 (17 200 to 22 000)	1.1 (1.0 to 1.2)	36.5 (14.3 to 59.1)	333 000 (288 000 to 379 000)	18.4 (15.9 to 20.9)	-9.1 (-25.5 to 6.5)
**East Asia**	6 610 (5 530 to 7 790)	0.5 (0.4 to 0.6)	-15.1 (-32.3 to 5.0)	12 500 (10 600 to 14 700)	0.9 (0.8 to 1.1)	46.3 (17.6 to 79.6)	176 000 (147 000 to 208 000)	12.9 (10.8 to 15.1)	-17.4 (-34.4 to 3.1)
**Oceania**	24.5 (18.9 to 33.5)	0.5 (0.4 to 0.7)	5.0 (-14.6 to 31.1)	27.2 (21.1 to 37.2)	0.6 (0.4 to 0.8)	6.7 (-13.0 to 33.5)	776 (595 to 1 080)	14.2 (11.0 to 19.5)	3.0 (-17.2 to 30.2)
**Southeast Asia**	5 440 (4 560 to 6 410)	1.4 (1.2 to 1.6)	1.8 (-17.9 to 22.0)	6 990 (5 820 to 8 370)	1.7 (1.4 to 2.0)	21.0 (-3.6 to 46.7)	156 000 (129 000 to 188 000)	36.3 (30.4 to 43.2)	0.2 (-20.1 to 21.9)
Sub-Saharan Africa	2 480 (2 080 to 2 950)	0.8 (0.7 to 0.9)	4.1 (-12.4 to 22.7)	2 600 (2 170 to 3 100)	0.8 (0.7 to 1.0)	5.8 (-11.0 to 25.1)	76 700 (63 200 to 92 000)	22.1 (18.4 to 26.2)	1.7 (-15.8 to 21.7)
**Central Sub-Saharan Africa**	271 (195 to 365)	0.8 (0.6 to 1.0)	-7.1 (-35.7 to 29.5)	280 (202 to 378)	0.8 (0.5 to 1.0)	-4.9 (-34.7 to 33.4)	8 360 (6 040 to 11 300)	20.6 (14.8 to 27.8)	-7.3 (-37.3 to 31.7)
**Eastern Sub-Saharan Africa**	1 320 (1 050 to 1 620)	1.2 (0.9 to 1.4)	4.0 (-15.0 to 26.1)	1 380 (1 100 to 1 700)	1.2 (0.9 to 1.4)	5.9 (-14.0 to 28.9)	42 200 (33 300 to 52 500)	33.5 (26.6 to 41.4)	3.4 (-16.9 to 27.9)
**Southern Sub-Saharan Africa**	381 (336 to 427)	1.0 (0.9 to 1.1)	8.8 (-3.6 to 22.5)	417 (367 to 471)	1.1 (1.0 to 1.2)	11.6 (-1.5 to 26.8)	11 400 (10 000 to 12 900)	28.5 (25.1 to 32.3)	4.1 (-8.3 to 17.8)
**Western Sub-Saharan Africa**	515 (431 to 614)	0.4 (0.4 to 0.5)	7.2 (-13.0 to 32.7)	527 (438 to 633)	0.4 (0.4 to 0.5)	8.1 (-12.0 to 34.1)	14 800 (12 200 to 17 900)	10.8 (9.0 to 12.9)	2.3 (-18.5 to 28.4)

SDI=Socio-demographic Index; DALYs=disability-adjusted life years; UI=uncertainty interval. See eFigure 3 (p53) and eTable 7 (p54) for details and definitions of the SDI regions.

eTable 10. Deaths, incidence, and DALYs counts and age-standardized rates (per 100 000) for Lip and oral cavity cancer (LOC), both sexes combined, by country or territory, in 2019, and change in age-standardized rates from 1990 to 2019

Super-region, **region**, or country or territory	Deaths, LOC			Incidence, LOC			DALYs, LOC		
	Counts, 2019 (95% UI)	Age-standardized rate, 2019 (95% UI)	% change of age-standardized rates, 1990–2019 (95% UI)	Counts, 2019 (95% UI)	Age-standardized rate, 2019 (95% UI)	% change of age-standardized rates, 1990–2019 (95% UI)	Counts, 2019 (95% UI)	Age-standardized rate, 2019 (95% UI)	% change of age-standardized rates, 1990–2019 (95% UI)
Central Europe, Eastern Europe, and Central Asia									
**Central Asia**									
Armenia	47.1 (39.0 to 55.9)	1.8 (1.5 to 2.1)	31.2 (4.5 to 62.2)	79.9 (65.5 to 95.5)	3.0 (2.5 to 3.6)	35.8 (8.4 to 68.1)	1 190 (978 to 1 430)	45.4 (37.4 to 54.1)	11.7 (-11.0 to 38.2)
Azerbaijan	92.5 (72.2 to 116)	1.7 (1.3 to 2.1)	145.7 (89.7 to 217.8)	156 (123 to 195)	2.6 (2.1 to 3.3)	167.8 (110.3 to 243.8)	2 700 (2 110 to 3 390)	40.9 (32.1 to 51.3)	142.5 (87.1 to 213.8)
Georgia	125 (104 to 148)	3.4 (2.8 to 4.0)	30.0 (2.0 to 65.8)	199 (165 to 238)	5.6 (4.6 to 6.7)	24.2 (-3.8 to 59.3)	3 250 (2 680 to 3 890)	94.3 (78.1 to 113.0)	17.5 (-9.2 to 51.6)
Kazakhstan	393 (337 to 458)	3.6 (3.1 to 4.1)	18.4 (-0.5 to 39.9)	696 (594 to 814)	6.1 (5.2 to 7.1)	31.0 (9.5 to 54.3)	11 000 (9 440 to 13 000)	92.9 (79.6 to 108.9)	13.6 (-5.2 to 35.0)
Kyrgyzstan	73.2 (62.9 to 86.0)	2.5 (2.2 to 2.9)	8.0 (-10.3 to 32.6)	121 (102 to 142)	3.9 (3.4 to 4.6)	15.5 (-5.2 to 43.3)	2 100 (1 790 to 2 480)	64.2 (55.0 to 75.4)	3.7 (-14.8 to 29.6)
Mongolia	53.2 (40.5 to 69.7)	3.6 (2.8 to 4.6)	7.9 (-21.0 to 48.9)	84.3 (64.2 to 110)	5.4 (4.2 to 6.9)	21.4 (-10.4 to 66.8)	1 620 (1 210 to 2 160)	93.7 (71.8 to 123.4)	20.8 (-12.9 to 69.3)
Tajikistan	45.0 (35.9 to 56.5)	1.6 (1.3 to 2.0)	72.0 (18.3 to 146.1)	71.3 (56.4 to 89.5)	2.3 (1.9 to 2.9)	87.2 (31.9 to 161.1)	1 340 (1 060 to 1 690)	37.4 (30.1 to 46.7)	91.9 (41.7 to 158.4)
Turkmenistan	87.6 (69.5 to 111)	3.4 (2.7 to 4.3)	109.4 (65.3 to 165.9)	151 (119 to 190)	5.6 (4.5 to 7.1)	130.9 (80.2 to 195.2)	2 730 (2 150 to 3 480)	95.5 (75.6 to 121.4)	113.2 (66.5 to 173.7)
Uzbekistan	454 (378 to 543)	3.4 (2.9 to 4.0)	213.9 (120.1 to 321.5)	820 (674 to 980)	5.5 (4.6 to 6.4)	244.0 (145.2 to 361.7)	15 000 (12 400 to 18 000)	89.7 (75.2 to 106.7)	228.2 (137.5 to 336.4)
**Central Europe**									
Albania	52.3 (39.4 to 68.5)	2.0 (1.5 to 2.6)	32.2 (-1.6 to 73.6)	92.6 (69.0 to 122)	3.6 (2.7 to 4.7)	48.3 (8.0 to 95.1)	1 140 (840 to 1 510)	45.4 (33.7 to 60.1)	9.2 (-20.6 to 45.6)
Bosnia and Herzegovina	97.5 (75.7 to 122)	2.7 (2.1 to 3.4)	39.2 (8.4 to 75.1)	171 (132 to 216)	4.7 (3.6 to 5.9)	51.1 (15.8 to 91.9)	2 330 (1 800 to 2 970)	65.3 (50.3 to 83.4)	19.5 (-8.1 to 53.5)
Bulgaria	289 (229 to 360)	3.6 (2.8 to 4.5)	50.7 (18.9 to 92.6)	648 (507 to 823)	8.3 (6.4 to 10.5)	54.6 (19.8 to 98.9)	7 810 (6 090 to 9 910)	106.5 (82.5 to 135.3)	37.2 (5.5 to 77.1)
Croatia	180 (138 to 227)	3.5 (2.7 to 4.5)	-28.7 (-45.8 to -8.3)	443 (344 to 565)	8.9 (6.9 to 11.5)	-18.8 (-38.4 to 5.0)	4 480 (3 440 to 5 770)	96.1 (73.3 to 124.1)	-39.3 (-54.7 to -20.4)
Czechia	413 (335 to 503)	3.4 (2.7 to 4.2)	15.3 (-6.6 to 41.4)	812 (655 to 992)	6.8 (5.5 to 8.4)	33.4 (7.2 to 63.9)	10 300 (8 270 to 12 700)	92.8 (73.8 to 114.4)	0.7 (-19.9 to 24.9)
Hungary	775 (632 to 942)	7.2 (5.8 to 8.8)	9.8 (-10.7 to 33.5)	1 550 (1 250 to 1 890)	14.8 (11.8 to 18.1)	22.0 (-0.6 to 50.0)	21 300 (17 200 to 25 900)	213.0 (171.1 to 262.1)	-1.4 (-21.0 to 21.5)
Montenegro	20.3 (16.2 to 24.8)	3.4 (2.7 to 4.1)	56.2 (18.6 to 99.6)	40.1 (31.8 to 49.1)	6.7 (5.3 to 8.2)	62.3 (21.1 to 108.9)	553 (436 to 684)	94.7 (74.8 to 117.3)	40.4 (5.5 to 80.3)

Super-region, **region,** or country or territory	Deaths, LOC			Incidence, LOC			DALYs, LOC		
	**Counts, 2019 (95% UI)**	**Age-standardized rate, 2019 (95% UI)**	**% change of age-standardized rates, 1990–2019 (95% UI)**	**Counts, 2019 (95% UI)**	**Age-standardized rate, 2019 (95% UI)**	**% change of age-standardized rates, 1990–2019 (95% UI)**	**Counts, 2019 (95% UI)**	**Age-standardized rate, 2019 (95% UI)**	**% change of age-standardized rates, 1990–2019 (95% UI)**
North Macedonia	48.8 (38.6 to 61.1)	2.7 (2.1 to 3.3)	57.9 (21.8 to 98.9)	87.9 (68.6 to 112)	4.5 (3.6 to 5.7)	76.3 (34.6 to 124.4)	1 190 (929 to 1 520)	60.9 (47.8 to 77.2)	43.1 (9.0 to 83.8)
Poland	1 810 (1 490 to 2 180)	4.3 (3.6 to 5.2)	60.8 (33.1 to 93.8)	2 880 (2 400 to 3 490)	7.0 (5.8 to 8.5)	82.2 (50.1 to 120.9)	45 700 (37 500 to 55 600)	117.4 (95.5 to 143.0)	45.9 (18.1 to 77.9)
Romania	1 120 (910 to 1 370)	5.4 (4.3 to 6.6)	84.7 (49.6 to 126.6)	2 100 (1 690 to 2 610)	10.3 (8.3 to 12.8)	107.2 (65.4 to 160.6)	31 600 (25 400 to 39 100)	162.8 (130.2 to 202.2)	72.7 (37.5 to 115.8)
Serbia	388 (308 to 485)	4.2 (3.3 to 5.3)	19.7 (-10.0 to 55.2)	693 (544 to 872)	7.6 (5.9 to 9.6)	32.6 (-0.6 to 75.0)	9 720 (7 530 to 12 300)	111.1 (85.8 to 141.0)	4.4 (-22.7 to 38.0)
Slovakia	296 (222 to 381)	5.3 (4.0 to 6.9)	3.4 (-23.8 to 36.2)	625 (469 to 810)	11.3 (8.5 to 14.7)	14.5 (-15.5 to 51.6)	8 360 (6 160 to 10 900)	157.3 (115.3 to 205.9)	-5.2 (-31.3 to 27.2)
Slovenia	64.1 (49.3 to 84.2)	2.5 (1.9 to 3.3)	-8.0 (-37.3 to 32.3)	174 (130 to 230)	7.3 (5.5 to 9.7)	0.6 (-33.5 to 47.8)	1 550 (1 180 to 2 050)	67.6 (51.5 to 89.0)	-24.5 (-50.1 to 11.0)
**Eastern Europe**									
Belarus	462 (352 to 605)	4.7 (3.6 to 6.2)	-16.6 (-37.3 to 9.8)	676 (511 to 887)	7.0 (5.3 to 9.2)	-10.3 (-32.5 to 17.5)	13 100 (9 820 to 17 200)	138.7 (104.1 to 182.7)	-18.6 (-39.1 to 8.2)
Estonia	50.8 (39.5 to 64.8)	3.3 (2.5 to 4.2)	4.1 (-19.9 to 33.1)	105 (81.5 to 135)	7.2 (5.5 to 9.4)	15.1 (-12.0 to 50.0)	1 210 (926 to 1 570)	87.6 (66.9 to 114.4)	-14.6 (-35.4 to 12.6)
Latvia	92.2 (75.7 to 115)	4.1 (3.3 to 5.1)	-0.2 (-20.2 to 25.6)	137 (113 to 173)	6.3 (5.1 to 8.0)	7.2 (-13.8 to 36.4)	2 320 (1 840 to 2 950)	114.1 (89.7 to 145.4)	-15.1 (-33.9 to 8.7)
Lithuania	134 (107 to 165)	4.1 (3.3 to 5.2)	-3.9 (-23.1 to 17.7)	178 (142 to 221)	5.7 (4.5 to 7.1)	1.2 (-19.7 to 24.8)	3 510 (2 760 to 4 410)	119.4 (93.7 to 150.3)	-15.1 (-33.5 to 5.1)
Republic of Moldova	129 (111 to 151)	3.5 (3.0 to 4.1)	0.8 (-14.8 to 18.9)	174 (149 to 203)	4.8 (4.1 to 5.6)	2.2 (-14.1 to 21.4)	3 670 (3 140 to 4 310)	104.0 (88.7 to 121.6)	-7.3 (-22.5 to 9.7)
Russian Federation	5 850 (4 950 to 6 850)	4.1 (3.4 to 4.8)	30.1 (10.5 to 51.6)	7 360 (6 360 to 8 470)	5.2 (4.5 to 6.0)	38.7 (20.4 to 60.4)	163 000 (136 000 to 192 000)	118.1 (98.8 to 139.2)	25.4 (5.6 to 47.4)
Ukraine	2 500 (2 040 to 3 050)	5.6 (4.5 to 6.8)	55.3 (20.3 to 102.9)	4 310 (3 540 to 5 250)	9.7 (7.9 to 11.8)	69.4 (30.1 to 115.7)	74 900 (60 500 to 92 000)	175.3 (141.3 to 215.7)	57.9 (20.7 to 110.0)
High-income									
**Australasia**									
Australia	902 (811 to 979)	3.5 (3.1 to 3.7)	17.2 (7.4 to 26.7)	1 490 (1 140 to 1 890)	6.1 (4.7 to 7.8)	15.5 (-10.8 to 47.6)	19 500 (18 000 to 21 000)	83.2 (77.0 to 89.6)	0.9 (-7.3 to 9.2)
New Zealand	93.7 (84.7 to 102)	1.9 (1.7 to 2.1)	39.6 (26.1 to 54.2)	347 (283 to 424)	7.6 (6.2 to 9.4)	48.8 (18.9 to 83.4)	2 120 (1 940 to 2 300)	48.0 (44.1 to 51.9)	26.4 (15.0 to 39.0)
**High-income Asia Pacific**									
Brunei Darussalam	9.66 (8.39 to 11.2)	6.0 (5.2 to 6.8)	122.8 (80.6 to 179.7)	16.4 (14.2 to 19.1)	8.6 (7.5 to 9.8)	139.4 (92.7 to 204.2)	280 (241 to 326)	129.7 (113.2 to 149.5)	112.8 (71.6 to 166.6)
Japan	4 680 (3 880 to 5 110)	1.9 (1.7 to 2.1)	164.5 (130.0 to 184.8)	6 930 (5 680 to 8 060)	3.5 (3.0 to 4.1)	127.0 (90.8 to 163.9)	76 900 (68 200 to 81 800)	43.5 (40.3 to 45.6)	77.5 (62.0 to 87.3)
Republic of Korea	796 (709 to 887)	1.4 (1.3 to 1.6)	195.0 (160.1 to 236.2)	1 400 (1 150 to 1 680)	2.5 (2.1 to 3.0)	234.7 (172.1 to 311.6)	17 800 (16 000 to 20 000)	32.4 (29.2 to 36.2)	128.7 (101.9 to 161.0)

© 2023 Cunha AR et al. *JAMA Oncol*.

Super-region, **region,** or country or territory	**Deaths, LOC**			**Incidence, LOC**			**DALYs, LOC**		
	**Counts, 2019 (95% UI)**	**Age-standardized rate, 2019 (95% UI)**	**% change of age-standardized rates, 1990–2019 (95% UI)**	**Counts, 2019 (95% UI)**	**Age-standardized rate, 2019 (95% UI)**	**% change of age-standardized rates, 1990–2019 (95% UI)**	**Counts, 2019 (95% UI)**	**Age-standardized rate, 2019 (95% UI)**	**% change of age-standardized rates, 1990–2019 (95% UI)**
Singapore	67.5 (60.6 to 75.0)	1.4 (1.2 to 1.5)	73.9 (54.8 to 98.0)	121 (96.2 to 152)	2.4 (1.9 to 3.1)	86.3 (45.7 to 135.9)	1 580 (1 430 to 1 760)	31.2 (28.2 to 34.5)	45.8 (29.5 to 67.2)
**High-income North America**									
Canada	1 070 (973 to 1 160)	2.5 (2.2 to 2.7)	24.6 (14.6 to 35.3)	2 420 (1 890 to 3 120)	5.9 (4.6 to 7.6)	26.6 (-2.0 to 65.5)	22 900 (20 900 to 24 800)	57.8 (53.1 to 62.5)	7.1 (-1.6 to 16.8)
Greenland	2.47 (1.98 to 2.95)	5.7 (4.7 to 6.8)	19.2 (-7.9 to 47.5)	4.44 (3.61 to 5.33)	9.8 (8.0 to 11.6)	27.4 (-1.0 to 58.4)	63.7 (50.5 to 77.1)	133.9 (107.3 to 160.9)	9.5 (-17.4 to 38.6)
United States of America	7 810 (7 370 to 8 130)	2.2 (2.1 to 2.3)	31.1 (25.9 to 35.5)	30 700 (26 400 to 35 800)	9.2 (7.9 to 10.7)	43.2 (22.3 to 66.8)	180 000 (172 000 to 188 000)	55.1 (52.5 to 57.3)	20.6 (15.8 to 24.9)
**Southern Latin America**									
Argentina	843 (778 to 907)	2.5 (2.3 to 2.7)	54.3 (41.2 to 68.6)	1 520 (1 190 to 1 920)	4.6 (3.6 to 5.8)	64.6 (28.8 to 109.1)	20 600 (19 000 to 22 400)	63.1 (58.2 to 68.7)	38.5 (26.4 to 52.8)
Chile	210 (190 to 228)	1.4 (1.3 to 1.5)	95.1 (75.7 to 114.3)	397 (305 to 503)	2.6 (2.0 to 3.3)	112.8 (62.2 to 173.8)	4 690 (4 290 to 5 110)	31.2 (28.6 to 34.0)	66.8 (49.6 to 85.4)
Uruguay	118 (107 to 129)	3.6 (3.2 to 3.9)	17.0 (5.9 to 31.5)	212 (164 to 270)	6.8 (5.2 to 8.7)	19.0 (-9.3 to 52.4)	2 750 (2 480 to 3 010)	92.0 (83.3 to 101.0)	4.9 (-6.8 to 18.4)
**Western Europe**									
Andorra	2.13 (1.63 to 2.75)	2.4 (1.8 to 3.1)	82.4 (23.8 to 162.5)	6.50 (4.90 to 8.54)	7.4 (5.6 to 9.8)	70.5 (13.2 to 152.4)	57.0 (42.3 to 75.6)	65.0 (48.1 to 86.6)	64.5 (8.6 to 142.3)
Austria	300 (276 to 325)	2.8 (2.6 to 3.1)	38.5 (27.1 to 51.7)	671 (534 to 832)	6.8 (5.4 to 8.4)	51.8 (20.4 to 89.4)	7 140 (6 580 to 7 740)	74.9 (68.8 to 81.1)	18.7 (7.9 to 30.6)
Belgium	399 (365 to 436)	3.0 (2.7 to 3.2)	42.5 (30.7 to 56.7)	1 130 (857 to 1 440)	9.1 (6.9 to 11.6)	40.5 (6.5 to 82.9)	9 600 (8 770 to 10 500)	80.4 (73.3 to 88.1)	25.7 (14.0 to 40.5)
Cyprus	19.9 (17.2 to 22.8)	1.7 (1.5 to 2.0)	116.0 (80.7 to 163.0)	56.2 (47.8 to 65.9)	4.8 (4.0 to 5.6)	148.0 (101.3 to 210.1)	468 (402 to 537)	40.4 (34.7 to 46.3)	97.0 (62.4 to 143.7)
Denmark	195 (175 to 214)	2.8 (2.5 to 3.0)	168.7 (139.4 to 199.6)	530 (406 to 684)	8.0 (6.1 to 10.4)	178.4 (112.0 to 259.9)	4 330 (3 920 to 4 780)	68.9 (62.1 to 75.8)	150.0 (121.1 to 181.6)
Finland	157 (141 to 170)	2.0 (1.9 to 2.2)	97.5 (79.8 to 118.3)	408 (318 to 513)	5.9 (4.6 to 7.4)	93.8 (51.6 to 146.9)	3 130 (2 840 to 3 410)	48.2 (44.2 to 52.4)	64.3 (48.8 to 83.2)
France	2 420 (2 190 to 2 640)	3.1 (2.8 to 3.3)	-19.8 (-27.0 to -12.1)	6 730 (5 190 to 8 690)	9.4 (7.1 to 12.2)	-21.8 (-40.6 to 2.0)	57 200 (51 800 to 63 000)	83.8 (76.1 to 93.0)	-32.1 (-38.6 to -24.5)
Germany	3 020 (2 780 to 3 250)	2.7 (2.5 to 3.0)	13.2 (4.2 to 22.3)	7 510 (5 810 to 9 740)	7.4 (5.7 to 9.7)	11.6 (-14.9 to 45.6)	71 100 (65 300 to 77 200)	73.4 (67.2 to 79.7)	-8.5 (-17.0 to 0.1)
Greece	268 (243 to 288)	1.8 (1.6 to 1.9)	72.3 (58.4 to 85.6)	648 (506 to 809)	4.9 (3.8 to 6.2)	55.7 (21.6 to 95.2)	5 260 (4 840 to 5 690)	42.8 (39.5 to 46.5)	49.0 (36.4 to 61.8)
Iceland	6.88 (6.04 to 7.71)	2.0 (1.8 to 2.2)	50.8 (31.2 to 72.7)	20.7 (17.8 to 23.8)	6.5 (5.6 to 7.5)	49.1 (25.6 to 79.0)	163 (145 to 183)	52.5 (46.6 to 58.9)	38.3 (20.2 to 58.0)
Ireland	106 (95.2 to 117)	2.3 (2.0 to 2.5)	22.3 (8.9 to 37.6)	302 (229 to 389)	6.6 (5.0 to 8.6)	31.4 (-3.2 to 71.4)	2 350 (2 110 to 2 610)	52.5 (47.2 to 58.3)	14.4 (0.8 to 29.5)

Super-region, **region,** or country or territory	**Deaths, LOC**			**Incidence, LOC**			**DALYs, LOC**		
	**Counts, 2019 (95% UI)**	**Age-standardized rate, 2019 (95% UI)**	**% change of age-standardized rates, 1990–2019 (95% UI)**	**Counts, 2019 (95% UI)**	**Age-standardized rate, 2019 (95% UI)**	**% change of age-standardized rates, 1990–2019 (95% UI)**	**Counts, 2019 (95% UI)**	**Age-standardized rate, 2019 (95% UI)**	**% change of age-standardized rates, 1990–2019 (95% UI)**
Israel	105 (93.7 to 114)	1.4 (1.3 to 1.5)	168.5 (143.0 to 196.7)	256 (199 to 325)	3.6 (2.8 to 4.6)	179.4 (113.3 to 260.4)	2 190 (1 990 to 2 390)	31.9 (29.0 to 34.9)	141.9 (117.6 to 168.0)
Italy	2 060 (1 850 to 2 180)	2.3 (2.1 to 2.4)	0.7 (-7.3 to 6.2)	4 340 (3 530 to 5 220)	5.5 (4.4 to 6.7)	0.3 (-18.6 to 21.9)	41 600 (38 600 to 43 600)	56.1 (52.9 to 58.8)	-19.3 (-24.0 to -15.6)
Luxembourg	14.9 (13.0 to 17.0)	2.4 (2.1 to 2.7)	9.1 (-5.8 to 26.7)	42.9 (34.7 to 52.1)	7.2 (5.8 to 8.8)	9.9 (-12.2 to 35.3)	375 (325 to 431)	64.1 (55.7 to 73.6)	-1.6 (-15.8 to 14.6)
Malta	9.99 (8.69 to 11.3)	1.8 (1.6 to 2.1)	50.9 (29.1 to 76.9)	27.9 (23.1 to 33.3)	5.5 (4.6 to 6.6)	56.6 (26.5 to 93.2)	233 (203 to 265)	48.8 (42.5 to 55.6)	32.8 (12.2 to 56.4)
Monaco	0.690 (0.550 to 0.826)	1.2 (1.0 to 1.5)	40.0 (6.7 to 84.7)	1.91 (1.47 to 2.32)	3.9 (3.0 to 4.8)	35.6 (-1.0 to 84.1)	15.1 (11.8 to 18.6)	33.1 (25.2 to 41.6)	33.1 (-3.2 to 81.4)
Netherlands	479 (436 to 521)	2.3 (2.1 to 2.4)	78.8 (64.2 to 94.8)	1 360 (1 060 to 1 710)	6.8 (5.3 to 8.6)	75.3 (34.6 to 122.1)	10 400 (9 530 to 11 400)	54.0 (49.4 to 59.0)	57.1 (42.6 to 72.9)
Norway	107 (97.2 to 113)	1.7 (1.6 to 1.8)	1.9 (-3.8 to 8.9)	289 (243 to 341)	5.1 (4.3 to 6.0)	1.2 (-14.9 to 21.6)	2 210 (2 070 to 2 350)	40.2 (37.8 to 42.7)	-6.8 (-11.5 to -0.3)
Portugal	445 (407 to 483)	3.3 (3.0 to 3.6)	32.6 (20.2 to 45.6)	1 370 (1 050 to 1 760)	10.8 (8.1 to 14.0)	64.7 (24.6 to 113.1)	11 100 (10 000 to 12 200)	95.0 (85.7 to 105.1)	21.8 (9.6 to 36.1)
San Marino	1.01 (0.676 to 1.45)	2.5 (1.6 to 3.7)	82.4 (18.3 to 168.5)	2.71 (2.03 to 3.60)	7.6 (5.6 to 10.1)	61.7 (16.4 to 126.0)	22.3 (14.3 to 33.2)	65.0 (40.8 to 97.9)	59.3 (-2.4 to 144.7)
Spain	1 580 (1 430 to 1 700)	2.7 (2.4 to 2.9)	25.6 (14.8 to 35.7)	6 470 (4 990 to 8 190)	12.0 (9.2 to 15.3)	28.6 (-1.5 to 64.8)	36 000 (32 700 to 39 300)	69.9 (63.5 to 76.6)	1.8 (-7.9 to 11.3)
Sweden	239 (215 to 258)	1.7 (1.6 to 1.9)	29.8 (19.8 to 41.1)	609 (511 to 707)	4.9 (4.1 to 5.7)	21.9 (2.0 to 44.5)	4 430 (4 090 to 4 760)	37.6 (34.7 to 40.4)	12.7 (3.8 to 22.9)
Switzerland	255 (228 to 279)	2.4 (2.1 to 2.6)	108.9 (88.8 to 129.6)	715 (549 to 923)	7.2 (5.5 to 9.4)	97.3 (49.3 to 160.1)	5 650 (5 110 to 6 200)	59.4 (53.9 to 65.5)	82.2 (63.4 to 101.7)
United Kingdom	2 190 (2 060 to 2 290)	2.9 (2.7 to 3.0)	46.3 (40.7 to 52.0)	7 020 (5 790 to 8 470)	10.0 (8.2 to 12.2)	76.3 (45.8 to 112.8)	49 300 (47 100 to 51 400)	72.4 (69.5 to 75.4)	39.1 (34.4 to 44.2)
Latin America and Caribbean									
**Andean Latin America**									
Bolivia (Plurinational State of)	113 (80.6 to 149)	2.1 (1.5 to 2.8)	171.0 (100.4 to 259.6)	172 (124 to 226)	3.1 (2.2 to 4.0)	203.7 (123.6 to 305.9)	2 860 (2 060 to 3 810)	48.9 (35.3 to 64.5)	144.1 (74.6 to 233.2)
Ecuador	147 (116 to 186)	1.6 (1.3 to 2.1)	236.5 (163.1 to 327.6)	240 (188 to 308)	2.5 (2.0 to 3.2)	278.9 (192.6 to 388.5)	3 440 (2 690 to 4 440)	35.3 (27.7 to 45.5)	191.9 (123.7 to 278.5)
Peru	259 (195 to 340)	1.3 (1.0 to 1.7)	111.1 (52.6 to 197.6)	441 (327 to 586)	2.2 (1.6 to 2.9)	142.2 (71.2 to 243.9)	6 200 (4 600 to 8 320)	29.9 (22.2 to 40.2)	83.5 (29.4 to 161.0)
**Caribbean**									
Antigua and Barbuda	1.70 (1.43 to 1.98)	2.7 (2.3 to 3.2)	60.1 (32.2 to 91.5)	2.90 (2.42 to 3.42)	4.5 (3.8 to 5.3)	74.0 (42.5 to 110.6)	41.5 (34.3 to 49.0)	62.4 (51.8 to 73.3)	67.1 (35.6 to 103.9)
Bahamas	11.2 (9.10 to 13.8)	4.6 (3.7 to 5.6)	98.9 (56.8 to 148.3)	19.3 (15.5 to 24.0)	7.6 (6.1 to 9.3)	103.3 (58.3 to 155.1)	311 (248 to 390)	116.6 (93.3 to 145.2)	92.2 (47.6 to 144.6)

Super-region, **region,** or country or territory	Deaths, LOC			Incidence, LOC			DALYs, LOC		
	**Counts, 2019 (95% UI)**	**Age-standardized rate, 2019 (95% UI)**	**% change of age-standardized rates, 1990–2019 (95% UI)**	**Counts, 2019 (95% UI)**	**Age-standardized rate, 2019 (95% UI)**	**% change of age-standardized rates, 1990–2019 (95% UI)**	**Counts, 2019 (95% UI)**	**Age-standardized rate, 2019 (95% UI)**	**% change of age-standardized rates, 1990–2019 (95% UI)**
Barbados	10.0 (8.26 to 11.9)	3.2 (2.7 to 3.9)	55.0 (26.1 to 85.1)	16.7 (13.8 to 20.1)	5.5 (4.5 to 6.6)	65.7 (33.0 to 101.3)	225 (183 to 270)	75.0 (61.3 to 90.2)	56.4 (24.6 to 89.9)
Belize	3.73 (3.16 to 4.30)	2.1 (1.8 to 2.5)	239.3 (185.2 to 298.3)	6.22 (5.26 to 7.23)	3.4 (2.9 to 3.9)	280.4 (218.6 to 355.6)	104 (87.4 to 121)	53.7 (45.2 to 62.3)	288.6 (223.4 to 362.8)
Bermuda	2.76 (2.32 to 3.31)	3.3 (2.8 to 4.0)	18.1 (-3.4 to 46.2)	5.21 (4.33 to 6.31)	6.5 (5.4 to 7.9)	27.4 (2.9 to 59.3)	58.4 (48.3 to 71.4)	76.1 (62.4 to 94.1)	1.1 (-18.4 to 26.9)
Cuba	568 (461 to 694)	4.7 (3.8 to 5.7)	97.4 (60.0 to 140.4)	1 030 (824 to 1 290)	8.7 (6.9 to 10.8)	109.1 (66.1 to 160.6)	13 300 (10 600 to 16 500)	113.5 (90.9 to 141.3)	91.7 (52.4 to 139.1)
Dominica	2.84 (2.28 to 3.49)	5.0 (4.0 to 6.1)	22.7 (-1.6 to 54.1)	4.25 (3.39 to 5.26)	7.5 (6.0 to 9.3)	22.7 (-1.9 to 55.9)	65.1 (51.6 to 81.0)	116.6 (92.7 to 145.1)	26.2 (-1.6 to 62.7)
Dominican Republic	257 (188 to 345)	4.5 (3.4 to 6.1)	235.7 (134.6 to 380.3)	397 (286 to 540)	6.8 (4.9 to 9.2)	264.3 (151.3 to 430.0)	6 120 (4 320 to 8 390)	101.6 (72.1 to 138.6)	219.7 (116.2 to 371.3)
Grenada	3.08 (2.69 to 3.49)	4.4 (3.8 to 4.9)	26.1 (4.7 to 48.9)	5.11 (4.42 to 5.85)	7.0 (6.1 to 8.0)	43.7 (18.8 to 71.0)	80.7 (69.4 to 93.2)	108.0 (93.4 to 123.9)	41.9 (17.7 to 68.1)
Guyana	10.4 (7.89 to 13.1)	2.6 (2.0 to 3.3)	43.8 (6.3 to 89.7)	16.4 (12.4 to 21.0)	4.0 (3.0 to 5.0)	52.2 (11.8 to 102.8)	297 (222 to 386)	68.0 (51.2 to 87.1)	45.5 (5.5 to 96.0)
Haiti	151 (90.6 to 230)	3.6 (2.2 to 5.4)	74.4 (26.0 to 151.5)	210 (127 to 317)	4.6 (2.8 to 7.0)	83.0 (29.5 to 163.5)	4 270 (2 560 to 6 500)	86.9 (51.9 to 132.1)	70.5 (18.0 to 149.9)
Jamaica	29.2 (23.0 to 36.4)	1.5 (1.2 to 1.9)	16.7 (-8.8 to 47.7)	48.6 (37.4 to 61.3)	2.6 (2.0 to 3.2)	22.2 (-5.7 to 57.3)	711 (548 to 898)	37.6 (29.0 to 47.6)	18.6 (-9.4 to 52.5)
Puerto Rico	94.7 (72.8 to 121)	2.1 (1.6 to 2.7)	-16.0 (-35.0 to 7.3)	173 (133 to 223)	4.2 (3.2 to 5.5)	-12.3 (-33.0 to 13.6)	2 030 (1 540 to 2 640)	52.6 (39.5 to 68.7)	-26.8 (-44.7 to -4.5)
Saint Kitts and Nevis	1.54 (1.26 to 1.82)	3.8 (3.2 to 4.4)	34.8 (9.3 to 64.4)	2.80 (2.24 to 3.38)	6.5 (5.3 to 7.7)	53.3 (20.7 to 91.3)	41.0 (31.9 to 49.8)	89.7 (71.2 to 108.0)	53.8 (17.7 to 94.5)
Saint Lucia	6.02 (5.05 to 7.12)	4.5 (3.7 to 5.3)	96.8 (60.8 to 137.3)	9.90 (8.25 to 11.8)	7.2 (6.0 to 8.6)	111.4 (71.7 to 158.6)	151 (124 to 182)	108.6 (89.8 to 130.3)	96.7 (58.1 to 140.9)
Saint Vincent and the Grenadines	5.23 (4.55 to 6.04)	6.2 (5.4 to 7.1)	88.2 (59.5 to 122.3)	8.24 (7.08 to 9.52)	9.6 (8.3 to 11.1)	92.6 (60.7 to 129.6)	133 (114 to 155)	153.1 (131.6 to 177.9)	93.7 (60.9 to 132.2)
Suriname	8.66 (7.00 to 10.5)	2.3 (1.9 to 2.8)	127.7 (82.8 to 184.5)	13.6 (10.8 to 16.7)	3.5 (2.8 to 4.3)	141.5 (90.5 to 207.9)	226 (180 to 282)	57.0 (45.6 to 70.5)	125.3 (77.1 to 187.5)
Trinidad and Tobago	22.8 (17.1 to 29.5)	2.0 (1.5 to 2.5)	49.8 (11.9 to 96.5)	37.9 (28.3 to 50.1)	3.3 (2.4 to 4.3)	56.6 (15.9 to 108.2)	581 (428 to 774)	50.0 (36.7 to 66.5)	42.5 (4.4 to 91.7)
United States Virgin Islands	4.25 (3.47 to 5.03)	3.7 (3.0 to 4.4)	160.5 (100.4 to 241.2)	7.26 (5.81 to 8.74)	6.3 (5.1 to 7.6)	163.4 (101.1 to 247.2)	103 (81.1 to 124)	91.9 (72.7 to 112.1)	126.1 (70.8 to 202.1)
**Central Latin America**									
Colombia	547 (425 to 693)	1.6 (1.3 to 2.0)	91.9 (50.5 to 142.5)	931 (717 to 1 190)	2.8 (2.1 to 3.6)	108.1 (60.1 to 165.1)	11 700 (8 980 to 15 100)	35.2 (27.0 to 45.2)	59.6 (22.7 to 104.3)
Costa Rica	60.3 (46.6 to 76.1)	1.9 (1.4 to 2.3)	123.2 (73.2 to 183.6)	110 (84.2 to 139)	3.4 (2.6 to 4.3)	127.9 (73.6 to 190.8)	1 410 (1 070 to 1 800)	42.9 (32.5 to 54.8)	97.0 (51.9 to 153.5)
El Salvador	65.2 (49.9 to 83.7)	1.7 (1.3 to 2.2)	65.8 (23.3 to 116.1)	107 (80.2 to 138)	2.8 (2.1 to 3.7)	84.9 (36.9 to 141.8)	1 490 (1 100 to 1 930)	40.0 (29.5 to 51.9)	45.9 (5.3 to 93.9)

Super-region, **region,** or country or territory	**Deaths, LOC**			**Incidence, LOC**			**DALYs, LOC**		
	**Counts, 2019 (95% UI)**	**Age-standardized rate, 2019 (95% UI)**	**% change of age-standardized rates, 1990–2019 (95% UI)**	**Counts, 2019 (95% UI)**	**Age-standardized rate, 2019 (95% UI)**	**% change of age-standardized rates, 1990–2019 (95% UI)**	**Counts, 2019 (95% UI)**	**Age-standardized rate, 2019 (95% UI)**	**% change of age-standardized rates, 1990–2019 (95% UI)**
Guatemala	107 (85.4 to 133)	1.6 (1.3 to 2.0)	160.5 (103.8 to 236.4)	165 (130 to 206)	2.3 (1.8 to 2.9)	186.1 (118.0 to 268.8)	2 600 (2 030 to 3 260)	34.9 (27.6 to 43.7)	130.1 (75.9 to 202.7)
Honduras	89.0 (62.0 to 126)	2.5 (1.7 to 3.4)	271.4 (164.8 to 427.0)	141 (99.1 to 199)	3.6 (2.5 to 5.1)	302.4 (190.8 to 465.5)	2 290 (1 610 to 3 250)	56.4 (39.4 to 79.4)	239.8 (143.7 to 376.3)
Mexico	1 100 (953 to 1 260)	1.5 (1.3 to 1.7)	144.4 (111.5 to 180.0)	1 890 (1 630 to 2 170)	2.6 (2.2 to 2.9)	174.2 (136.9 to 214.4)	26 900 (23 000 to 30 800)	35.3 (30.2 to 40.3)	132.9 (100.0 to 169.2)
Nicaragua	37.3 (30.5 to 44.7)	1.5 (1.2 to 1.7)	198.1 (133.0 to 289.8)	63.2 (51.0 to 76.5)	2.3 (1.9 to 2.8)	239.8 (167.4 to 334.9)	894 (714 to 1 100)	31.0 (24.9 to 37.6)	175.6 (113.5 to 252.9)
Panama	46.9 (36.4 to 59.9)	1.8 (1.4 to 2.3)	71.0 (30.1 to 119.8)	80.3 (61.7 to 103)	3.1 (2.4 to 3.9)	85.5 (40.0 to 143.4)	1 070 (810 to 1 390)	40.6 (30.8 to 52.6)	64.1 (23.0 to 115.4)
Venezuela (Bolivarian Republic of)	413 (310 to 529)	2.3 (1.7 to 2.9)	189.2 (116.7 to 272.6)	719 (533 to 927)	3.9 (2.9 to 5.0)	220.1 (139.4 to 318.4)	10 200 (7 560 to 13 300)	54.2 (40.2 to 70.3)	173.7 (100.3 to 257.9)
**Tropical Latin America**									
Brazil	5 550 (5 190 to 5 870)	3.7 (3.4 to 3.9)	129.8 (114.9 to 145.6)	9 540 (8 960 to 10 000)	6.3 (5.9 to 6.6)	147.0 (131.1 to 163.4)	148 000 (140 000 to 156 000)	95.6 (90.4 to 101.0)	110.1 (96.9 to 124.2)
Paraguay	89.7 (65.9 to 117)	2.5 (1.9 to 3.3)	167.0 (90.9 to 266.5)	152 (111 to 201)	4.2 (3.1 to 5.5)	195.2 (107.1 to 312.9)	2 410 (1 740 to 3 200)	65.0 (46.9 to 85.9)	165.2 (85.8 to 268.3)
North Africa and Middle East									
**North Africa and Middle East**									
Afghanistan	137 (82.3 to 190)	1.7 (1.1 to 2.3)	56.5 (7.6 to 123.8)	204 (120 to 284)	2.3 (1.4 to 3.1)	76.7 (20.1 to 155.2)	4 510 (2 610 to 6 450)	43.7 (26.2 to 60.1)	79.4 (20.8 to 165.2)
Algeria	259 (203 to 323)	1.3 (1.0 to 1.6)	122.5 (61.5 to 206.0)	484 (380 to 607)	2.2 (1.7 to 2.7)	151.8 (80.4 to 243.0)	7 240 (5 610 to 9 130)	31.1 (24.2 to 38.9)	108.1 (48.6 to 189.8)
Bahrain	9.96 (7.34 to 13.0)	1.8 (1.4 to 2.3)	200.4 (107.1 to 334.1)	21.2 (15.6 to 27.9)	3.2 (2.4 to 4.0)	267.5 (155.9 to 428.5)	309 (225 to 407)	39.2 (29.6 to 50.2)	201.4 (104.4 to 337.8)
Egypt	407 (285 to 569)	1.1 (0.8 to 1.5)	148.6 (68.9 to 259.0)	703 (495 to 982)	1.7 (1.2 to 2.4)	190.3 (97.8 to 316.4)	11 300 (7 870 to 15 900)	25.7 (18.0 to 36.0)	149.0 (69.7 to 254.9)
Iran (Islamic Republic of)	483 (442 to 530)	1.1 (1.0 to 1.2)	188.9 (133.9 to 277.6)	912 (835 to 1 000)	2.0 (1.8 to 2.1)	221.4 (161.3 to 315.4)	12 200 (11 200 to 13 300)	24.7 (22.7 to 27.0)	152.8 (105.4 to 222.8)
Iraq	213 (162 to 271)	1.5 (1.1 to 1.8)	159.4 (84.7 to 268.7)	410 (305 to 532)	2.5 (2.0 to 3.2)	210.8 (120.1 to 343.0)	6 500 (4 810 to 8 550)	37.5 (28.1 to 48.3)	164.5 (85.8 to 283.3)
Jordan	62.3 (51.1 to 76.8)	1.7 (1.4 to 2.0)	260.6 (172.7 to 377.2)	125 (103 to 153)	2.9 (2.4 to 3.5)	329.5 (224.7 to 468.0)	1 800 (1 470 to 2 220)	37.5 (30.7 to 46.1)	239.1 (153.6 to 351.6)
Kuwait	17.3 (14.3 to 20.7)	1.1 (0.9 to 1.4)	148.2 (106.0 to 200.2)	37.9 (31.0 to 45.9)	2.1 (1.7 to 2.6)	164.7 (114.5 to 225.4)	479 (392 to 582)	23.9 (19.7 to 28.9)	115.1 (75.4 to 165.3)
Lebanon	67.4 (53.4 to 88.7)	2.1 (1.6 to 2.7)	104.6 (48.6 to 210.8)	141 (109 to 186)	4.3 (3.3 to 5.6)	151.8 (76.4 to 284.2)	1 700 (1 300 to 2 260)	51.7 (39.1 to 68.9)	79.9 (26.0 to 174.3)
Libya	56.3 (43.3 to 73.3)	1.7 (1.3 to 2.2)	178.1 (98.1 to 332.5)	107 (82.3 to 140)	3.0 (2.3 to 3.9)	215.3 (119.2 to 389.4)	1 670 (1 270 to 2 230)	43.5 (33.4 to 57.4)	182.7 (94.8 to 344.9)

Super-region, **region,** or country or territory	Deaths, LOC			Incidence, LOC			DALYs, LOC		
	**Counts, 2019 (95% UI)**	**Age-standardized rate, 2019 (95% UI)**	**% change of age-standardized rates, 1990–2019 (95% UI)**	**Counts, 2019 (95% UI)**	**Age-standardized rate, 2019 (95% UI)**	**% change of age-standardized rates, 1990–2019 (95% UI)**	**Counts, 2019 (95% UI)**	**Age-standardized rate, 2019 (95% UI)**	**% change of age-standardized rates, 1990–2019 (95% UI)**
Morocco	377 (287 to 483)	1.9 (1.5 to 2.4)	140.2 (69.1 to 231.1)	662 (501 to 847)	3.2 (2.4 to 4.0)	173.1 (94.8 to 279.9)	10 900 (8 200 to 14 300)	49.9 (38.0 to 64.4)	130.2 (62.3 to 223.7)
Oman	20.9 (17.1 to 26.7)	2.1 (1.8 to 2.4)	119.5 (53.2 to 228.3)	47.5 (38.9 to 61.9)	3.8 (3.2 to 4.6)	181.0 (96.4 to 321.3)	667 (539 to 894)	46.1 (38.2 to 58.0)	119.6 (50.8 to 232.4)
Palestine	16.6 (13.8 to 19.4)	1.2 (1.0 to 1.4)	132.9 (62.7 to 251.8)	31.2 (26.1 to 37.0)	2.0 (1.7 to 2.4)	169.9 (86.3 to 310.3)	470 (389 to 556)	27.6 (22.9 to 32.3)	142.8 (65.4 to 269.3)
Qatar	8.63 (6.26 to 11.5)	2.0 (1.6 to 2.6)	498.9 (293.2 to 829.3)	21.5 (15.6 to 29.1)	3.6 (2.8 to 4.6)	712.1 (440.5 to 1 160.6)	294 (209 to 399)	38.4 (29.5 to 49.8)	515.9 (301.2 to 850.8)
Saudi Arabia	204 (153 to 267)	1.7 (1.3 to 2.1)	221.2 (110.6 to 402.5)	479 (357 to 641)	3.3 (2.6 to 4.2)	418.1 (245.0 to 718.7)	7 090 (5 210 to 9 600)	42.5 (32.5 to 54.8)	269.3 (136.9 to 489.9)
Sudan	146 (96.8 to 203)	1.3 (0.8 to 1.7)	86.4 (27.6 to 178.2)	242 (156 to 339)	1.9 (1.2 to 2.6)	115.6 (38.4 to 226.9)	4 230 (2 760 to 6 050)	30.8 (20.3 to 43.4)	85.6 (23.6 to 188.5)
Syrian Arab Republic	61.3 (46.6 to 82.0)	0.9 (0.7 to 1.2)	103.0 (41.7 to 203.1)	113 (84.9 to 154)	1.5 (1.1 to 2.0)	139.1 (66.4 to 255.0)	1 620 (1 210 to 2 220)	20.0 (15.2 to 27.0)	94.1 (34.7 to 189.8)
Tunisia	161 (116 to 218)	2.1 (1.5 to 2.8)	155.3 (70.9 to 279.9)	315 (225 to 429)	3.9 (2.8 to 5.3)	193.6 (96.8 to 340.3)	4 250 (2 990 to 5 850)	51.3 (36.3 to 70.0)	137.5 (58.3 to 261.6)
Turkey	617 (489 to 761)	1.1 (0.9 to 1.4)	76.7 (28.4 to 143.9)	1 150 (920 to 1 430)	2.1 (1.7 to 2.6)	114.3 (52.5 to 199.8)	15 000 (11 800 to 18 800)	26.2 (20.6 to 32.8)	50.3 (7.5 to 111.9)
United Arab Emirates	75.1 (42.9 to 132)	2.4 (1.5 to 3.8)	663.3 (350.7 to 1 304.9)	157 (89.8 to 279)	3.9 (2.4 to 6.5)	757.7 (401.6 to 1 474.2)	2 970 (1 660 to 5 290)	59.0 (35.8 to 99.3)	681.6 (349.6 to 1 372.3)
Yemen	110 (77.6 to 154)	1.4 (1.0 to 1.9)	157.4 (80.7 to 282.2)	175 (123 to 245)	2.0 (1.4 to 2.7)	180.8 (92.4 to 322.9)	3 240 (2 260 to 4 630)	33.1 (23.3 to 46.6)	151.8 (69.2 to 291.9)
South Asia									
**South Asia**									
Bangladesh	5 150 (3 550 to 7 180)	6.4 (4.4 to 8.8)	72.4 (19.6 to 159.1)	8 100 (5 520 to 11 400)	9.6 (6.6 to 13.3)	95.9 (35.8 to 199.4)	142 000 (96 300 to 201 000)	162.2 (110.9 to 228.6)	57.4 (7.4 to 147.0)
Bhutan	26.4 (18.8 to 35.2)	7.6 (5.6 to 9.8)	93.0 (30.6 to 183.9)	42.6 (29.7 to 58.0)	11.6 (8.2 to 15.3)	119.5 (47.2 to 232.6)	728 (498 to 1 030)	188.5 (131.0 to 260.8)	65.7 (7.4 to 161.9)
India	65 300 (54 200 to 78 200)	9.2 (7.6 to 11.0)	147.7 (90.3 to 215.4)	104 000 (85 600 to 124 000)	13.9 (11.4 to 16.4)	170.7 (106.0 to 240.2)	1 900 000 (1 570 000 to 2 300 000)	243.1 (200.8 to 292.3)	129.5 (76.3 to 191.9)
Nepal	983 (760 to 1 210)	7.3 (5.6 to 8.9)	111.1 (49.0 to 193.2)	1 500 (1 150 to 1 880)	10.4 (8.1 to 12.9)	132.6 (62.7 to 226.8)	27 300 (20 700 to 34 500)	181.0 (138.5 to 226.4)	86.7 (29.6 to 166.4)
Pakistan	17 400 (13 900 to 21 900)	23.2 (18.7 to 28.9)	145.3 (85.7 to 232.2)	28 000 (22 400 to 35 100)	34.4 (28.0 to 43.3)	178.0 (108.2 to 275.4)	578 000 (462 000 to 738 000)	659.6 (527.7 to 838.1)	166.3 (101.6 to 260.0)
Southeast Asia, East Asia, and Oceania									

Super-region, **region,** or country or territory	**Deaths, LOC**			**Incidence, LOC**			**DALYs, LOC**		
	**Counts, 2019 (95% UI)**	**Age-standardized rate, 2019 (95% UI)**	**% change of age-standardized rates, 1990–2019 (95% UI)**	**Counts, 2019 (95% UI)**	**Age-standardized rate, 2019 (95% UI)**	**% change of age-standardized rates, 1990–2019 (95% UI)**	**Counts, 2019 (95% UI)**	**Age-standardized rate, 2019 (95% UI)**	**% change of age-standardized rates, 1990–2019 (95% UI)**
**East Asia**									
China	22 600 (18 900 to 27 000)	1.8 (1.5 to 2.2)	208.9 (141.4 to 290.9)	45 000 (37 500 to 54 000)	3.5 (3.0 to 4.2)	272.2 (197.6 to 374.7)	573 000 (477 000 to 688 000)	44.3 (36.9 to 52.9)	165.0 (105.0 to 235.8)
Democratic People's Republic of Korea	388 (305 to 505)	1.9 (1.5 to 2.5)	96.5 (45.0 to 166.8)	747 (572 to 989)	3.6 (2.8 to 4.8)	79.0 (29.8 to 147.7)	10 900 (8 120 to 14 600)	51.9 (39.3 to 70.1)	72.8 (22.7 to 146.7)
Taiwan (Province of China)	2 240 (1 730 to 2 990)	9.3 (7.1 to 12.4)	432.2 (311.4 to 603.4)	6 070 (4 610 to 8 000)	25.8 (19.6 to 34.0)	486.0 (342.0 to 678.4)	69 500 (52 900 to 93 500)	298.4 (225.8 to 401.1)	391.2 (275.7 to 557.8)
**Oceania**									
American Samoa	0.393 (0.327 to 0.462)	1.4 (1.2 to 1.7)	106.6 (63.9 to 158.3)	0.740 (0.611 to 0.890)	2.5 (2.1 to 3.0)	98.1 (53.5 to 153.1)	10.0 (8.16 to 12.0)	32.4 (26.8 to 38.7)	82.7 (40.2 to 133.1)
Cook Islands	0.343 (0.282 to 0.411)	2.3 (1.9 to 2.7)	77.4 (36.9 to 130.0)	0.688 (0.556 to 0.843)	4.6 (3.7 to 5.7)	81.3 (37.0 to 140.2)	8.47 (6.75 to 10.4)	57.0 (44.5 to 70.5)	58.0 (17.1 to 113.9)
Fiji	15.1 (11.8 to 18.9)	3.5 (2.8 to 4.4)	86.0 (33.1 to 163.2)	27.8 (21.2 to 35.2)	5.8 (4.6 to 7.3)	80.5 (27.2 to 160.6)	436 (334 to 551)	85.7 (66.3 to 107.5)	69.0 (18.5 to 141.1)
Guam	3.19 (2.60 to 3.86)	2.7 (2.2 to 3.2)	208.1 (139.3 to 288.1)	6.22 (5.05 to 7.54)	5.2 (4.3 to 6.3)	185.9 (121.5 to 263.5)	88.3 (70.5 to 107)	73.7 (59.6 to 89.4)	189.9 (122.9 to 272.0)
Kiribati	8.17 (6.19 to 10.6)	19.4 (15.2 to 24.4)	95.3 (41.9 to 168.3)	13.8 (10.4 to 17.9)	28.9 (22.3 to 36.7)	105.5 (48.4 to 184.2)	272 (203 to 355)	520.6 (398.1 to 669.5)	94.1 (37.6 to 172.0)
Marshall Islands	0.773 (0.565 to 1.05)	3.7 (2.7 to 4.9)	117.0 (56.7 to 209.3)	1.38 (1.01 to 1.87)	5.7 (4.3 to 7.6)	130.2 (63.8 to 232.3)	25.4 (18.1 to 34.9)	96.0 (70.3 to 129.7)	124.6 (58.9 to 225.7)
Micronesia (Federated States of)	1.60 (1.08 to 2.25)	3.8 (2.7 to 5.1)	58.7 (8.3 to 123.7)	2.93 (1.95 to 4.16)	6.2 (4.3 to 8.5)	73.7 (14.9 to 149.8)	50.5 (32.9 to 73.5)	98.7 (66.9 to 139.9)	56.8 (1.9 to 129.5)
Nauru	0.114 (0.0761 to 0.156)	4.3 (3.1 to 5.6)	7.8 (-19.7 to 45.6)	0.254 (0.170 to 0.351)	7.6 (5.4 to 10.1)	17.9 (-11.8 to 57.1)	4.25 (2.74 to 5.91)	112.5 (77.4 to 150.8)	11.6 (-18.3 to 51.7)
Niue	0.0436 (0.0342 to 0.0538)	3.2 (2.5 to 4.0)	-1.8 (-24.7 to 27.4)	0.0849 (0.0655 to 0.107)	6.4 (4.9 to 8.1)	9.4 (-17.6 to 45.1)	1.07 (0.803 to 1.36)	80.3 (60.5 to 102.8)	-2.2 (-28.0 to 31.8)
Northern Mariana Islands	3.35 (2.81 to 3.97)	10.9 (9.3 to 12.6)	434.5 (309.6 to 584.9)	7.17 (5.88 to 8.68)	21.3 (17.7 to 25.1)	360.6 (240.9 to 514.8)	97.4 (79.3 to 119)	269.9 (223.5 to 322.9)	328.7 (215.2 to 486.1)
Palau	2.81 (2.21 to 3.58)	22.6 (18.0 to 28.2)	91.7 (38.1 to 163.8)	6.37 (4.91 to 8.25)	46.6 (36.3 to 59.2)	101.4 (44.4 to 179.4)	84.3 (65.0 to 110)	581.3 (453.6 to 749.9)	92.2 (35.0 to 166.5)
Papua New Guinea	82.1 (56.8 to 125)	2.8 (1.9 to 4.1)	198.3 (119.4 to 313.8)	138 (94.9 to 208)	4.1 (2.9 to 6.1)	206.9 (120.9 to 325.1)	2 660 (1 790 to 4 160)	71.4 (49.5 to 108.5)	199.9 (116.0 to 323.1)
Samoa	2.00 (1.56 to 2.60)	2.1 (1.7 to 2.7)	44.7 (4.6 to 100.6)	3.66 (2.79 to 4.75)	3.7 (2.9 to 4.8)	53.3 (7.6 to 115.5)	58.8 (43.9 to 77.9)	57.8 (43.8 to 75.9)	43.9 (-2.1 to 106.2)
Solomon Islands	8.03 (4.47 to 12.0)	3.9 (2.4 to 5.6)	166.5 (89.4 to 272.4)	15.2 (8.46 to 21.9)	6.4 (3.8 to 9.1)	189.5 (104.4 to 310.6)	283 (151 to 426)	109.5 (61.7 to 162.9)	169.9 (89.4 to 282.6)

Super-region, **region,** or country or territory	**Deaths, LOC**			**Incidence, LOC**			**DALYs, LOC**		
	**Counts, 2019 (95% UI)**	**Age-standardized rate, 2019 (95% UI)**	**% change of age-standardized rates, 1990–2019 (95% UI)**	**Counts, 2019 (95% UI)**	**Age-standardized rate, 2019 (95% UI)**	**% change of age-standardized rates, 1990–2019 (95% UI)**	**Counts, 2019 (95% UI)**	**Age-standardized rate, 2019 (95% UI)**	**% change of age-standardized rates, 1990–2019 (95% UI)**
Tokelau	0.0215 (0.0166 to 0.0275)	2.7 (2.1 to 3.4)	-0.5 (-26.8 to 34.5)	0.0403 (0.0302 to 0.0531)	4.8 (3.6 to 6.3)	13.4 (-17.5 to 55.7)	0.553 (0.410 to 0.736)	65.2 (48.5 to 86.8)	2.0 (-27.3 to 41.4)
Tonga	1.26 (0.993 to 1.60)	2.5 (2.0 to 3.2)	77.0 (32.5 to 133.6)	2.16 (1.67 to 2.79)	4.3 (3.3 to 5.5)	74.9 (31.2 to 134.7)	31.5 (24.1 to 41.0)	61.3 (47.0 to 79.9)	62.7 (19.7 to 122.7)
Tuvalu	0.195 (0.148 to 0.257)	3.2 (2.4 to 4.1)	50.5 (8.4 to 112.0)	0.349 (0.261 to 0.463)	5.3 (4.0 to 7.1)	58.2 (12.8 to 126.9)	5.50 (4.03 to 7.39)	81.9 (60.6 to 109.3)	42.1 (0.3 to 103.7)
Vanuatu	3.41 (2.32 to 5.04)	3.2 (2.2 to 4.7)	205.9 (125.5 to 337.0)	5.57 (3.78 to 8.19)	4.8 (3.3 to 7.0)	208.3 (123.9 to 350.4)	102 (67.7 to 153)	83.3 (55.7 to 123.7)	194.6 (107.8 to 337.9)
**Southeast Asia**									
Cambodia	318 (238 to 410)	4.5 (3.4 to 5.7)	192.1 (106.6 to 303.5)	545 (399 to 712)	7.1 (5.2 to 9.1)	227.2 (134.5 to 349.1)	8 650 (6 390 to 11 300)	106.8 (79.3 to 139.1)	167.3 (85.1 to 272.6)
Indonesia	4 190 (3 100 to 5 670)	3.4 (2.5 to 4.6)	146.7 (84.2 to 212.9)	7 230 (5 260 to 9 790)	5.3 (3.9 to 7.2)	158.4 (93.3 to 228.9)	112 000 (82 300 to 152 000)	76.7 (56.7 to 103.5)	122.6 (65.3 to 182.8)
Lao People's Democratic Republic	94.0 (70.5 to 121)	3.6 (2.8 to 4.5)	67.8 (13.2 to 146.8)	154 (115 to 199)	5.4 (4.0 to 6.9)	87.9 (30.0 to 176.8)	2 660 (1 980 to 3 480)	86.4 (64.4 to 112.6)	58.3 (5.1 to 139.7)
Malaysia	668 (522 to 850)	4.2 (3.3 to 5.3)	151.5 (83.0 to 244.0)	1 430 (1 110 to 1 830)	8.3 (6.5 to 10.5)	192.7 (116.1 to 291.8)	17 700 (13 500 to 22 800)	99.0 (76.3 to 127.2)	135.8 (69.7 to 219.4)
Maldives	7.57 (6.19 to 9.13)	4.6 (3.7 to 5.5)	159.0 (98.4 to 267.3)	15.8 (12.9 to 19.1)	8.5 (6.9 to 10.3)	212.6 (129.8 to 348.1)	172 (140 to 208)	86.1 (70.0 to 103.9)	110.1 (56.5 to 208.9)
Mauritius	42.9 (34.8 to 52.4)	3.9 (3.2 to 4.8)	166.8 (112.5 to 227.9)	88.1 (70.2 to 108)	8.0 (6.4 to 9.8)	179.2 (122.1 to 245.6)	1 140 (910 to 1 410)	102.2 (81.7 to 125.5)	145.6 (95.6 to 207.2)
Myanmar	956 (733 to 1 270)	3.4 (2.7 to 4.5)	74.7 (21.4 to 152.9)	1 610 (1 220 to 2 150)	5.4 (4.1 to 7.2)	91.0 (33.2 to 171.3)	25 600 (19 200 to 34 400)	82.3 (62.3 to 109.2)	57.0 (7.2 to 130.7)
Philippines	1 540 (1 260 to 1 860)	3.3 (2.8 to 3.9)	93.6 (54.0 to 151.9)	2 740 (2 200 to 3 410)	5.4 (4.4 to 6.6)	92.2 (50.6 to 153.9)	42 700 (34 800 to 51 900)	79.6 (65.1 to 96.1)	82.0 (44.6 to 137.4)
Seychelles	7.84 (6.63 to 9.35)	11.5 (9.8 to 13.5)	151.1 (100.6 to 214.8)	14.6 (12.2 to 17.5)	20.3 (17.1 to 24.1)	191.6 (130.6 to 270.2)	212 (175 to 255)	283.6 (237.2 to 339.0)	163.6 (105.6 to 236.2)
Sri Lanka	1 020 (739 to 1 360)	6.7 (4.9 to 8.7)	148.2 (76.8 to 236.2)	2 060 (1 500 to 2 720)	12.8 (9.3 to 16.8)	186.3 (103.3 to 293.5)	24 400 (17 500 to 32 700)	149.1 (107.4 to 198.2)	129.6 (59.2 to 217.4)
Thailand	2 950 (2 210 to 3 820)	4.7 (3.5 to 6.0)	131.7 (69.6 to 215.2)	5 710 (4 190 to 7 540)	9.0 (6.6 to 11.9)	149.8 (77.8 to 244.5)	67 600 (48 900 to 89 100)	105.4 (76.6 to 138.1)	90.9 (33.1 to 163.7)
Timor-Leste	15.3 (11.2 to 19.6)	3.2 (2.4 to 4.0)	223.3 (129.8 to 358.5)	24.8 (17.9 to 32.1)	4.8 (3.5 to 6.1)	233.8 (133.7 to 367.2)	396 (268 to 524)	73.8 (52.2 to 96.6)	165.0 (78.2 to 277.6)
Viet Nam	3 440 (2 650 to 4 330)	5.8 (4.5 to 7.3)	183.3 (103.7 to 282.8)	7 080 (5 360 to 9 040)	11.3 (8.7 to 14.3)	244.8 (143.7 to 370.0)	99 300 (74 200 to 128 000)	151.8 (115.4 to 193.5)	188.8 (100.2 to 304.4)
Sub-Saharan Africa									
**Central sub-Saharan Africa**									

Super-region, **region,** or country or territory	Deaths, LOC			Incidence, LOC			DALYs, LOC		
	**Counts, 2019 (95% UI)**	**Age-standardized rate, 2019 (95% UI)**	**% change of age-standardized rates, 1990–2019 (95% UI)**	**Counts, 2019 (95% UI)**	**Age-standardized rate, 2019 (95% UI)**	**% change of age-standardized rates, 1990–2019 (95% UI)**	**Counts, 2019 (95% UI)**	**Age-standardized rate, 2019 (95% UI)**	**% change of age-standardized rates, 1990–2019 (95% UI)**
Angola	254 (190 to 328)	3.6 (2.8 to 4.6)	190.7 (99.3 to 332.2)	371 (277 to 485)	4.8 (3.6 to 6.2)	218.9 (120.8 to 369.6)	7 910 (5 840 to 10 400)	92.0 (68.9 to 119.0)	183.7 (89.7 to 322.3)
Central African Republic	51.8 (32.7 to 77.5)	3.6 (2.4 to 5.2)	64.3 (19.9 to 120.1)	68.5 (43.4 to 102)	4.4 (2.9 to 6.4)	64.5 (19.1 to 120.8)	1 710 (1 050 to 2 600)	98.1 (62.9 to 146.1)	68.9 (20.5 to 131.2)
Congo	61.8 (45.4 to 82.2)	3.8 (2.9 to 4.9)	105.6 (39.6 to 215.0)	91.0 (65.4 to 122)	5.1 (3.8 to 6.6)	119.5 (46.3 to 239.4)	1 900 (1 330 to 2 590)	95.4 (70.1 to 126.7)	103.0 (32.8 to 223.3)
Democratic Republic of the Congo	644 (445 to 866)	2.9 (2.0 to 3.9)	118.3 (53.0 to 194.3)	908 (632 to 1 220)	3.8 (2.6 to 5.1)	125.3 (58.1 to 206.5)	19 500 (13 500 to 26 400)	73.3 (50.6 to 98.5)	117.3 (51.1 to 198.6)
Equatorial Guinea	10.8 (6.86 to 16.5)	3.7 (2.5 to 5.3)	154.6 (27.0 to 362.3)	17.0 (10.5 to 26.3)	5.2 (3.4 to 7.7)	208.7 (52.8 to 463.9)	323 (192 to 512)	89.4 (56.1 to 136.4)	146.0 (18.4 to 362.0)
Gabon	29.4 (20.5 to 40.1)	4.5 (3.2 to 5.9)	71.9 (20.5 to 141.1)	44.5 (30.6 to 61.2)	6.3 (4.4 to 8.5)	85.1 (27.5 to 164.2)	856 (575 to 1 200)	113.2 (77.8 to 156.2)	72.5 (16.9 to 151.8)
**Eastern sub-Saharan Africa**									
Burundi	124 (84.4 to 176)	4.0 (2.8 to 5.6)	70.6 (15.0 to 153.6)	179 (122 to 254)	5.3 (3.7 to 7.5)	81.2 (21.7 to 170.3)	4 060 (2 730 to 5 880)	111.1 (75.7 to 157.8)	75.1 (16.5 to 166.9)
Comoros	10.3 (7.57 to 13.8)	3.3 (2.5 to 4.3)	132.2 (61.6 to 353.9)	15.0 (10.7 to 20.5)	4.6 (3.4 to 6.2)	150.7 (70.1 to 423.7)	302 (208 to 421)	88.6 (62.1 to 121.8)	128.7 (51.4 to 425.6)
Djibouti	15.9 (10.7 to 24.0)	3.9 (2.9 to 5.6)	373.2 (221.3 to 605.8)	24.7 (16.1 to 37.7)	5.5 (3.9 to 8.1)	398.5 (236.7 to 645.3)	523 (330 to 812)	105.7 (71.5 to 158.9)	352.9 (193.1 to 594.6)
Eritrea	80.1 (60.5 to 106)	4.3 (3.3 to 5.5)	220.5 (126.3 to 364.7)	119 (87.9 to 160)	5.8 (4.5 to 7.6)	247.6 (144.6 to 403.2)	2 740 (2 010 to 3 690)	120.9 (91.3 to 160.3)	211.2 (115.8 to 353.0)
Ethiopia	771 (525 to 1 000)	2.9 (2.0 to 3.7)	60.7 (16.1 to 158.1)	1 150 (805 to 1 500)	4.0 (2.8 to 5.2)	76.2 (27.8 to 189.2)	23 500 (15 900 to 30 500)	75.5 (51.0 to 98.2)	48.4 (6.0 to 147.4)
Kenya	883 (679 to 1 100)	6.1 (4.8 to 7.5)	295.6 (227.0 to 389.4)	1 300 (982 to 1 620)	8.1 (6.2 to 10.0)	292.3 (218.8 to 390.2)	28 100 (21 400 to 35 400)	162.4 (124.9 to 202.8)	304.9 (228.5 to 406.6)
Madagascar	227 (167 to 300)	3.0 (2.3 to 3.9)	113.8 (54.4 to 192.0)	337 (245 to 443)	4.1 (3.0 to 5.4)	125.2 (60.6 to 213.4)	7 620 (5 540 to 10 100)	83.8 (61.6 to 110.1)	120.7 (56.6 to 209.0)
Malawi	128 (99.2 to 160)	2.7 (2.1 to 3.3)	94.3 (48.2 to 152.8)	193 (147 to 242)	3.7 (2.9 to 4.6)	102.1 (51.0 to 169.5)	4 090 (3 060 to 5 220)	73.0 (56.0 to 92.3)	91.0 (38.7 to 156.9)
Mozambique	190 (134 to 259)	2.7 (2.0 to 3.7)	173.2 (87.9 to 299.9)	263 (185 to 363)	3.6 (2.6 to 4.8)	196.7 (100.9 to 336.5)	5 670 (3 960 to 7 910)	71.4 (50.3 to 98.4)	184.9 (90.3 to 324.6)
Rwanda	149 (115 to 190)	3.8 (3.0 to 4.7)	69.3 (17.1 to 161.1)	229 (174 to 297)	5.4 (4.2 to 6.7)	88.9 (27.8 to 195.8)	4 720 (3 550 to 6 230)	102.2 (78.3 to 131.5)	64.9 (9.4 to 166.9)
Somalia	144 (83.6 to 225)	3.2 (1.9 to 4.9)	149.3 (65.5 to 251.4)	197 (113 to 309)	4.0 (2.3 to 6.2)	148.1 (59.8 to 252.4)	4 910 (2 780 to 7 790)	89.3 (51.5 to 139.4)	147.2 (60.2 to 256.9)
South Sudan	79.9 (51.6 to 125)	3.2 (2.1 to 4.8)	51.0 (4.7 to 113.2)	110 (72.1 to 172)	4.1 (2.7 to 6.1)	54.3 (6.0 to 120.5)	2 510 (1 550 to 4 070)	83.6 (53.8 to 131.8)	54.1 (2.9 to 127.9)
Uganda	407 (307 to 512)	4.3 (3.3 to 5.3)	192.1 (114.6 to 289.7)	624 (465 to 788)	6.0 (4.6 to 7.5)	221.0 (132.9 to 336.4)	13 200 (9 780 to 17 200)	117.9 (88.3 to 149.4)	204.1 (118.7 to 320.5)
United Republic of Tanzania	617 (476 to 814)	3.8 (3.1 to 4.9)	132.4 (72.8 to 205.5)	932 (708 to 1 240)	5.4 (4.2 to 7.1)	145.7 (79.9 to 229.5)	19 300 (14 400 to 26 400)	103.4 (78.9 to 139.0)	132.6 (66.8 to 217.8)

Super-region, **region,** or country or territory	**Deaths, LOC**			**Incidence, LOC**			**DALYs, LOC**		
	**Counts, 2019 (95% UI)**	**Age-standardized rate, 2019 (95% UI)**	**% change of age-standardized rates, 1990–2019 (95% UI)**	**Counts, 2019 (95% UI)**	**Age-standardized rate, 2019 (95% UI)**	**% change of age-standardized rates, 1990–2019 (95% UI)**	**Counts, 2019 (95% UI)**	**Age-standardized rate, 2019 (95% UI)**	**% change of age-standardized rates, 1990–2019 (95% UI)**
Zambia	210 (155 to 282)	4.6 (3.5 to 6.0)	162.1 (92.1 to 257.8)	330 (242 to 444)	6.6 (4.9 to 8.7)	193.0 (112.4 to 296.0)	7 060 (5 070 to 9 590)	128.1 (93.6 to 172.4)	171.4 (93.2 to 277.4)
**Southern sub-Saharan Africa**									
Botswana	51.9 (35.7 to 71.8)	5.8 (4.2 to 7.8)	184.8 (91.2 to 327.8)	88.2 (59.2 to 124)	9.1 (6.4 to 12.4)	221.6 (110.7 to 395.8)	1 630 (1 070 to 2 310)	155.9 (105.7 to 216.8)	192.6 (90.6 to 358.4)
Eswatini	19.7 (13.7 to 26.9)	5.3 (3.8 to 7.1)	116.3 (48.3 to 208.9)	29.9 (20.5 to 41.1)	7.5 (5.3 to 10.1)	125.6 (51.8 to 226.5)	623 (419 to 873)	146.2 (100.6 to 201.3)	120.5 (44.0 to 222.2)
Lesotho	48.8 (32.8 to 68.8)	6.0 (4.1 to 8.3)	94.5 (28.0 to 194.9)	69.4 (46.5 to 98.6)	8.0 (5.5 to 11.3)	99.6 (30.3 to 207.4)	1 520 (997 to 2 180)	166.5 (110.3 to 237.1)	108.6 (32.5 to 220.2)
Namibia	74.2 (56.1 to 97.7)	8.1 (6.3 to 10.4)	159.4 (84.0 to 291.7)	119 (87.7 to 159)	12.3 (9.3 to 16.2)	193.7 (102.6 to 357.7)	2 270 (1 630 to 3 080)	223.4 (164.8 to 298.4)	167.4 (81.3 to 327.1)
South Africa	1 040 (939 to 1 180)	3.7 (3.4 to 4.2)	68.9 (47.9 to 92.1)	1 630 (1 470 to 1 860)	5.6 (5.0 to 6.3)	72.2 (52.3 to 92.5)	29 000 (26 100 to 33 400)	94.7 (85.4 to 108.5)	56.7 (39.2 to 76.3)
Zimbabwe	163 (124 to 207)	3.6 (2.8 to 4.5)	143.6 (84.8 to 219.9)	253 (194 to 322)	5.1 (4.0 to 6.4)	143.6 (82.6 to 222.5)	5 250 (3 920 to 6 810)	98.0 (74.2 to 124.7)	161.4 (93.8 to 250.0)
**Western sub-Saharan Africa**									
Benin	69.4 (53.3 to 90.4)	2.3 (1.9 to 3.0)	168.9 (106.9 to 256.7)	99.7 (75.2 to 132)	3.1 (2.4 to 4.0)	184.7 (115.4 to 282.1)	2 000 (1 470 to 2 690)	57.0 (43.2 to 74.9)	174.6 (101.2 to 275.6)
Burkina Faso	143 (109 to 186)	2.6 (2.0 to 3.3)	146.0 (88.9 to 220.2)	207 (158 to 269)	3.4 (2.6 to 4.4)	159.1 (96.1 to 242.3)	4 220 (3 160 to 5 560)	63.6 (48.1 to 82.8)	149.5 (84.9 to 233.7)
Cabo Verde	15.3 (12.5 to 18.6)	5.5 (4.5 to 6.7)	870.0 (677.8 to 1 099.3)	24.0 (19.5 to 29.4)	8.5 (6.9 to 10.3)	1 008.5 (779.6 to 1 276.5)	392 (316 to 486)	134.1 (108.7 to 165.4)	971.9 (746.7 to 1 254.3)
Cameroon	220 (163 to 301)	3.0 (2.3 to 3.9)	211.7 (124.5 to 328.4)	331 (239 to 457)	4.1 (3.1 to 5.6)	233.9 (142.0 to 359.3)	6 620 (4 680 to 9 200)	74.9 (54.7 to 103.2)	211.5 (118.4 to 341.9)
Chad	81.6 (61.9 to 104)	2.4 (1.8 to 3.0)	163.0 (100.2 to 252.1)	112 (83.9 to 144)	3.0 (2.3 to 3.8)	171.3 (104.7 to 262.9)	2 380 (1 770 to 3 090)	58.9 (44.3 to 75.8)	173.2 (101.0 to 272.5)
Côte d'Ivoire	175 (130 to 223)	2.7 (2.1 to 3.3)	170.6 (97.1 to 267.3)	256 (187 to 331)	3.5 (2.7 to 4.4)	172.8 (93.1 to 272.8)	5 370 (3 830 to 7 050)	65.8 (48.9 to 84.3)	156.4 (78.9 to 264.6)
Gambia	14.1 (10.3 to 18.6)	2.3 (1.7 to 3.0)	258.6 (143.4 to 422.0)	21.2 (15.3 to 28.2)	3.2 (2.4 to 4.2)	271.1 (150.3 to 447.9)	421 (299 to 567)	60.2 (42.9 to 80.0)	246.8 (130.9 to 422.7)
Ghana	123 (97.6 to 155)	1.3 (1.0 to 1.6)	150.6 (85.1 to 237.8)	187 (146 to 235)	1.8 (1.4 to 2.2)	164.7 (94.0 to 257.5)	3 460 (2 670 to 4 400)	30.5 (23.9 to 38.3)	140.1 (72.5 to 228.7)
Guinea	222 (170 to 285)	6.3 (4.9 to 8.0)	102.3 (48.9 to 163.2)	314 (238 to 407)	8.4 (6.4 to 10.8)	113.3 (55.9 to 182.2)	6 640 (4 990 to 8 730)	166.5 (125.6 to 217.4)	109.1 (49.6 to 175.3)
Guinea-Bissau	13.8 (10.2 to 18.1)	3.0 (2.3 to 3.9)	77.4 (25.4 to 152.7)	20.1 (14.7 to 26.2)	4.0 (3.0 to 5.2)	87.0 (32.8 to 168.8)	436 (316 to 576)	79.1 (58.2 to 103.1)	79.7 (26.3 to 163.4)
Liberia	27.5 (20.3 to 36.7)	2.2 (1.7 to 2.8)	85.0 (32.5 to 154.9)	40.2 (29.3 to 53.9)	2.9 (2.2 to 3.8)	102.2 (43.6 to 179.7)	817 (577 to 1 110)	52.8 (38.5 to 70.9)	97.3 (34.9 to 179.6)
Mali	96.8 (74.8 to 127)	1.7 (1.4 to 2.2)	122.0 (63.8 to 193.4)	143 (110 to 189)	2.4 (1.9 to 3.1)	137.6 (74.5 to 219.7)	2 990 (2 250 to 3 970)	46.6 (35.6 to 61.5)	122.0 (59.9 to 203.1)

Super-region, **region,** or country or territory	**Deaths, LOC**			**Incidence, LOC**			**DALYs, LOC**		
	**Counts, 2019 (95% UI)**	**Age-standardized rate, 2019 (95% UI)**	**% change of age-standardized rates, 1990–2019 (95% UI)**	**Counts, 2019 (95% UI)**	**Age-standardized rate, 2019 (95% UI)**	**% change of age-standardized rates, 1990–2019 (95% UI)**	**Counts, 2019 (95% UI)**	**Age-standardized rate, 2019 (95% UI)**	**% change of age-standardized rates, 1990–2019 (95% UI)**
Mauritania	26.2 (18.7 to 35.6)	2.1 (1.5 to 2.8)	85.9 (35.3 to 167.7)	38.7 (27.0 to 53.4)	2.9 (2.1 to 4.0)	99.9 (41.6 to 188.4)	686 (464 to 980)	48.8 (33.6 to 68.2)	71.0 (17.6 to 154.8)
Niger	91.8 (60.5 to 126)	2.0 (1.3 to 2.7)	192.7 (120.8 to 289.5)	130 (85.5 to 178)	2.6 (1.7 to 3.5)	203.7 (126.9 to 307.1)	2 680 (1 730 to 3 720)	48.1 (31.6 to 65.9)	174.8 (101.7 to 272.3)
Nigeria	602 (469 to 750)	1.2 (0.9 to 1.5)	124.8 (58.1 to 220.3)	890 (674 to 1 140)	1.6 (1.2 to 2.0)	142.6 (72.1 to 241.4)	16 700 (12 500 to 21 500)	26.9 (20.9 to 33.7)	127.0 (55.9 to 229.7)
São Tomé and Príncipe	0.902 (0.673 to 1.22)	1.4 (1.1 to 1.9)	119.8 (64.2 to 202.7)	1.36 (1.01 to 1.86)	2.0 (1.5 to 2.7)	145.8 (82.1 to 239.8)	24.5 (17.9 to 33.5)	33.5 (24.8 to 45.6)	134.7 (70.3 to 228.5)
Senegal	110 (81.9 to 142)	2.4 (1.8 to 3.1)	163.3 (95.9 to 251.6)	156 (115 to 206)	3.2 (2.4 to 4.2)	172.5 (97.6 to 265.5)	3 030 (2 150 to 4 060)	58.3 (42.5 to 76.7)	151.3 (79.8 to 242.4)
Sierra Leone	51.0 (38.6 to 68.2)	2.3 (1.8 to 3.0)	107.8 (50.6 to 184.8)	72.9 (54.6 to 97.5)	3.1 (2.4 to 4.1)	122.9 (59.8 to 211.4)	1 470 (1 080 to 2 010)	57.2 (42.8 to 77.5)	116.4 (52.4 to 208.6)
Togo	57.7 (43.8 to 75.0)	2.6 (2.0 to 3.2)	229.2 (142.8 to 348.4)	86.4 (65.0 to 114)	3.5 (2.7 to 4.5)	238.6 (148.1 to 372.1)	1 750 (1 280 to 2 350)	63.9 (48.3 to 83.6)	224.1 (131.0 to 356.8)

SDI=Socio-demographic Index; DALYs=disability-adjusted life-years; UI=uncertainty interval. See eFigure 3 (p53) and eTable 7 (p54) for details and definitions of the SDI regions.

eTable 11. Deaths, incidence, and DALYs counts and age-standardized rates (per 100 000) for Other pharynx cancer (OPC), both sexes combined, by country or territory, in 2019, and change in age-standardized rates from 1990 to 2019

Super-region, **region,** or country or territory	Deaths, OPC			Incidence, OPC			DALYs, OPC		
	**Counts, 2019 (95% UI)**	**Age-standardized rate, 2019 (95% UI)**	**% change of age-standardized rates, 1990–2019 (95% UI)**	**Counts, 2019 (95% UI)**	**Age-standardized rate, 2019 (95% UI)**	**% change of age-standardized rates, 1990–2019 (95% UI)**	**Counts, 2019 (95% UI)**	**Age-standardized rate, 2019 (95% UI)**	**% change of age-standardized rates, 1990–2019 (95% UI)**
Central Europe, Eastern Europe, and Central Asia									
**Central Asia**									
Armenia	15.7 (12.7 to 19.1)	0.6 (0.5 to 0.7)	15.3 (-11.8 to 51.9)	19.5 (15.8 to 23.7)	0.7 (0.6 to 0.9)	28.0 (-1.8 to 67.8)	420 (338 to 514)	15.7 (12.7 to 19.2)	4.0 (-20.4 to 36.6)
Azerbaijan	38.5 (29.2 to 50.4)	0.6 (0.5 to 0.8)	152.1 (76.7 to 238.9)	46.9 (35.9 to 60.5)	0.7 (0.5 to 0.9)	177.0 (97.7 to 272.2)	1 190 (903 to 1 550)	16.8 (12.9 to 21.8)	144.7 (74.1 to 229.5)
Georgia	56.5 (46.3 to 67.7)	1.6 (1.3 to 1.9)	64.2 (14.4 to 117.0)	64.6 (53.1 to 78.1)	1.8 (1.5 to 2.2)	61.3 (13.0 to 113.7)	1 500 (1 230 to 1 830)	43.7 (35.9 to 52.8)	52.0 (4.1 to 102.7)
Kazakhstan	164 (137 to 200)	1.4 (1.2 to 1.7)	-10.8 (-26.5 to 9.5)	212 (177 to 257)	1.8 (1.5 to 2.2)	2.8 (-15.6 to 26.3)	4 770 (3 960 to 5 790)	39.4 (32.9 to 47.7)	-15.0 (-30.2 to 4.6)
Kyrgyzstan	31.3 (26.0 to 37.2)	1.0 (0.9 to 1.2)	60.8 (23.4 to 104.6)	37.9 (31.6 to 45.1)	1.2 (1.0 to 1.4)	79.5 (38.8 to 125.6)	926 (771 to 1 110)	28.1 (23.5 to 33.4)	58.3 (21.7 to 100.3)
Mongolia	12.5 (9.48 to 16.1)	1.0 (0.8 to 1.3)	64.9 (20.4 to 127.0)	13.5 (10.3 to 17.5)	1.0 (0.8 to 1.3)	85.5 (35.6 to 157.5)	335 (251 to 443)	22.0 (16.8 to 28.7)	97.5 (42.7 to 179.5)
Tajikistan	31.3 (24.7 to 39.5)	1.0 (0.8 to 1.3)	80.7 (31.2 to 147.8)	36.1 (28.4 to 45.8)	1.1 (0.9 to 1.4)	93.2 (42.6 to 165.2)	956 (743 to 1 220)	25.3 (19.8 to 31.8)	90.3 (38.6 to 160.6)
Turkmenistan	43.9 (33.6 to 56.6)	1.6 (1.3 to 2.1)	98.7 (48.6 to 162.2)	52.6 (40.3 to 67.6)	1.9 (1.5 to 2.4)	118.9 (64.4 to 188.8)	1 370 (1 050 to 1 760)	47.0 (36.1 to 60.3)	101.1 (51.0 to 165.6)
Uzbekistan	267 (219 to 322)	1.9 (1.6 to 2.2)	278.2 (147.5 to 499.6)	339 (277 to 411)	2.2 (1.8 to 2.6)	325.3 (179.2 to 564.7)	8 530 (6 970 to 10 300)	50.6 (41.5 to 60.6)	289.1 (160.1 to 503.0)
**Central Europe**									
Albania	25.3 (18.2 to 33.9)	0.9 (0.7 to 1.3)	27.7 (-8.4 to 74.2)	35.4 (25.5 to 47.5)	1.3 (1.0 to 1.8)	61.7 (15.8 to 120.8)	603 (430 to 830)	23.5 (16.8 to 32.2)	11.8 (-21.4 to 56.3)
Bosnia and Herzegovina	64.6 (49.5 to 82.0)	1.8 (1.4 to 2.3)	33.7 (1.6 to 78.3)	83.9 (64.1 to 107)	2.3 (1.8 to 3.0)	50.1 (13.5 to 99.3)	1 710 (1 290 to 2 220)	48.7 (37.0 to 62.6)	15.5 (-13.3 to 54.3)
Bulgaria	157 (121 to 203)	2.0 (1.6 to 2.6)	59.5 (20.8 to 107.6)	219 (168 to 281)	3.0 (2.3 to 3.8)	80.5 (36.8 to 133.9)	4 570 (3 490 to 5 920)	64.3 (48.9 to 83.7)	50.3 (12.9 to 96.7)
Croatia	151 (113 to 199)	3.1 (2.3 to 4.1)	-7.0 (-31.9 to 26.7)	220 (164 to 289)	4.6 (3.4 to 6.1)	10.9 (-18.2 to 51.1)	4 090 (3 010 to 5 390)	89.3 (65.9 to 117.3)	-18.7 (-40.6 to 10.2)
Czechia	294 (230 to 374)	2.5 (2.0 to 3.2)	88.0 (44.5 to 144.7)	624 (491 to 790)	5.5 (4.3 to 7.0)	183.5 (119.2 to 267.9)	8 040 (6 270 to 10 300)	73.2 (56.8 to 93.8)	71.5 (31.5 to 124.8)
Hungary	576 (456 to 723)	5.6 (4.4 to 7.0)	57.8 (24.7 to 101.4)	930 (732 to 1 160)	9.2 (7.2 to 11.7)	103.5 (60.8 to 161.0)	16 900 (13 300 to 21 500)	173.5 (135.3 to 221.3)	41.1 (10.5 to 81.5)
Montenegro	6.73 (5.29 to 8.37)	1.1 (0.9 to 1.4)	47.8 (11.4 to 94.8)	10.2 (8.04 to 12.7)	1.7 (1.4 to 2.2)	69.3 (28.6 to 121.4)	198 (155 to 249)	34.1 (26.3 to 42.9)	36.0 (1.9 to 82.0)

© 2023 Cunha AR et al. *JAMA Oncol.*

Super-region, **region,** or country or territory	**Deaths, OPC**			**Incidence, OPC**			**DALYs, OPC**		
	**Counts, 2019 (95% UI)**	**Age-standardized rate, 2019 (95% UI)**	**% change of age-standardized rates, 1990–2019 (95% UI)**	**Counts, 2019 (95% UI)**	**Age-standardized rate, 2019 (95% UI)**	**% change of age-standardized rates, 1990–2019 (95% UI)**	**Counts, 2019 (95% UI)**	**Age-standardized rate, 2019 (95% UI)**	**% change of age-standardized rates, 1990–2019 (95% UI)**
North Macedonia	24.5 (18.6 to 31.7)	1.2 (0.9 to 1.6)	77.1 (33.1 to 133.4)	34.4 (26.1 to 44.5)	1.7 (1.3 to 2.2)	115.3 (60.0 to 185.8)	722 (540 to 941)	36.3 (27.0 to 47.6)	63.7 (20.8 to 116.6)
Poland	1 020 (828 to 1 260)	2.5 (2.0 to 3.1)	95.7 (58.0 to 143.4)	1 900 (1 530 to 2 360)	4.8 (3.8 to 6.0)	194.2 (135.5 to 266.3)	28 400 (22 800 to 35 100)	73.7 (59.0 to 91.6)	82.6 (46.4 to 129.1)
Romania	911 (717 to 1 110)	4.5 (3.5 to 5.5)	170.7 (112.7 to 233.5)	1 260 (989 to 1 550)	6.4 (5.0 to 7.9)	224.7 (155.0 to 300.5)	27 100 (21 100 to 33 100)	141.2 (109.3 to 174.9)	152.9 (95.5 to 212.5)
Serbia	246 (185 to 318)	2.7 (2.0 to 3.4)	21.4 (-12.4 to 68.3)	364 (274 to 472)	4.1 (3.0 to 5.3)	53.2 (11.3 to 108.5)	6 770 (5 040 to 8 820)	78.2 (57.7 to 102.0)	9.9 (-21.2 to 51.2)
Slovakia	305 (226 to 401)	5.5 (4.1 to 7.2)	77.3 (28.0 to 141.2)	393 (294 to 514)	7.2 (5.4 to 9.5)	89.5 (37.3 to 154.9)	8 890 (6 540 to 11 700)	166.3 (122.4 to 220.1)	63.3 (17.0 to 126.2)
Slovenia	94.1 (70.5 to 126)	3.9 (2.9 to 5.3)	45.9 (-1.7 to 118.3)	179 (134 to 242)	7.7 (5.7 to 10.6)	104.3 (36.4 to 205.0)	2 580 (1 930 to 3 490)	115.6 (85.6 to 154.9)	29.4 (-13.0 to 93.6)
**Eastern Europe**									
Belarus	295 (218 to 399)	3.0 (2.2 to 4.0)	112.6 (56.3 to 189.1)	416 (307 to 568)	4.3 (3.1 to 5.8)	158.1 (89.7 to 251.1)	8 770 (6 470 to 11 900)	91.5 (67.1 to 124.2)	102.9 (48.0 to 177.7)
Estonia	37.6 (28.0 to 49.0)	2.6 (2.0 to 3.5)	23.8 (-9.4 to 65.7)	56.6 (41.9 to 74.0)	4.1 (3.0 to 5.4)	57.6 (14.8 to 109.3)	1 020 (753 to 1 340)	76.4 (56.6 to 100.9)	10.8 (-19.4 to 49.7)
Latvia	58.4 (43.6 to 77.7)	2.7 (2.0 to 3.6)	37.4 (1.5 to 83.7)	77.8 (58.2 to 103)	3.7 (2.8 to 4.9)	57.3 (16.1 to 108.5)	1 600 (1 180 to 2 140)	79.6 (58.6 to 106.4)	24.2 (-9.6 to 67.9)
Lithuania	115 (89.7 to 147)	3.7 (2.9 to 4.7)	94.6 (50.2 to 145.4)	145 (113 to 185)	4.7 (3.7 to 6.1)	111.2 (62.5 to 167.6)	3 260 (2 520 to 4 200)	110.8 (85.3 to 143.1)	80.0 (37.3 to 129.4)
Republic of Moldova	145 (121 to 174)	4.0 (3.3 to 4.8)	86.0 (50.4 to 124.2)	175 (146 to 210)	4.8 (4.0 to 5.8)	99.7 (61.4 to 140.8)	4 400 (3 640 to 5 260)	123.1 (101.7 to 147.7)	74.8 (40.6 to 112.3)
Russian Federation	2 920 (2 410 to 3 540)	2.0 (1.6 to 2.4)	26.5 (4.0 to 52.1)	7 690 (6 310 to 9 280)	5.4 (4.4 to 6.5)	95.8 (61.5 to 136.7)	86 400 (71 700 to 105 000)	61.1 (50.6 to 74.2)	22.3 (0.7 to 47.5)
Ukraine	1 620 (1 250 to 2 030)	3.6 (2.8 to 4.5)	71.2 (15.5 to 150.5)	2 390 (1 900 to 2 990)	5.4 (4.3 to 6.7)	101.5 (38.9 to 196.6)	49 400 (38 100 to 62 400)	114.0 (87.5 to 144.9)	70.7 (13.7 to 153.3)
High-income									
**Australasia**									
Australia	346 (305 to 392)	1.4 (1.2 to 1.6)	37.6 (20.6 to 56.5)	594 (446 to 781)	2.5 (1.9 to 3.3)	70.8 (27.2 to 126.3)	8 500 (7 480 to 9 610)	36.6 (32.4 to 41.3)	27.3 (11.7 to 45.4)
New Zealand	40.9 (36.2 to 46.1)	0.9 (0.8 to 1.0)	65.7 (41.6 to 96.1)	87.7 (67.5 to 112)	1.9 (1.5 to 2.5)	122.9 (66.7 to 194.0)	1 000 (898 to 1 130)	22.8 (20.4 to 25.5)	62.3 (38.8 to 93.3)
**High-income Asia Pacific**									
Brunei Darussalam	5.81 (4.80 to 7.02)	3.3 (2.7 to 3.9)	281.2 (176.6 to 418.2)	8.04 (6.59 to 9.78)	4.1 (3.4 to 5.0)	354.8 (230.0 to 522.2)	161 (133 to 196)	74.8 (61.9 to 90.1)	278.8 (175.5 to 411.1)
Japan	2 700 (2 440 to 2 910)	1.3 (1.2 to 1.4)	248.5 (222.3 to 273.7)	5 710 (4 690 to 6 990)	3.1 (2.5 to 3.8)	350.2 (271.5 to 450.4)	53 400 (49 400 to 57 500)	30.3 (28.3 to 32.6)	168.1 (151.2 to 187.6)

Super-region, **region,** or country or territory	Deaths, OPC			Incidence, OPC			DALYs, OPC		
	**Counts, 2019 (95% UI)**	**Age-standardized rate, 2019 (95% UI)**	**% change of age-standardized rates, 1990–2019 (95% UI)**	**Counts, 2019 (95% UI)**	**Age-standardized rate, 2019 (95% UI)**	**% change of age-standardized rates, 1990–2019 (95% UI)**	**Counts, 2019 (95% UI)**	**Age-standardized rate, 2019 (95% UI)**	**% change of age-standardized rates, 1990–2019 (95% UI)**
Republic of Korea	497 (416 to 581)	0.9 (0.7 to 1.0)	340.7 (262.1 to 427.2)	1 090 (855 to 1 350)	1.9 (1.5 to 2.4)	704.1 (521.4 to 932.4)	11 900 (9 970 to 14 000)	20.7 (17.3 to 24.3)	265.5 (197.9 to 339.9)
Singapore	40.6 (34.3 to 48.3)	0.8 (0.7 to 1.0)	138.4 (97.1 to 187.3)	88.1 (67.1 to 116)	1.7 (1.3 to 2.3)	278.2 (186.2 to 397.9)	973 (823 to 1 160)	18.9 (16.0 to 22.5)	108.0 (70.8 to 153.6)
**High-income North America**									
Canada	399 (349 to 449)	0.9 (0.8 to 1.1)	72.2 (51.2 to 95.6)	1 020 (757 to 1 330)	2.5 (1.9 to 3.3)	140.3 (78.4 to 214.1)	9 610 (8 390 to 10 900)	24.2 (21.1 to 27.4)	56.7 (37.3 to 79.4)
Greenland	2.27 (1.71 to 2.86)	4.8 (3.6 to 5.9)	26.1 (-10.0 to 69.3)	2.91 (2.22 to 3.65)	6.0 (4.6 to 7.4)	38.3 (0.1 to 84.4)	64.6 (48.3 to 82.6)	129.0 (96.7 to 163.6)	9.8 (-23.7 to 49.7)
United States of America	3 110 (2 960 to 3 250)	0.9 (0.9 to 0.9)	55.5 (45.4 to 64.2)	14 200 (11 700 to 17 000)	4.4 (3.6 to 5.2)	97.3 (62.7 to 137.2)	81 500 (77 300 to 85 700)	25.2 (23.9 to 26.5)	51.1 (41.3 to 60.2)
**Southern Latin America**									
Argentina	233 (209 to 260)	0.7 (0.6 to 0.8)	-24.7 (-34.5 to -14.6)	303 (233 to 388)	0.9 (0.7 to 1.2)	-12.4 (-33.8 to 13.2)	6 110 (5 500 to 6 840)	18.9 (17.0 to 21.1)	-30.8 (-40.1 to -21.2)
Chile	90.9 (79.8 to 102)	0.6 (0.5 to 0.7)	63.1 (40.1 to 91.8)	137 (105 to 178)	0.9 (0.7 to 1.2)	114.9 (59.9 to 184.3)	2 120 (1 880 to 2 380)	14.0 (12.4 to 15.7)	44.9 (23.6 to 69.6)
Uruguay	47.8 (41.5 to 55.3)	1.5 (1.3 to 1.8)	-14.7 (-28.6 to 1.0)	64.4 (47.8 to 84.7)	2.1 (1.6 to 2.8)	-1.6 (-28.7 to 32.5)	1 220 (1 050 to 1 420)	41.7 (36.0 to 48.2)	-19.8 (-33.2 to -4.0)
**Western Europe**									
Andorra	1.23 (0.898 to 1.60)	1.4 (1.0 to 1.8)	87.7 (23.1 to 181.7)	3.76 (2.71 to 4.97)	4.3 (3.1 to 5.6)	186.8 (86.2 to 330.4)	36.6 (26.3 to 48.7)	41.3 (29.6 to 54.9)	85.5 (21.6 to 179.6)
Austria	222 (198 to 248)	2.2 (2.0 to 2.5)	113.6 (87.1 to 141.6)	525 (407 to 665)	5.5 (4.3 to 7.0)	205.7 (135.3 to 287.6)	5 900 (5 270 to 6 620)	62.9 (56.1 to 70.7)	93.9 (69.6 to 119.3)
Belgium	226 (202 to 250)	1.8 (1.6 to 2.0)	66.8 (48.0 to 88.4)	591 (442 to 762)	5.0 (3.7 to 6.5)	153.7 (87.6 to 232.2)	5 990 (5 350 to 6 670)	51.7 (46.3 to 57.5)	55.9 (37.3 to 76.7)
Cyprus	4.79 (4.05 to 5.63)	0.4 (0.3 to 0.5)	135.5 (85.4 to 203.8)	12.2 (10.2 to 14.6)	1.0 (0.9 to 1.2)	338.5 (240.8 to 463.3)	121 (102 to 142)	10.3 (8.7 to 12.1)	125.3 (77.1 to 189.9)
Denmark	152 (130 to 176)	2.3 (2.0 to 2.7)	151.9 (112.7 to 198.7)	451 (342 to 599)	7.3 (5.5 to 9.7)	285.9 (187.7 to 422.5)	3 930 (3 380 to 4 620)	65.0 (55.7 to 76.4)	142.2 (104.4 to 190.2)
Finland	59.2 (51.4 to 68.3)	0.9 (0.7 to 1.0)	66.2 (39.4 to 98.4)	165 (122 to 220)	2.6 (1.9 to 3.5)	163.1 (94.3 to 256.3)	1 440 (1 230 to 1 670)	23.5 (20.2 to 27.2)	50.1 (23.7 to 79.6)
France	2 060 (1 790 to 2 340)	2.8 (2.4 to 3.2)	-16.0 (-27.0 to -3.3)	5 410 (4 020 to 7 230)	7.9 (5.9 to 10.7)	31.9 (-2.3 to 76.8)	54 100 (46 800 to 62 000)	81.1 (70.0 to 93.2)	-25.3 (-36.1 to -13.1)
Germany	2 840 (2 520 to 3 180)	2.7 (2.4 to 3.1)	84.0 (61.6 to 108.8)	6 380 (4 880 to 8 400)	6.5 (4.9 to 8.5)	164.3 (100.3 to 253.2)	73 900 (65 400 to 82 600)	77.6 (68.8 to 86.9)	50.1 (31.0 to 71.2)
Greece	52.5 (47.7 to 57.9)	0.4 (0.4 to 0.4)	59.3 (40.9 to 78.9)	119 (91.5 to 153)	1.0 (0.8 to 1.3)	104.5 (53.0 to 166.2)	1 220 (1 100 to 1 340)	10.7 (9.7 to 11.8)	47.5 (30.7 to 65.9)
Iceland	1.91 (1.63 to 2.25)	0.6 (0.5 to 0.7)	62.8 (34.7 to 96.4)	6.00 (4.95 to 7.28)	1.9 (1.6 to 2.4)	143.7 (94.6 to 200.8)	51.9 (43.8 to 61.5)	17.0 (14.4 to 20.0)	61.5 (32.1 to 95.5)

© 2023 Cunha AR et al. *JAMA Oncol*.

Super-region, **region,** or country or territory	**Deaths, OPC**			**Incidence, OPC**			**DALYs, OPC**		
	**Counts, 2019 (95% UI)**	**Age-standardized rate, 2019 (95% UI)**	**% change of age-standardized rates, 1990–2019 (95% UI)**	**Counts, 2019 (95% UI)**	**Age-standardized rate, 2019 (95% UI)**	**% change of age-standardized rates, 1990–2019 (95% UI)**	**Counts, 2019 (95% UI)**	**Age-standardized rate, 2019 (95% UI)**	**% change of age-standardized rates, 1990–2019 (95% UI)**
Ireland	53.1 (46.3 to 61.1)	1.2 (1.0 to 1.3)	99.1 (70.2 to 134.3)	151 (113 to 202)	3.4 (2.5 to 4.5)	259.1 (169.2 to 385.1)	1 330 (1 150 to 1 530)	29.9 (25.9 to 34.5)	99.7 (69.9 to 137.0)
Israel	21.7 (19.1 to 24.6)	0.3 (0.3 to 0.3)	112.8 (80.2 to 147.4)	46.2 (34.7 to 60.4)	0.7 (0.5 to 0.9)	231.3 (143.5 to 334.4)	531 (466 to 601)	8.0 (7.1 to 9.0)	109.2 (78.6 to 142.5)
Italy	898 (823 to 974)	1.1 (1.1 to 1.2)	6.5 (-2.1 to 16.5)	2 140 (1 660 to 2 680)	2.9 (2.3 to 3.7)	49.9 (16.5 to 88.9)	21 900 (20 200 to 23 700)	31.2 (28.8 to 33.8)	-6.3 (-13.5 to 2.4)
Luxembourg	10.5 (8.93 to 12.7)	1.8 (1.5 to 2.1)	28.5 (4.6 to 57.5)	30.2 (24.0 to 38.7)	5.2 (4.1 to 6.6)	118.9 (67.4 to 186.7)	296 (248 to 360)	51.1 (43.0 to 61.9)	24.1 (1.3 to 53.4)
Malta	4.16 (3.57 to 4.88)	0.8 (0.7 to 0.9)	65.4 (36.5 to 101.1)	10.2 (8.45 to 12.5)	2.2 (1.8 to 2.7)	158.2 (104.1 to 226.6)	109 (93.8 to 127)	23.7 (20.5 to 28.1)	55.6 (28.9 to 88.7)
Monaco	0.341 (0.264 to 0.425)	0.7 (0.5 to 0.9)	87.3 (36.4 to 157.1)	0.997 (0.755 to 1.26)	2.2 (1.6 to 2.8)	166.9 (89.1 to 269.5)	8.84 (6.68 to 11.3)	20.0 (14.9 to 26.0)	85.6 (30.5 to 158.1)
Netherlands	249 (220 to 282)	1.2 (1.1 to 1.4)	112.7 (84.3 to 144.9)	723 (546 to 942)	3.8 (2.9 to 4.9)	220.7 (136.9 to 321.6)	6 230 (5 460 to 7 110)	33.1 (29.0 to 37.6)	100.0 (72.3 to 131.3)
Norway	45.9 (41.7 to 50.4)	0.8 (0.7 to 0.9)	24.1 (13.0 to 37.6)	173 (138 to 212)	3.2 (2.6 to 4.0)	118.7 (75.3 to 168.7)	1 140 (1 040 to 1 270)	21.5 (19.6 to 23.8)	22.9 (11.2 to 37.2)
Portugal	310 (273 to 348)	2.6 (2.3 to 2.9)	177.4 (140.0 to 216.8)	673 (495 to 888)	5.9 (4.3 to 7.9)	357.8 (227.7 to 511.7)	8 990 (7 840 to 10 200)	81.6 (71.4 to 92.8)	173.0 (133.4 to 214.3)
San Marino	0.308 (0.194 to 0.456)	0.9 (0.5 to 1.3)	72.7 (2.9 to 170.5)	0.854 (0.614 to 1.16)	2.5 (1.8 to 3.5)	139.4 (63.1 to 241.8)	8.13 (4.96 to 12.4)	24.7 (14.9 to 37.9)	65.3 (-3.4 to 166.4)
Spain	851 (751 to 957)	1.6 (1.4 to 1.8)	47.9 (29.2 to 69.2)	2 040 (1 520 to 2 700)	4.1 (3.1 to 5.5)	114.9 (58.1 to 185.6)	22 800 (20 100 to 25 900)	46.9 (41.4 to 53.3)	35.5 (17.5 to 55.8)
Sweden	117 (106 to 130)	1.0 (0.9 to 1.1)	38.7 (22.8 to 57.3)	294 (239 to 357)	2.7 (2.2 to 3.2)	96.4 (59.9 to 139.5)	2 640 (2 390 to 2 920)	24.3 (21.9 to 26.8)	38.0 (21.1 to 55.3)
Switzerland	160 (141 to 182)	1.6 (1.4 to 1.8)	200.5 (155.8 to 251.3)	437 (325 to 576)	4.6 (3.4 to 6.1)	317.8 (209.4 to 460.0)	3 900 (3 420 to 4 440)	41.5 (36.3 to 47.2)	175.2 (131.0 to 223.9)
United Kingdom	982 (935 to 1 020)	1.3 (1.3 to 1.4)	70.2 (63.1 to 79.0)	3 360 (2 710 to 4 150)	4.9 (4.0 to 6.1)	162.2 (112.6 to 224.9)	24 700 (23 700 to 25 700)	37.0 (35.6 to 38.6)	72.4 (64.8 to 81.2)
Latin America and Caribbean									
**Andean Latin America**									
Bolivia (Plurinational State of)	36.3 (27.2 to 47.0)	0.7 (0.5 to 0.9)	137.4 (70.5 to 226.7)	38.0 (28.3 to 49.6)	0.7 (0.5 to 0.9)	149.1 (78.9 to 246.7)	938 (682 to 1 240)	15.9 (11.7 to 20.9)	113.6 (51.4 to 205.1)
Ecuador	37.3 (28.8 to 48.7)	0.4 (0.3 to 0.5)	103.7 (56.6 to 165.6)	42.5 (32.9 to 55.7)	0.4 (0.3 to 0.6)	128.4 (74.3 to 199.9)	922 (701 to 1 210)	9.3 (7.2 to 12.3)	79.5 (34.1 to 138.9)
Peru	70.2 (51.8 to 94.1)	0.3 (0.3 to 0.5)	41.1 (0.9 to 99.1)	84.9 (62.1 to 114)	0.4 (0.3 to 0.6)	66.0 (17.1 to 135.6)	1 750 (1 270 to 2 420)	8.5 (6.2 to 11.6)	24.9 (-13.7 to 78.2)
**Caribbean**									

Super-region, **region,** or country or territory	Deaths, OPC			Incidence, OPC			DALYs, OPC		
	Counts, 2019 (95% UI)	Age-standardized rate, 2019 (95% UI)	% change of age-standardized rates, 1990–2019 (95% UI)	Counts, 2019 (95% UI)	Age-standardized rate, 2019 (95% UI)	% change of age-standardized rates, 1990–2019 (95% UI)	Counts, 2019 (95% UI)	Age-standardized rate, 2019 (95% UI)	% change of age-standardized rates, 1990–2019 (95% UI)
Antigua and Barbuda	1.07 (0.873 to 1.30)	1.7 (1.4 to 2.0)	33.8 (4.5 to 67.1)	1.28 (1.05 to 1.56)	2.0 (1.6 to 2.4)	52.5 (18.5 to 92.4)	27.1 (21.7 to 33.2)	40.0 (32.3 to 48.7)	40.3 (7.2 to 78.0)
Bahamas	6.35 (4.99 to 7.99)	2.5 (2.0 to 3.1)	67.2 (28.8 to 114.7)	7.51 (5.90 to 9.47)	2.9 (2.3 to 3.6)	78.3 (36.9 to 130.1)	181 (142 to 229)	66.3 (52.0 to 83.8)	63.0 (23.9 to 112.0)
Barbados	6.70 (5.32 to 8.17)	2.2 (1.7 to 2.6)	31.8 (3.9 to 62.0)	8.01 (6.29 to 9.82)	2.6 (2.1 to 3.2)	49.2 (16.5 to 83.7)	159 (125 to 197)	52.9 (41.7 to 65.6)	33.8 (3.4 to 66.7)
Belize	1.82 (1.53 to 2.14)	1.0 (0.9 to 1.2)	191.1 (134.1 to 253.7)	2.07 (1.75 to 2.44)	1.1 (0.9 to 1.3)	221.8 (157.8 to 298.8)	52.1 (43.6 to 61.6)	26.6 (22.3 to 31.3)	225.1 (159.6 to 299.5)
Bermuda	1.37 (1.11 to 1.68)	1.7 (1.4 to 2.1)	-0.9 (-23.0 to 26.1)	2.16 (1.74 to 2.68)	2.8 (2.2 to 3.4)	32.5 (2.5 to 71.5)	31.2 (24.9 to 39.2)	41.2 (32.8 to 51.6)	-12.8 (-32.6 to 13.5)
Cuba	224 (177 to 280)	1.9 (1.5 to 2.3)	49.1 (16.5 to 89.2)	308 (243 to 388)	2.6 (2.0 to 3.3)	78.8 (40.0 to 127.5)	5 610 (4 370 to 7 080)	48.0 (37.2 to 60.4)	49.2 (15.3 to 91.7)
Dominica	1.82 (1.41 to 2.31)	3.2 (2.5 to 4.0)	7.6 (-21.0 to 44.3)	1.92 (1.47 to 2.46)	3.4 (2.6 to 4.3)	11.1 (-18.9 to 49.8)	44.7 (33.7 to 58.3)	79.8 (60.6 to 103.9)	12.0 (-19.7 to 53.0)
Dominican Republic	150 (110 to 204)	2.6 (1.9 to 3.5)	266.3 (157.6 to 412.6)	160 (115 to 220)	2.7 (2.0 to 3.7)	293.3 (172.0 to 459.8)	3 630 (2 560 to 5 130)	60.2 (42.6 to 84.7)	246.1 (132.6 to 395.9)
Grenada	1.64 (1.40 to 1.91)	2.3 (2.0 to 2.7)	31.4 (7.6 to 59.9)	1.87 (1.60 to 2.19)	2.6 (2.2 to 3.0)	49.2 (22.4 to 82.3)	43.5 (37.0 to 50.8)	57.5 (49.1 to 67.2)	45.3 (18.2 to 78.9)
Guyana	4.36 (3.23 to 5.78)	1.1 (0.8 to 1.4)	23.9 (-12.7 to 69.7)	4.76 (3.52 to 6.32)	1.1 (0.8 to 1.5)	30.1 (-8.8 to 78.5)	128 (94.6 to 172)	28.7 (21.2 to 38.1)	25.8 (-12.4 to 72.4)
Haiti	72.6 (46.1 to 117)	1.7 (1.1 to 2.7)	57.3 (4.7 to 129.8)	74.1 (46.8 to 120)	1.6 (1.0 to 2.6)	59.5 (5.9 to 133.6)	2 090 (1 330 to 3 390)	42.1 (26.5 to 68.1)	55.5 (1.4 to 128.4)
Jamaica	19.0 (14.4 to 24.3)	1.0 (0.7 to 1.3)	46.6 (9.0 to 94.1)	22.1 (16.5 to 28.6)	1.2 (0.9 to 1.5)	58.6 (16.9 to 111.0)	487 (364 to 639)	25.7 (19.1 to 33.6)	49.9 (9.7 to 104.0)
Puerto Rico	44.8 (33.7 to 58.4)	1.1 (0.8 to 1.4)	-27.1 (-46.1 to -2.2)	64.9 (48.6 to 85.4)	1.6 (1.2 to 2.2)	-9.8 (-34.5 to 21.0)	1 030 (761 to 1 370)	27.3 (19.8 to 36.6)	-33.9 (-52.3 to -11.1)
Saint Kitts and Nevis	0.724 (0.577 to 0.878)	1.7 (1.4 to 2.0)	20.8 (-3.4 to 51.6)	0.937 (0.736 to 1.15)	2.1 (1.7 to 2.5)	46.1 (13.9 to 84.7)	19.9 (15.2 to 24.8)	42.0 (32.8 to 51.8)	37.3 (5.0 to 77.3)
Saint Lucia	2.36 (1.92 to 2.86)	1.7 (1.4 to 2.1)	66.0 (35.0 to 107.4)	2.72 (2.21 to 3.31)	2.0 (1.6 to 2.4)	83.5 (48.4 to 129.6)	62.3 (50.1 to 76.2)	44.3 (35.8 to 54.0)	67.6 (33.8 to 110.5)
Saint Vincent and the Grenadines	2.04 (1.75 to 2.40)	2.4 (2.1 to 2.8)	60.0 (30.9 to 93.8)	2.24 (1.91 to 2.64)	2.6 (2.2 to 3.0)	68.9 (37.9 to 105.2)	53.8 (45.4 to 63.5)	61.2 (51.9 to 72.0)	65.9 (34.7 to 103.2)
Suriname	3.50 (2.81 to 4.31)	0.9 (0.7 to 1.1)	82.8 (42.4 to 134.0)	3.81 (3.05 to 4.72)	1.0 (0.8 to 1.2)	94.4 (51.2 to 149.8)	94.6 (75.1 to 118)	23.5 (18.7 to 29.2)	82.3 (40.3 to 135.7)
Trinidad and Tobago	11.9 (8.74 to 15.6)	1.0 (0.8 to 1.3)	33.0 (-3.5 to 78.4)	13.6 (9.97 to 18.2)	1.2 (0.9 to 1.6)	43.0 (2.4 to 92.9)	320 (230 to 435)	27.4 (19.7 to 37.3)	28.2 (-9.4 to 73.7)
United States Virgin Islands	2.73 (2.22 to 3.30)	2.4 (1.9 to 2.9)	107.3 (57.8 to 167.4)	3.28 (2.65 to 3.96)	2.9 (2.3 to 3.5)	121.5 (67.3 to 188.8)	66.0 (52.6 to 80.2)	59.2 (47.1 to 72.4)	81.5 (35.1 to 141.0)
**Central Latin America**									
Colombia	139 (105 to 181)	0.4 (0.3 to 0.5)	71.4 (27.9 to 124.1)	175 (130 to 229)	0.5 (0.4 to 0.7)	101.2 (48.0 to 164.5)	3 220 (2 390 to 4 250)	9.6 (7.1 to 12.7)	44.5 (6.0 to 92.8)

Super-region, **region,** or country or territory	Deaths, OPC			Incidence, OPC			DALYs, OPC		
	**Counts, 2019 (95% UI)**	**Age-standardized rate, 2019 (95% UI)**	**% change of age-standardized rates, 1990–2019 (95% UI)**	**Counts, 2019 (95% UI)**	**Age-standardized rate, 2019 (95% UI)**	**% change of age-standardized rates, 1990–2019 (95% UI)**	**Counts, 2019 (95% UI)**	**Age-standardized rate, 2019 (95% UI)**	**% change of age-standardized rates, 1990–2019 (95% UI)**
Costa Rica	27.5 (20.5 to 36.2)	0.8 (0.6 to 1.1)	133.0 (70.5 to 213.4)	37.4 (27.8 to 49.1)	1.1 (0.9 to 1.5)	167.3 (95.0 to 260.1)	679 (502 to 899)	20.5 (15.2 to 27.1)	111.4 (54.8 to 186.7)
El Salvador	19.5 (14.6 to 25.4)	0.5 (0.4 to 0.7)	10.1 (-17.1 to 44.8)	22.3 (16.6 to 29.3)	0.6 (0.4 to 0.8)	23.7 (-7.7 to 62.8)	468 (345 to 620)	12.7 (9.3 to 16.8)	-2.6 (-28.2 to 29.0)
Guatemala	39.6 (30.9 to 49.8)	0.6 (0.5 to 0.7)	95.0 (48.2 to 155.7)	41.7 (32.4 to 52.8)	0.6 (0.5 to 0.7)	105.5 (54.0 to 169.4)	1 000 (778 to 1 280)	13.4 (10.4 to 17.1)	75.6 (32.4 to 133.3)
Honduras	34.7 (26.5 to 46.7)	0.9 (0.7 to 1.2)	282.0 (187.0 to 414.4)	37.3 (28.2 to 50.4)	1.0 (0.7 to 1.3)	301.0 (202.1 to 441.4)	917 (685 to 1 250)	22.4 (16.9 to 30.4)	245.5 (159.6 to 369.0)
Mexico	280 (235 to 327)	0.4 (0.3 to 0.4)	110.5 (77.2 to 146.8)	332 (280 to 392)	0.4 (0.4 to 0.5)	140.4 (102.3 to 182.7)	6 870 (5 740 to 8 090)	9.0 (7.5 to 10.6)	98.3 (65.2 to 135.3)
Nicaragua	17.1 (13.4 to 20.9)	0.6 (0.5 to 0.8)	171.1 (103.6 to 251.4)	19.9 (15.5 to 24.7)	0.7 (0.6 to 0.9)	212.3 (134.2 to 304.7)	417 (323 to 525)	14.4 (11.2 to 17.8)	151.5 (88.5 to 230.0)
Panama	24.4 (18.0 to 31.8)	0.9 (0.7 to 1.2)	81.9 (31.7 to 140.4)	30.3 (22.4 to 39.5)	1.2 (0.9 to 1.5)	114.5 (54.5 to 183.7)	596 (436 to 785)	22.6 (16.6 to 29.8)	83.9 (31.1 to 146.4)
Venezuela (Bolivarian Republic of)	160 (116 to 210)	0.9 (0.6 to 1.1)	146.4 (75.6 to 231.3)	196 (141 to 259)	1.0 (0.8 to 1.4)	183.0 (100.6 to 282.4)	4 230 (3 030 to 5 670)	22.0 (15.8 to 29.4)	136.1 (65.4 to 221.9)
**Tropical Latin America**									
Brazil	3 870 (3 600 to 4 120)	2.5 (2.3 to 2.7)	129.7 (112.4 to 146.3)	4 700 (4 400 to 4 970)	3.0 (2.9 to 3.2)	157.6 (139.2 to 175.2)	110 000 (103 000 to 117 000)	70.1 (65.6 to 74.8)	116.3 (100.1 to 132.4)
Paraguay	57.1 (41.2 to 75.2)	1.6 (1.2 to 2.1)	206.9 (111.9 to 326.7)	66.6 (47.4 to 88.3)	1.8 (1.3 to 2.4)	241.2 (134.3 to 377.6)	1 580 (1 120 to 2 120)	42.6 (30.3 to 57.0)	202.9 (104.4 to 331.2)
North Africa and Middle East									
**North Africa and Middle East**									
Afghanistan	55.5 (34.5 to 89.4)	0.7 (0.4 to 1.0)	50.2 (5.5 to 111.6)	58.6 (35.3 to 95.0)	0.7 (0.4 to 1.0)	56.9 (9.3 to 121.9)	1 840 (1 050 to 3 080)	17.5 (11.0 to 27.9)	66.8 (14.8 to 143.6)
Algeria	209 (158 to 269)	1.0 (0.7 to 1.2)	130.6 (58.3 to 220.3)	275 (208 to 351)	1.2 (0.9 to 1.5)	178.0 (91.3 to 287.1)	6 130 (4 600 to 7 920)	25.6 (19.3 to 32.8)	123.4 (50.4 to 215.1)
Bahrain	2.86 (2.10 to 3.82)	0.5 (0.4 to 0.6)	218.3 (114.8 to 355.5)	4.44 (3.31 to 5.91)	0.6 (0.5 to 0.8)	343.5 (201.2 to 536.2)	86.6 (63.8 to 117)	10.8 (8.1 to 14.1)	222.1 (116.0 to 363.3)
Egypt	153 (106 to 212)	0.4 (0.3 to 0.5)	155.4 (73.9 to 254.6)	182 (126 to 254)	0.4 (0.3 to 0.6)	190.2 (99.3 to 302.1)	4 440 (3 050 to 6 240)	9.6 (6.7 to 13.4)	152.1 (71.7 to 250.2)
Iran (Islamic Republic of)	145 (133 to 159)	0.3 (0.3 to 0.4)	123.4 (88.0 to 176.9)	198 (182 to 217)	0.4 (0.4 to 0.5)	176.0 (130.9 to 242.3)	3 700 (3 410 to 4 030)	7.5 (6.9 to 8.2)	98.8 (65.7 to 142.1)
Iraq	98.3 (72.7 to 124)	0.7 (0.5 to 0.8)	187.8 (90.0 to 318.2)	125 (91.3 to 159)	0.8 (0.6 to 1.0)	239.6 (120.1 to 396.1)	2 900 (2 100 to 3 770)	17.1 (12.6 to 21.7)	184.5 (81.3 to 320.2)
Jordan	18.7 (14.9 to 23.7)	0.5 (0.4 to 0.6)	318.4 (195.2 to 483.7)	26.2 (21.0 to 33.1)	0.6 (0.5 to 0.8)	433.6 (274.6 to 648.5)	526 (421 to 668)	11.1 (8.8 to 14.0)	294.9 (174.5 to 458.8)

Super-region, **region,** or country or territory	Deaths, OPC			Incidence, OPC			DALYs, OPC		
	**Counts, 2019 (95% UI)**	**Age-standardized rate, 2019 (95% UI)**	**% change of age-standardized rates, 1990–2019 (95% UI)**	**Counts, 2019 (95% UI)**	**Age-standardized rate, 2019 (95% UI)**	**% change of age-standardized rates, 1990–2019 (95% UI)**	**Counts, 2019 (95% UI)**	**Age-standardized rate, 2019 (95% UI)**	**% change of age-standardized rates, 1990–2019 (95% UI)**
Kuwait	6.59 (5.06 to 8.19)	0.4 (0.3 to 0.5)	127.7 (79.6 to 183.3)	12.1 (9.38 to 14.9)	0.7 (0.5 to 0.8)	202.5 (138.4 to 280.3)	187 (143 to 233)	9.3 (7.1 to 11.6)	100.1 (55.5 to 154.4)
Lebanon	19.0 (14.8 to 25.1)	0.6 (0.5 to 0.8)	80.8 (29.9 to 159.8)	34.3 (26.5 to 44.9)	1.0 (0.8 to 1.4)	185.7 (103.6 to 305.3)	517 (394 to 681)	15.8 (12.0 to 20.8)	67.5 (18.1 to 139.9)
Libya	13.6 (10.1 to 18.3)	0.4 (0.3 to 0.5)	155.2 (62.7 to 326.3)	17.8 (13.3 to 23.9)	0.5 (0.4 to 0.7)	201.4 (93.3 to 405.0)	401 (298 to 544)	10.6 (7.9 to 14.4)	160.5 (66.1 to 337.9)
Morocco	212 (154 to 271)	1.0 (0.8 to 1.3)	149.4 (72.7 to 243.9)	251 (181 to 321)	1.2 (0.9 to 1.5)	176.0 (90.0 to 283.0)	6 200 (4 420 to 8 060)	27.8 (19.9 to 35.8)	140.1 (63.0 to 239.0)
Oman	6.78 (4.94 to 9.50)	0.6 (0.5 to 0.8)	126.5 (37.2 to 284.7)	11.2 (8.20 to 15.6)	0.9 (0.7 to 1.1)	227.1 (98.0 to 450.2)	215 (153 to 309)	14.5 (10.8 to 20.0)	130.7 (35.9 to 296.1)
Palestine	4.19 (3.50 to 5.02)	0.3 (0.2 to 0.4)	135.0 (65.8 to 244.8)	5.30 (4.42 to 6.35)	0.3 (0.3 to 0.4)	178.0 (95.5 to 309.3)	116 (96.8 to 139)	6.9 (5.7 to 8.3)	148.4 (72.3 to 266.1)
Qatar	3.73 (2.51 to 5.44)	1.1 (0.7 to 1.5)	715.8 (382.5 to 1 255.3)	6.74 (4.58 to 9.75)	1.3 (0.9 to 1.9)	1 172.5 (654.4 to 2 001.2)	118 (78.9 to 173)	18.0 (12.3 to 25.8)	697.3 (373.2 to 1 229.0)
Saudi Arabia	66.7 (50.5 to 90.2)	0.5 (0.4 to 0.7)	217.9 (102.1 to 398.9)	117 (87.4 to 161)	0.8 (0.6 to 1.0)	484.3 (281.1 to 801.3)	2 310 (1 710 to 3 170)	13.5 (10.4 to 18.0)	263.4 (127.3 to 478.3)
Sudan	50.8 (35.1 to 72.1)	0.4 (0.3 to 0.6)	78.7 (22.7 to 153.5)	56.7 (37.6 to 80.6)	0.5 (0.3 to 0.6)	94.9 (29.4 to 179.9)	1 460 (984 to 2 100)	10.8 (7.3 to 15.4)	74.9 (16.4 to 162.8)
Syrian Arab Republic	25.7 (18.4 to 35.4)	0.3 (0.2 to 0.5)	120.1 (40.3 to 248.8)	33.4 (23.8 to 46.0)	0.4 (0.3 to 0.6)	167.1 (70.9 to 325.5)	726 (517 to 998)	8.4 (6.0 to 11.4)	111.6 (33.1 to 242.5)
Tunisia	62.3 (44.7 to 84.7)	0.8 (0.6 to 1.0)	139.6 (61.6 to 247.6)	93.4 (66.5 to 128)	1.1 (0.8 to 1.5)	217.8 (113.0 to 361.5)	1 730 (1 220 to 2 390)	20.5 (14.5 to 28.3)	129.3 (51.6 to 238.7)
Turkey	186 (145 to 237)	0.3 (0.3 to 0.4)	59.5 (14.9 to 122.6)	276 (214 to 352)	0.5 (0.4 to 0.6)	118.5 (55.8 to 204.5)	5 060 (3 940 to 6 510)	8.7 (6.7 to 11.0)	40.7 (-0.5 to 99.1)
United Arab Emirates	28.6 (15.1 to 50.2)	0.9 (0.4 to 1.4)	672.6 (355.6 to 1 365.6)	38.8 (20.6 to 68.3)	1.0 (0.5 to 1.6)	804.3 (433.7 to 1 609.8)	1 100 (585 to 1 960)	21.9 (11.3 to 36.7)	702.9 (367.4 to 1 434.0)
Yemen	38.8 (26.0 to 58.7)	0.5 (0.3 to 0.7)	142.3 (55.7 to 272.2)	42.0 (27.8 to 63.9)	0.5 (0.3 to 0.7)	153.5 (62.6 to 293.0)	1 150 (752 to 1 760)	11.7 (7.7 to 17.8)	136.7 (47.4 to 273.2)
South Asia									
**South Asia**									
Bangladesh	4 430 (3 030 to 6 410)	5.3 (3.7 to 7.7)	157.0 (61.2 to 274.5)	4 770 (3 260 to 6 960)	5.6 (3.8 to 8.0)	170.5 (66.6 to 295.6)	125 000 (84 300 to 186 000)	142.1 (96.2 to 209.4)	137.8 (45.5 to 257.6)
Bhutan	22.7 (14.1 to 31.4)	6.4 (4.0 to 8.7)	174.7 (80.1 to 303.3)	24.7 (15.1 to 34.3)	6.7 (4.2 to 9.3)	189.4 (89.1 to 329.6)	628 (374 to 890)	163.8 (98.8 to 230.4)	138.7 (52.7 to 264.1)
India	56 400 (46 800 to 67 700)	7.7 (6.4 to 9.2)	167.5 (102.9 to 237.9)	60 700 (50 400 to 72 200)	8.1 (6.7 to 9.6)	177.3 (110.3 to 246.1)	1 640 000 (1 350 000 to 1 960 000)	208.4 (172.1 to 250.4)	148.0 (89.5 to 213.7)
Nepal	796 (574 to 1 080)	5.6 (4.0 to 7.6)	183.2 (93.3 to 322.6)	836 (600 to 1 130)	5.7 (4.2 to 7.8)	191.3 (96.3 to 337.5)	22 200 (15 800 to 30 200)	146.0 (104.3 to 198.0)	151.3 (63.8 to 275.7)

Super-region, **region,** or country or territory	Deaths, OPC			Incidence, OPC			DALYs, OPC		
	**Counts, 2019 (95% UI)**	**Age-standardized rate, 2019 (95% UI)**	**% change of age-standardized rates, 1990–2019 (95% UI)**	**Counts, 2019 (95% UI)**	**Age-standardized rate, 2019 (95% UI)**	**% change of age-standardized rates, 1990–2019 (95% UI)**	**Counts, 2019 (95% UI)**	**Age-standardized rate, 2019 (95% UI)**	**% change of age-standardized rates, 1990–2019 (95% UI)**
Pakistan	4 610 (3 540 to 6 020)	6.0 (4.7 to 7.8)	151.0 (86.4 to 242.5)	4 950 (3 810 to 6 430)	6.2 (4.8 to 8.0)	164.3 (90.1 to 264.6)	148 000 (114 000 to 194 000)	173.8 (133.3 to 226.7)	165.9 (98.4 to 257.3)
Southeast Asia, East Asia, and Oceania									
**East Asia**									
China	5 590 (4 530 to 6 730)	0.4 (0.4 to 0.5)	87.0 (42.9 to 139.0)	10 100 (8 290 to 12 100)	0.8 (0.6 to 0.9)	205.3 (133.2 to 294.9)	145 000 (117 000 to 176 000)	11.0 (8.9 to 13.3)	64.2 (23.8 to 112.7)
Democratic People's Republic of Korea	89.5 (67.1 to 121)	0.4 (0.3 to 0.6)	48.0 (1.7 to 122.2)	109 (80.7 to 149)	0.5 (0.4 to 0.7)	46.1 (-0.3 to 122.4)	2 520 (1 830 to 3 530)	11.9 (8.6 to 16.5)	32.8 (-11.4 to 108.3)
Taiwan (Province of China)	928 (697 to 1 230)	3.8 (2.9 to 5.1)	394.8 (271.4 to 567.2)	2 340 (1 760 to 3 140)	9.8 (7.4 to 13.0)	704.0 (503.2 to 998.6)	28 500 (21 400 to 38 100)	120.5 (90.9 to 160.6)	394.2 (266.2 to 568.6)
**Oceania**									
American Samoa	0.200 (0.165 to 0.242)	0.7 (0.6 to 0.8)	159.2 (100.8 to 243.6)	0.232 (0.190 to 0.283)	0.8 (0.6 to 0.9)	165.8 (104.7 to 255.0)	5.66 (4.55 to 7.02)	17.7 (14.3 to 21.9)	146.4 (86.5 to 233.4)
Cook Islands	0.0527 (0.0426 to 0.0643)	0.3 (0.3 to 0.4)	62.1 (21.2 to 114.9)	0.0748 (0.0603 to 0.0920)	0.5 (0.4 to 0.6)	83.1 (35.7 to 145.7)	1.31 (1.04 to 1.62)	8.6 (6.8 to 10.7)	45.3 (5.5 to 98.2)
Fiji	2.99 (2.23 to 3.83)	0.6 (0.5 to 0.8)	113.9 (50.0 to 198.7)	3.33 (2.46 to 4.29)	0.7 (0.5 to 0.9)	117.5 (52.3 to 204.9)	88.4 (65.2 to 115)	16.6 (12.3 to 21.3)	101.7 (39.6 to 189.5)
Guam	0.760 (0.602 to 0.954)	0.6 (0.5 to 0.8)	223.1 (142.6 to 331.3)	0.995 (0.784 to 1.25)	0.8 (0.7 to 1.0)	224.9 (142.4 to 328.7)	21.9 (17.1 to 27.4)	18.5 (14.5 to 22.9)	216.0 (135.9 to 318.3)
Kiribati	0.482 (0.357 to 0.634)	1.0 (0.7 to 1.2)	72.9 (22.2 to 142.2)	0.525 (0.384 to 0.699)	1.0 (0.8 to 1.3)	79.2 (25.6 to 152.7)	17.5 (12.5 to 24.0)	30.2 (22.1 to 40.3)	83.6 (25.0 to 165.3)
Marshall Islands	0.152 (0.106 to 0.229)	0.7 (0.5 to 1.0)	104.4 (44.2 to 205.1)	0.168 (0.117 to 0.255)	0.7 (0.5 to 1.0)	114.7 (50.5 to 223.4)	5.02 (3.45 to 7.66)	18.5 (12.9 to 27.7)	117.1 (50.6 to 232.1)
Micronesia (Federated States of)	0.291 (0.196 to 0.451)	0.6 (0.5 to 1.0)	36.2 (-9.8 to 98.8)	0.330 (0.218 to 0.512)	0.7 (0.5 to 1.0)	46.6 (-4.1 to 114.6)	9.18 (5.85 to 14.6)	17.5 (11.5 to 27.2)	37.6 (-12.7 to 103.5)
Nauru	0.0211 (0.0141 to 0.0296)	0.7 (0.5 to 1.0)	-9.8 (-34.8 to 26.2)	0.0273 (0.0182 to 0.0384)	0.8 (0.6 to 1.1)	-1.1 (-28.6 to 39.2)	0.775 (0.501 to 1.11)	20.0 (13.8 to 27.4)	-5.6 (-32.5 to 33.7)
Niue	0.00733 (0.00575 to 0.00927)	0.5 (0.4 to 0.7)	-10.6 (-32.0 to 15.7)	0.00966 (0.00749 to 0.0122)	0.7 (0.6 to 0.9)	5.2 (-20.8 to 38.3)	0.190 (0.143 to 0.243)	14.1 (10.6 to 18.4)	-11.6 (-35.2 to 19.1)
Northern Mariana Islands	0.658 (0.534 to 0.802)	2.0 (1.6 to 2.4)	164.6 (88.5 to 249.8)	0.976 (0.777 to 1.20)	2.8 (2.3 to 3.4)	168.0 (87.8 to 261.5)	19.2 (15.1 to 24.0)	52.6 (42.3 to 64.8)	107.1 (39.2 to 189.3)
Palau	0.0423 (0.0324 to 0.0542)	0.3 (0.3 to 0.4)	79.3 (25.0 to 144.3)	0.0621 (0.0470 to 0.0801)	0.4 (0.3 to 0.6)	105.6 (39.8 to 184.5)	1.36 (1.00 to 1.77)	9.3 (6.9 to 12.0)	75.6 (17.2 to 150.0)

Super-region, **region,** or country or territory	**Deaths, OPC**			**Incidence, OPC**			**DALYs, OPC**		
	**Counts, 2019 (95% UI)**	**Age-standardized rate, 2019 (95% UI)**	**% change of age-standardized rates, 1990–2019 (95% UI)**	**Counts, 2019 (95% UI)**	**Age-standardized rate, 2019 (95% UI)**	**% change of age-standardized rates, 1990–2019 (95% UI)**	**Counts, 2019 (95% UI)**	**Age-standardized rate, 2019 (95% UI)**	**% change of age-standardized rates, 1990–2019 (95% UI)**
Papua New Guinea	14.8 (10.6 to 22.0)	0.5 (0.3 to 0.7)	177.1 (106.1 to 280.3)	16.0 (11.3 to 23.6)	0.5 (0.3 to 0.7)	180.6 (107.7 to 289.8)	477 (333 to 715)	12.7 (9.1 to 18.9)	178.3 (104.3 to 288.6)
Samoa	0.527 (0.406 to 0.665)	0.6 (0.4 to 0.7)	44.4 (4.3 to 96.7)	0.613 (0.463 to 0.785)	0.6 (0.5 to 0.8)	54.2 (11.2 to 112.2)	15.2 (11.3 to 19.8)	15.0 (11.2 to 19.4)	46.6 (2.5 to 109.2)
Solomon Islands	1.48 (0.988 to 2.56)	0.7 (0.5 to 1.2)	127.3 (61.3 to 215.0)	1.68 (1.10 to 2.95)	0.7 (0.5 to 1.3)	139.0 (69.1 to 232.1)	51.1 (32.3 to 93.7)	19.9 (13.4 to 34.6)	134.1 (64.6 to 231.6)
Tokelau	0.00391 (0.00290 to 0.00517)	0.5 (0.4 to 0.6)	-13.4 (-37.2 to 15.3)	0.00468 (0.00345 to 0.00623)	0.6 (0.4 to 0.7)	-1.0 (-29.1 to 33.8)	0.108 (0.0775 to 0.147)	12.7 (9.1 to 17.2)	-10.2 (-37.1 to 24.6)
Tonga	0.216 (0.152 to 0.299)	0.4 (0.3 to 0.6)	49.0 (11.0 to 101.6)	0.240 (0.168 to 0.334)	0.5 (0.3 to 0.7)	52.1 (12.5 to 107.5)	5.79 (3.99 to 8.19)	11.2 (7.8 to 15.9)	40.4 (1.9 to 94.5)
Tuvalu	0.0370 (0.0257 to 0.0540)	0.6 (0.4 to 0.8)	28.0 (-8.8 to 77.6)	0.0413 (0.0285 to 0.0607)	0.6 (0.4 to 0.9)	34.7 (-4.3 to 88.6)	1.07 (0.725 to 1.59)	15.6 (10.6 to 23.0)	23.2 (-13.9 to 75.5)
Vanuatu	0.625 (0.441 to 0.914)	0.6 (0.4 to 0.8)	172.1 (90.1 to 290.3)	0.660 (0.467 to 0.965)	0.6 (0.4 to 0.8)	174.9 (92.2 to 296.8)	18.9 (13.2 to 28.0)	15.1 (10.7 to 22.1)	167.9 (81.9 to 296.1)
**Southeast Asia**									
Cambodia	74.7 (56.9 to 94.5)	1.0 (0.8 to 1.3)	152.0 (74.1 to 253.5)	81.1 (61.7 to 104)	1.0 (0.8 to 1.3)	166.9 (84.0 to 275.0)	2 060 (1 550 to 2 650)	25.0 (19.0 to 32.0)	130.6 (55.7 to 227.6)
Indonesia	1 200 (838 to 2 050)	0.9 (0.6 to 1.5)	128.7 (71.3 to 193.3)	1 310 (934 to 2 150)	0.9 (0.7 to 1.5)	138.9 (79.2 to 209.7)	32 900 (22 900 to 56 700)	21.7 (15.2 to 37.3)	107.8 (54.9 to 170.9)
Lao People's Democratic Republic	23.7 (17.0 to 31.3)	0.9 (0.6 to 1.1)	48.5 (0.9 to 115.6)	25.0 (17.9 to 33.3)	0.9 (0.6 to 1.1)	54.2 (4.3 to 126.7)	679 (482 to 916)	21.8 (15.6 to 28.9)	40.8 (-5.6 to 109.5)
Malaysia	230 (172 to 298)	1.4 (1.0 to 1.7)	159.9 (86.5 to 251.1)	316 (235 to 411)	1.8 (1.3 to 2.3)	220.0 (127.7 to 337.9)	6 250 (4 640 to 8 210)	34.2 (25.5 to 44.7)	143.6 (73.8 to 232.7)
Maldives	2.14 (1.69 to 2.70)	1.2 (0.9 to 1.5)	230.7 (101.5 to 418.9)	3.17 (2.51 to 4.00)	1.6 (1.3 to 2.0)	359.9 (179.7 to 626.9)	56.7 (44.8 to 71.7)	26.5 (20.9 to 33.7)	209.3 (87.0 to 396.5)
Mauritius	10.0 (7.90 to 12.7)	0.9 (0.7 to 1.1)	276.1 (194.5 to 382.1)	13.8 (10.8 to 17.5)	1.2 (0.9 to 1.5)	324.9 (226.8 to 451.3)	276 (215 to 356)	23.9 (18.7 to 30.7)	251.3 (169.7 to 361.3)
Myanmar	227 (178 to 289)	0.8 (0.6 to 1.0)	58.1 (2.0 to 126.0)	244 (190 to 314)	0.8 (0.6 to 1.0)	66.0 (11.1 to 135.8)	6 240 (4 760 to 8 150)	19.6 (15.2 to 25.4)	43.5 (-7.8 to 112.2)
Philippines	347 (282 to 426)	0.7 (0.6 to 0.9)	100.0 (54.5 to 167.4)	389 (310 to 490)	0.8 (0.6 to 0.9)	103.7 (55.1 to 178.9)	9 850 (7 920 to 12 300)	18.0 (14.5 to 22.1)	89.0 (46.1 to 151.5)
Seychelles	4.45 (3.56 to 5.49)	6.2 (5.0 to 7.7)	230.9 (151.2 to 329.1)	5.69 (4.55 to 7.04)	7.7 (6.2 to 9.6)	287.9 (192.4 to 401.3)	125 (99.4 to 155)	162.6 (130.1 to 202.5)	240.8 (157.3 to 345.6)
Sri Lanka	384 (272 to 533)	2.3 (1.7 to 3.2)	69.5 (12.3 to 142.2)	549 (386 to 770)	3.3 (2.3 to 4.6)	122.4 (47.4 to 219.1)	9 810 (6 830 to 13 800)	58.0 (40.6 to 81.2)	65.6 (8.9 to 140.1)
Thailand	520 (374 to 706)	0.8 (0.6 to 1.1)	108.8 (45.5 to 199.5)	760 (542 to 1 040)	1.2 (0.8 to 1.6)	166.6 (83.2 to 284.8)	14 600 (10 400 to 20 200)	22.1 (15.8 to 30.4)	89.1 (28.9 to 174.9)

***Super-region*, region, or country or territory**	**Deaths, OPC**			**Incidence, OPC**			**DALYs, OPC**		
	**Counts, 2019 (95% UI)**	**Age-standardized rate, 2019 (95% UI)**	**% change of age-standardized rates, 1990–2019 (95% UI)**	**Counts, 2019 (95% UI)**	**Age-standardized rate, 2019 (95% UI)**	**% change of age-standardized rates, 1990–2019 (95% UI)**	**Counts, 2019 (95% UI)**	**Age-standardized rate, 2019 (95% UI)**	**% change of age-standardized rates, 1990–2019 (95% UI)**
Timor-Leste	4.22 (2.83 to 6.41)	0.8 (0.6 to 1.2)	183.2 (92.4 to 306.2)	4.40 (2.92 to 6.70)	0.8 (0.6 to 1.3)	183.5 (91.9 to 313.6)	111 (71.5 to 170)	20.4 (13.2 to 31.1)	132.3 (53.5 to 250.6)
Viet Nam	2 410 (1 740 to 3 200)	3.8 (2.8 to 5.0)	209.5 (112.0 to 333.5)	3 280 (2 350 to 4 380)	5.0 (3.7 to 6.6)	291.5 (168.0 to 452.9)	72 800 (51 300 to 97 600)	107.8 (77.0 to 144.2)	215.2 (111.0 to 354.4)
Sub-Saharan Africa									
**Central sub-Saharan Africa**									
Angola	70.7 (53.4 to 93.1)	0.9 (0.7 to 1.2)	196.0 (88.0 to 352.8)	73.7 (55.4 to 97.1)	0.9 (0.7 to 1.2)	206.2 (92.2 to 371.0)	2 200 (1 640 to 2 900)	25.3 (19.1 to 33.2)	191.7 (84.1 to 352.3)
Central African Republic	12.5 (8.79 to 18.7)	0.8 (0.6 to 1.2)	58.4 (8.8 to 125.6)	12.7 (8.88 to 19.0)	0.8 (0.6 to 1.2)	60.7 (10.5 to 129.6)	404 (281 to 620)	23.4 (16.5 to 34.9)	63.1 (11.3 to 135.7)
Congo	17.8 (12.9 to 26.3)	1.0 (0.8 to 1.5)	106.9 (32.8 to 224.2)	18.6 (13.5 to 27.4)	1.0 (0.8 to 1.5)	114.5 (36.0 to 237.6)	551 (392 to 818)	27.4 (19.8 to 40.4)	109.9 (29.5 to 245.6)
Democratic Republic of the Congo	158 (99.2 to 237)	0.7 (0.4 to 1.0)	104.7 (28.2 to 210.6)	163 (103 to 244)	0.7 (0.4 to 1.0)	108.8 (30.2 to 216.4)	4 850 (3 070 to 7 210)	17.9 (11.3 to 26.9)	109.4 (29.5 to 218.2)
Equatorial Guinea	3.22 (1.92 to 4.90)	1.0 (0.6 to 1.5)	201.6 (37.7 to 418.3)	3.43 (2.05 to 5.30)	1.1 (0.6 to 1.6)	221.8 (46.3 to 452.4)	96.1 (56.3 to 151)	27.0 (16.1 to 41.7)	194.1 (28.8 to 413.6)
Gabon	8.73 (6.00 to 11.8)	1.3 (0.9 to 1.7)	75.9 (9.8 to 171.8)	9.25 (6.33 to 12.6)	1.3 (0.9 to 1.7)	84.4 (14.5 to 187.8)	259 (174 to 357)	33.8 (23.1 to 46.2)	78.8 (9.3 to 188.1)
**Eastern sub-Saharan Africa**									
Burundi	41.4 (26.8 to 63.0)	1.3 (0.8 to 1.9)	62.5 (0.3 to 158.0)	43.0 (27.7 to 65.5)	1.3 (0.8 to 1.9)	67.1 (2.2 to 169.8)	1 340 (853 to 2 060)	37.0 (23.8 to 56.4)	67.4 (1.4 to 178.2)
Comoros	3.67 (2.40 to 5.37)	1.1 (0.8 to 1.7)	99.9 (20.9 to 316.1)	3.81 (2.46 to 5.60)	1.2 (0.8 to 1.7)	106.1 (23.3 to 343.1)	111 (70.2 to 167)	32.6 (20.6 to 48.4)	102.0 (17.5 to 383.3)
Djibouti	6.55 (4.25 to 10.1)	1.5 (1.0 to 2.3)	324.0 (165.2 to 557.0)	6.95 (4.36 to 10.9)	1.5 (1.1 to 2.3)	332.6 (169.4 to 573.7)	213 (131 to 336)	43.0 (28.2 to 66.2)	311.8 (151.7 to 555.6)
Eritrea	27.8 (19.1 to 37.0)	1.4 (1.0 to 1.9)	182.6 (93.7 to 321.9)	29.2 (20.1 to 39.3)	1.4 (1.0 to 1.9)	191.1 (96.5 to 335.5)	937 (635 to 1 260)	41.5 (28.6 to 55.2)	179.5 (86.9 to 323.1)
Ethiopia	150 (101 to 219)	0.5 (0.4 to 0.8)	65.3 (4.6 to 168.0)	156 (104 to 228)	0.5 (0.4 to 0.8)	68.2 (5.4 to 177.4)	4 620 (3 120 to 6 780)	15.0 (10.0 to 22.0)	55.9 (-2.1 to 163.2)
Kenya	228 (166 to 306)	1.5 (1.1 to 2.0)	300.8 (221.3 to 413.8)	233 (168 to 311)	1.4 (1.0 to 1.9)	290.2 (213.5 to 402.0)	7 380 (5 330 to 9 910)	42.2 (30.6 to 56.6)	311.2 (226.0 to 429.2)
Madagascar	82.5 (55.9 to 119)	1.0 (0.7 to 1.5)	96.3 (30.2 to 182.8)	86.5 (58.5 to 124)	1.0 (0.7 to 1.5)	101.3 (33.5 to 192.4)	2 730 (1 840 to 3 920)	30.1 (20.4 to 43.3)	103.4 (34.3 to 196.0)
Malawi	15.7 (11.2 to 21.5)	0.3 (0.2 to 0.4)	101.9 (42.4 to 193.3)	16.2 (11.5 to 22.3)	0.3 (0.2 to 0.4)	107.2 (45.4 to 200.9)	478 (331 to 665)	8.9 (6.3 to 12.3)	111.1 (46.0 to 207.2)
Mozambique	71.6 (52.9 to 95.6)	1.0 (0.7 to 1.3)	147.6 (68.2 to 259.6)	74.0 (54.2 to 99.8)	1.0 (0.7 to 1.3)	153.9 (71.0 to 271.6)	2 240 (1 610 to 3 060)	27.1 (19.8 to 36.4)	151.0 (64.9 to 274.1)

Super-region, **region,** or country or territory	**Deaths, OPC**			**Incidence, OPC**			**DALYs, OPC**		
	**Counts, 2019 (95% UI)**	**Age-standardized rate, 2019 (95% UI)**	**% change of age-standardized rates, 1990–2019 (95% UI)**	**Counts, 2019 (95% UI)**	**Age-standardized rate, 2019 (95% UI)**	**% change of age-standardized rates, 1990–2019 (95% UI)**	**Counts, 2019 (95% UI)**	**Age-standardized rate, 2019 (95% UI)**	**% change of age-standardized rates, 1990–2019 (95% UI)**
Rwanda	52.5 (36.6 to 73.0)	1.3 (0.9 to 1.7)	57.2 (2.9 to 158.8)	55.6 (38.6 to 77.5)	1.3 (0.9 to 1.8)	63.0 (6.4 to 171.3)	1 650 (1 120 to 2 350)	35.9 (25.0 to 49.9)	55.8 (-0.4 to 162.7)
Somalia	44.9 (26.8 to 76.6)	0.9 (0.6 to 1.6)	115.1 (37.7 to 232.2)	45.2 (26.3 to 77.4)	0.9 (0.5 to 1.6)	111.1 (31.6 to 224.7)	1 500 (891 to 2 580)	27.9 (16.5 to 47.0)	112.4 (35.6 to 227.5)
South Sudan	34.6 (19.2 to 51.8)	1.3 (0.7 to 1.9)	33.4 (-14.2 to 107.7)	35.3 (19.6 to 52.7)	1.3 (0.7 to 1.9)	36.0 (-12.0 to 112.3)	1 080 (593 to 1 660)	36.3 (20.1 to 54.4)	36.8 (-14.4 to 113.3)
Uganda	247 (182 to 321)	2.5 (1.9 to 3.2)	182.3 (99.9 to 287.9)	262 (193 to 343)	2.6 (1.9 to 3.3)	196.1 (108.2 to 308.7)	8 000 (5 820 to 10 700)	72.7 (53.4 to 94.9)	193.5 (100.4 to 316.1)
United Republic of Tanzania	233 (162 to 323)	1.4 (1.0 to 1.9)	116.8 (56.7 to 197.7)	245 (170 to 341)	1.4 (1.0 to 2.0)	123.7 (61.0 to 207.9)	7 310 (4 980 to 10 400)	39.7 (27.3 to 55.5)	121.0 (57.0 to 212.2)
Zambia	77.8 (56.5 to 105)	1.6 (1.2 to 2.2)	130.9 (61.0 to 218.7)	83.1 (60.5 to 112)	1.7 (1.3 to 2.2)	141.9 (70.0 to 236.7)	2 570 (1 840 to 3 490)	47.9 (34.7 to 64.5)	140.0 (65.9 to 237.1)
**Southern sub-Saharan Africa**									
Botswana	14.8 (9.78 to 20.4)	1.6 (1.1 to 2.1)	201.5 (95.0 to 335.5)	16.9 (11.2 to 23.5)	1.7 (1.2 to 2.3)	231.1 (111.7 to 382.1)	470 (300 to 665)	44.2 (29.0 to 61.1)	212.8 (96.1 to 361.1)
Eswatini	5.43 (3.72 to 7.51)	1.4 (1.0 to 1.9)	125.7 (51.7 to 237.2)	5.74 (3.90 to 8.00)	1.4 (1.0 to 2.0)	130.6 (53.1 to 243.7)	171 (114 to 243)	40.1 (27.5 to 56.3)	127.3 (46.6 to 246.6)
Lesotho	12.7 (9.22 to 16.9)	1.5 (1.1 to 2.0)	116.2 (45.1 to 219.5)	13.1 (9.36 to 17.4)	1.5 (1.1 to 2.0)	118.1 (45.5 to 226.9)	392 (276 to 526)	43.0 (30.6 to 57.5)	124.2 (44.9 to 243.2)
Namibia	21.8 (16.3 to 29.2)	2.3 (1.7 to 3.0)	140.4 (61.4 to 271.4)	24.0 (17.9 to 32.4)	2.5 (1.9 to 3.3)	157.7 (70.8 to 301.4)	682 (498 to 933)	67.3 (49.8 to 90.9)	146.6 (60.9 to 292.8)
South Africa	280 (244 to 321)	1.0 (0.8 to 1.1)	116.9 (89.4 to 147.3)	309 (269 to 356)	1.0 (0.9 to 1.2)	122.3 (91.3 to 155.5)	8 190 (7 040 to 9 430)	26.3 (22.6 to 30.2)	100.0 (71.5 to 129.8)
Zimbabwe	45.4 (34.2 to 58.0)	0.9 (0.7 to 1.2)	123.0 (61.4 to 197.0)	48.8 (36.9 to 62.4)	1.0 (0.7 to 1.2)	125.3 (63.6 to 199.1)	1 480 (1 100 to 1 930)	27.6 (20.7 to 35.5)	139.0 (68.8 to 223.1)
**Western sub-Saharan Africa**									
Benin	15.4 (11.3 to 20.5)	0.5 (0.4 to 0.6)	140.3 (72.0 to 232.4)	15.7 (11.4 to 21.0)	0.5 (0.4 to 0.6)	147.5 (74.1 to 244.1)	445 (314 to 607)	12.7 (9.1 to 17.0)	148.4 (73.1 to 251.1)
Burkina Faso	29.8 (22.7 to 39.1)	0.5 (0.4 to 0.7)	120.4 (63.9 to 193.3)	30.5 (23.2 to 40.1)	0.5 (0.4 to 0.7)	125.6 (67.7 to 201.4)	873 (652 to 1 150)	13.2 (10.0 to 17.3)	126.4 (66.8 to 207.4)
Cabo Verde	2.59 (2.07 to 3.22)	0.9 (0.8 to 1.2)	444.3 (319.1 to 597.0)	2.91 (2.29 to 3.66)	1.0 (0.8 to 1.3)	512.2 (365.8 to 692.3)	72.3 (55.9 to 92.4)	24.6 (19.3 to 31.2)	504.1 (350.9 to 699.8)
Cameroon	49.0 (35.4 to 68.7)	0.6 (0.5 to 0.9)	163.0 (82.5 to 288.7)	51.1 (36.7 to 72.0)	0.6 (0.5 to 0.9)	171.4 (88.6 to 305.5)	1 460 (1 030 to 2 070)	16.6 (12.0 to 23.5)	165.1 (80.3 to 302.7)
Chad	19.4 (14.2 to 26.1)	0.5 (0.4 to 0.7)	154.5 (85.6 to 257.0)	19.5 (14.3 to 26.3)	0.5 (0.4 to 0.7)	159.4 (87.9 to 266.7)	558 (407 to 765)	13.9 (10.2 to 18.9)	164.3 (88.3 to 279.7)
Côte d'Ivoire	40.6 (29.8 to 54.5)	0.6 (0.5 to 0.8)	133.0 (55.7 to 244.7)	42.1 (30.5 to 57.0)	0.6 (0.4 to 0.8)	134.8 (56.3 to 249.1)	1 230 (870 to 1 700)	15.2 (11.1 to 20.4)	126.3 (47.4 to 243.0)
Gambia	3.04 (2.12 to 4.19)	0.5 (0.3 to 0.7)	207.2 (93.3 to 392.3)	3.14 (2.19 to 4.32)	0.5 (0.3 to 0.7)	210.5 (93.6 to 399.7)	90.5 (61.9 to 128)	13.3 (9.2 to 18.5)	200.3 (81.9 to 386.4)

***Super-region*, region, or country or territory**	**Deaths, OPC**			**Incidence, OPC**			**DALYs, OPC**		
	**Counts, 2019 (95% UI)**	**Age-standardized rate, 2019 (95% UI)**	**% change of age-standardized rates, 1990–2019 (95% UI)**	**Counts, 2019 (95% UI)**	**Age-standardized rate, 2019 (95% UI)**	**% change of age-standardized rates, 1990–2019 (95% UI)**	**Counts, 2019 (95% UI)**	**Age-standardized rate, 2019 (95% UI)**	**% change of age-standardized rates, 1990–2019 (95% UI)**
Ghana	37.4 (29.8 to 47.1)	0.4 (0.3 to 0.5)	154.7 (87.8 to 253.4)	38.4 (30.4 to 48.7)	0.4 (0.3 to 0.5)	163.4 (93.1 to 268.4)	1 030 (802 to 1 330)	9.1 (7.2 to 11.6)	152.7 (81.9 to 253.2)
Guinea	26.7 (19.9 to 35.8)	0.8 (0.6 to 1.0)	92.4 (35.4 to 171.9)	27.0 (20.1 to 36.6)	0.7 (0.6 to 1.0)	96.4 (37.5 to 179.1)	775 (572 to 1 060)	20.1 (14.9 to 27.1)	98.3 (37.3 to 179.2)
Guinea-Bissau	2.97 (2.09 to 4.06)	0.6 (0.5 to 0.8)	57.5 (3.4 to 145.6)	3.10 (2.18 to 4.25)	0.6 (0.5 to 0.9)	60.8 (5.2 to 152.3)	91.5 (63.6 to 127)	16.9 (11.8 to 23.2)	61.5 (4.2 to 156.5)
Liberia	5.92 (3.71 to 8.58)	0.5 (0.3 to 0.7)	52.7 (-0.2 to 126.3)	6.10 (3.80 to 8.88)	0.4 (0.3 to 0.6)	59.4 (3.8 to 138.1)	176 (106 to 261)	11.6 (7.2 to 16.9)	65.1 (4.8 to 154.2)
Mali	28.2 (20.6 to 38.6)	0.5 (0.4 to 0.7)	119.2 (53.8 to 212.3)	29.2 (21.3 to 40.3)	0.5 (0.4 to 0.7)	123.6 (55.6 to 218.1)	861 (614 to 1 200)	13.6 (9.9 to 18.7)	119.0 (49.9 to 219.0)
Mauritania	6.08 (4.01 to 10.2)	0.5 (0.3 to 0.8)	62.4 (4.4 to 133.8)	6.22 (4.04 to 10.5)	0.5 (0.3 to 0.8)	66.1 (4.8 to 142.1)	162 (101 to 285)	11.4 (7.3 to 19.8)	52.2 (-8.0 to 129.2)
Niger	19.5 (13.5 to 28.4)	0.4 (0.3 to 0.6)	148.6 (75.4 to 241.6)	19.8 (13.5 to 29.1)	0.4 (0.3 to 0.6)	150.1 (75.2 to 246.5)	564 (384 to 839)	10.0 (6.9 to 14.6)	138.0 (64.0 to 236.0)
Nigeria	179 (135 to 238)	0.3 (0.3 to 0.4)	120.5 (56.1 to 217.2)	182 (137 to 243)	0.3 (0.3 to 0.4)	125.4 (56.3 to 231.9)	5 000 (3 680 to 6 780)	8.0 (6.0 to 10.6)	119.8 (51.6 to 220.3)
São Tomé and Príncipe	0.307 (0.227 to 0.405)	0.5 (0.3 to 0.6)	111.6 (49.5 to 192.4)	0.325 (0.238 to 0.429)	0.5 (0.4 to 0.6)	125.1 (58.7 to 213.3)	8.61 (6.19 to 11.5)	11.6 (8.5 to 15.4)	124.2 (55.4 to 220.5)
Senegal	25.1 (18.2 to 33.6)	0.5 (0.4 to 0.7)	126.8 (58.1 to 223.0)	25.4 (18.3 to 34.4)	0.5 (0.4 to 0.7)	130.5 (59.8 to 231.5)	698 (490 to 964)	13.3 (9.6 to 18.1)	122.2 (53.1 to 223.6)
Sierra Leone	11.1 (7.95 to 15.2)	0.5 (0.4 to 0.7)	82.2 (25.6 to 171.1)	11.3 (7.99 to 15.6)	0.5 (0.3 to 0.7)	88.8 (29.1 to 181.8)	319 (220 to 447)	12.5 (8.7 to 17.3)	92.0 (30.1 to 190.9)
Togo	12.2 (8.54 to 17.3)	0.5 (0.4 to 0.7)	184.1 (96.9 to 311.2)	12.8 (8.91 to 18.2)	0.5 (0.4 to 0.7)	190.9 (99.3 to 323.1)	371 (254 to 537)	13.4 (9.4 to 19.0)	188.2 (93.2 to 327.5)

SDI=Socio-demographic Index; DALYs=disability-adjusted life-years; UI=uncertainty interval. See eFigure 3 (p53) and eTable 7 (p54) for details and definitions of the SDI regions.

A

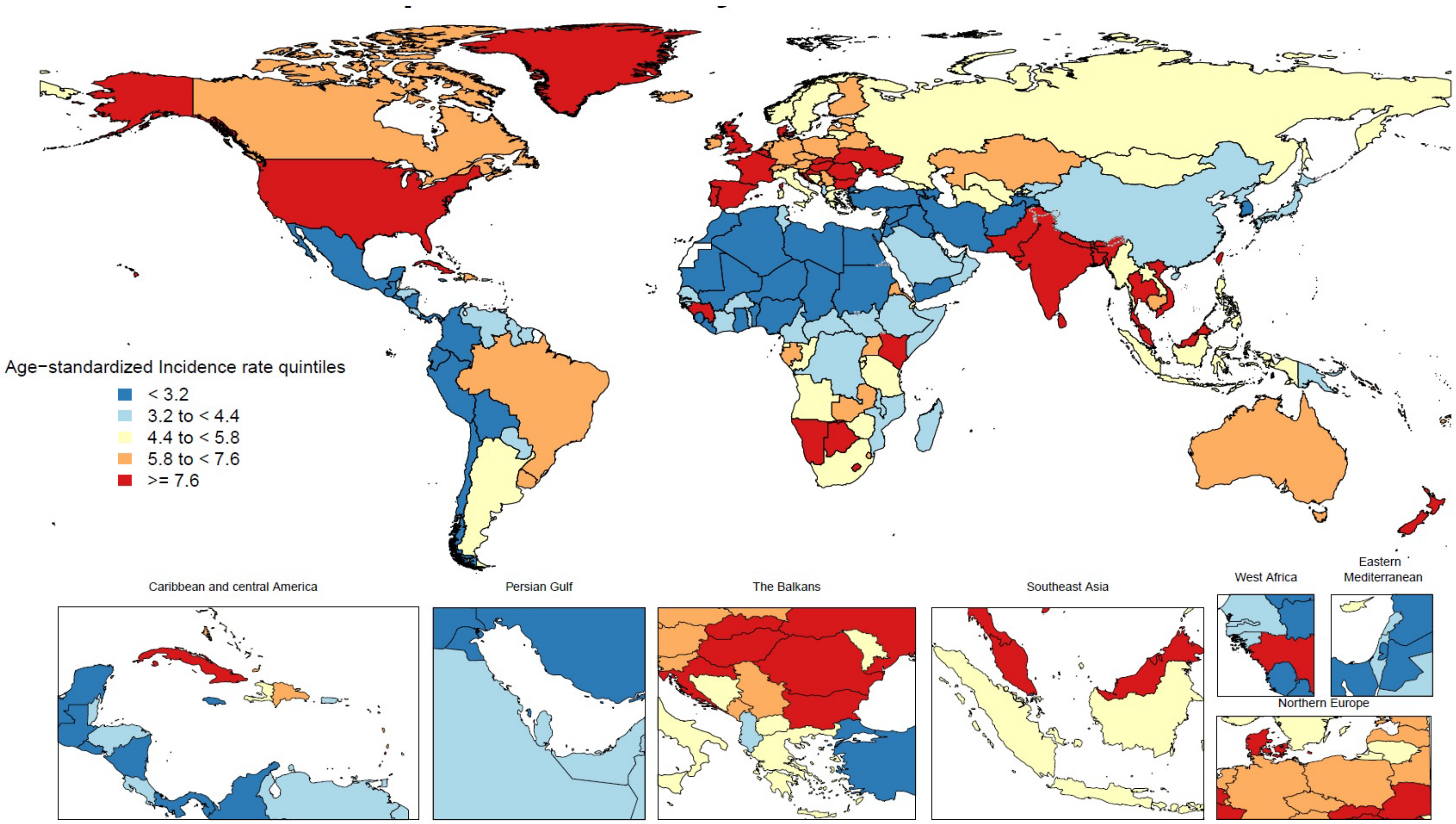
Age−standardized Incidence rate quintiles
< 3.2
3.2 to < 4.4
4.4 to < 5.8
5.8 to < 7.6
>= 7.6
Caribbean and central America
Persian Gulf
The Balkans
Southeast Asia
West Africa
Eastern Mediterranean
Northern Europe

B

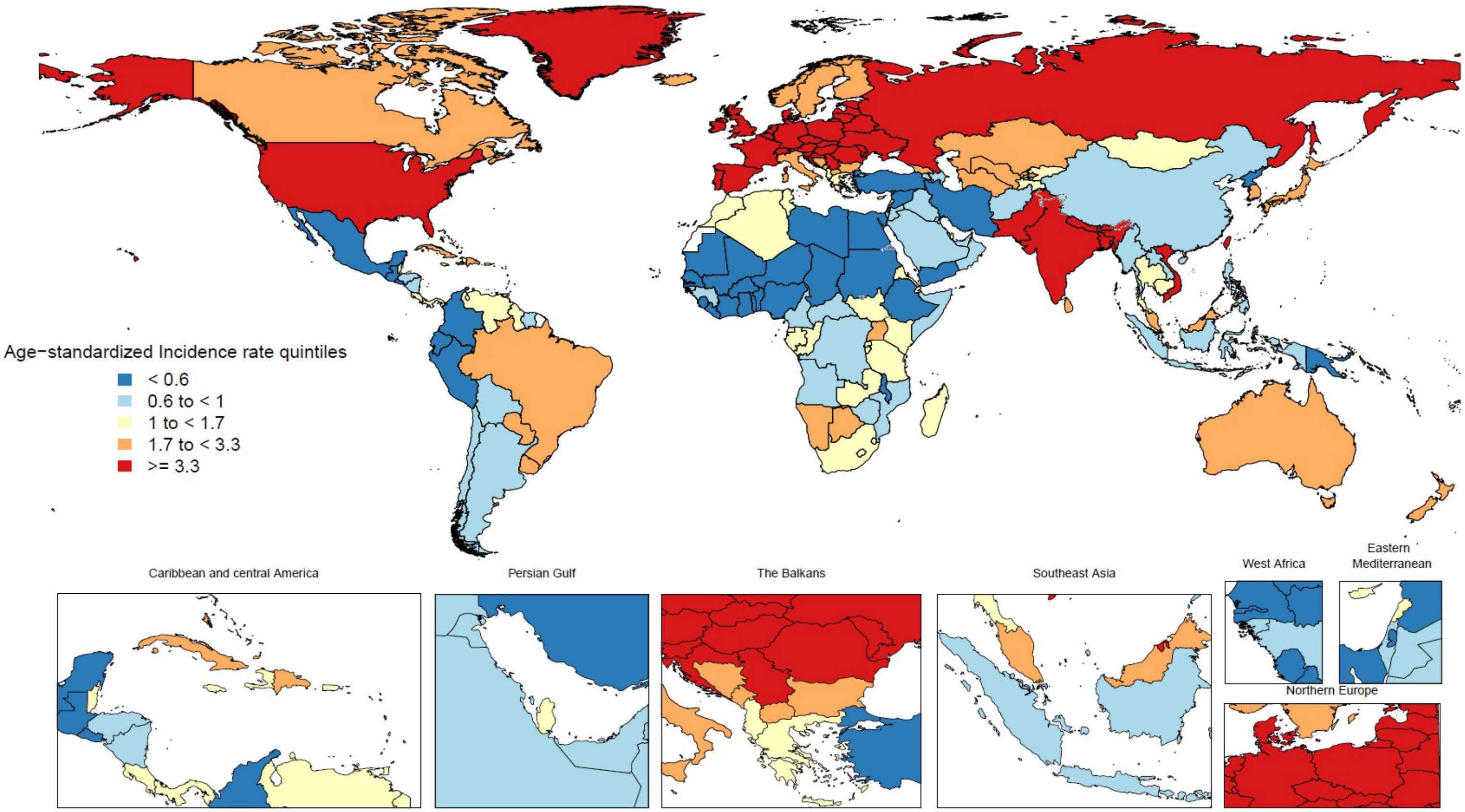

eFigure 6. Global map of age-standardized incidence rate quintiles for A) lip and oral cavity cancer, and B) other pharynx cancer, both sexes combined, 2019

Each map represents estimates at the national level and for the age range 20 to 95+ years. Quintiles are based on age-standardized incidence rates per 100,000 person-years. There are several geographic locations where estimates are not available (e.g., Western Sahara, French Guiana) as they were not modelled locations in the Global Burden of Diseases, Injuries, and Risk Factors 2019 study; these locations are white in this map.

A

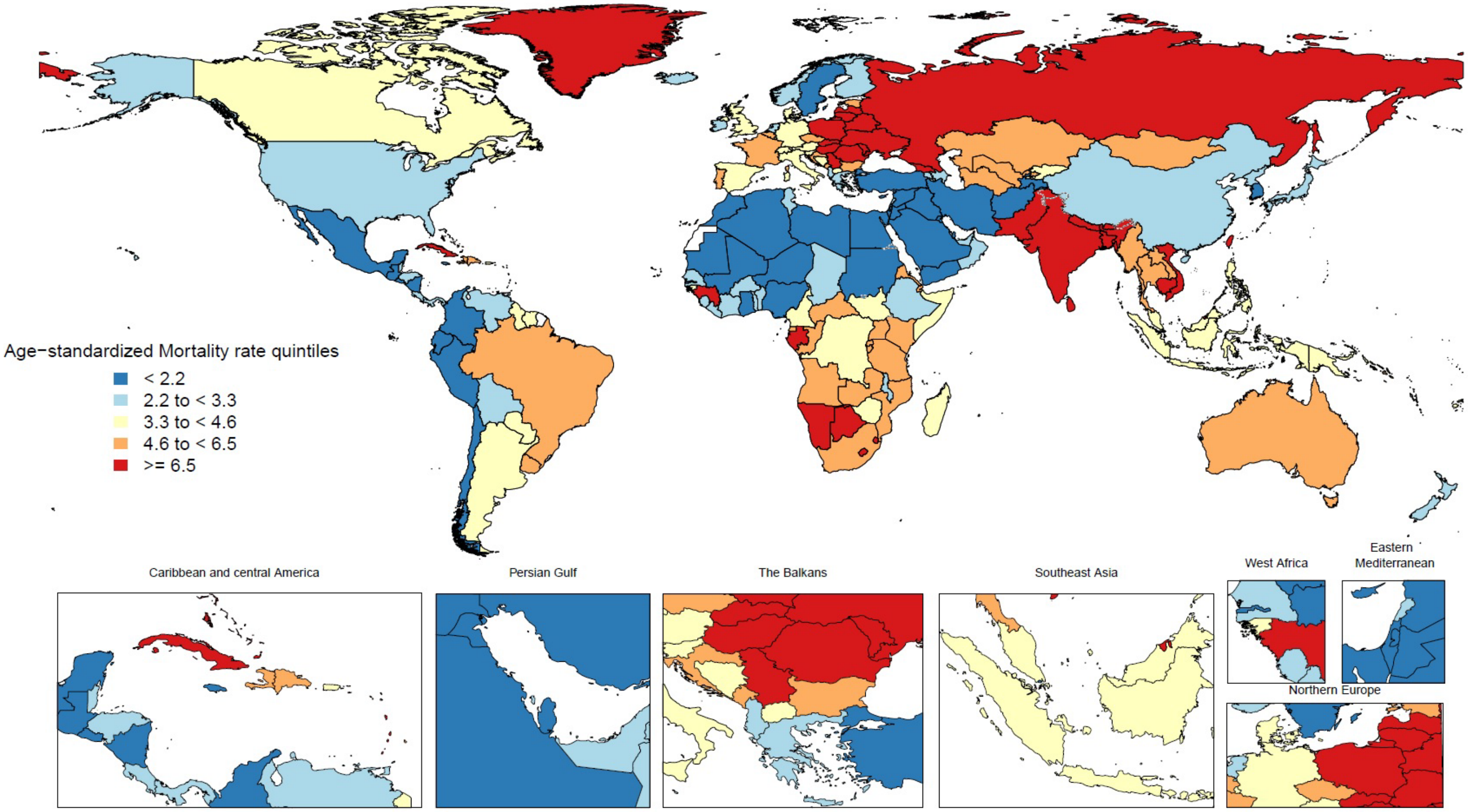
Age−standardized Mortality rate quintiles
< 2.2
2.2 to < 3.3
3.3 to < 4.6
4.6 to < 6.5
>= 6.5
Caribbean and central America
Persian Gulf
The Balkans
Southeast Asia
West Africa
Eastern Mediterranean
Northern Europe

B

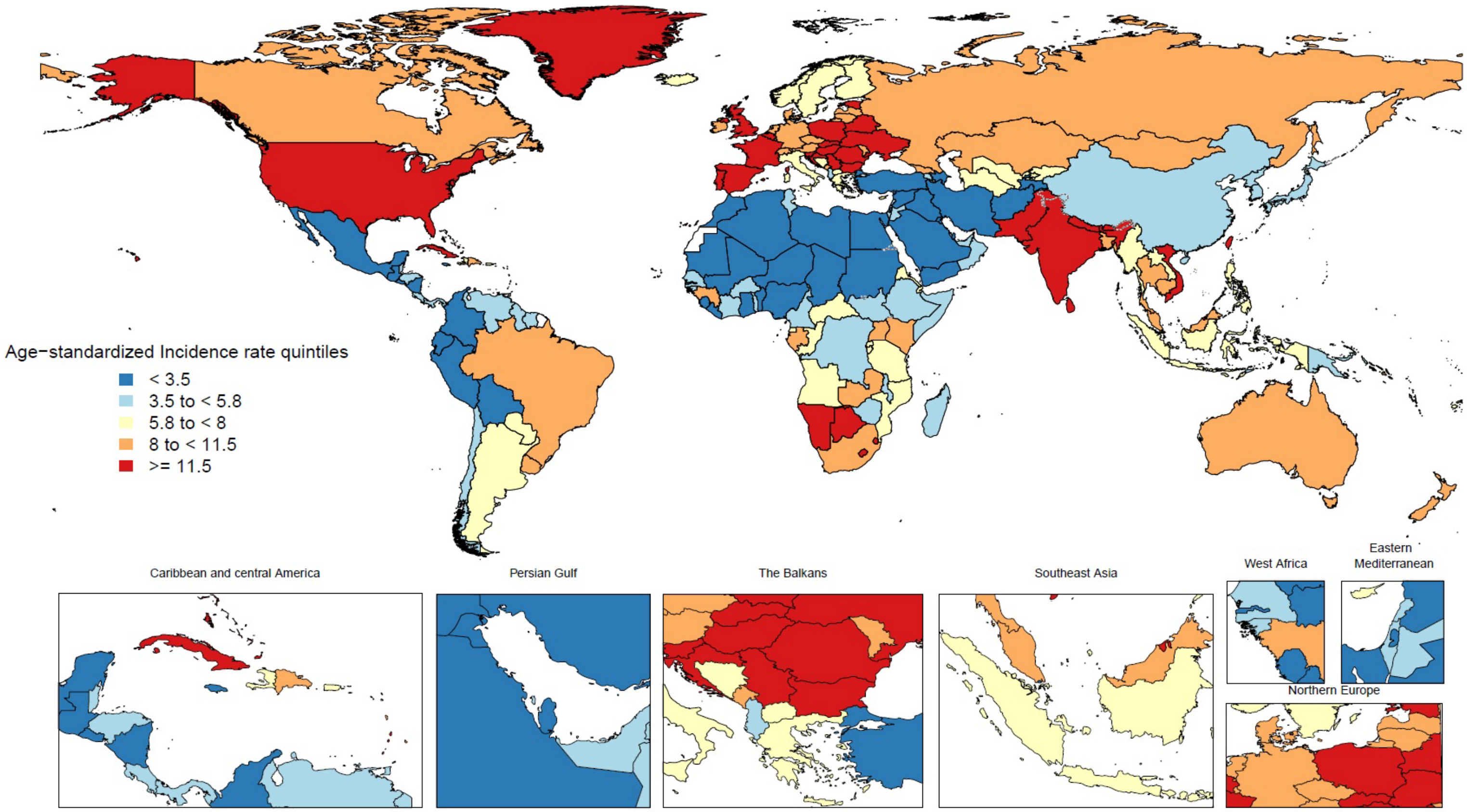

eFigure 7. Global map of A) age-standardized mortality rate quintiles, and B) age-standardized incidence rate quintiles for lip and oral cavity cancer, males, 2019

Each map represents estimates at the national level and for the age range 20 to 95+ years. Quintiles are based on age-standardized mortality and incidence rates per 100,000 person-years. There are several geographic locations where estimates are not available (e.g., Western Sahara, French Guiana) as they were not modelled locations in the Global Burden of Diseases, Injuries, and Risk Factors 2019 study; these locations are white in this map.

A

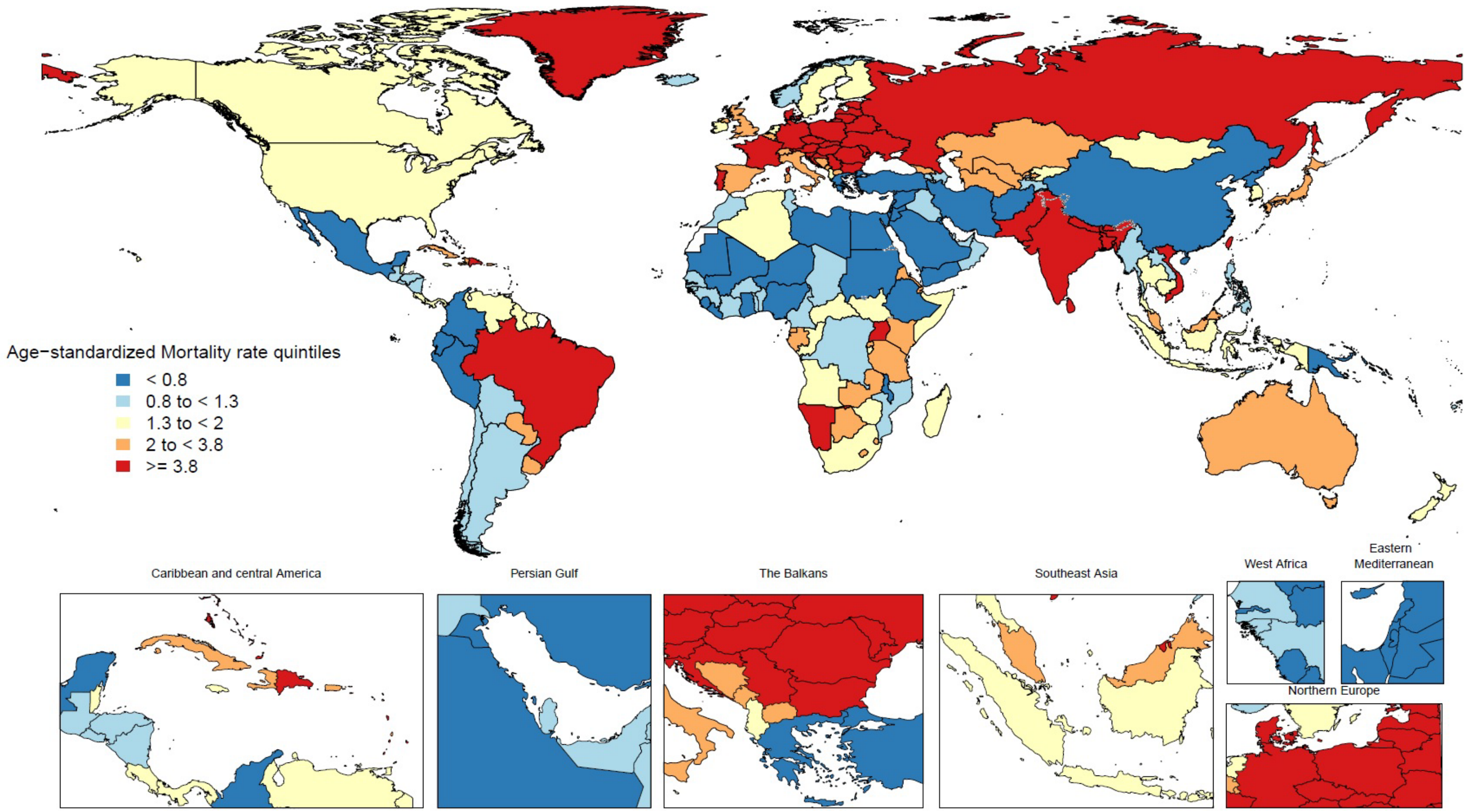

Age−standardized Mortality rate quintiles
< 0.8
0.8 to < 1.3
1.3 to < 2
2 to < 3.8
>= 3.8
Caribbean and central America
Persian Gulf
The Balkans
Southeast Asia
West Africa
Eastern Mediterranean
Northern Europe

B

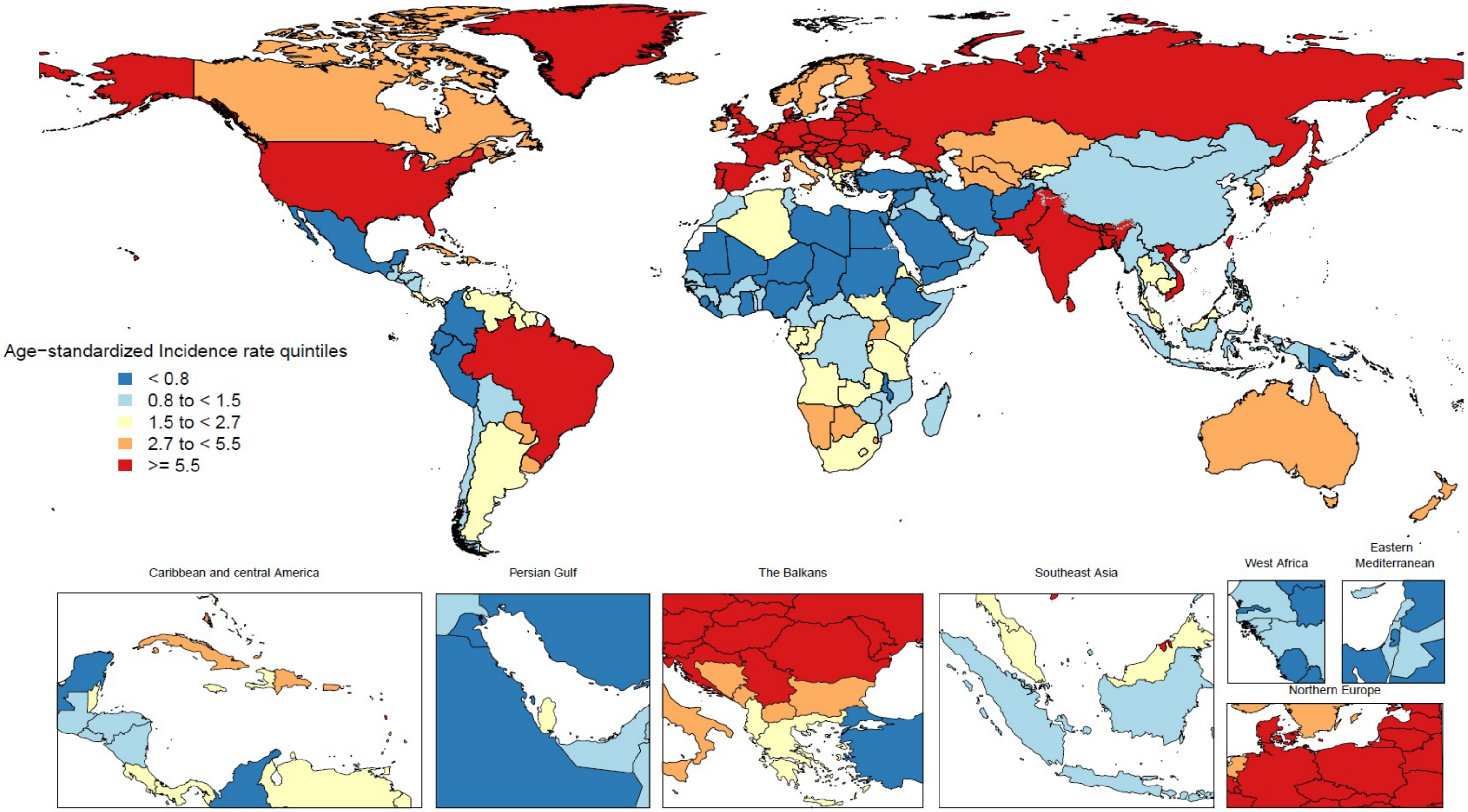

eFigure 8. Global map of A) age-standardized mortality rate quintiles, and B) age-standardized incidence rate quintiles for other pharynx cancer, males, 2019

Each map represents estimates at the national level and for the age range 20 to 95+ years. Quintiles are based on age-standardized mortality and incidence rates per 100,000 person-years. There are several geographic locations where estimates are not available (e.g., Western Sahara, French Guiana) as they were not modelled locations in the Global Burden of Diseases, Injuries, and Risk Factors 2019 study; these locations are white in this map.

A

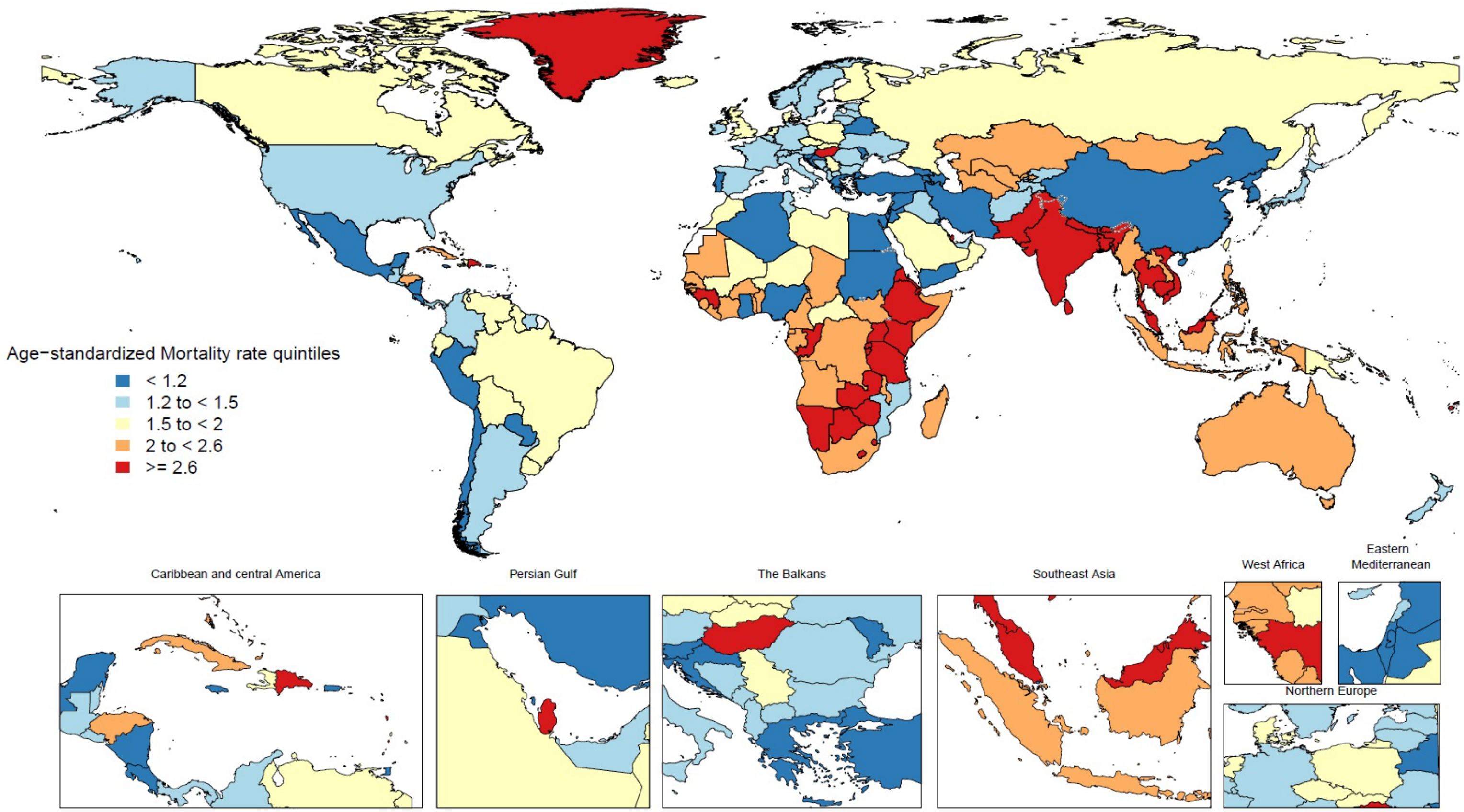
Age−standardized Mortality rate quintiles
< 1.2
1.2 to < 1.5
1.5 to < 2
2 to < 2.6
>= 2.6
Caribbean and central America
Persian Gulf
The Balkans
Southeast Asia
West Africa
Eastern Mediterranean
Northern Europe

B

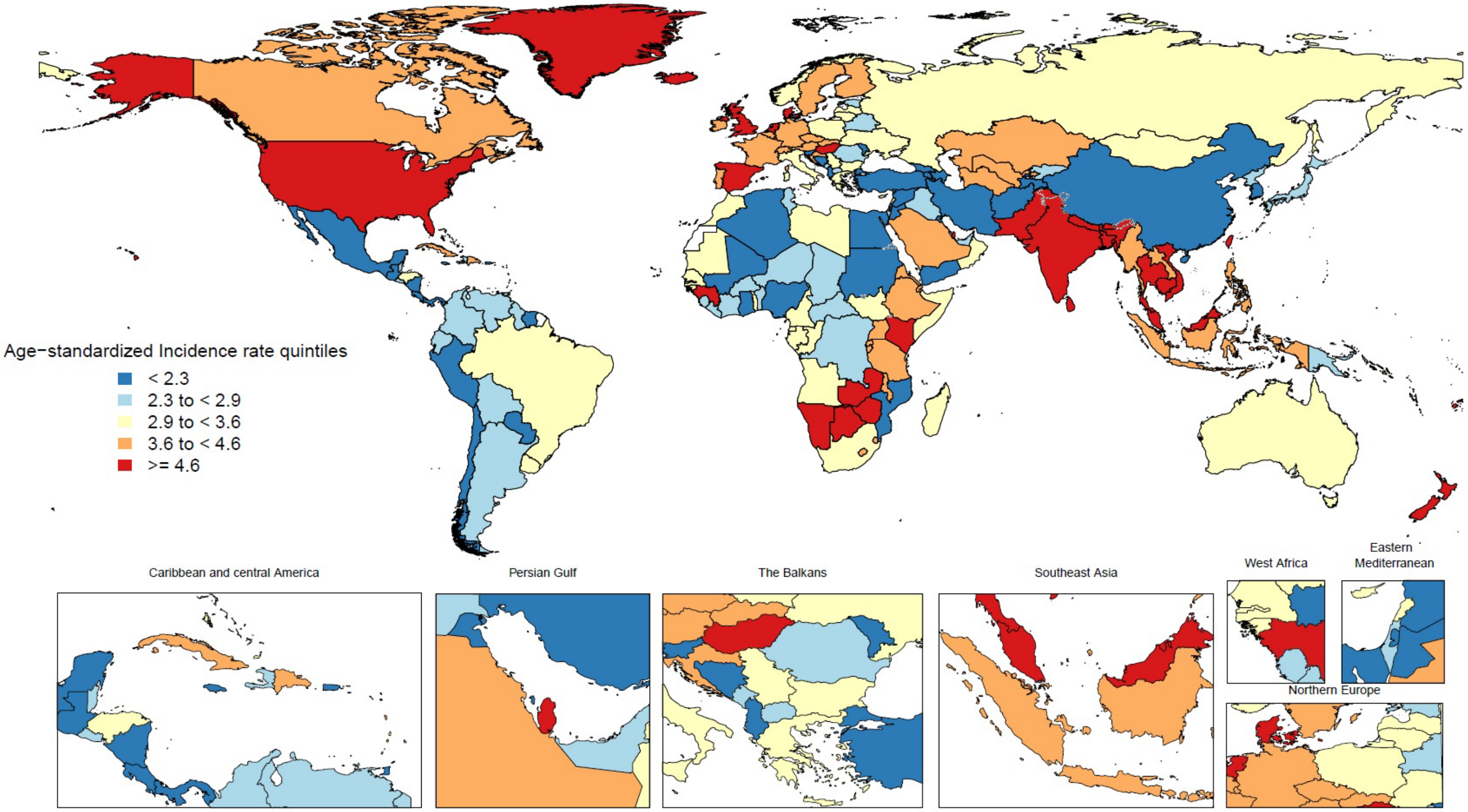

eFigure 9. Global map of A) age-standardized mortality rate quintiles, and B) age-standardized incidence rate quintiles for lip and oral cavity cancer, females, 2019

Each map represents estimates at the national level and for the age range 20 to 95+ years. Quintiles are based on age-standardized mortality and incidence rates per 100,000 person-years. There are several geographic locations where estimates are not available (e.g., Western Sahara, French Guiana) as they were not modelled locations in the Global Burden of Diseases, Injuries, and Risk Factors 2019 study; these locations are white in this map.

A

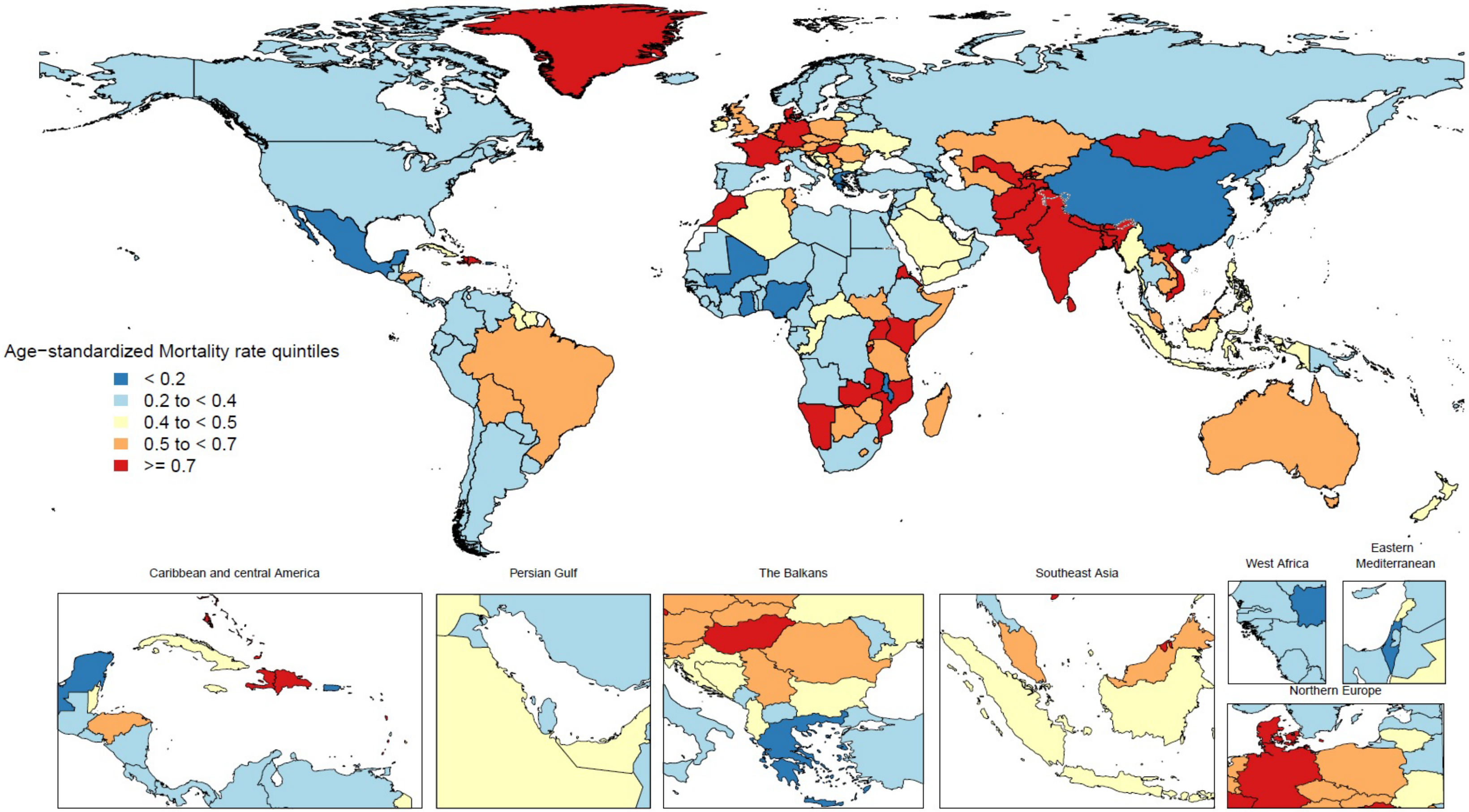
Age−standardized Mortality rate quintiles
< 0.2
0.2 to < 0.4
0.4 to < 0.5
0.5 to < 0.7
>= 0.7
Caribbean and central America
Persian Gulf
The Balkans
Southeast Asia
West Africa
Eastern Mediterranean
Northern Europe

B

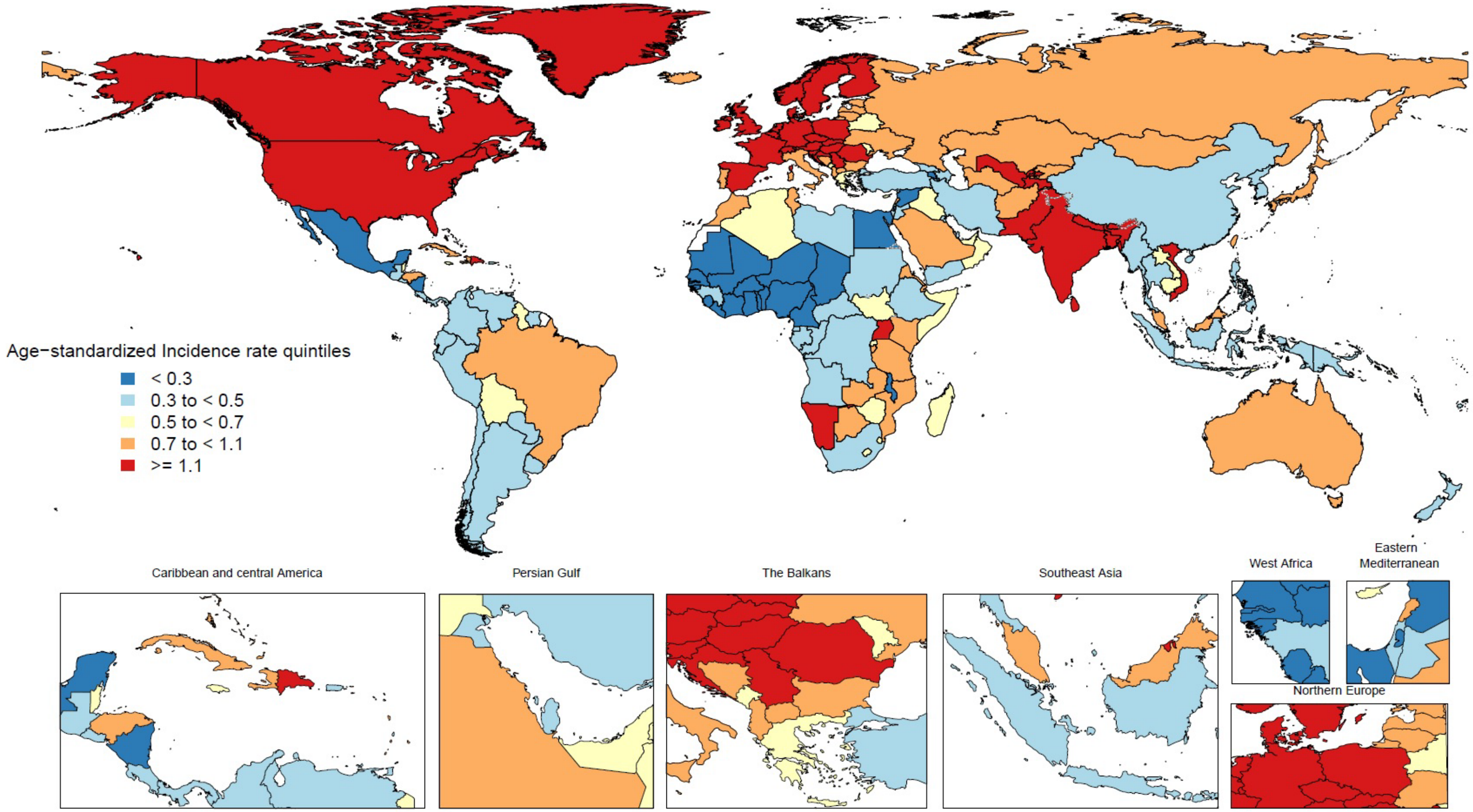

eFigure 10. Global map of A) age-standardized mortality rate quintiles, and B) age-standardized incidence rate quintiles for other pharynx cancer, females, 2019

Each map represents estimates at the national level and for the age range 20 to 95+ years. Quintiles are based on age-standardized mortality and incidence rates per 100,000 person-years. There are several geographic locations where estimates are not available (e.g., Western Sahara, French Guiana) as they were not modelled locations in the Global Burden of Diseases, Injuries, and Risk Factors 2019 study; these locations are white in this map.

Lip and oral cavity cancer

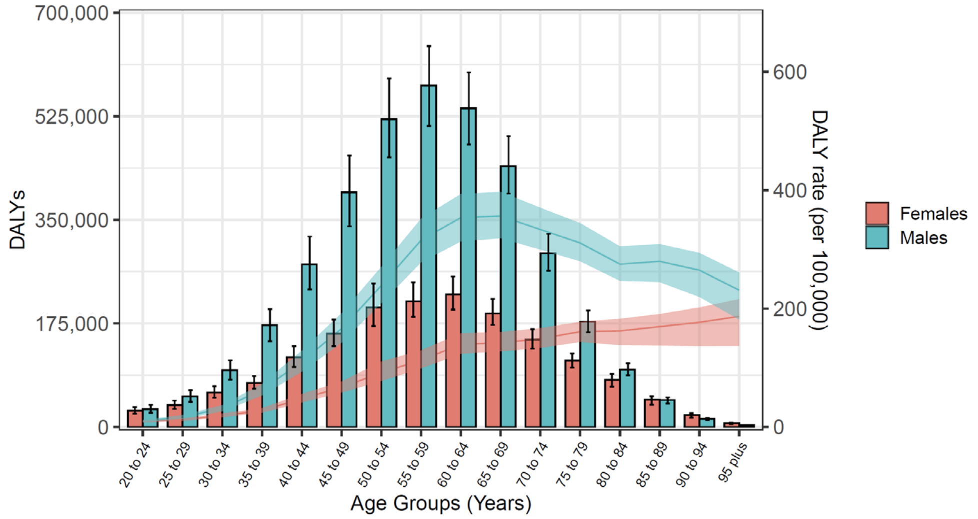

Other pharynx cancer

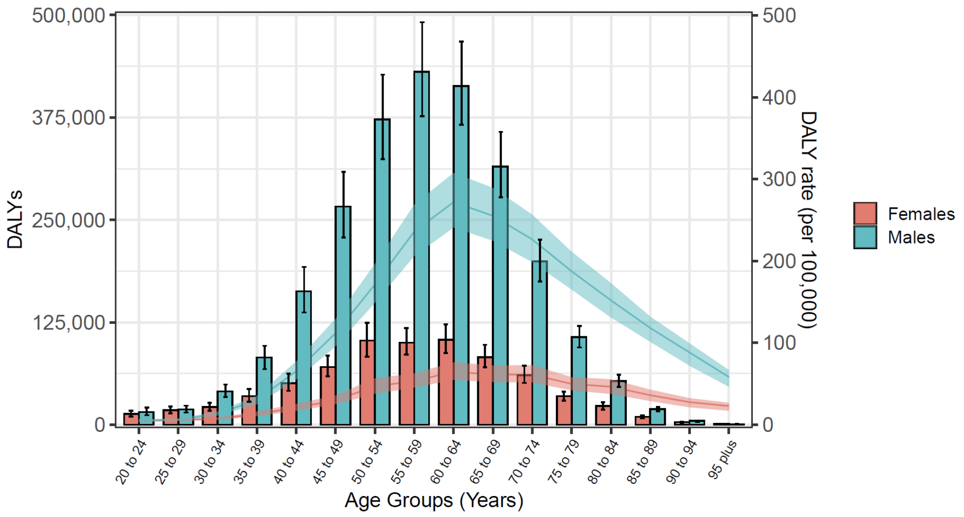

eFigure 11. Global absolute DALYs and age-specific DALY rates (per 100,000) for Lip and oral cavity cancer, and Other pharynx cancer by 5-year age group and sex in 2019
DALYs=disability-adjusted life years; bars indicate absolute numbers, and lines indicate rates, with shaded areas representing respective 95% uncertainty intervals (UIs).

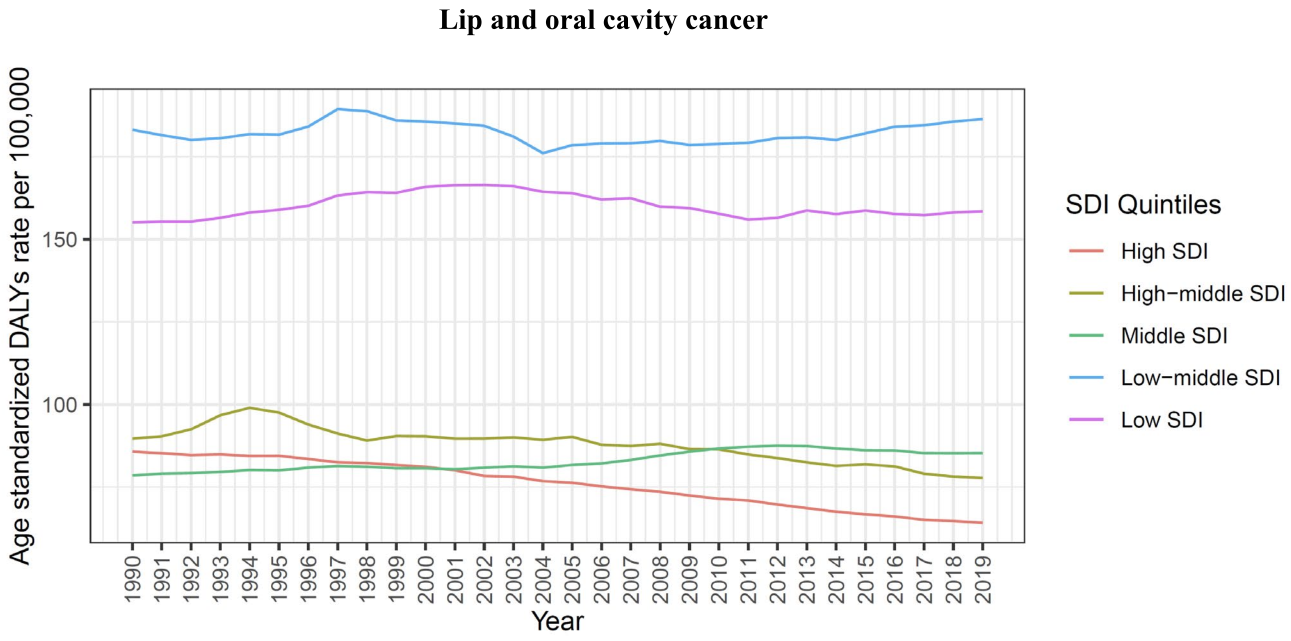

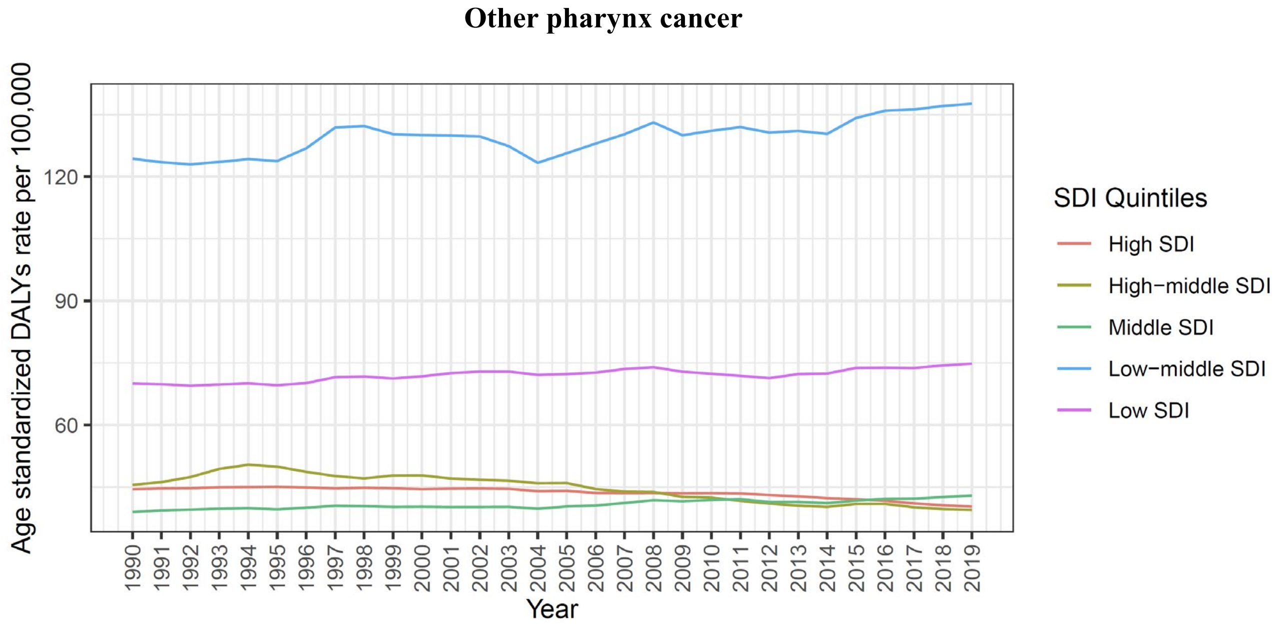

eFigure 12. Time trends of age-standardized DALY rates for Lip and oral cavity cancer and Other pharynx cancer from 1990 to 2019, by SDI quintile

Results in this figure represent both sexes combined. Rates are expressed per 100,000 person-years. See eFigure 3 (p53) and eTable 7 (p54) for details and definitions of the SDI quintiles. SDI=Socio-demographic Index; DALYs=disability-adjusted life years.

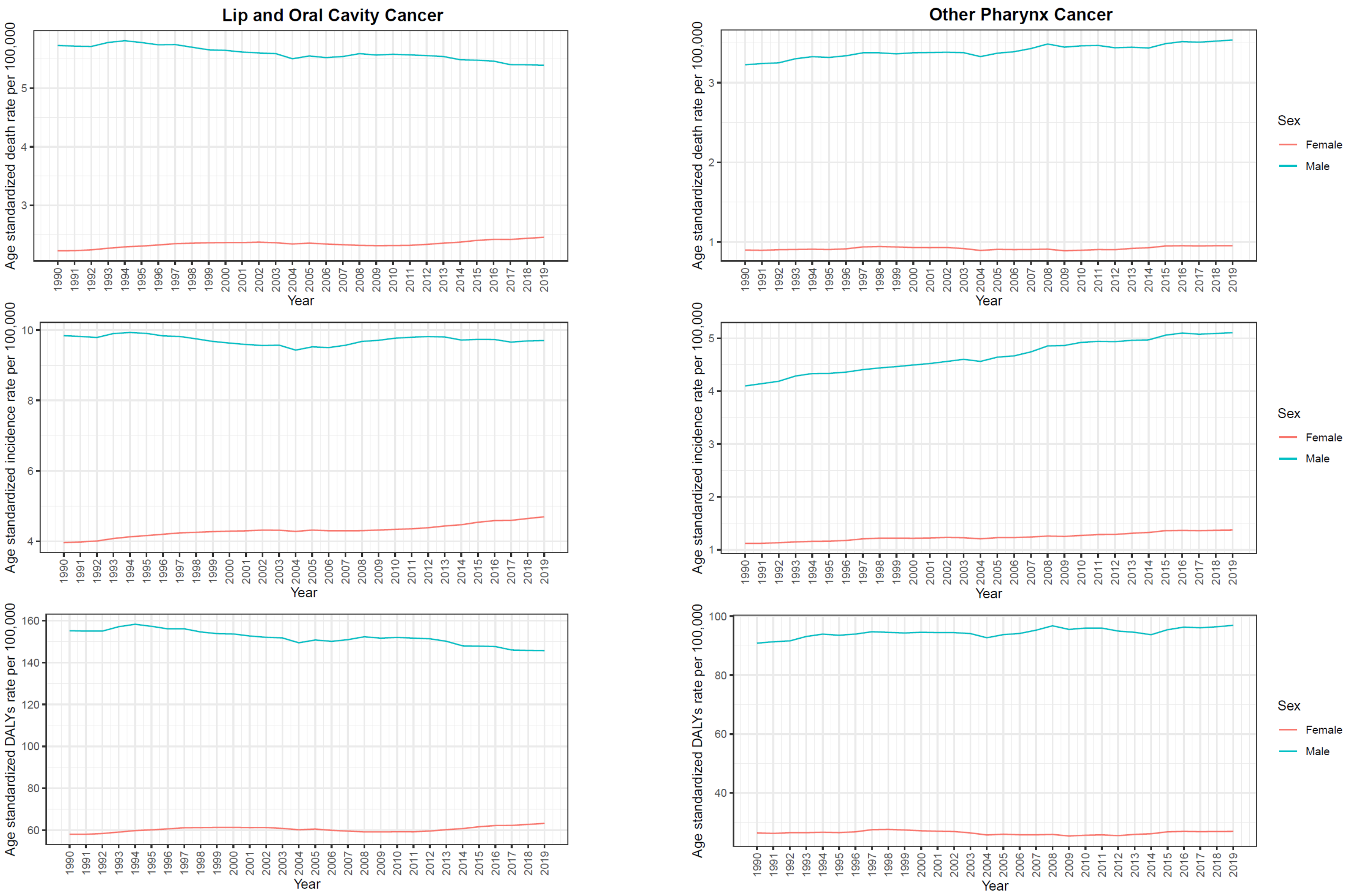

eFigure 13. Time trends of age-standardized deaths, incidence, and DALY rates for Lip and oral cavity cancer and Other pharynx cancer from 1990 to 2019, by sex

Rates are expressed per 100,000 person-years. DALYs=disability-adjusted life-years.

© 2023 Cunha AR et al. *JAMA Oncol*.

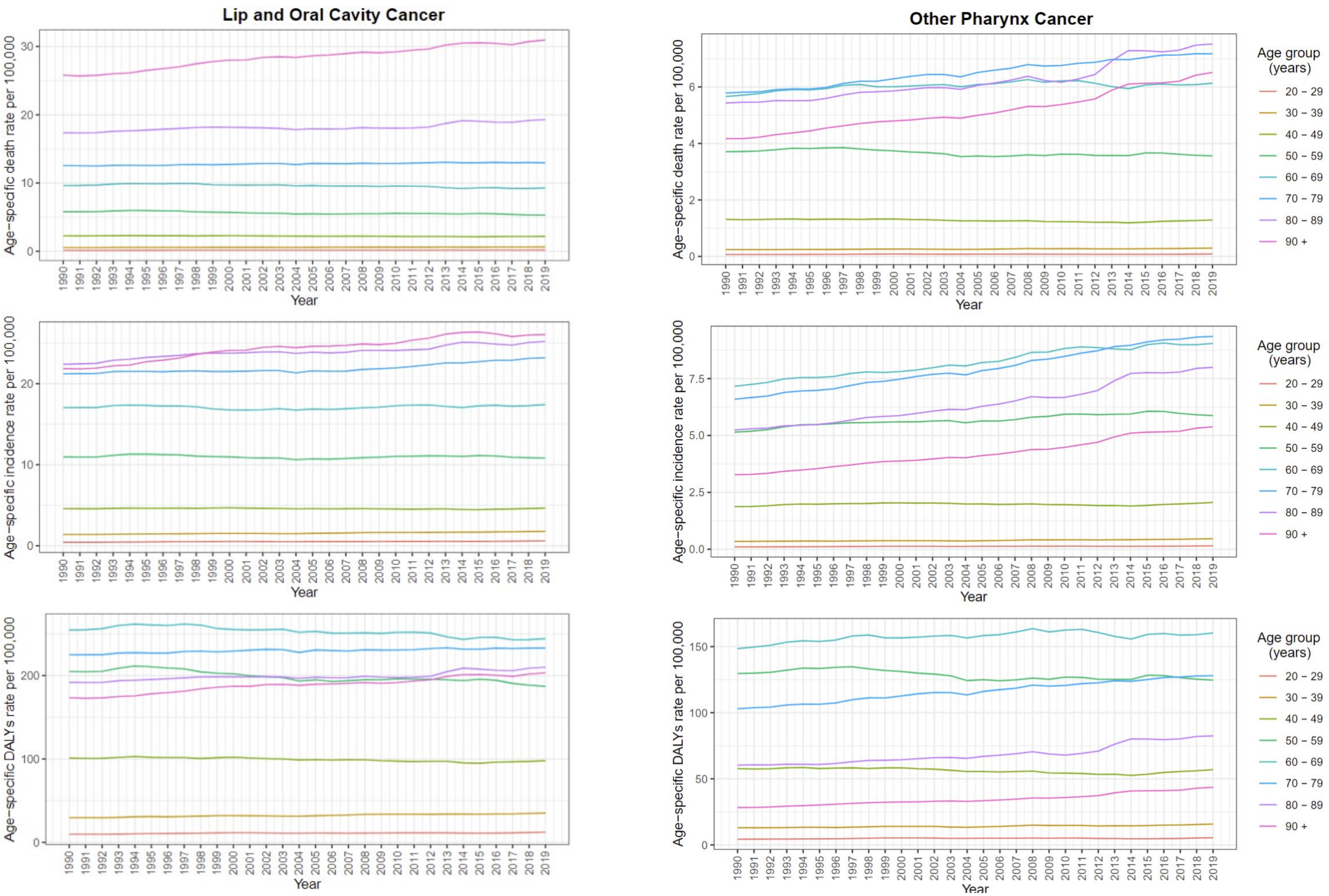

eFigure 14. Time trends of age-specific deaths, incidence, and DALY rates for Lip and oral cavity cancer and Other pharynx cancer from 1990 to 2019, by ten-year age group globally

Results in this figure represent both sexes combined. Rates are expressed per 100,000 person-years. See eFigure 3 (p53) and eTable 7 (p54) for details and definitions of the SDI regions. SDI=Socio-demographic Index; DALYs=disability-adjusted life-years.

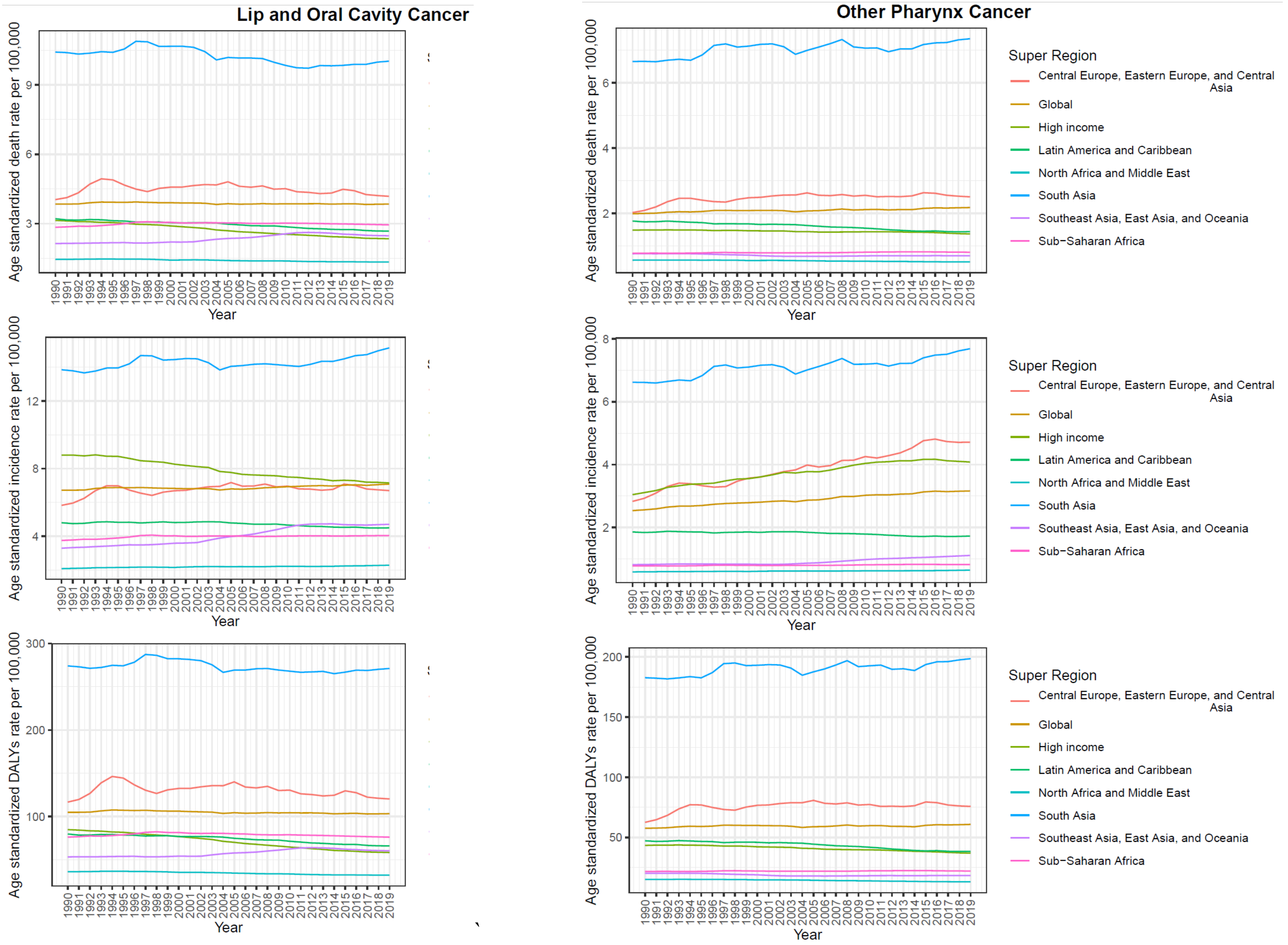

eFigure 15. Time trends of age-standardized deaths, incidence, and DALY rates for Lip and oral cavity cancer and Other pharynx cancer from 1990 to 2019, by GBD super-region

Rates are expressed per 100,000 person-years. SDI=Socio-demographic Index; DALYs=disability-adjusted life-years.

© 2023 Cunha AR et al. JAMA Oncol.

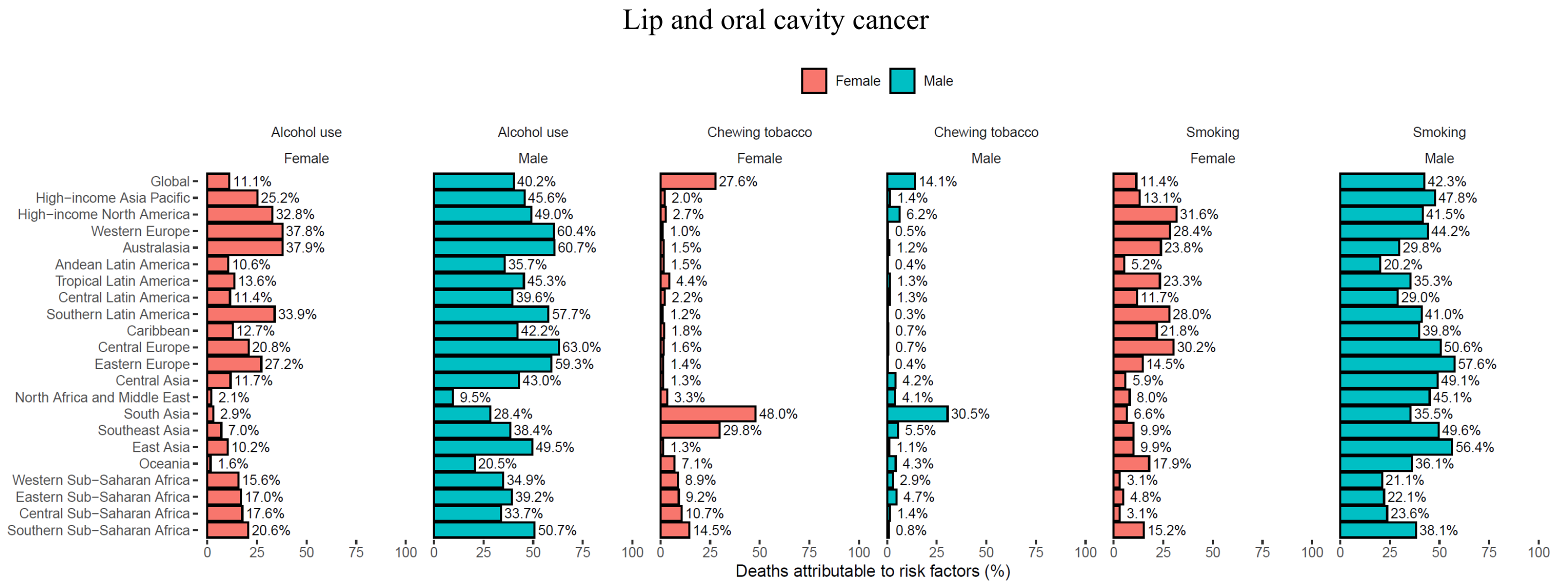

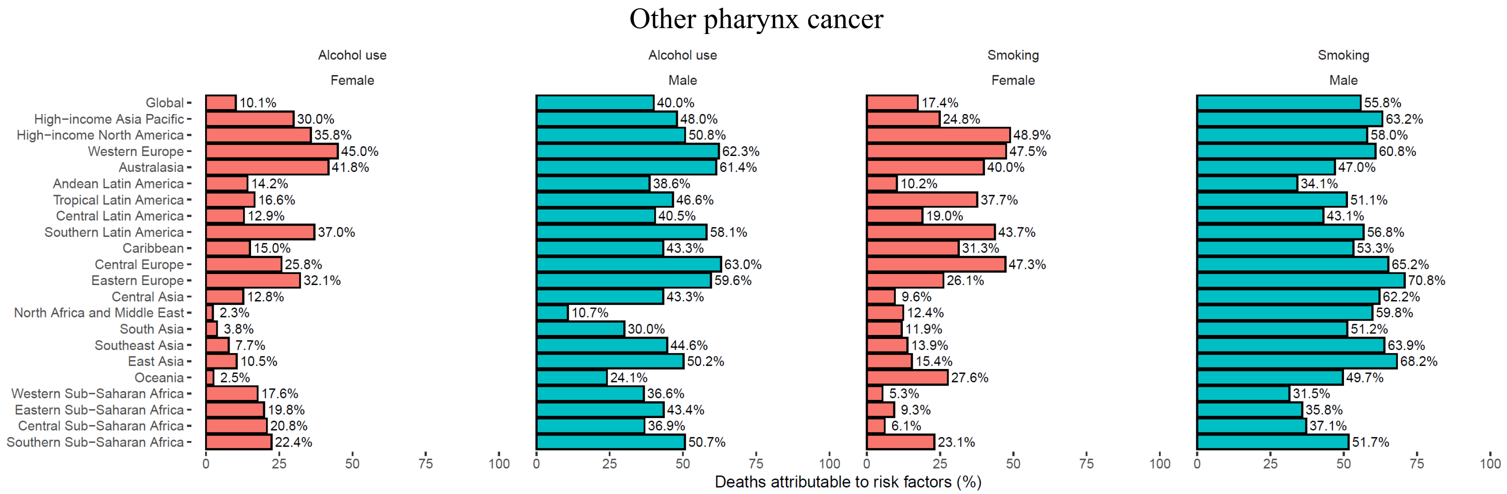

eFigure 16. Proportion of deaths attributable to risk factors for Lip and oral cavity cancer and Other pharynx cancer for males and females in 2019 by GBD world region

GBD= Global Burden of Diseases, Injuries, and Risk Factors 2019 study.

A

Lip and oral cavity cancer

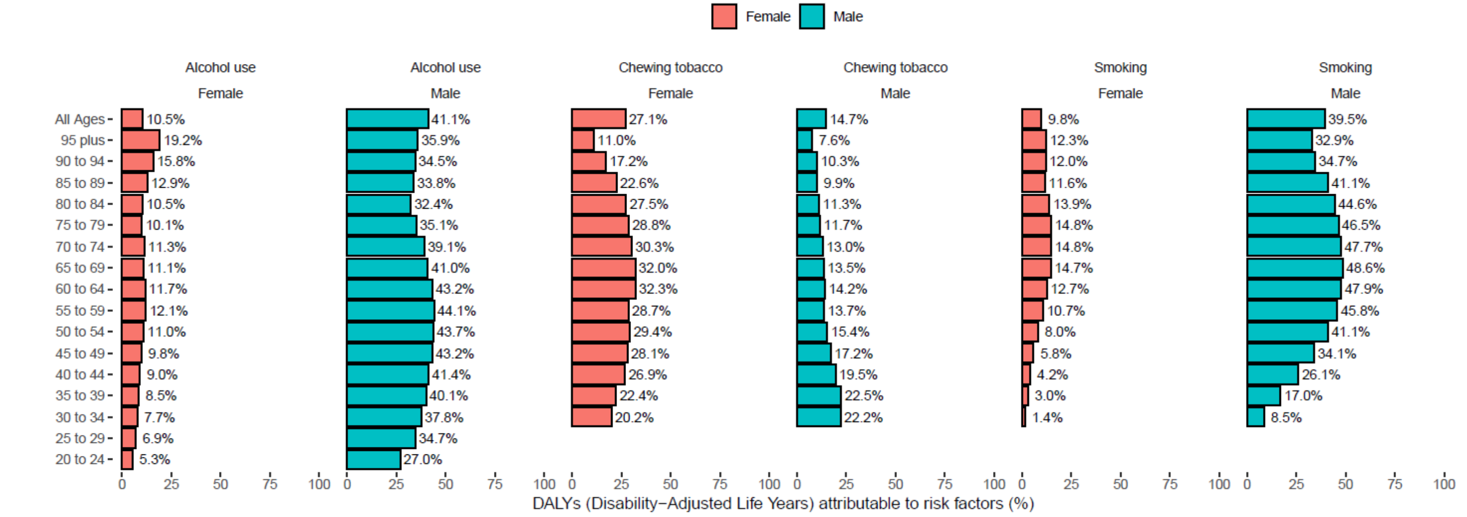

Other pharynx cancer

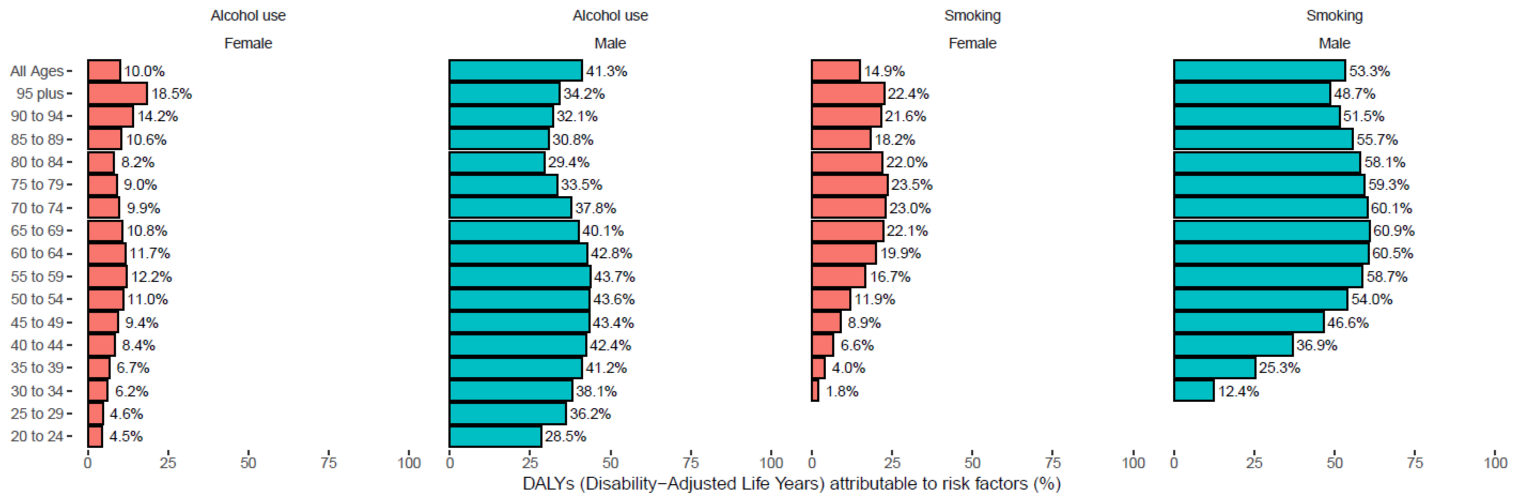

© 2023 Cunha AR et al. JAMA Oncol.

B

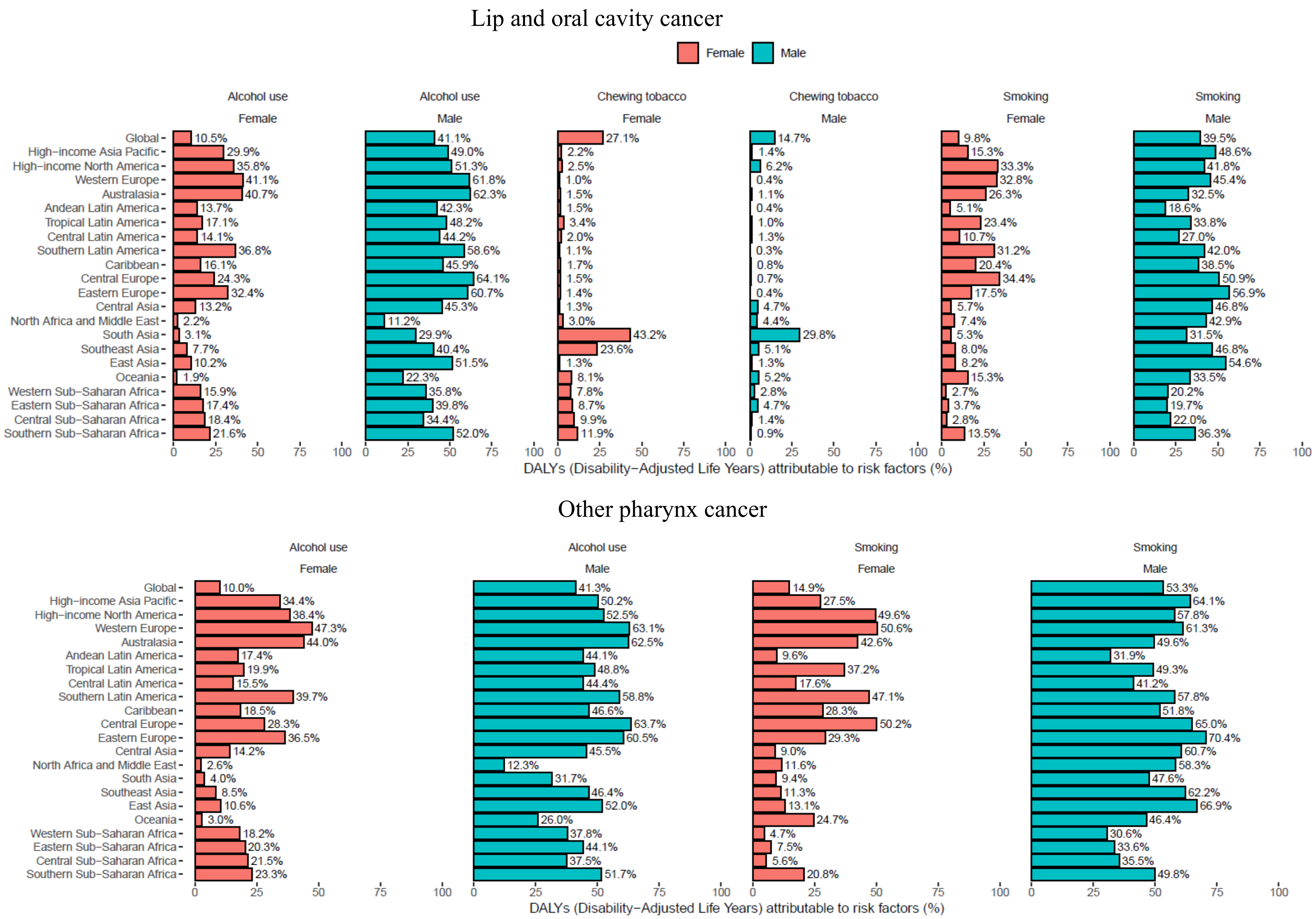

eFigure 17. Proportion of DALYs attributable to risk factors for Lip and oral cavity cancer and Other pharynx cancer A) by five-year age group globally, and B) by GBD world region, for males and females in 2019

The chewing tobacco and smoking risk factors were modeled with lower age restrictions of 30 years in the GBD 2019 study; thus, estimates were not produced for these risk factors in the age groups of 20 to 24 years and 25 to 29 years. GBD = Global Burden of Disease Study; DALYs = disability-adjusted life-years.

eTable 12. **Proportion of Lip and oral cavity cancer (LOC) deaths and DALYs attributable to risk factors, in 2019, by country or territory, both sexes combined**

Super-region, **region,** or country or territory	**Proportion of LOC deaths attributable to each risk factor (95% UI)**			**Proportion of LOC DALYs attributable to each risk factor (95% UI)**		
	**Alcohol use**	**Chewing tobacco**	**Smoking**	**Alcohol use**	**Chewing tobacco**	**Smoking**
Central Europe, Eastern Europe, and Central Asia						
**Central Asia**						
Armenia	26.6 (19.3 to 34.3)	0.6 (0.4 to 0.9)	46.4 (40.9 to 51.6)	30.9 (23.1 to 39.3)	0.5 (0.3 to 0.8)	47.4 (41.7 to 52.5)
Azerbaijan	38.6 (27.1 to 48.8)	0.7 (0.4 to 1.0)	37.1 (31.0 to 43.7)	40.7 (29.1 to 51.2)	0.7 (0.4 to 1.0)	35.9 (29.3 to 42.4)
Georgia	41.6 (34.1 to 49.2)	0.7 (0.4 to 1.0)	46.4 (40.4 to 52.0)	46.1 (38.2 to 53.8)	0.7 (0.4 to 1.1)	48.8 (42.2 to 54.8)
Kazakhstan	36.7 (28.0 to 44.8)	1.5 (0.9 to 2.4)	39.0 (32.6 to 44.6)	40.0 (30.8 to 48.3)	1.5 (0.9 to 2.3)	39.4 (32.3 to 45.8)
Kyrgyzstan	33.8 (26.8 to 40.7)	6.8 (3.8 to 10.6)	39.8 (34.1 to 45.1)	37.5 (30.2 to 44.7)	7.3 (4.1 to 11.4)	39.7 (33.6 to 45.5)
Mongolia	39.8 (31.7 to 47.7)	1.5 (0.9 to 2.4)	37.1 (28.4 to 44.5)	43.0 (34.6 to 50.8)	1.6 (0.9 to 2.5)	35.8 (26.6 to 43.7)
Tajikistan	18.9 (13.0 to 25.2)	6.7 (4.2 to 9.9)	24.1 (19.9 to 28.6)	21.1 (14.9 to 27.6)	6.9 (4.4 to 10.4)	22.3 (18.2 to 26.5)
Turkmenistan	34.8 (27.5 to 42.1)	0.8 (0.5 to 1.1)	32.8 (27.7 to 37.8)	36.8 (29.3 to 44.5)	0.8 (0.5 to 1.1)	32.1 (26.9 to 37.2)
Uzbekistan	25.9 (19.2 to 32.3)	6.1 (3.3 to 9.5)	26.5 (21.0 to 31.7)	28.0 (21.1 to 34.6)	6.5 (3.6 to 10.2)	24.6 (18.9 to 29.8)
**Central Europe**						
Albania	32.2 (24.1 to 39.8)	1.1 (0.7 to 1.7)	44.6 (38.3 to 50.5)	36.0 (27.7 to 44.1)	1.3 (0.8 to 1.9)	42.9 (37.2 to 48.3)
Bosnia and Herzegovina	41.5 (34.1 to 48.2)	0.9 (0.5 to 1.3)	51.6 (44.9 to 57.7)	46.4 (38.9 to 53.4)	0.9 (0.5 to 1.3)	53.7 (46.7 to 60.0)
Bulgaria	56.7 (48.9 to 63.6)	1.7 (1.0 to 2.6)	49.5 (43.2 to 55.5)	59.8 (52.2 to 66.8)	1.7 (1.0 to 2.7)	52.8 (46.1 to 58.7)
Croatia	53.2 (44.2 to 61.1)	0.6 (0.4 to 0.9)	47.4 (40.9 to 54.1)	55.6 (46.9 to 63.5)	0.6 (0.3 to 0.9)	49.4 (42.1 to 55.9)

Super-region**, region,** or country or territory	**Proportion of LOC deaths attributable to each risk factor (95% UI)**			**Proportion of LOC DALYs attributable to each risk factor (95% UI)**		
	**Alcohol use**	**Chewing tobacco**	**Smoking**	**Alcohol use**	**Chewing tobacco**	**Smoking**
Czechia	58.9 (51.1 to 65.5)	1.2 (0.7 to 1.8)	43.5 (36.5 to 50.0)	61.5 (53.8 to 67.9)	1.1 (0.7 to 1.7)	44.8 (37.1 to 51.6)
Hungary	55.1 (47.3 to 61.8)	0.6 (0.4 to 0.9)	47.7 (40.9 to 54.5)	57.1 (49.4 to 64.0)	0.6 (0.3 to 0.9)	49.6 (42.2 to 56.5)
Montenegro	52.7 (44.4 to 60.6)	0.9 (0.5 to 1.3)	54.9 (48.0 to 61.4)	54.4 (45.8 to 62.3)	0.9 (0.5 to 1.3)	55.5 (48.1 to 61.8)
North Macedonia	44.9 (37.4 to 52.0)	0.9 (0.6 to 1.3)	49.8 (43.1 to 55.9)	48.0 (40.5 to 55.1)	0.9 (0.5 to 1.3)	51.5 (44.6 to 57.7)
Poland	50.2 (42.8 to 56.7)	0.8 (0.5 to 1.2)	44.3 (37.6 to 50.6)	54.9 (47.5 to 61.5)	0.8 (0.5 to 1.2)	46.4 (39.0 to 53.1)
Romania	58.7 (50.8 to 66.2)	0.9 (0.5 to 1.5)	46.8 (39.5 to 53.3)	60.9 (53.1 to 68.2)	1.0 (0.5 to 1.5)	48.7 (40.4 to 55.7)
Serbia	48.1 (40.2 to 55.4)	0.9 (0.5 to 1.3)	49.0 (42.3 to 55.5)	51.6 (43.8 to 59.1)	0.9 (0.5 to 1.3)	51.1 (43.9 to 57.8)
Slovakia	58.0 (49.9 to 64.8)	0.5 (0.3 to 0.8)	42.3 (34.8 to 49.2)	60.3 (52.2 to 67.0)	0.5 (0.3 to 0.8)	43.4 (34.9 to 50.7)
Slovenia	41.9 (23.2 to 57.5)	0.9 (0.5 to 1.3)	40.1 (33.8 to 46.0)	44.3 (25.0 to 60.2)	0.8 (0.5 to 1.3)	44.0 (37.5 to 50.2)
**Eastern Europe**						
Belarus	59.6 (52.2 to 66.4)	0.5 (0.3 to 0.7)	50.5 (43.3 to 56.7)	62.0 (54.5 to 68.8)	0.4 (0.3 to 0.7)	51.9 (43.9 to 58.7)
Estonia	55.0 (47.3 to 62.4)	0.8 (0.5 to 1.1)	44.1 (37.6 to 50.7)	59.2 (51.4 to 66.5)	0.8 (0.5 to 1.2)	47.7 (40.7 to 54.3)
Latvia	53.5 (45.1 to 60.7)	0.6 (0.3 to 0.9)	45.3 (38.1 to 51.9)	57.7 (49.4 to 64.6)	0.5 (0.3 to 0.7)	48.5 (40.3 to 55.6)
Lithuania	57.0 (48.8 to 63.9)	0.5 (0.3 to 0.8)	41.4 (34.0 to 48.0)	60.4 (52.4 to 67.3)	0.5 (0.3 to 0.7)	43.9 (35.4 to 51.2)
Republic of Moldova	57.2 (48.7 to 64.9)	0.5 (0.3 to 0.8)	49.1 (42.5 to 55.1)	59.8 (51.2 to 67.1)	0.5 (0.3 to 0.8)	51.0 (44.4 to 57.2)
Russian Federation	51.8 (43.8 to 59.6)	0.6 (0.4 to 1.0)	47.4 (40.8 to 53.7)	55.4 (47.2 to 63.1)	0.6 (0.4 to 0.9)	49.4 (42.0 to 56.0)
Ukraine	51.4 (41.9 to 59.6)	0.6 (0.4 to 0.9)	50.1 (43.0 to 56.6)	54.3 (44.9 to 62.4)	0.6 (0.4 to 1.0)	50.8 (43.1 to 57.6)

Super-region, **region,** or country or territory	**Proportion of LOC deaths attributable to each risk factor (95% UI)**			**Proportion of LOC DALYs attributable to each risk factor (95% UI)**		
	**Alcohol use**	**Chewing tobacco**	**Smoking**	**Alcohol use**	**Chewing tobacco**	**Smoking**
High-income						
**Australasia**						
Australia	51.9 (43.7 to 59.8)	1.3 (0.8 to 2.0)	27.3 (21.8 to 32.8)	55.1 (47.0 to 62.8)	1.2 (0.8 to 1.9)	30.5 (24.3 to 36.5)
New Zealand	50.6 (42.4 to 58.7)	1.4 (0.8 to 2.1)	28.7 (22.6 to 35.1)	53.7 (45.4 to 61.4)	1.4 (0.8 to 2.0)	30.3 (23.6 to 36.8)
**High-income Asia Pacific**						
Brunei Darussalam	2.6 (0.0 to 6.6)	3.1 (2.0 to 4.5)	30.4 (24.6 to 35.9)	3.0 (0.0 to 7.6)	3.1 (2.0 to 4.4)	28.8 (22.9 to 34.3)
Japan	34.5 (26.1 to 43.2)	1.7 (1.0 to 2.7)	31.4 (26.0 to 37.2)	39.9 (31.2 to 48.2)	1.7 (1.1 to 2.5)	36.2 (30.7 to 41.8)
Republic of Korea	50.6 (41.5 to 58.9)	1.7 (1.0 to 2.5)	37.9 (32.6 to 43.3)	54.5 (45.6 to 62.6)	1.7 (1.0 to 2.5)	39.2 (33.2 to 44.6)
Singapore	15.9 (9.5 to 22.4)	1.7 (1.1 to 2.6)	26.7 (21.3 to 32.1)	18.9 (11.6 to 26.1)	1.8 (1.1 to 2.7)	27.6 (21.7 to 33.3)
**High-income North America**						
Canada	45.5 (37.1 to 53.4)	2.3 (1.3 to 3.5)	35.2 (29.1 to 41.7)	49.1 (40.7 to 56.6)	2.2 (1.3 to 3.4)	36.6 (30.4 to 43.0)
Greenland	40.0 (26.9 to 51.2)	4.5 (2.5 to 7.1)	50.9 (43.0 to 58.2)	42.2 (29.0 to 53.8)	4.6 (2.5 to 7.2)	51.2 (42.6 to 58.6)
United States of America	43.4 (35.8 to 50.5)	5.4 (3.2 to 8.4)	38.7 (32.2 to 46.0)	46.5 (38.7 to 53.6)	5.5 (3.2 to 8.5)	39.8 (33.5 to 46.0)
**Southern Latin America**						
Argentina	50.4 (41.8 to 58.0)	0.6 (0.3 to 0.9)	38.2 (31.3 to 44.8)	53.2 (44.7 to 60.7)	0.5 (0.3 to 0.8)	39.7 (32.3 to 46.7)
Chile	48.0 (39.6 to 56.4)	0.7 (0.4 to 1.1)	30.4 (23.5 to 36.9)	51.2 (42.7 to 59.0)	0.7 (0.4 to 1.0)	35.3 (27.1 to 42.8)
Uruguay	47.5 (39.0 to 55.8)	0.6 (0.4 to 1.0)	37.3 (30.7 to 43.6)	50.1 (41.4 to 58.4)	0.5 (0.4 to 0.8)	41.2 (33.9 to 47.8)
**Western Europe**						

***Super-region*, region,** or country or territory	**Proportion of LOC deaths attributable to each risk factor (95% UI)**			**Proportion of LOC DALYs attributable to each risk factor (95% UI)**		
	**Alcohol use**	**Chewing tobacco**	**Smoking**	**Alcohol use**	**Chewing tobacco**	**Smoking**
Andorra	56.6 (48.3 to 63.6)	0.6 (0.4 to 1.0)	41.4 (34.3 to 48.1)	58.9 (50.7 to 65.8)	0.6 (0.4 to 0.9)	43.3 (35.0 to 50.8)
Austria	55.2 (46.7 to 62.7)	0.8 (0.5 to 1.2)	41.8 (35.4 to 48.2)	57.9 (49.7 to 65.2)	0.8 (0.5 to 1.2)	44.8 (37.4 to 51.5)
Belgium	57.7 (49.6 to 65.0)	0.5 (0.3 to 0.8)	41.3 (35.0 to 47.6)	59.8 (51.7 to 67.0)	0.5 (0.3 to 0.7)	43.1 (36.7 to 49.4)
Cyprus	45.6 (37.3 to 53.9)	0.6 (0.4 to 1.0)	42.3 (35.9 to 48.4)	49.2 (41.2 to 57.4)	0.6 (0.4 to 0.9)	45.2 (38.6 to 51.3)
Denmark	54.0 (44.9 to 62.4)	1.0 (0.6 to 1.5)	43.5 (36.8 to 50.5)	56.0 (47.2 to 64.0)	0.9 (0.6 to 1.4)	43.2 (36.3 to 49.8)
Finland	41.1 (32.3 to 49.5)	0.7 (0.4 to 1.2)	30.0 (24.1 to 36.4)	46.8 (38.1 to 55.0)	0.7 (0.4 to 1.1)	33.8 (27.5 to 40.1)
France	58.8 (49.8 to 66.3)	0.5 (0.3 to 0.8)	37.8 (31.3 to 44.3)	61.0 (52.1 to 68.3)	0.5 (0.3 to 0.7)	41.0 (33.0 to 47.7)
Germany	61.7 (53.5 to 68.9)	0.7 (0.4 to 1.1)	40.5 (34.1 to 46.8)	63.6 (55.7 to 70.6)	0.7 (0.4 to 1.0)	43.3 (36.4 to 49.6)
Greece	41.5 (33.9 to 48.3)	0.7 (0.4 to 1.1)	45.9 (39.7 to 52.1)	47.0 (39.3 to 53.7)	0.6 (0.4 to 0.9)	50.0 (43.6 to 55.8)
Iceland	44.6 (35.8 to 52.6)	2.2 (1.4 to 3.4)	34.9 (28.8 to 40.9)	48.5 (39.6 to 56.3)	2.3 (1.4 to 3.4)	35.7 (29.7 to 41.7)
Ireland	50.3 (41.8 to 58.0)	0.5 (0.3 to 0.8)	38.6 (32.4 to 45.0)	53.2 (44.8 to 60.4)	0.5 (0.3 to 0.8)	38.9 (32.4 to 45.1)
Israel	19.1 (12.2 to 26.2)	0.7 (0.4 to 1.1)	33.0 (27.3 to 38.9)	23.5 (15.7 to 31.3)	0.7 (0.4 to 1.0)	35.2 (29.0 to 41.2)
Italy	47.8 (39.5 to 56.0)	0.6 (0.4 to 1.0)	35.7 (29.6 to 42.1)	51.2 (42.7 to 58.9)	0.6 (0.4 to 0.9)	38.8 (32.5 to 45.2)
Luxembourg	57.1 (48.3 to 64.3)	0.5 (0.3 to 0.8)	38.7 (31.6 to 45.7)	59.7 (50.9 to 66.8)	0.5 (0.3 to 0.7)	39.8 (32.2 to 47.0)
Malta	43.7 (35.3 to 51.4)	0.6 (0.4 to 1.0)	35.6 (29.5 to 41.8)	47.0 (38.7 to 54.7)	0.6 (0.4 to 0.8)	38.7 (32.3 to 44.9)
Monaco	33.2 (0.3 to 58.2)	0.8 (0.5 to 1.2)	36.2 (29.0 to 43.0)	36.3 (0.4 to 61.5)	0.7 (0.5 to 1.1)	39.5 (32.1 to 46.5)

Super-region, **region,** or country or territory	**Proportion of LOC deaths attributable to each risk factor (95% UI)**			**Proportion of LOC DALYs attributable to each risk factor (95% UI)**		
	**Alcohol use**	**Chewing tobacco**	**Smoking**	**Alcohol use**	**Chewing tobacco**	**Smoking**
Netherlands	52.1 (43.3 to 59.6)	0.6 (0.4 to 0.9)	41.0 (34.2 to 47.9)	54.6 (45.9 to 61.9)	0.5 (0.3 to 0.8)	41.8 (34.7 to 48.7)
Norway	40.8 (31.7 to 49.4)	2.8 (1.7 to 4.1)	28.3 (22.8 to 34.1)	44.6 (35.1 to 52.8)	3.1 (2.0 to 4.6)	32.4 (25.9 to 38.7)
Portugal	55.1 (47.1 to 62.4)	0.5 (0.3 to 0.7)	35.0 (28.9 to 40.6)	59.4 (51.3 to 66.6)	0.4 (0.2 to 0.6)	40.0 (32.8 to 46.5)
San Marino	46.1 (2.9 to 61.0)	0.8 (0.5 to 1.2)	31.6 (25.4 to 38.0)	49.5 (3.3 to 64.2)	0.7 (0.5 to 1.1)	35.0 (28.3 to 41.5)
Spain	47.1 (38.6 to 55.0)	0.5 (0.3 to 0.8)	41.5 (35.5 to 47.5)	51.7 (43.0 to 59.4)	0.5 (0.3 to 0.7)	47.2 (40.9 to 53.4)
Sweden	51.0 (41.2 to 59.6)	1.8 (1.1 to 2.8)	32.3 (26.2 to 39.1)	53.2 (43.5 to 61.7)	2.3 (1.3 to 3.6)	33.0 (26.9 to 39.6)
Switzerland	56.4 (46.9 to 64.2)	0.7 (0.4 to 1.1)	39.5 (33.1 to 45.9)	58.0 (48.9 to 65.7)	0.7 (0.4 to 1.0)	40.6 (33.6 to 46.9)
United Kingdom	46.0 (36.9 to 54.5)	0.6 (0.4 to 0.9)	39.7 (33.3 to 46.5)	49.6 (40.3 to 58.2)	0.5 (0.4 to 0.8)	41.4 (34.8 to 48.0)
Latin America and Caribbean						
**Andean Latin America**						
Bolivia (Plurinational State of)	25.0 (16.9 to 33.2)	1.0 (0.6 to 1.5)	16.4 (11.0 to 22.4)	29.2 (20.6 to 37.8)	1.0 (0.6 to 1.5)	15.3 (9.9 to 21.0)
Ecuador	18.8 (13.9 to 24.1)	0.7 (0.4 to 1.2)	18.7 (13.4 to 23.9)	24.0 (18.4 to 30.0)	0.7 (0.4 to 1.0)	17.6 (12.1 to 23.0)
Peru	26.0 (17.4 to 34.4)	1.1 (0.7 to 1.7)	8.5 (5.1 to 12.4)	32.4 (22.7 to 41.5)	1.1 (0.7 to 1.7)	8.4 (4.8 to 12.3)
**Caribbean**						
Antigua and Barbuda	37.2 (29.3 to 44.6)	0.7 (0.4 to 1.1)	22.6 (17.5 to 28.2)	41.5 (33.4 to 48.9)	0.7 (0.4 to 1.1)	23.2 (17.6 to 29.1)
Bahamas	33.8 (17.0 to 46.2)	0.7 (0.4 to 1.1)	19.7 (15.0 to 25.1)	37.5 (19.2 to 50.1)	0.7 (0.4 to 1.0)	19.7 (14.7 to 25.3)
Barbados	40.9 (33.4 to 48.2)	0.6 (0.4 to 0.9)	19.6 (15.1 to 24.3)	45.3 (37.5 to 52.8)	0.6 (0.4 to 0.9)	20.1 (15.0 to 25.3)

Super-region, **region,** or country or territory	**Proportion of LOC deaths attributable to each risk factor (95% UI)**			**Proportion of LOC DALYs attributable to each risk factor (95% UI)**		
	**Alcohol use**	**Chewing tobacco**	**Smoking**	**Alcohol use**	**Chewing tobacco**	**Smoking**
Belize	33.8 (27.0 to 40.4)	0.8 (0.5 to 1.2)	25.0 (19.1 to 30.8)	38.0 (30.6 to 44.7)	0.8 (0.5 to 1.2)	23.9 (17.7 to 30.3)
Bermuda	42.8 (33.6 to 51.1)	0.7 (0.4 to 1.1)	26.9 (20.3 to 33.4)	48.0 (38.6 to 56.2)	0.7 (0.4 to 1.0)	28.9 (22.0 to 35.7)
Cuba	34.5 (27.5 to 41.4)	0.8 (0.5 to 1.2)	44.5 (37.6 to 51.0)	39.2 (31.8 to 46.4)	0.7 (0.5 to 1.1)	46.0 (38.8 to 52.8)
Dominica	36.7 (29.1 to 44.3)	0.6 (0.4 to 1.0)	19.1 (13.9 to 24.6)	41.9 (34.0 to 49.7)	0.6 (0.4 to 0.8)	20.3 (14.3 to 26.2)
Dominican Republic	30.2 (23.7 to 37.2)	1.4 (0.8 to 2.0)	33.5 (27.1 to 39.7)	35.9 (28.7 to 43.4)	1.5 (0.9 to 2.2)	29.7 (23.6 to 35.7)
Grenada	46.4 (38.4 to 53.5)	0.9 (0.5 to 1.4)	26.1 (18.8 to 32.5)	49.8 (41.6 to 57.1)	0.9 (0.5 to 1.5)	26.4 (18.7 to 33.4)
Guyana	38.8 (32.3 to 45.4)	0.9 (0.6 to 1.3)	23.6 (17.0 to 30.5)	42.0 (35.3 to 48.8)	0.9 (0.6 to 1.3)	24.0 (17.0 to 31.3)
Haiti	38.5 (30.7 to 46.7)	1.4 (0.9 to 2.1)	14.6 (9.0 to 20.5)	40.9 (32.9 to 49.0)	1.5 (0.9 to 2.2)	14.4 (8.8 to 20.4)
Jamaica	26.5 (20.0 to 33.0)	0.9 (0.6 to 1.4)	26.5 (21.1 to 31.8)	30.5 (23.3 to 37.4)	0.8 (0.5 to 1.2)	26.7 (20.9 to 32.3)
Puerto Rico	32.8 (26.6 to 39.6)	0.9 (0.5 to 1.4)	24.9 (18.8 to 30.9)	38.1 (31.4 to 44.9)	0.8 (0.5 to 1.2)	26.3 (19.5 to 32.5)
Saint Kitts and Nevis	23.0 (0.8 to 46.6)	0.5 (0.3 to 0.8)	20.8 (15.7 to 26.1)	25.4 (1.0 to 50.1)	0.5 (0.3 to 0.7)	21.9 (16.3 to 27.6)
Saint Lucia	44.4 (36.5 to 52.0)	0.8 (0.5 to 1.2)	26.9 (20.8 to 32.5)	49.0 (40.9 to 56.8)	0.8 (0.5 to 1.2)	27.9 (21.3 to 33.9)
Saint Vincent and the Grenadines	42.9 (35.4 to 50.0)	0.5 (0.3 to 0.8)	23.4 (17.2 to 29.7)	47.2 (39.4 to 54.0)	0.5 (0.3 to 0.7)	24.1 (17.3 to 31.0)
Suriname	35.0 (27.1 to 42.3)	0.8 (0.5 to 1.2)	35.2 (28.1 to 41.7)	39.2 (31.1 to 47.1)	0.8 (0.5 to 1.2)	36.1 (28.3 to 43.2)
Trinidad and Tobago	38.3 (30.8 to 45.6)	0.7 (0.4 to 1.0)	28.9 (22.1 to 35.4)	41.7 (34.0 to 49.1)	0.7 (0.4 to 1.0)	28.9 (21.6 to 35.8)
United States Virgin Islands	33.7 (1.6 to 56.4)	0.5 (0.3 to 0.8)	22.7 (16.9 to 28.2)	35.9 (1.8 to 58.9)	0.5 (0.3 to 0.8)	22.7 (16.9 to 28.2)
**Central Latin America**						

***Super-region*, region,** or country or territory	**Proportion of LOC deaths attributable to each risk factor (95% UI)**			**Proportion of LOC DALYs attributable to each risk factor (95% UI)**		
	**Alcohol use**	**Chewing tobacco**	**Smoking**	**Alcohol use**	**Chewing tobacco**	**Smoking**
Colombia	20.9 (15.8 to 26.6)	1.4 (0.8 to 2.2)	19.4 (13.8 to 25.0)	26.2 (20.2 to 32.5)	1.3 (0.8 to 2.0)	20.4 (14.1 to 26.4)
Costa Rica	27.7 (20.9 to 34.7)	0.9 (0.5 to 1.4)	24.6 (18.8 to 30.2)	32.8 (25.7 to 40.0)	0.7 (0.4 to 1.1)	24.0 (17.8 to 29.9)
El Salvador	19.4 (13.5 to 24.8)	1.3 (0.8 to 2.1)	14.2 (10.1 to 18.3)	24.0 (17.2 to 30.0)	1.3 (0.8 to 1.9)	15.0 (10.5 to 19.5)
Guatemala	12.8 (8.6 to 17.5)	1.2 (0.8 to 1.9)	16.0 (10.9 to 21.2)	16.8 (11.9 to 22.3)	1.1 (0.7 to 1.7)	14.0 (8.9 to 19.2)
Honduras	19.3 (13.6 to 25.6)	1.4 (0.9 to 2.1)	22.5 (16.1 to 28.3)	20.9 (15.1 to 27.1)	1.4 (0.9 to 2.1)	20.9 (14.2 to 27.0)
Mexico	32.4 (26.1 to 38.9)	1.2 (0.8 to 2.0)	22.5 (15.9 to 28.8)	36.6 (29.6 to 43.4)	1.2 (0.8 to 1.8)	20.2 (13.4 to 26.5)
Nicaragua	22.9 (17.4 to 28.6)	1.3 (0.8 to 1.9)	19.7 (14.6 to 25.2)	25.9 (19.9 to 31.7)	1.2 (0.8 to 1.8)	19.4 (13.7 to 25.4)
Panama	32.4 (25.5 to 39.1)	1.2 (0.7 to 1.8)	18.6 (14.0 to 23.2)	37.8 (30.5 to 44.7)	1.1 (0.7 to 1.6)	17.6 (13.1 to 22.2)
Venezuela (Bolivarian Republic of)	31.7 (24.3 to 38.7)	3.5 (2.1 to 5.2)	25.9 (18.7 to 32.8)	36.4 (28.7 to 44.0)	3.6 (2.1 to 5.5)	25.3 (17.9 to 32.9)
**Tropical Latin America**						
Brazil	36.8 (29.7 to 43.6)	2.1 (1.3 to 3.1)	32.1 (25.5 to 38.2)	41.2 (33.7 to 48.3)	1.5 (1.0 to 2.3)	31.6 (24.4 to 37.9)
Paraguay	48.2 (39.9 to 56.1)	5.2 (3.2 to 7.6)	39.2 (31.6 to 46.2)	51.3 (42.8 to 59.2)	4.6 (2.8 to 7.0)	36.3 (28.3 to 43.9)
North Africa and Middle East						
**North Africa and Middle East**						
Afghanistan	1.0 (0.4 to 1.9)	8.2 (4.8 to 12.8)	17.2 (12.1 to 23.5)	1.2 (0.4 to 2.3)	9.8 (5.5 to 15.4)	16.6 (11.0 to 23.5)
Algeria	8.3 (5.3 to 11.7)	9.6 (5.6 to 14.9)	32.3 (27.0 to 37.6)	10.2 (6.7 to 14.3)	9.8 (5.6 to 15.5)	30.1 (24.5 to 35.9)
Bahrain	13.7 (8.9 to 18.6)	2.5 (1.4 to 3.9)	31.0 (24.8 to 37.2)	15.4 (10.2 to 20.8)	2.5 (1.5 to 3.9)	29.1 (22.8 to 35.3)

Super-region, **region,** or country or territory	**Proportion of LOC deaths attributable to each risk factor (95% UI)**			**Proportion of LOC DALYs attributable to each risk factor (95% UI)**		
	**Alcohol use**	**Chewing tobacco**	**Smoking**	**Alcohol use**	**Chewing tobacco**	**Smoking**
Egypt	3.4 (1.6 to 5.9)	1.7 (1.0 to 2.7)	35.6 (30.1 to 41.6)	3.9 (1.8 to 6.7)	1.5 (0.9 to 2.2)	34.4 (28.6 to 40.3)
Iran (Islamic Republic of)	5.4 (3.7 to 7.5)	2.5 (1.6 to 3.6)	25.1 (20.6 to 29.6)	6.8 (4.7 to 9.3)	2.4 (1.5 to 3.5)	25.1 (20.2 to 29.8)
Iraq	5.0 (2.5 to 8.0)	1.1 (0.7 to 1.6)	31.9 (26.7 to 36.9)	5.8 (3.0 to 9.3)	1.1 (0.7 to 1.7)	28.9 (23.9 to 34.0)
Jordan	6.5 (3.2 to 10.5)	2.5 (1.5 to 3.8)	42.2 (35.5 to 48.2)	7.9 (4.0 to 12.7)	2.5 (1.5 to 3.9)	40.0 (33.2 to 47.0)
Kuwait	0.7 (0.1 to 1.8)	2.1 (1.3 to 3.2)	30.7 (24.8 to 36.5)	0.9 (0.1 to 2.3)	2.1 (1.2 to 3.3)	29.9 (23.6 to 36.2)
Lebanon	11.2 (7.6 to 15.3)	2.5 (1.5 to 3.8)	48.4 (41.6 to 54.7)	13.2 (9.0 to 18.0)	2.5 (1.5 to 3.7)	46.9 (40.0 to 53.0)
Libya	3.5 (0.8 to 5.1)	2.3 (1.5 to 3.4)	25.9 (21.3 to 30.6)	4.2 (1.0 to 6.0)	2.1 (1.3 to 3.1)	25.6 (20.3 to 30.7)
Morocco	4.2 (2.4 to 6.5)	2.5 (1.6 to 3.6)	19.8 (15.3 to 24.1)	5.1 (3.0 to 7.8)	2.4 (1.5 to 3.6)	19.8 (14.9 to 24.4)
Oman	4.0 (1.8 to 6.8)	1.4 (0.9 to 2.2)	18.0 (13.6 to 22.8)	4.8 (2.3 to 7.9)	1.5 (0.9 to 2.3)	17.1 (12.4 to 22.1)
Palestine	11.8 (8.3 to 15.8)	2.5 (1.6 to 3.6)	31.5 (26.5 to 36.6)	13.7 (9.8 to 18.3)	2.4 (1.5 to 3.5)	29.7 (24.7 to 34.5)
Qatar	7.8 (4.8 to 11.2)	1.0 (0.6 to 1.5)	24.6 (19.3 to 30.1)	9.0 (5.6 to 12.6)	1.0 (0.7 to 1.5)	23.5 (18.0 to 29.1)
Saudi Arabia	2.9 (0.5 to 6.3)	1.7 (1.1 to 2.4)	22.7 (17.8 to 27.7)	3.1 (0.5 to 6.8)	1.7 (1.1 to 2.5)	21.7 (16.7 to 26.8)
Sudan	0.2 (0.0 to 0.5)	5.0 (2.8 to 8.0)	26.2 (20.8 to 32.1)	0.3 (0.0 to 0.6)	5.5 (3.0 to 8.6)	24.6 (19.3 to 30.2)
Syrian Arab Republic	5.6 (3.4 to 8.4)	0.8 (0.5 to 1.3)	35.7 (30.0 to 41.3)	6.3 (3.8 to 9.3)	0.9 (0.5 to 1.3)	34.5 (28.9 to 40.0)
Tunisia	13.2 (9.2 to 17.4)	10.7 (6.9 to 15.1)	37.6 (32.3 to 43.1)	16.4 (11.8 to 21.2)	9.6 (6.1 to 13.8)	36.9 (31.1 to 42.7)
Turkey	11.8 (8.0 to 16.1)	0.9 (0.6 to 1.4)	37.0 (31.4 to 42.1)	15.3 (10.6 to 20.2)	0.9 (0.6 to 1.3)	39.8 (33.6 to 45.2)

***Super-region*, region,** or country or territory	**Proportion of LOC deaths attributable to each risk factor (95% UI)**			**Proportion of LOC DALYs attributable to each risk factor (95% UI)**		
	**Alcohol use**	**Chewing tobacco**	**Smoking**	**Alcohol use**	**Chewing tobacco**	**Smoking**
United Arab Emirates	16.6 (9.7 to 23.8)	2.8 (1.5 to 4.8)	29.5 (21.7 to 36.8)	17.1 (10.0 to 24.3)	2.9 (1.5 to 5.0)	28.2 (20.2 to 35.7)
Yemen	5.2 (3.0 to 7.8)	21.9 (15.8 to 28.7)	34.7 (28.7 to 40.9)	6.2 (3.7 to 9.1)	21.6 (15.5 to 28.3)	32.9 (26.8 to 39.2)
South Asia						
**South Asia**						
Bangladesh	3.7 (0.4 to 6.8)	48.3 (38.8 to 57.4)	30.4 (23.1 to 37.5)	3.9 (0.4 to 7.2)	46.4 (37.1 to 55.3)	27.8 (20.3 to 35.0)
Bhutan	10.6 (5.0 to 17.8)	43.8 (34.3 to 53.2)	16.7 (12.3 to 21.8)	12.0 (5.7 to 19.8)	41.6 (32.3 to 51.1)	15.2 (10.6 to 20.1)
India	23.0 (16.7 to 29.4)	39.6 (31.5 to 47.8)	24.1 (18.1 to 29.6)	25.3 (18.7 to 31.9)	37.9 (29.7 to 46.0)	22.0 (15.9 to 27.7)
Nepal	20.2 (9.8 to 31.3)	44.5 (34.8 to 54.3)	31.6 (22.0 to 40.4)	21.9 (10.6 to 33.4)	42.5 (32.9 to 51.7)	26.8 (17.9 to 35.1)
Pakistan	7.6 (4.9 to 10.8)	25.3 (18.6 to 32.4)	24.7 (18.4 to 30.8)	8.2 (5.2 to 11.6)	23.4 (16.9 to 30.1)	21.6 (15.5 to 28.0)
Southeast Asia, East Asia, and Oceania						
**East Asia**						
China	41.0 (33.4 to 48.2)	1.1 (0.7 to 1.8)	46.7 (40.2 to 52.8)	43.3 (35.5 to 50.4)	1.3 (0.7 to 2.1)	45.7 (39.4 to 51.8)
Democratic People's Republic of Korea	30.4 (23.1 to 37.7)	0.7 (0.4 to 1.0)	37.8 (31.1 to 44.2)	33.1 (25.2 to 40.8)	0.7 (0.4 to 1.0)	38.0 (30.9 to 44.9)
Taiwan (Province of China)	43.7 (34.9 to 51.7)	1.1 (0.6 to 1.8)	45.4 (38.5 to 51.3)	46.1 (36.8 to 54.5)	1.2 (0.6 to 1.9)	45.6 (37.8 to 52.2)
**Oceania**						
American Samoa	4.4 (1.4 to 8.4)	4.3 (2.8 to 6.3)	30.5 (22.4 to 37.5)	5.5 (1.8 to 10.5)	5.3 (3.6 to 7.5)	31.0 (22.2 to 39.1)
Cook Islands	40.5 (29.3 to 49.8)	3.3 (2.1 to 4.9)	34.6 (26.3 to 42.3)	45.1 (34.2 to 54.4)	4.3 (2.7 to 6.3)	34.5 (25.1 to 43.3)
Fiji	19.1 (13.6 to 24.7)	4.5 (3.0 to 6.4)	26.8 (18.6 to 34.7)	21.4 (15.5 to 27.3)	5.3 (3.6 to 7.6)	25.5 (17.3 to 33.3)

Super-region, **region,** or country or territory	**Proportion of LOC deaths attributable to each risk factor (95% UI)**			**Proportion of LOC DALYs attributable to each risk factor (95% UI)**		
	**Alcohol use**	**Chewing tobacco**	**Smoking**	**Alcohol use**	**Chewing tobacco**	**Smoking**
Guam	20.0 (2.0 to 37.8)	5.5 (3.3 to 8.4)	32.4 (23.9 to 40.1)	23.5 (2.4 to 43.2)	7.3 (4.5 to 11.2)	32.3 (22.9 to 40.9)
Kiribati	11.1 (2.8 to 21.1)	4.5 (2.7 to 6.6)	45.6 (35.4 to 54.2)	12.8 (3.2 to 24.0)	5.2 (3.1 to 7.6)	40.9 (30.7 to 49.5)
Marshall Islands	20.1 (10.0 to 31.5)	3.8 (2.4 to 5.6)	27.7 (19.7 to 35.5)	22.1 (11.3 to 34.0)	4.7 (3.0 to 6.9)	26.5 (18.2 to 34.5)
Micronesia (Federated States of)	18.3 (11.9 to 25.7)	5.0 (3.1 to 7.3)	39.7 (28.6 to 49.9)	21.2 (14.2 to 28.8)	5.9 (3.6 to 8.7)	37.6 (26.5 to 48.3)
Nauru	35.5 (24.9 to 45.6)	1.1 (0.6 to 1.6)	35.1 (24.5 to 45.0)	37.5 (26.6 to 47.9)	1.0 (0.6 to 1.5)	32.0 (21.8 to 41.6)
Niue	23.4 (6.4 to 34.8)	0.9 (0.6 to 1.4)	28.5 (21.5 to 35.1)	27.9 (8.2 to 40.1)	1.0 (0.6 to 1.4)	29.0 (21.0 to 36.4)
Northern Mariana Islands	17.2 (0.6 to 36.3)	3.9 (2.3 to 6.0)	38.5 (28.6 to 47.1)	19.2 (0.8 to 39.6)	4.6 (2.7 to 7.0)	37.7 (27.2 to 47.2)
Palau	16.9 (5.5 to 28.5)	25.5 (19.2 to 32.5)	26.0 (18.5 to 33.8)	19.7 (6.6 to 32.6)	29.2 (22.0 to 36.8)	25.9 (17.9 to 34.0)
Papua New Guinea	13.6 (7.0 to 21.5)	5.1 (3.1 to 7.6)	28.8 (18.7 to 38.1)	15.1 (8.0 to 23.6)	6.2 (3.8 to 9.1)	26.7 (16.7 to 35.9)
Samoa	24.4 (15.0 to 33.0)	1.6 (1.0 to 2.4)	42.5 (35.3 to 49.1)	28.1 (17.9 to 37.6)	1.7 (1.0 to 2.5)	41.7 (33.7 to 49.1)
Solomon Islands	13.2 (6.6 to 21.0)	4.0 (2.4 to 5.9)	35.6 (25.9 to 45.3)	14.4 (7.3 to 22.6)	4.5 (2.6 to 6.7)	31.8 (22.1 to 41.7)
Tokelau	18.6 (10.2 to 28.2)	4.1 (2.7 to 5.9)	30.8 (22.9 to 38.6)	21.8 (12.5 to 31.6)	5.1 (3.4 to 7.2)	30.4 (21.9 to 39.0)
Tonga	10.8 (4.4 to 18.8)	3.7 (2.5 to 5.4)	36.3 (28.8 to 43.3)	13.4 (5.8 to 23.2)	4.7 (3.1 to 6.7)	34.2 (26.2 to 41.4)
Tuvalu	14.5 (7.9 to 22.5)	4.3 (2.8 to 6.2)	34.5 (25.5 to 43.0)	17.3 (9.6 to 26.1)	5.2 (3.3 to 7.5)	33.1 (23.5 to 42.1)
Vanuatu	14.7 (8.1 to 21.7)	4.4 (2.8 to 6.4)	25.9 (17.6 to 34.5)	17.1 (9.8 to 24.6)	5.4 (3.4 to 7.9)	23.3 (15.3 to 31.7)
**Southeast Asia**						
Cambodia	37.7 (29.3 to 46.2)	27.3 (21.0 to 33.8)	39.6 (32.7 to 46.9)	41.2 (32.1 to 49.7)	23.0 (17.5 to 29.3)	37.2 (30.2 to 44.8)

Super-region, **region,** or country or territory	**Proportion of LOC deaths attributable to each risk factor (95% UI)**			**Proportion of LOC DALYs attributable to each risk factor (95% UI)**		
	**Alcohol use**	**Chewing tobacco**	**Smoking**	**Alcohol use**	**Chewing tobacco**	**Smoking**
Indonesia	3.0 (0.7 to 6.4)	11.3 (7.1 to 16.1)	36.7 (28.7 to 44.3)	3.6 (0.8 to 7.4)	8.1 (4.9 to 12.0)	35.7 (27.1 to 43.7)
Lao People's Democratic Republic	34.6 (23.2 to 45.0)	18.1 (12.8 to 24.3)	37.0 (29.9 to 44.0)	38.6 (26.5 to 49.2)	14.5 (10.0 to 20.1)	34.5 (27.0 to 41.6)
Malaysia	11.9 (6.3 to 17.7)	9.4 (6.5 to 13.3)	28.1 (22.3 to 33.2)	13.8 (7.5 to 20.1)	9.1 (6.3 to 12.9)	27.5 (21.1 to 33.0)
Maldives	6.1 (1.6 to 12.5)	25.3 (17.6 to 33.1)	28.3 (23.0 to 33.4)	8.1 (2.3 to 16.4)	22.8 (16.4 to 29.7)	27.8 (21.9 to 33.1)
Mauritius	31.5 (22.0 to 40.4)	12.4 (8.5 to 16.6)	30.8 (23.8 to 37.4)	34.0 (24.2 to 43.3)	10.2 (7.1 to 13.9)	30.1 (22.5 to 37.4)
Myanmar	23.6 (16.1 to 31.1)	28.6 (21.1 to 36.6)	35.9 (27.2 to 44.3)	26.7 (18.6 to 34.4)	28.7 (21.2 to 36.4)	32.5 (23.7 to 40.6)
Philippines	36.6 (28.9 to 44.4)	8.7 (5.8 to 12.1)	37.8 (30.0 to 44.9)	40.9 (33.1 to 48.9)	7.1 (4.8 to 10.0)	35.7 (27.7 to 43.0)
Seychelles	46.4 (35.8 to 55.9)	4.4 (2.8 to 6.3)	41.8 (34.6 to 48.4)	50.3 (39.6 to 59.7)	2.8 (1.8 to 4.0)	40.5 (32.5 to 47.9)
Sri Lanka	34.1 (26.7 to 41.1)	32.5 (24.5 to 41.7)	27.0 (19.2 to 34.3)	37.6 (29.9 to 44.9)	31.1 (22.9 to 40.3)	26.3 (18.3 to 33.9)
Thailand	34.8 (27.9 to 41.2)	19.2 (13.3 to 25.5)	30.4 (24.2 to 36.3)	41.5 (34.3 to 48.1)	13.1 (9.2 to 17.2)	31.7 (24.6 to 38.5)
Timor-Leste	23.4 (14.3 to 32.9)	12.7 (8.6 to 18.2)	34.2 (25.5 to 42.2)	25.8 (16.2 to 35.8)	10.4 (6.9 to 15.3)	32.8 (23.8 to 41.0)
Viet Nam	44.8 (36.7 to 52.0)	7.6 (4.9 to 10.7)	38.6 (30.7 to 45.4)	47.8 (39.6 to 55.0)	5.1 (3.4 to 7.1)	38.4 (29.9 to 45.6)
Sub-Saharan Africa						
**Central sub-Saharan Africa**						
Angola	46.4 (38.5 to 54.5)	1.6 (1.0 to 2.4)	22.9 (16.5 to 29.4)	47.6 (39.6 to 55.6)	1.5 (1.0 to 2.2)	21.7 (15.1 to 28.1)
Central African Republic	24.9 (13.9 to 36.1)	3.0 (1.7 to 4.8)	16.3 (10.1 to 23.5)	25.8 (14.6 to 37.1)	2.7 (1.5 to 4.2)	15.6 (9.4 to 22.9)
Congo	36.2 (23.2 to 48.3)	3.7 (2.2 to 5.5)	17.8 (11.9 to 23.6)	37.4 (24.5 to 49.8)	3.4 (2.1 to 5.1)	17.0 (10.9 to 22.9)

Super-region, **region,** or country or territory	**Proportion of LOC deaths attributable to each risk factor (95% UI)**			**Proportion of LOC DALYs attributable to each risk factor (95% UI)**		
	**Alcohol use**	**Chewing tobacco**	**Smoking**	**Alcohol use**	**Chewing tobacco**	**Smoking**
Democratic Republic of the Congo	19.2 (9.3 to 29.9)	6.4 (4.1 to 9.2)	13.7 (8.2 to 19.7)	20.5 (10.0 to 31.2)	5.9 (3.8 to 8.5)	13.3 (8.0 to 19.3)
Equatorial Guinea	42.8 (30.9 to 53.5)	3.7 (2.3 to 5.6)	17.7 (10.9 to 24.6)	44.6 (32.5 to 54.8)	3.2 (2.1 to 4.8)	16.8 (10.2 to 23.8)
Gabon	48.2 (37.9 to 57.4)	1.8 (1.0 to 2.8)	18.1 (11.4 to 24.4)	49.8 (39.8 to 58.9)	1.5 (0.9 to 2.3)	17.9 (11.0 to 24.4)
**Eastern sub-Saharan Africa**						
Burundi	41.9 (32.8 to 50.9)	9.7 (6.4 to 13.8)	15.8 (9.6 to 22.2)	43.0 (33.8 to 52.3)	10.0 (6.6 to 14.2)	14.3 (8.5 to 20.5)
Comoros	5.6 (1.4 to 12.1)	22.5 (16.4 to 29.1)	17.4 (11.3 to 23.1)	6.0 (1.5 to 13.0)	22.5 (16.1 to 29.0)	15.8 (10.0 to 21.7)
Djibouti	4.7 (1.3 to 9.3)	4.7 (3.0 to 6.9)	27.9 (19.0 to 36.2)	4.9 (1.4 to 9.6)	4.6 (2.9 to 6.7)	25.7 (17.0 to 34.2)
Eritrea	15.6 (8.2 to 24.5)	5.0 (2.8 to 8.1)	13.0 (7.6 to 18.2)	16.3 (8.6 to 25.6)	5.0 (2.8 to 8.2)	13.0 (7.6 to 18.3)
Ethiopia	23.4 (13.8 to 32.9)	4.5 (2.9 to 6.8)	6.7 (3.9 to 9.9)	24.6 (14.8 to 34.2)	4.2 (2.7 to 6.4)	6.0 (3.4 to 8.9)
Kenya	29.3 (20.7 to 37.9)	6.1 (4.0 to 8.7)	15.0 (9.2 to 20.6)	30.4 (21.6 to 39.3)	5.8 (3.8 to 8.3)	13.7 (8.2 to 19.1)
Madagascar	18.0 (9.4 to 27.8)	27.7 (19.7 to 35.9)	13.2 (7.9 to 18.8)	18.5 (9.6 to 28.5)	29.7 (21.4 to 38.3)	12.3 (7.2 to 17.6)
Malawi	23.0 (14.5 to 32.0)	6.1 (3.9 to 9.1)	15.3 (9.3 to 21.4)	24.9 (15.9 to 34.3)	5.3 (3.4 to 8.2)	13.4 (7.8 to 19.2)
Mozambique	19.3 (9.7 to 30.5)	7.1 (4.6 to 10.0)	20.8 (13.1 to 28.6)	20.4 (10.3 to 32.3)	6.7 (4.4 to 9.5)	19.5 (12.0 to 26.8)
Rwanda	44.7 (34.3 to 54.5)	8.2 (5.0 to 12.4)	25.5 (17.4 to 33.2)	46.6 (36.1 to 56.6)	7.6 (4.7 to 11.5)	21.4 (14.1 to 28.5)
Somalia	0.0 (0.0 to 0.0)	4.9 (3.2 to 6.9)	15.3 (8.9 to 22.0)	0.0 (0.0 to 0.0)	4.6 (3.0 to 6.6)	13.9 (7.9 to 20.2)
South Sudan	4.1 (1.1 to 8.2)	4.7 (3.1 to 6.8)	17.1 (10.3 to 24.6)	4.4 (1.1 to 8.8)	4.6 (2.9 to 6.6)	15.4 (8.9 to 22.6)
Uganda	49.6 (39.9 to 58.1)	4.1 (2.7 to 6.0)	14.2 (8.5 to 19.7)	51.7 (42.2 to 60.3)	3.4 (2.2 to 4.8)	12.7 (7.5 to 17.8)

Super-region, **region,** or country or territory	**Proportion of LOC deaths attributable to each risk factor (95% UI)**			**Proportion of LOC DALYs attributable to each risk factor (95% UI)**		
	**Alcohol use**	**Chewing tobacco**	**Smoking**	**Alcohol use**	**Chewing tobacco**	**Smoking**
United Republic of Tanzania	42.9 (33.3 to 51.9)	4.2 (2.7 to 6.2)	21.2 (13.4 to 29.0)	43.8 (34.3 to 52.9)	3.7 (2.4 to 5.3)	19.2 (12.0 to 26.8)
Zambia	35.5 (25.1 to 44.8)	4.4 (2.9 to 6.3)	18.0 (11.3 to 24.8)	37.4 (26.8 to 46.6)	3.8 (2.5 to 5.3)	15.3 (9.4 to 21.4)
**Southern sub-Saharan Africa**						
Botswana	39.3 (30.4 to 47.5)	9.7 (6.6 to 13.5)	30.0 (21.9 to 37.5)	41.4 (32.4 to 49.5)	9.2 (6.3 to 12.8)	28.2 (19.9 to 35.9)
Eswatini	41.3 (32.5 to 49.4)	1.4 (0.9 to 2.1)	14.2 (8.8 to 19.8)	43.0 (34.3 to 50.9)	1.3 (0.8 to 2.0)	12.8 (7.7 to 18.1)
Lesotho	36.8 (25.4 to 48.3)	4.0 (2.5 to 5.8)	33.9 (26.6 to 41.3)	39.5 (27.6 to 51.1)	3.5 (2.2 to 5.1)	33.4 (25.5 to 41.2)
Namibia	42.5 (30.8 to 52.1)	4.7 (3.0 to 6.8)	21.8 (13.9 to 29.5)	46.1 (34.1 to 55.5)	3.6 (2.4 to 5.2)	18.3 (11.2 to 25.5)
South Africa	43.5 (35.6 to 50.9)	5.6 (3.5 to 8.3)	32.6 (25.0 to 39.5)	45.9 (37.8 to 53.5)	4.4 (2.8 to 6.4)	32.3 (24.3 to 40.0)
Zimbabwe	24.5 (15.5 to 33.3)	3.9 (2.3 to 6.0)	23.2 (17.1 to 29.0)	27.1 (17.8 to 36.1)	2.7 (1.7 to 4.1)	20.6 (14.7 to 26.5)
**Western sub-Saharan Africa**						
Benin	22.8 (14.1 to 31.4)	8.9 (5.9 to 12.7)	13.0 (8.7 to 17.8)	25.1 (15.9 to 34.5)	8.1 (5.3 to 11.8)	11.9 (7.6 to 16.6)
Burkina Faso	43.1 (30.9 to 54.8)	14.3 (10.0 to 19.4)	10.6 (6.2 to 15.4)	44.6 (32.7 to 56.0)	14.4 (10.2 to 19.5)	10.9 (6.3 to 15.9)
Cabo Verde	33.8 (25.3 to 43.0)	10.4 (6.6 to 15.2)	12.5 (8.1 to 17.3)	38.9 (30.3 to 48.1)	8.8 (5.9 to 12.6)	14.2 (8.9 to 19.7)
Cameroon	39.8 (31.3 to 48.6)	6.3 (4.1 to 9.1)	15.5 (10.5 to 20.7)	41.7 (33.2 to 50.2)	5.4 (3.5 to 7.8)	15.2 (10.0 to 20.8)
Chad	24.8 (10.5 to 37.7)	8.5 (5.7 to 12.0)	15.9 (10.1 to 21.6)	27.1 (12.0 to 40.4)	7.5 (5.0 to 10.5)	14.9 (9.3 to 20.8)
Côte d'Ivoire	37.2 (25.5 to 47.8)	3.8 (2.4 to 5.7)	20.9 (15.0 to 26.8)	39.5 (27.7 to 50.1)	3.2 (2.0 to 4.9)	20.9 (14.7 to 27.3)
Gambia	22.7 (14.4 to 30.6)	5.2 (3.3 to 7.8)	14.9 (10.5 to 19.2)	24.4 (15.5 to 32.6)	4.4 (2.8 to 6.5)	14.3 (9.7 to 18.7)

***Super-region*, region,** or country or territory	**Proportion of LOC deaths attributable to each risk factor (95% UI)**			**Proportion of LOC DALYs attributable to each risk factor (95% UI)**		
	**Alcohol use**	**Chewing tobacco**	**Smoking**	**Alcohol use**	**Chewing tobacco**	**Smoking**
Ghana	33.1 (23.4 to 43.3)	4.7 (3.2 to 6.9)	9.9 (6.1 to 14.0)	34.1 (24.2 to 44.5)	4.3 (2.8 to 6.1)	9.0 (5.3 to 12.9)
Guinea	12.7 (6.9 to 19.5)	5.0 (3.3 to 7.3)	20.8 (14.4 to 26.9)	13.4 (7.6 to 20.7)	4.4 (2.9 to 6.4)	19.4 (13.1 to 25.4)
Guinea-Bissau	28.6 (19.4 to 37.7)	4.9 (3.1 to 7.2)	8.9 (5.6 to 12.8)	30.6 (21.0 to 39.7)	4.3 (2.8 to 6.3)	8.7 (5.3 to 12.6)
Liberia	30.8 (21.1 to 40.3)	3.7 (2.4 to 5.5)	12.5 (7.7 to 17.7)	34.2 (24.1 to 44.1)	3.1 (2.1 to 4.6)	12.0 (7.1 to 17.3)
Mali	12.3 (7.3 to 18.0)	9.0 (6.0 to 12.7)	14.1 (8.6 to 19.8)	12.4 (7.5 to 17.9)	8.9 (5.9 to 12.6)	12.7 (7.4 to 18.0)
Mauritania	0.0 (0.0 to 0.0)	6.0 (3.8 to 9.3)	17.0 (11.5 to 23.3)	0.0 (0.0 to 0.0)	5.6 (3.6 to 8.4)	17.8 (11.6 to 24.6)
Niger	4.3 (1.4 to 7.9)	9.4 (6.1 to 13.2)	8.1 (4.9 to 11.6)	4.7 (1.6 to 8.7)	9.5 (6.2 to 13.4)	8.0 (4.8 to 11.7)
Nigeria	29.7 (21.7 to 37.7)	2.3 (1.5 to 3.5)	7.8 (4.8 to 11.0)	29.9 (22.0 to 38.1)	2.1 (1.3 to 3.1)	7.3 (4.3 to 10.4)
São Tomé and Príncipe	38.9 (28.9 to 47.6)	2.9 (1.8 to 4.3)	13.0 (8.6 to 17.7)	41.0 (31.1 to 49.6)	2.5 (1.6 to 3.5)	12.4 (8.0 to 17.1)
Senegal	5.1 (2.6 to 8.3)	3.9 (2.3 to 6.2)	14.9 (10.5 to 19.8)	6.5 (3.5 to 10.2)	3.2 (1.9 to 4.9)	15.3 (10.4 to 20.7)
Sierra Leone	31.7 (22.2 to 41.2)	7.5 (5.0 to 11.0)	19.9 (13.0 to 27.0)	33.9 (24.0 to 43.3)	6.8 (4.6 to 9.6)	19.5 (12.4 to 26.7)
Togo	24.6 (15.7 to 33.6)	6.0 (3.9 to 8.8)	18.7 (13.9 to 23.9)	26.5 (17.1 to 35.5)	5.3 (3.4 to 7.7)	17.7 (12.7 to 23.0)

DALYs=disability-adjusted life-years; UI=uncertainty interval.

eTable 13. **Proportion of Other pharynx cancer (OPC) deaths and DALYs attributable to risk factors, in 2019, by country or territory, both sexes combined**

***Super-region*, region,** or country or territory	Proportion of OPC deaths attributable to each risk factor (95% UI)		Proportion of OPC DALYs attributable to each risk factor (95% UI)	
	**Alcohol use**	**Smoking**	**Alcohol use**	**Smoking**
Central Europe, Eastern Europe, and Central Asia				
**Central Asia**				
Armenia	31.9 (22.1 to 41.5)	63.4 (58.6 to 67.4)	34.8 (24.4 to 44.7)	63.8 (58.6 to 68.1)
Azerbaijan	42.9 (31.7 to 53.4)	52.9 (46.0 to 59.6)	43.9 (32.5 to 54.6)	50.8 (43.5 to 58.0)
Georgia	44.6 (35.4 to 53.1)	61.2 (55.9 to 65.8)	47.9 (38.6 to 56.3)	62.5 (56.5 to 67.4)
Kazakhstan	41.7 (31.3 to 50.7)	55.3 (49.2 to 60.8)	44.0 (33.4 to 53.1)	54.8 (47.9 to 60.8)
Kyrgyzstan	36.0 (27.0 to 43.9)	52.5 (47.2 to 57.6)	37.7 (28.6 to 46.2)	49.7 (43.9 to 55.0)
Mongolia	37.3 (28.4 to 45.9)	47.4 (39.4 to 54.3)	42.2 (32.9 to 51.0)	47.8 (39.4 to 55.1)
Tajikistan	18.6 (12.0 to 25.0)	29.9 (25.4 to 35.2)	19.8 (13.0 to 26.8)	27.5 (23.0 to 32.4)
Turkmenistan	40.8 (31.4 to 49.5)	50.5 (45.1 to 55.4)	41.9 (32.5 to 50.9)	49.5 (43.8 to 54.7)
Uzbekistan	27.7 (19.6 to 35.8)	38.4 (32.8 to 43.6)	29.1 (20.9 to 37.5)	35.7 (30.0 to 40.8)
**Central Europe**				
Albania	38.2 (28.2 to 47.4)	60.2 (54.8 to 64.9)	40.9 (30.8 to 50.0)	59.2 (53.5 to 64.3)
Bosnia and Herzegovina	48.6 (39.4 to 56.6)	67.9 (62.6 to 72.6)	51.2 (41.7 to 59.5)	68.4 (62.7 to 73.3)
Bulgaria	60.7 (51.7 to 69.1)	66.7 (61.2 to 71.4)	62.2 (53.2 to 70.5)	67.9 (62.0 to 72.6)
Croatia	57.9 (46.9 to 66.5)	64.7 (59.2 to 69.8)	58.7 (47.8 to 67.4)	65.2 (59.3 to 70.6)

Super-region, **region,** or country or territory	**Proportion of OPC deaths attributable to each risk factor (95% UI)**		**Proportion of OPC DALYs attributable to each risk factor (95% UI)**	
	**Alcohol use**	**Smoking**	**Alcohol use**	**Smoking**
Czechia	63.0 (54.0 to 70.8)	61.5 (55.0 to 67.0)	64.0 (55.2 to 72.0)	61.5 (54.6 to 67.4)
Hungary	58.8 (49.9 to 66.8)	64.2 (57.8 to 69.6)	59.5 (50.6 to 67.6)	64.6 (58.0 to 70.3)
Montenegro	56.9 (46.8 to 65.6)	69.8 (64.3 to 74.4)	57.5 (47.2 to 66.3)	69.4 (63.4 to 74.3)
North Macedonia	52.0 (42.1 to 60.2)	66.5 (60.3 to 71.5)	53.4 (43.3 to 61.5)	66.0 (59.4 to 71.3)
Poland	56.6 (47.5 to 64.4)	62.0 (55.2 to 67.4)	58.6 (49.5 to 66.3)	62.1 (55.3 to 67.9)
Romania	61.0 (51.6 to 69.4)	63.1 (56.5 to 68.8)	62.1 (52.8 to 70.3)	63.7 (56.8 to 69.7)
Serbia	53.4 (43.5 to 62.1)	65.9 (59.6 to 71.1)	55.1 (45.1 to 63.9)	66.6 (60.0 to 71.8)
Slovakia	62.2 (53.2 to 70.2)	59.9 (52.7 to 66.4)	63.0 (54.0 to 71.0)	60.0 (52.6 to 66.9)
Slovenia	49.6 (28.8 to 63.6)	60.9 (55.4 to 66.3)	50.0 (29.1 to 64.2)	62.0 (56.0 to 67.1)
**Eastern Europe**				
Belarus	63.3 (54.2 to 71.6)	67.9 (61.3 to 73.1)	64.1 (55.2 to 72.4)	67.9 (61.0 to 73.2)
Estonia	63.0 (53.9 to 70.9)	65.5 (59.1 to 70.7)	64.1 (54.9 to 71.9)	66.0 (59.4 to 71.4)
Latvia	60.8 (51.1 to 68.8)	65.5 (59.2 to 71.0)	62.3 (52.9 to 70.2)	66.2 (59.6 to 71.8)
Lithuania	62.4 (52.6 to 70.7)	61.9 (54.8 to 68.0)	63.5 (53.7 to 71.7)	62.3 (54.9 to 68.7)
Republic of Moldova	61.2 (51.0 to 69.5)	67.6 (61.8 to 72.6)	62.0 (52.1 to 70.2)	67.8 (61.8 to 72.6)
Russian Federation	56.9 (47.3 to 65.5)	66.3 (60.4 to 71.6)	58.5 (48.9 to 67.0)	66.8 (60.9 to 72.3)
Ukraine	55.0 (43.5 to 65.1)	67.3 (61.0 to 72.5)	56.4 (45.0 to 66.4)	67.1 (60.6 to 72.6)

Super-region, **region,** or country or territory	**Proportion of OPC deaths attributable to each risk factor (95% UI)**		**Proportion of OPC DALYs attributable to each risk factor (95% UI)**	
	**Alcohol use**	**Smoking**	**Alcohol use**	**Smoking**
High-income				
**Australasia**				
Australia	57.6 (47.8 to 66.6)	45.3 (38.7 to 51.5)	59.3 (49.4 to 68.1)	48.2 (41.2 to 54.8)
New Zealand	54.4 (44.0 to 63.7)	47.6 (39.9 to 54.5)	56.1 (45.7 to 65.2)	48.4 (40.5 to 55.7)
**High-income Asia Pacific**				
Brunei Darussalam	4.7 (0.0 to 10.2)	49.2 (43.1 to 55.1)	5.1 (0.0 to 10.9)	47.6 (41.3 to 53.6)
Japan	44.4 (33.1 to 54.6)	58.4 (52.8 to 63.4)	47.0 (35.7 to 57.1)	60.0 (54.0 to 64.9)
Republic of Korea	56.5 (46.6 to 65.4)	62.5 (57.5 to 67.6)	58.2 (48.3 to 67.0)	62.5 (57.3 to 67.5)
Singapore	21.5 (13.6 to 30.5)	46.3 (39.9 to 52.3)	23.9 (15.3 to 33.4)	46.7 (39.7 to 52.9)
**High-income North America**				
Canada	49.8 (39.4 to 59.0)	53.5 (47.5 to 59.5)	52.0 (41.2 to 61.2)	54.0 (47.6 to 60.1)
Greenland	46.9 (33.4 to 58.9)	65.8 (59.2 to 71.8)	48.2 (34.5 to 60.1)	65.2 (58.2 to 71.5)
United States of America	47.0 (36.8 to 56.1)	56.2 (50.4 to 62.2)	49.3 (38.9 to 58.2)	56.3 (50.6 to 62.1)
**Southern Latin America**				
Argentina	55.3 (44.9 to 64.4)	56.2 (49.0 to 62.6)	56.6 (46.1 to 65.7)	56.7 (49.2 to 63.4)
Chile	51.8 (41.2 to 61.2)	47.9 (40.3 to 55.6)	53.8 (43.3 to 63.1)	52.1 (44.2 to 59.8)
Uruguay	52.7 (42.4 to 61.6)	57.6 (50.8 to 63.4)	54.1 (43.4 to 63.3)	59.7 (52.8 to 65.8)
**Western Europe**				

***Super-region*, region,** or country or territory	**Proportion of OPC deaths attributable to each risk factor (95% UI)**		**Proportion of OPC DALYs attributable to each risk factor (95% UI)**	
	**Alcohol use**	**Smoking**	**Alcohol use**	**Smoking**
Andorra	60.0 (50.4 to 68.4)	59.5 (52.0 to 65.8)	60.9 (51.3 to 69.2)	59.7 (51.6 to 66.7)
Austria	59.6 (50.2 to 68.1)	60.7 (54.5 to 66.2)	60.7 (51.2 to 69.0)	61.8 (55.3 to 67.6)
Belgium	60.1 (50.4 to 68.4)	58.6 (52.6 to 64.2)	61.2 (51.6 to 69.5)	59.1 (52.5 to 65.0)
Cyprus	51.4 (41.2 to 60.5)	61.0 (55.1 to 66.1)	53.0 (42.8 to 62.1)	61.5 (55.3 to 66.8)
Denmark	57.7 (47.5 to 66.7)	59.5 (53.0 to 65.1)	58.5 (48.4 to 67.5)	58.6 (51.9 to 64.8)
Finland	51.3 (41.1 to 60.9)	51.7 (44.5 to 57.9)	54.2 (44.1 to 63.3)	53.3 (45.9 to 59.6)
France	62.0 (51.9 to 70.5)	57.1 (50.1 to 63.5)	62.7 (52.9 to 71.3)	58.3 (50.6 to 65.1)
Germany	63.9 (54.7 to 72.0)	59.4 (52.9 to 65.1)	64.7 (55.5 to 72.7)	60.5 (53.7 to 66.2)
Greece	48.8 (39.3 to 57.2)	64.3 (59.6 to 68.6)	52.3 (42.5 to 60.8)	66.5 (61.4 to 71.1)
Iceland	52.0 (42.0 to 61.5)	53.2 (47.4 to 59.2)	53.7 (43.6 to 63.0)	52.9 (46.3 to 59.1)
Ireland	54.2 (44.0 to 63.2)	55.3 (49.1 to 61.0)	55.8 (45.6 to 64.8)	55.0 (48.2 to 61.1)
Israel	26.4 (17.3 to 35.7)	51.9 (45.8 to 57.5)	29.7 (20.0 to 39.4)	52.0 (45.6 to 57.7)
Italy	54.0 (44.1 to 62.8)	55.8 (49.4 to 61.5)	55.6 (45.6 to 64.5)	56.8 (50.0 to 62.6)
Luxembourg	60.2 (50.7 to 68.9)	55.9 (48.8 to 62.2)	61.3 (51.9 to 69.8)	55.8 (48.1 to 62.5)
Malta	50.7 (40.7 to 59.6)	55.6 (49.8 to 61.2)	52.4 (42.1 to 61.4)	56.6 (50.6 to 62.2)
Monaco	38.2 (2.6 to 62.2)	54.6 (47.3 to 60.9)	40.1 (2.9 to 63.9)	56.3 (48.8 to 62.8)

Super-region, **region,** or country or territory	**Proportion of OPC deaths attributable to each risk factor (95% UI)**		**Proportion of OPC DALYs attributable to each risk factor (95% UI)**	
	**Alcohol use**	**Smoking**	**Alcohol use**	**Smoking**
Netherlands	56.5 (46.5 to 65.1)	58.7 (52.1 to 64.7)	57.5 (47.7 to 66.1)	58.6 (51.6 to 64.9)
Norway	48.0 (37.0 to 57.9)	47.4 (40.2 to 54.0)	49.9 (39.0 to 59.6)	49.8 (42.5 to 56.8)
Portugal	61.6 (52.7 to 69.7)	57.2 (50.7 to 63.3)	62.9 (54.2 to 71.2)	58.9 (51.7 to 65.4)
San Marino	53.4 (7.7 to 67.7)	53.0 (46.0 to 59.4)	54.8 (8.3 to 69.1)	53.9 (46.5 to 60.6)
Spain	55.6 (45.3 to 64.5)	65.1 (60.0 to 69.9)	57.3 (46.9 to 66.4)	66.5 (60.8 to 71.5)
Sweden	56.0 (46.0 to 65.1)	49.8 (43.2 to 56.5)	57.4 (47.3 to 66.4)	49.1 (41.9 to 55.8)
Switzerland	59.1 (48.6 to 67.9)	57.1 (51.0 to 62.8)	59.8 (49.4 to 68.6)	57.2 (50.5 to 62.9)
United Kingdom	51.2 (40.8 to 60.9)	57.6 (51.6 to 63.6)	53.3 (42.9 to 62.8)	58.3 (51.8 to 64.1)
Latin America and Caribbean				
**Andean Latin America**				
Bolivia (Plurinational State of)	29.6 (20.5 to 38.6)	28.5 (21.4 to 35.9)	33.0 (23.2 to 42.0)	26.6 (19.6 to 33.7)
Ecuador	24.4 (17.4 to 31.6)	33.4 (26.5 to 41.1)	29.1 (21.4 to 37.0)	31.1 (23.9 to 38.5)
Peru	32.0 (22.2 to 42.1)	18.9 (12.8 to 26.0)	37.0 (26.6 to 47.5)	17.7 (11.8 to 24.3)
**Caribbean**				
Antigua and Barbuda	40.8 (31.0 to 49.9)	38.2 (31.4 to 45.0)	44.2 (34.1 to 53.3)	38.2 (31.2 to 45.0)
Bahamas	37.5 (20.5 to 50.4)	34.4 (27.5 to 41.5)	40.5 (22.8 to 53.8)	33.7 (26.8 to 41.0)
Barbados	46.2 (37.5 to 54.7)	34.7 (28.6 to 41.0)	49.7 (40.7 to 58.1)	34.9 (28.4 to 41.8)

Super-region, **region,** or country or territory	**Proportion of OPC deaths attributable to each risk factor (95% UI)**		**Proportion of OPC DALYs attributable to each risk factor (95% UI)**	
	**Alcohol use**	**Smoking**	**Alcohol use**	**Smoking**
Belize	38.3 (29.7 to 46.3)	40.5 (33.3 to 47.7)	41.5 (32.6 to 49.7)	38.6 (31.1 to 46.3)
Bermuda	47.4 (36.4 to 57.3)	44.4 (36.8 to 52.0)	51.2 (40.3 to 60.6)	45.5 (37.6 to 53.6)
Cuba	39.4 (29.7 to 48.1)	60.8 (54.4 to 66.2)	43.0 (33.0 to 52.0)	61.1 (54.4 to 67.0)
Dominica	43.3 (33.4 to 52.4)	36.9 (29.3 to 44.7)	47.1 (36.9 to 56.4)	37.4 (29.5 to 45.5)
Dominican Republic	34.0 (25.2 to 42.5)	48.2 (41.2 to 54.4)	39.0 (29.6 to 47.9)	44.0 (37.1 to 50.5)
Grenada	45.8 (36.3 to 54.8)	38.8 (30.5 to 46.5)	48.8 (39.0 to 58.0)	38.9 (30.3 to 46.7)
Guyana	43.8 (34.7 to 51.7)	38.9 (31.4 to 47.1)	46.1 (36.8 to 53.9)	38.9 (30.8 to 47.1)
Haiti	39.1 (29.8 to 48.4)	25.9 (18.4 to 34.0)	40.7 (31.2 to 50.2)	24.9 (17.5 to 33.3)
Jamaica	32.5 (23.4 to 40.7)	43.9 (36.7 to 50.4)	35.6 (26.2 to 44.2)	43.2 (35.9 to 50.1)
Puerto Rico	37.9 (29.2 to 46.6)	42.3 (35.2 to 49.6)	42.0 (32.8 to 51.0)	42.9 (35.2 to 50.7)
Saint Kitts and Nevis	26.7 (1.8 to 50.2)	34.9 (28.5 to 41.7)	28.7 (2.0 to 52.8)	35.8 (28.9 to 43.1)
Saint Lucia	48.1 (37.9 to 56.7)	43.5 (36.4 to 50.7)	51.5 (41.2 to 60.4)	43.8 (36.3 to 51.2)
Saint Vincent and the Grenadines	44.5 (35.3 to 53.1)	37.4 (30.1 to 45.1)	48.1 (38.7 to 56.6)	37.6 (29.7 to 45.9)
Suriname	38.2 (29.3 to 47.2)	50.4 (43.7 to 57.3)	41.6 (32.2 to 50.8)	50.8 (43.6 to 58.1)
Trinidad and Tobago	43.5 (33.9 to 52.6)	46.3 (38.9 to 53.6)	46.5 (36.4 to 55.7)	45.9 (37.8 to 53.6)
United States Virgin Islands	33.9 (3.7 to 55.0)	34.6 (27.9 to 41.3)	36.2 (4.1 to 57.7)	34.6 (27.7 to 41.4)
**Central Latin America**				

Super-region, **region,** or country or territory	Proportion of OPC deaths attributable to each risk factor (95% UI)		Proportion of OPC DALYs attributable to each risk factor (95% UI)	
	**Alcohol use**	**Smoking**	**Alcohol use**	**Smoking**
Colombia	27.9 (20.7 to 35.0)	33.9 (26.4 to 41.1)	32.8 (24.9 to 40.4)	34.5 (26.7 to 42.2)
Costa Rica	34.7 (25.0 to 43.7)	42.5 (35.2 to 49.6)	38.4 (28.4 to 47.6)	41.4 (34.2 to 48.8)
El Salvador	25.3 (17.9 to 33.3)	28.9 (22.3 to 35.7)	29.4 (21.5 to 38.1)	29.3 (22.5 to 36.4)
Guatemala	16.4 (10.3 to 22.9)	28.9 (21.6 to 36.0)	20.4 (13.5 to 27.6)	25.4 (18.1 to 32.3)
Honduras	23.2 (16.9 to 30.1)	37.1 (30.0 to 43.9)	24.8 (18.3 to 31.9)	35.2 (27.9 to 42.5)
Mexico	37.2 (28.6 to 46.1)	37.0 (28.9 to 44.8)	40.6 (31.6 to 49.6)	34.0 (25.8 to 41.7)
Nicaragua	28.6 (21.0 to 36.4)	37.8 (30.7 to 45.0)	30.7 (22.8 to 38.2)	36.4 (28.9 to 44.0)
Panama	38.9 (30.5 to 47.1)	33.2 (26.9 to 39.4)	43.0 (34.1 to 51.5)	31.7 (25.4 to 38.3)
Venezuela (Bolivarian Republic of)	38.3 (29.3 to 47.3)	41.8 (33.6 to 50.1)	41.9 (32.7 to 50.9)	40.7 (32.0 to 49.3)
**Tropical Latin America**				
Brazil	42.2 (33.2 to 50.6)	49.0 (42.2 to 55.9)	45.1 (35.8 to 53.8)	47.7 (40.3 to 54.9)
Paraguay	52.8 (43.5 to 62.2)	55.5 (48.0 to 62.3)	54.7 (45.2 to 64.1)	52.5 (44.4 to 59.9)
North Africa and Middle East				
**North Africa and Middle East**				
Afghanistan	1.0 (0.4 to 1.8)	21.9 (16.3 to 29.3)	1.1 (0.5 to 2.1)	20.3 (14.4 to 28.5)
Algeria	10.7 (6.5 to 15.6)	47.9 (41.6 to 53.2)	12.2 (7.6 to 17.6)	45.6 (39.1 to 51.5)
Bahrain	14.2 (8.8 to 20.3)	43.9 (37.5 to 50.5)	15.9 (9.9 to 22.4)	41.8 (34.9 to 48.8)

***Super-region*, region,** or country or territory	**Proportion of OPC deaths attributable to each risk factor (95% UI)**		**Proportion of OPC DALYs attributable to each risk factor (95% UI)**	
	**Alcohol use**	**Smoking**	**Alcohol use**	**Smoking**
Egypt	4.6 (2.3 to 8.0)	48.6 (42.3 to 54.3)	5.1 (2.5 to 8.7)	47.0 (40.5 to 52.9)
Iran (Islamic Republic of)	5.7 (3.8 to 8.2)	38.4 (33.2 to 43.3)	6.9 (4.7 to 9.7)	37.8 (32.5 to 43.1)
Iraq	6.3 (3.3 to 9.9)	47.6 (42.0 to 53.1)	7.2 (3.8 to 11.1)	43.9 (38.0 to 49.7)
Jordan	7.7 (3.8 to 12.3)	57.9 (52.2 to 63.2)	8.9 (4.5 to 14.1)	55.8 (49.4 to 61.8)
Kuwait	1.1 (0.2 to 2.7)	46.8 (40.7 to 52.1)	1.3 (0.2 to 3.2)	45.2 (38.8 to 51.1)
Lebanon	12.4 (8.0 to 17.1)	61.9 (56.1 to 67.1)	13.6 (8.6 to 18.7)	59.9 (53.6 to 65.8)
Libya	3.5 (0.9 to 5.1)	36.3 (30.2 to 42.5)	4.0 (1.0 to 5.8)	34.3 (27.9 to 40.9)
Morocco	6.0 (3.4 to 9.3)	35.7 (29.3 to 41.9)	6.7 (3.9 to 10.3)	35.2 (28.3 to 41.6)
Oman	5.7 (2.9 to 9.3)	32.9 (26.3 to 40.2)	6.5 (3.3 to 10.3)	31.7 (24.8 to 39.3)
Palestine	12.1 (8.2 to 16.7)	44.4 (39.0 to 49.4)	13.7 (9.4 to 18.7)	42.5 (36.8 to 47.7)
Qatar	10.4 (6.3 to 15.4)	46.2 (39.4 to 52.3)	11.6 (7.1 to 17.0)	44.0 (37.2 to 50.5)
Saudi Arabia	3.5 (0.8 to 7.3)	33.1 (27.3 to 38.7)	3.7 (0.8 to 7.7)	31.3 (25.5 to 37.1)
Sudan	0.3 (0.1 to 0.6)	35.5 (28.7 to 42.2)	0.4 (0.1 to 0.7)	32.6 (25.8 to 40.0)
Syrian Arab Republic	6.8 (4.0 to 10.2)	50.9 (45.3 to 56.3)	7.3 (4.4 to 11.0)	48.5 (42.4 to 54.5)
Tunisia	14.1 (9.3 to 19.5)	45.9 (40.0 to 51.3)	16.3 (11.0 to 22.3)	44.9 (38.6 to 50.5)
Turkey	14.5 (9.6 to 19.6)	54.4 (48.9 to 59.4)	17.1 (11.5 to 23.0)	54.4 (48.4 to 59.6)

Super-region, **region,** or country or territory	**Proportion of OPC deaths attributable to each risk factor (95% UI)**		**Proportion of OPC DALYs attributable to each risk factor (95% UI)**	
	**Alcohol use**	**Smoking**	**Alcohol use**	**Smoking**
United Arab Emirates	18.4 (11.6 to 26.0)	42.5 (33.8 to 50.7)	18.9 (11.9 to 26.7)	41.0 (32.2 to 49.5)
Yemen	4.8 (2.7 to 7.4)	44.4 (37.4 to 50.9)	5.4 (3.0 to 8.3)	41.6 (34.2 to 48.3)
South Asia				
**South Asia**				
Bangladesh	4.5 (0.6 to 8.4)	48.6 (40.4 to 56.3)	4.8 (0.7 to 8.9)	45.3 (36.8 to 53.1)
Bhutan	14.3 (7.1 to 22.2)	30.1 (23.8 to 36.8)	15.5 (7.8 to 23.7)	27.8 (21.5 to 34.6)
India	25.3 (18.0 to 32.8)	39.6 (32.4 to 46.8)	26.9 (19.3 to 34.5)	36.4 (29.2 to 43.5)
Nepal	24.4 (12.8 to 35.4)	45.3 (35.6 to 53.8)	25.5 (13.5 to 37.0)	40.3 (30.7 to 48.6)
Pakistan	10.3 (6.4 to 14.7)	43.2 (36.0 to 51.0)	10.8 (6.7 to 15.2)	39.9 (32.4 to 48.2)
Southeast Asia, East Asia, and Oceania				
**East Asia**				
China	43.6 (34.6 to 52.1)	59.6 (53.7 to 64.8)	45.8 (36.9 to 54.3)	58.9 (52.7 to 64.6)
Democratic People's Republic of Korea	33.2 (24.1 to 41.8)	50.8 (44.1 to 57.5)	35.4 (26.1 to 44.2)	50.8 (43.5 to 58.0)
Taiwan (Province of China)	46.4 (36.1 to 56.1)	60.9 (55.0 to 66.2)	48.1 (37.8 to 57.5)	60.8 (53.9 to 66.6)
**Oceania**				
American Samoa	11.8 (5.5 to 20.2)	53.5 (44.9 to 60.9)	13.5 (6.4 to 22.7)	50.8 (41.9 to 58.9)
Cook Islands	41.2 (30.1 to 51.2)	48.9 (40.9 to 56.6)	43.8 (33.1 to 53.5)	47.5 (39.1 to 55.9)
Fiji	26.0 (18.1 to 33.9)	46.0 (36.8 to 54.2)	27.6 (19.2 to 35.8)	42.4 (33.0 to 50.4)

Super-region, **region,** or country or territory	**Proportion of OPC deaths attributable to each risk factor (95% UI)**		**Proportion of OPC DALYs attributable to each risk factor (95% UI)**	
	**Alcohol use**	**Smoking**	**Alcohol use**	**Smoking**
Guam	25.2 (4.6 to 44.4)	48.2 (39.6 to 56.4)	28.0 (5.4 to 48.2)	46.1 (36.5 to 54.6)
Kiribati	16.8 (5.7 to 30.4)	57.0 (48.1 to 64.5)	18.7 (6.5 to 33.4)	51.1 (41.8 to 59.1)
Marshall Islands	20.6 (10.9 to 31.7)	37.7 (29.4 to 45.7)	22.1 (11.9 to 33.6)	35.2 (27.0 to 43.1)
Micronesia (Federated States of)	17.3 (11.0 to 24.2)	52.1 (41.6 to 61.1)	19.4 (12.6 to 26.7)	49.0 (38.4 to 58.1)
Nauru	33.5 (22.9 to 44.0)	48.2 (37.2 to 57.4)	35.2 (24.1 to 45.8)	44.2 (33.4 to 53.4)
Niue	28.1 (10.7 to 40.9)	44.1 (36.7 to 50.8)	31.1 (12.1 to 44.3)	43.2 (35.3 to 50.8)
Northern Mariana Islands	20.6 (2.5 to 40.3)	53.7 (44.0 to 62.2)	22.1 (2.7 to 42.3)	51.9 (41.8 to 61.1)
Palau	20.5 (7.9 to 32.7)	38.5 (31.0 to 46.2)	22.1 (8.7 to 34.9)	35.6 (27.2 to 43.8)
Papua New Guinea	15.4 (8.3 to 23.7)	40.4 (29.6 to 49.4)	16.5 (9.0 to 25.1)	37.1 (26.8 to 46.5)
Samoa	23.5 (14.5 to 33.1)	54.1 (47.7 to 60.4)	25.4 (16.0 to 35.5)	52.3 (45.2 to 58.9)
Solomon Islands	12.9 (6.4 to 20.7)	43.7 (33.9 to 52.6)	13.9 (6.7 to 22.1)	39.1 (28.9 to 48.5)
Tokelau	21.5 (12.2 to 31.0)	43.6 (35.7 to 51.6)	23.5 (13.8 to 33.5)	41.3 (32.8 to 50.0)
Tonga	15.2 (7.7 to 24.7)	50.4 (43.1 to 57.8)	17.3 (9.0 to 27.6)	46.7 (39.1 to 54.6)
Tuvalu	16.7 (9.5 to 25.2)	47.2 (38.3 to 55.7)	18.9 (10.8 to 28.1)	44.2 (34.8 to 53.1)
Vanuatu	15.0 (8.6 to 22.1)	35.4 (27.1 to 43.3)	16.8 (9.7 to 24.7)	31.3 (23.3 to 39.2)
**Southeast Asia**				
Cambodia	40.9 (30.9 to 50.4)	52.7 (45.8 to 59.3)	43.1 (32.9 to 52.5)	50.2 (42.9 to 57.1)

Super-region, **region,** or country or territory	**Proportion of OPC deaths attributable to each risk factor (95% UI)**		**Proportion of OPC DALYs attributable to each risk factor (95% UI)**	
	**Alcohol use**	**Smoking**	**Alcohol use**	**Smoking**
Indonesia	5.3 (2.0 to 10.5)	52.8 (43.4 to 63.0)	5.9 (2.2 to 11.5)	51.5 (41.5 to 61.8)
Lao People's Democratic Republic	38.1 (26.7 to 49.4)	50.2 (43.4 to 57.4)	40.8 (28.9 to 52.3)	47.4 (40.1 to 55.2)
Malaysia	16.8 (10.0 to 24.6)	48.1 (41.8 to 53.7)	18.3 (11.1 to 26.4)	47.0 (40.4 to 53.1)
Maldives	15.9 (6.1 to 28.8)	61.0 (54.7 to 66.3)	18.0 (7.3 to 31.9)	58.3 (51.6 to 64.1)
Mauritius	39.0 (27.6 to 49.0)	48.3 (40.9 to 55.3)	40.9 (29.4 to 51.2)	47.4 (38.9 to 55.0)
Myanmar	26.9 (17.7 to 35.9)	50.0 (41.4 to 57.8)	29.0 (19.7 to 38.3)	46.3 (37.4 to 54.5)
Philippines	40.8 (31.5 to 50.0)	52.8 (45.3 to 59.7)	43.7 (34.4 to 52.9)	50.2 (42.3 to 57.4)
Seychelles	50.1 (38.1 to 59.9)	57.6 (50.4 to 64.2)	52.3 (40.2 to 62.1)	56.0 (48.2 to 63.2)
Sri Lanka	40.1 (31.7 to 48.4)	44.4 (35.2 to 52.8)	42.6 (34.1 to 51.0)	43.1 (33.2 to 51.6)
Thailand	49.1 (39.9 to 57.3)	53.6 (46.5 to 60.5)	52.2 (43.1 to 60.5)	52.4 (44.6 to 59.8)
Timor-Leste	27.6 (17.6 to 38.2)	49.0 (40.2 to 57.6)	29.4 (19.1 to 40.3)	47.4 (38.4 to 56.3)
Viet Nam	52.5 (42.0 to 61.3)	57.3 (49.6 to 63.9)	53.7 (43.0 to 62.5)	56.3 (48.3 to 63.3)
Sub-Saharan Africa				
**Central sub-Saharan Africa**				
Angola	48.7 (38.7 to 58.0)	37.8 (30.1 to 45.1)	49.4 (39.2 to 58.7)	36.1 (28.4 to 43.6)
Central African Republic	27.8 (16.1 to 40.0)	27.0 (19.1 to 34.5)	28.3 (16.7 to 40.5)	25.8 (18.1 to 33.6)
Congo	40.0 (26.9 to 52.2)	31.5 (23.5 to 39.9)	40.7 (27.7 to 52.9)	30.1 (21.8 to 38.7)

Super-region, **region,** or country or territory	**Proportion of OPC deaths attributable to each risk factor (95% UI)**		**Proportion of OPC DALYs attributable to each risk factor (95% UI)**	
	**Alcohol use**	**Smoking**	**Alcohol use**	**Smoking**
Democratic Republic of the Congo	23.9 (13.1 to 36.5)	25.3 (17.7 to 33.1)	24.7 (13.7 to 37.4)	24.2 (16.9 to 31.8)
Equatorial Guinea	46.5 (34.6 to 57.1)	33.1 (23.3 to 42.9)	47.6 (35.9 to 57.9)	31.5 (21.7 to 41.1)
Gabon	50.9 (38.3 to 61.4)	32.5 (24.2 to 40.9)	51.8 (39.1 to 62.2)	31.7 (23.1 to 40.3)
**Eastern sub-Saharan Africa**				
Burundi	43.6 (33.2 to 53.3)	27.2 (19.3 to 35.1)	44.1 (33.8 to 53.8)	25.2 (17.5 to 32.9)
Comoros	7.5 (2.3 to 14.7)	32.1 (23.5 to 40.8)	7.8 (2.4 to 15.5)	30.1 (21.6 to 39.3)
Djibouti	7.8 (3.4 to 13.8)	45.2 (34.8 to 54.6)	8.1 (3.6 to 14.2)	42.8 (32.5 to 52.4)
Eritrea	19.6 (10.7 to 29.9)	23.7 (17.2 to 30.9)	20.2 (11.1 to 30.7)	23.6 (17.1 to 31.1)
Ethiopia	28.2 (17.8 to 39.8)	13.2 (8.8 to 18.3)	28.9 (18.3 to 40.5)	12.0 (7.9 to 16.7)
Kenya	35.6 (25.3 to 45.4)	28.0 (20.9 to 35.5)	35.9 (25.5 to 46.0)	26.0 (19.1 to 33.3)
Madagascar	22.5 (12.6 to 32.9)	24.4 (17.2 to 31.9)	22.9 (12.8 to 33.4)	23.0 (15.9 to 30.2)
Malawi	29.1 (18.7 to 39.5)	34.3 (24.7 to 43.4)	30.6 (19.9 to 41.1)	30.8 (21.5 to 39.6)
Mozambique	20.6 (11.4 to 31.1)	26.4 (18.3 to 34.4)	21.2 (11.8 to 32.1)	24.3 (16.6 to 31.9)
Rwanda	49.1 (37.9 to 59.2)	39.5 (30.3 to 48.1)	50.2 (38.9 to 60.3)	35.4 (26.9 to 44.2)
Somalia	0.0 (0.0 to 0.0)	24.7 (16.4 to 33.6)	0.0 (0.0 to 0.0)	22.9 (14.9 to 31.7)
South Sudan	6.9 (2.9 to 13.2)	30.8 (21.2 to 40.6)	7.2 (3.0 to 13.6)	28.7 (19.2 to 38.3)
Uganda	52.3 (41.5 to 62.1)	25.8 (17.9 to 34.0)	53.6 (42.8 to 63.5)	23.9 (16.4 to 31.9)

Super-region, **region,** or country or territory	Proportion of OPC deaths attributable to each risk factor (95% UI)		Proportion of OPC DALYs attributable to each risk factor (95% UI)	
	**Alcohol use**	**Smoking**	**Alcohol use**	**Smoking**
United Republic of Tanzania	46.8 (36.1 to 56.3)	39.1 (29.3 to 49.7)	47.3 (36.8 to 56.8)	36.6 (27.0 to 47.2)
Zambia	38.8 (27.0 to 50.6)	30.2 (21.6 to 38.4)	40.2 (28.5 to 51.7)	27.2 (19.0 to 34.9)
**Southern sub-Saharan Africa**				
Botswana	42.0 (32.7 to 51.1)	44.2 (36.0 to 52.5)	43.4 (34.0 to 52.5)	42.1 (33.7 to 50.6)
Eswatini	42.6 (32.9 to 51.8)	23.8 (16.1 to 31.5)	43.8 (34.1 to 53.0)	22.0 (14.6 to 29.6)
Lesotho	38.2 (26.1 to 48.9)	45.4 (38.0 to 52.5)	40.1 (28.0 to 50.9)	44.7 (37.0 to 52.0)
Namibia	45.9 (33.5 to 57.0)	32.5 (23.3 to 40.8)	48.1 (35.5 to 59.2)	29.1 (20.3 to 36.9)
South Africa	46.3 (37.6 to 54.4)	47.3 (39.3 to 54.8)	47.8 (38.9 to 55.9)	46.4 (37.8 to 54.2)
Zimbabwe	30.5 (19.7 to 40.5)	38.0 (30.3 to 45.8)	32.3 (21.3 to 42.5)	35.2 (27.3 to 43.0)
**Western sub-Saharan Africa**				
Benin	28.0 (18.0 to 38.4)	27.7 (20.8 to 35.1)	29.7 (19.2 to 39.9)	25.0 (18.0 to 32.2)
Burkina Faso	46.6 (33.8 to 58.1)	22.5 (15.7 to 29.5)	47.5 (34.9 to 58.7)	22.5 (15.6 to 29.5)
Cabo Verde	41.2 (31.0 to 50.9)	30.0 (22.5 to 37.7)	43.6 (33.3 to 53.7)	30.6 (22.6 to 38.5)
Cameroon	43.5 (33.9 to 53.3)	30.2 (22.9 to 37.3)	44.8 (35.0 to 54.4)	29.5 (21.6 to 36.5)
Chad	26.8 (13.3 to 38.9)	30.6 (22.7 to 38.7)	28.8 (14.5 to 41.2)	28.9 (21.0 to 36.9)
Côte d'Ivoire	42.5 (30.2 to 54.0)	37.2 (29.7 to 44.7)	44.4 (32.0 to 55.5)	37.1 (29.6 to 44.7)
Gambia	30.5 (20.3 to 40.6)	36.7 (29.0 to 44.5)	31.7 (21.3 to 42.2)	35.5 (27.4 to 43.6)

***Super-region*, region,** or country or territory	**Proportion of OPC deaths attributable to each risk factor (95% UI)**		**Proportion of OPC DALYs attributable to each risk factor (95% UI)**	
	**Alcohol use**	**Smoking**	**Alcohol use**	**Smoking**
Ghana	39.3 (28.1 to 49.6)	20.4 (14.4 to 27.2)	40.3 (28.9 to 50.4)	18.2 (12.4 to 24.6)
Guinea	16.2 (9.0 to 24.4)	37.4 (29.5 to 45.4)	17.0 (9.7 to 25.5)	35.7 (27.4 to 43.7)
Guinea-Bissau	32.4 (21.8 to 43.5)	19.6 (14.2 to 25.6)	34.0 (23.4 to 45.1)	18.8 (13.3 to 24.9)
Liberia	35.4 (24.7 to 46.0)	26.2 (18.3 to 34.1)	38.1 (26.8 to 48.6)	24.6 (17.0 to 32.4)
Mali	13.1 (8.0 to 18.8)	30.1 (21.9 to 38.6)	12.9 (8.0 to 18.5)	28.3 (20.2 to 36.6)
Mauritania	0.0 (0.0 to 0.1)	35.0 (26.4 to 44.4)	0.0 (0.0 to 0.1)	35.5 (26.6 to 45.1)
Niger	6.8 (3.0 to 12.0)	18.1 (12.3 to 24.3)	7.3 (3.1 to 12.7)	17.7 (11.8 to 24.0)
Nigeria	36.0 (27.3 to 45.3)	17.1 (12.1 to 22.7)	36.5 (27.7 to 45.8)	16.0 (11.2 to 21.3)
São Tomé and Príncipe	41.0 (30.8 to 51.5)	24.3 (17.5 to 32.0)	42.3 (32.0 to 52.6)	23.0 (16.3 to 30.2)
Senegal	6.8 (3.6 to 10.8)	32.2 (25.3 to 39.3)	8.3 (4.5 to 12.8)	31.9 (25.0 to 39.1)
Sierra Leone	36.0 (25.5 to 45.9)	37.2 (27.9 to 45.4)	37.6 (26.9 to 47.5)	35.7 (26.1 to 43.9)
Togo	29.9 (19.8 to 39.8)	35.0 (28.1 to 42.0)	31.2 (21.2 to 41.0)	33.2 (26.0 to 40.7)

DALYs=disability-adjusted life-years; UI=uncertainty interval.

A Lip and oral cavity cancer

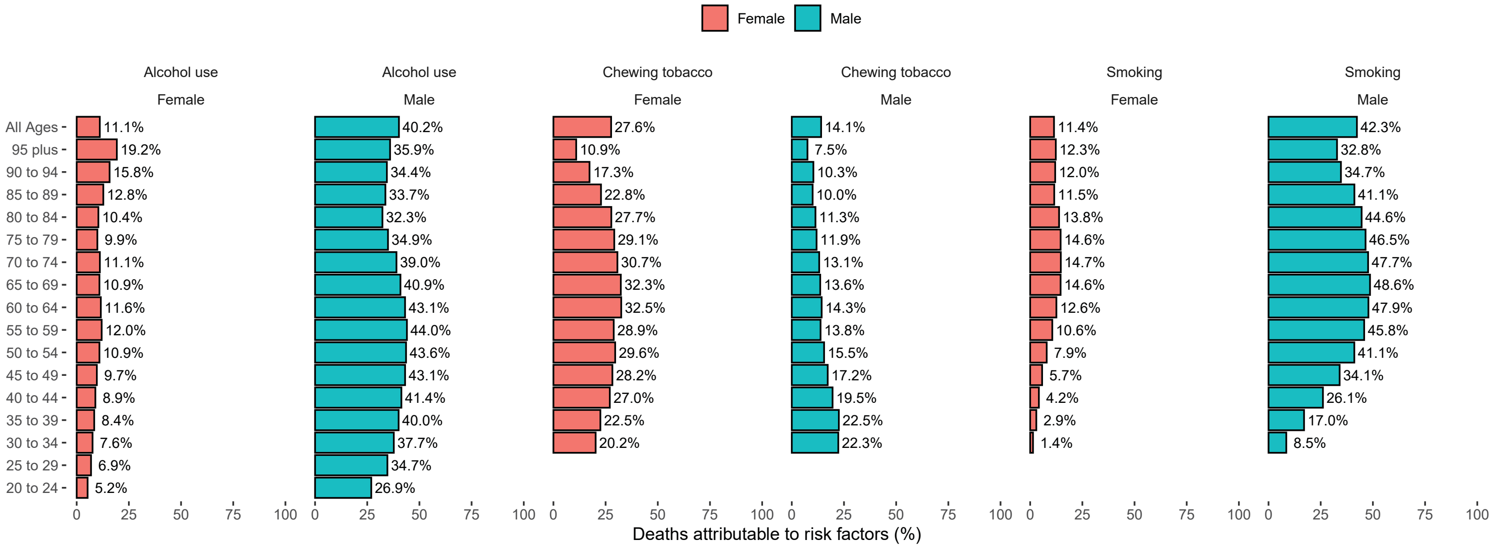

B

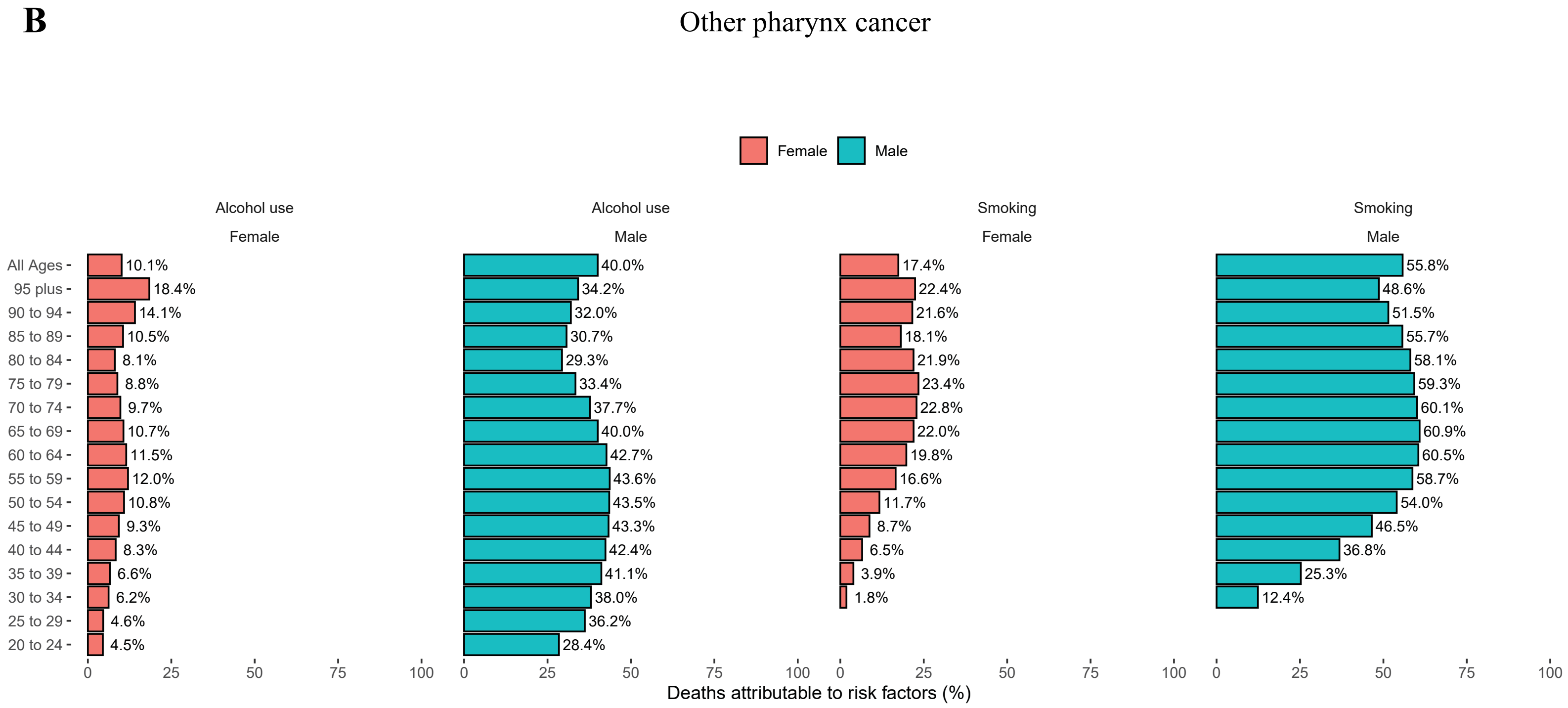

eFigure 18. Proportion of deaths attributable to risk factors for A) Lip and oral cavity cancer and B) Other pharynx cancer for males and females in 2019

The chewing tobacco and smoking risk factors were modeled with lower age restrictions of 30 years in the GBD 2019 study; thus, estimates were not produced for these risk factors in the age groups of 20 to 24 years and 25 to 29 years.
