# Supplementary material for: The Global, Regional, and National Burden of Adult Lip, Oral, and Pharyngeal Cancer in 204 Countries and Territories: A Systematic Analysis for the Global Burden of Disease Study 2019
Source: JAMA Oncol. 2023 Sep 7;9(10):1401–16. doi: 10.1001/jamaoncol.2023.2960 (PMC10485745; doi:10.1001/jamaoncol.2023.2960)
Supplement: Supplement 2. — Data Sharing Statement [file jamaoncol-e232960-s002.pdf]

Data Sharing Statement

Cunha. The Global, Regional, and National Burden of Adult Lip, Oral, and Pharyngeal Cancer in 204 Countries and Territories. *JAMA Oncol*. Published September 07, 2023. doi:10.1001/jamaoncol.2023.2960

Data
Data available: Yes
Data types: Deidentified participant data, Other (please specify)
Additional Information: Some of the data that will be made available are aggregate data.
How to access data: Data available for the GBD 2019 Study are available, in compliance with GATHER requirements, on the Global Health Data Exchange (GHDx) and online at: https://ghdx.healthdata.org/gbd-2019
When available: With publication

Supporting Documents
Document types: Statistical/analytic code
How to access documents: GBD 2019 data can be accessed at: http://ghdx.healthdata.org/gbd-2019/code
When available: With publication

Additional Information
Who can access the data: Publicly available online
Types of analyses: Publicly available online
Mechanisms of data availability: Publicly available online
Any additional restrictions: The GBD Study complies with data confidentiality and data use agreements required by data providers worldwide. While we aim to be as transparent as possible, some data may be restricted per provider requirements.
